# The role and mechanism of “eight famous herbals in Zhejiang” in cancer via network pharmacology and experimental validation

**DOI:** 10.3389/fonc.2024.1475000

**Published:** 2024-11-15

**Authors:** Ziheng Ni, Hao Zhang, Fengyun Chen, Mengjie Yang, Liting Yang, Yuan Zhou, Xianmin Zhou, Jiayi Guo, Xinyu Rao, Jiaqi Cen, Qun Lv, Jianjun Wang, Lailing Du, Gongxing Chen, Shuiping Liu

**Affiliations:** ^1^ School of Pharmacy, Hangzhou Normal University, Hangzhou, Zhejiang, China; ^2^ Department of Respiratory Medicine of Affiliated Hospital, Hangzhou Normal University, Hangzhou, Zhejiang, China; ^3^ Key Laboratory of Pollution Exposure and Health Intervention of Zhejiang Province, Shulan International Medical College, Zhejiang Shuren University, Hangzhou, China; ^4^ Key Laboratory of Elemene Class Anti-Cancer Chinese Medicines, Engineering Laboratory of Development and Application of Traditional Chinese Medicines, Collaborative Innovation Center of Traditional Chinese Medicines of Zhejiang Province, Hangzhou Normal University, Hangzhou, Zhejiang, China

**Keywords:** Zhe-Ba-Wei, cancer, active ingredients, drug target, network pharmacology

## Abstract

In recent years, some components and active ingredients from the herbal formula “eight famous herbals in Zhejiang” (Zhe-Ba-Wei) have been reported to possess antitumor properties. However, there is still no systemic study on the role and mechanism of Zhe-Ba-Wei in cancer. To systematically investigate the anticancer efficacy of Zhe-Ba-Wei, we first identified 17 reported active ingredients with gene targets associated with various types of tumors. Second, we screened these active ingredients and their responding multiple shared targets by analyzing the convergence of diverse and tumor-specific target sites and identified four crucial active ingredients (ferulic acid, quercetin, rutin, luteolin), which were characterized by 27 overlapping gene targets. Third, these 27 gene targets were subsequently mapped onto the Kyoto Encyclopedia of Genes and Genomes (KEGG) pathway and Gene Ontology term, and among the 27 total potential targets, 12 were involved in plasma membrane function. Fourth, we investigated the binding affinities between the four crucial active ingredients and their potential targets such as EGFR and MET, both of which are well-known oncogenes in various cancers. Subsequently, an investigation of the computational ADMET properties showed that most of these four ingredients exhibited good ADMET properties. Finally, we found that three active ingredients (ferulic acid, luteolin, and quercetin) could inhibit the proliferation of non-small cell lung cancer cells and decrease the protein expression of EGFR in a concentration-dependent manner. All these results shed light on the bioactive components, pharmacological effects, and drug development and utilization of Zhe-Ba-Wei, aiming to provide useful support for its further research and clinical application.

## Introduction

The traditional Chinese medicine “eight famous herbals in Zhejiang” (well-known as Zhe-Ba-Wei), consisting of *Atractylodes macrocephala* Koidz., *Paeonia lactiflora* Pall., *Fritillaria thunbergii* Miq., *Chrysanthemum morifolium* Ramat., *Corydalis yanhusuo* W., *Scrophularia ningpoensis* Hemsl., *Ophiopogon japonicus* (Thunb.) Ker-Gawl., and *Curcuma wenyujin* Y.H. Chen et C. Ling, is a famous herbal formula in Zhejiang (1, 2). Recently, increasing evidence shows that traditional Chinese medicine has been widely used in the treatment of various types of cancer, with reduced drug resistance and side effects (3–6).

Modern pharmacological studies have shown that some components or active ingredients of Zhe-Ba-Wei can exert therapeutic activities on various cancers such as malignant brain glioma (7), leukemia (8, 9), non-small cell lung cancer (NSCLC) (10–12), breast cancer (13), bladder cancer (14), prostate cancer (15, 16), colorectal cancer (17–19), cervical cancer (20), and gastric cancer (21–23). Taking *A. macrocephala* Koidz. as an example, it has a certain inhibitory effect on bladder cancer (24), colorectal cancer (25), breast cancer (26), and pancreatic cancer (27). Most recently, an increasing number of bioactive ingredients and their potential targets have been identified with the improvement of separation and identification technology (28–31). For instance, *C. wenyujin* Y.H. Chen et C. Ling, which is one component of Zhe-Ba-Wei exerting therapeutic activities on cancer, contains mainly volatile oils (elemene, furandiene, curcumenol, gemarone, diterpenoid C, curcumin), curcuminoid, and other relative active compounds (32–36).

Although several articles revealed the biofunction of the components or active ingredients of Zhe-Ba-Wei in various cancers, there is a limited systemic study on the therapeutic effects of Zhe-Ba-Wei as an entirety in cancer. This study primarily utilized network pharmacology inference to explore the potential therapeutic effects of Zhe-Ba-Wei on malignant tumor cells. Meanwhile, we analyzed and provided relevant information on the selected active ingredients and their potential targets, as well as conducted a prognostic analysis of these targets in various tumors. Furthermore, we conducted experiments to verify the anticancer effect of selected bioactive ingredients and their corresponding targets.

## Materials and methods

### Data retrieval

We retrieved pertinent information regarding the active ingredients of Zhe-Ba-Wei with anticancer properties from PubMed (https://pubmed.ncbi.nlm.gov), using *Atractylodes macrocephala* Koidz., *Paeonia lactiflora* Pall., *Fritillaria thunbergii* Miq., *Chrysanthemum morifolium* Ramat., *Corydalis yanhusuo* W., *Scrophularia ningpoensis* Hemsl., *Ophiopogon japonicus* (Thunb.) Ker-Gawl., and *Curcuma wenyujin* Y.H. Chen et C. Ling as keywords.

### Prediction of the potential targets of active ingredients

SwissTargetPrediction (http://www.wisstargetprediction.ch) is a free database based on compound structures to predict the potential targets for active ingredients. OMIM (http://www.omim.org/) and GeneCards (http://www.genecards.org) are searchable and integrated human genes database that provide concise genomic information on all known and predicted human genes. Discovery Studio 2019 is a professional life science molecular simulation software applied to probe the binding affinity between active ingredients and potential disease targets through semiflexible docking. Discovery Studio 2019 receptors and ligands were obtained from the PDB database (https://www.rcsb.org/) and PubChem database (https://pubchem.ncbi.nlm.nih.gov/), respectively. The PDB codes of the receptor proteins are MET:5EOB and EGFR:1M17. These receptor proteins were treated in Discovery Studio 2019 as follows:1) delete water, 2) clean protein, 3) define receptor, 4) define binding sites, and 5) delete ligand.

Ligands were also processed in Discovery Studio 2019. We need to select “prepare ligands” and then “apply forcefield” operation on the prepared ligands. “CDOCKER Energy” was used for assessing the docking efficiency of these active ingredients, estimating their ability to form stable interactions.

### Forecasting ADMET

The Discovery Studio 2019 software was used to predict the ADMET (absorption, distribution, metabolism, excretion, and toxicity) properties of active ingredients including aqueous solubility, blood–brain barrier penetration, human intestinal absorption, hepatotoxicity, cytochrome P450 2D6 inhibition, and plasma protein binding. Similarly, the Discovery Studio 2019 was employed to predict the toxicity of active ingredients. The “TOPKAT” module was utilized to forecast toxicity parameters such as rodent carcinogenicity, mutagenicity, aerobic biodegradability, and rat oral LD_50_.

### Cell proliferation inhibition assays

Human NSCLC cell lines (A549, PC9, and H1975) were from the ATCC and authenticated by short tandem repeat DNA profiling analysis. All cells were cultured with RPMI 1640 medium (Gibco BRL, USA) containing 10% fetal bovine serum (FBS) and incubated in a cell incubator at 37°C, 5% CO_2_. For the cell proliferation inhibition assays, 3,000 NSCLC cells in a 100-μL medium were inoculated into each well of a 96-well plate, together with an equal amount of PBS in the marginal wells to avoid edge effect. After treating with different concentrations of drugs for the indicated hours, 100 μL of the diluted CCK-8 solution was added into each well and incubated for 1–2 h according to the manufacturer’s instructions. Finally, the OD value was evaluated by the Multiskan FC microplate reader (Thermo Scientific, USA), and GraphPad 7.0 was used to calculate and analyze the IC_50_ of the drug (37). All experiments were repeated at least three times.

### Western blot

The cells were collected and lysed with RIPA buffer (#P0013B, Beyotime, China). Twenty micrograms of total protein was used for SDS-PAGE after concentration measurement and then transferred to a PVDF membrane at 250 mA for an appropriate time. After blocking with 5% skim milk for 1 h, the membrane was incubated overnight with primary antibody (1:1,000). Subsequently, it was washed with PBST buffer and incubated with HRP-conjugated secondary antibody (1:1,000) for 1 h. Finally, the ECL kit (#1705061, Bio-Rad, USA) was used and visualized with ChemiDoc Imaging System (Bio-Rad, USA) (38).

## Results

### Prediction of potential molecular targets of various active ingredients

Numerous active ingredients associated with antitumor properties have been discovered in Zhe-Ba-Wei as reported in the literature. Taking cancer-related ingredient as the criterion, five active ingredients from *A. macrocephala* Koidz., one from *P. lactiflora* Pall., five from *C. morifolium* Ramat., five from *C. yanhusuo* W., and one from *C. wenyujin* Y.H. Chen et C. Ling were selected (**Table 1**). It is worth noting that no literature has reported any active compounds with anticancer potential in *F. thunbergii* Miq., *S. ningpoensis* Hemsl., and *O. japonicus* (Thunb.) Ker-Gawl. Subsequently, the SMILES number of these 17 active ingredients were identified in PubChem, and the potential targets for various active ingredients were obtained through the online server SwissTargetPrediction (39) (**Supplementary Table S1**).

**Table 1:** Active ingredients in traditional Zhe-Ba-Wei.

Herb name	*Atractylodes macrocephala* Koidz.	*Paeonia lactiflora* Pall.	*Chrysanthemum morifolium* Ramat.	*Corydalis* *yanhusuo* W.	*Curcuma wenyujin* Y.H. Chen et C. Ling
**Active ingredients**	Atractylenolide I Atractylenolide II Atractylenolide III Coumarin Atractylon	Benzoylpaeoniflorin	Chlorogenic acid Luteolin Quercetin Rutin Ferulic acid	Tetrahydroberberine Tetrahydrocoptisine Berberine Epiberberine Coptisine	β-Elemene

To investigate the overlapping targets of active ingredients, these 17 active ingredients were paired together to select the overlapping targets influenced by every two active ingredients. Subsequently, the overlapping targets of every two active ingredients were amalgamated, resulting in the formation of a merged group encompassing every three active ingredients’ overlapping targets. Similarly, the subset comprising the three active components with a greater number of overlapping targets underwent pairwise pairing to obtain a cohort of four active ingredients with the highest degree of shared targets. However, there were limited overlapping targets for every five active ingredients. Thus, based on the screening process, a group of four active ingredients, namely, ferulic acid, rutin, quercetin, and luteolin (referred to as group 1), characterized by a substantial number of overlapping targets, has been successfully identified (**Table 2**). Interestingly, all four active ingredients come from the same herbal *C. morifolium* Ramat. Meanwhile, an additional group, denoted as group 2 (such as palmatine, coptisine, tetrahydrocoptisine, and berberine), derived from the same herbal *C. yanhusuo* W., displayed a notable intersection of targets and possessed a considerable number of overlapping targets (**Table 2**).

**Table 2:** Overlapping targets of active ingredients in group 1 and group 2.

	Group 1	Group 2
**Active ingredients**	Ferulic acid Rutin Quercetin Luteolin	Tetrahydrocoptisine Berberine Epiberberine Coptisine
**Overlapping targets**	NOX4, AKR1B1, MAOA, CA2 ALOX5, CA7, GLO1, APP, GSK3B MMP9, CA12, MMP2, CA4, CA1 CYP1B1, ABCG2, ALOX15, CA9 EGFR, F2, PYGL, CA3, CA6, CA14 MET, CA13, KDM4E	SIGMAR1, CDK2, CHEK1 HTR2B, ADRA2C, PIK3CD PIK3CG, ACHE, ABL1, CHEK2 XBP1, ADRA2B, SAE1, MAOB AURKB, MAPKAPK2, UBA2

**Figure 1:**
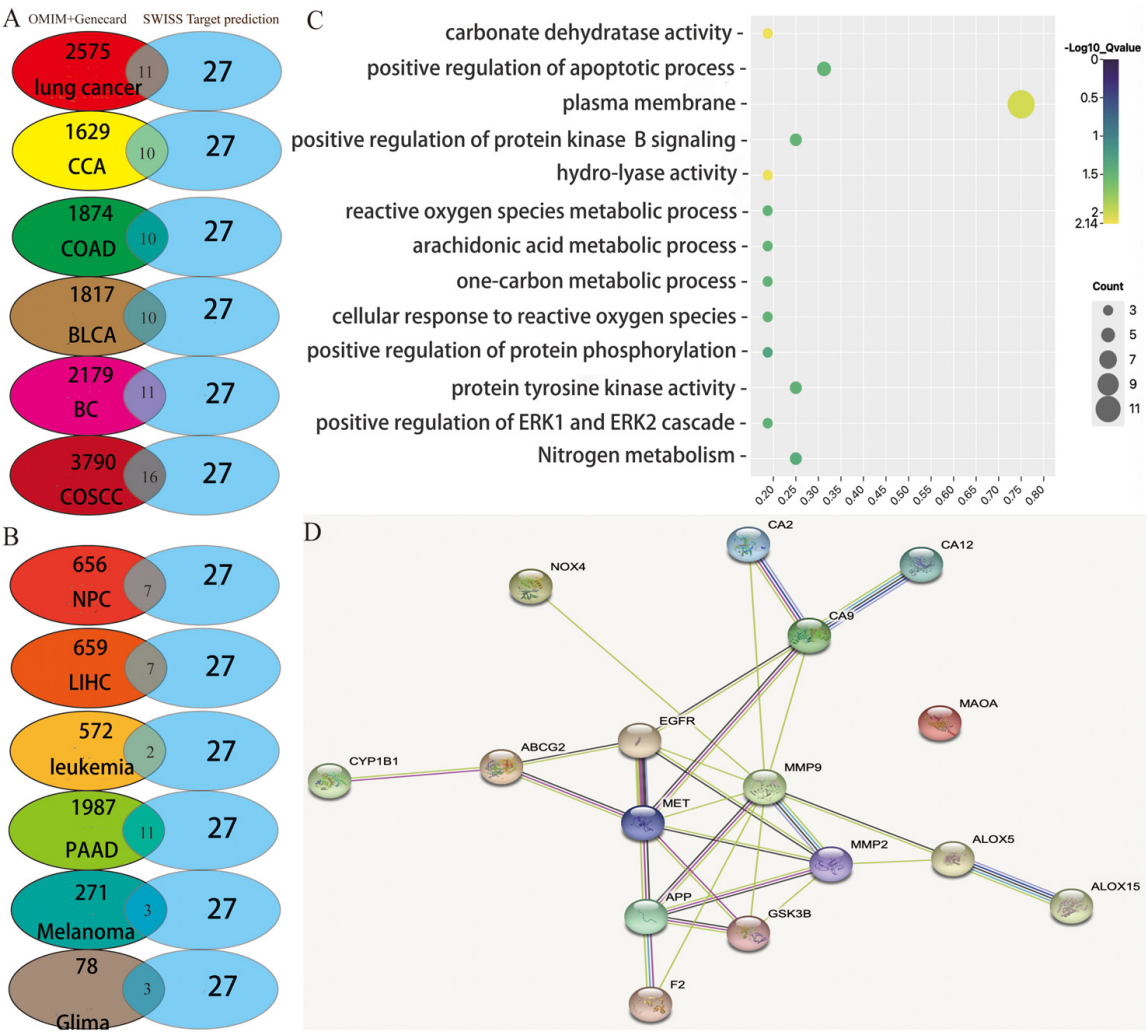
Potential target prediction and analysis of the KEGG pathway, GO_BP, and target–target interaction. **(A, B)** Overlapping target analysis by Venn diagrams; **(C)** KEGG pathway enrichment and GO_biological process analysis of the overlapping targets; **(D)** target–target interaction of the overlapping targets analyzed by STRING.

Based on the literature reported, the antitumor properties of diverse active ingredients primarily target the following 12 types of cancers: lung cancer, cervical cancer, colorectal cancer, bladder cancer, breast cancer, oral squamous cell carcinoma, nasopharyngeal carcinoma, hepatocellular carcinoma, leukemia, pancreatic cancer, melanoma, and glioma. Henceforth, all the reported gene targets that correlated with each of these 12 types of cancer were identified from the OMIM (40) and GeneCards databases (41), respectively (**Supplementary Table S2**). Next, we analyzed the overlapping targets between the four active ingredients (group 1 or group 2) and cancer disease using Venn diagrams. As a result, the numbers of overlapping targets between group 1 and different cancers are shown in **Figures 1A, B**, but the numbers of overlapping targets between group 2 and cancer disease were limited for further analysis. Thus, we focus our study on the overlapping targets between group 1 and cancers.

Through analysis of the Venn diagrams, it was observed that when the active ingredients of group 1 acted in concert, they shared nine common therapeutic targets among nine types of cancers: lung cancer, cervical cancer, colorectal cancer, bladder cancer, breast cancer, oral squamous cell carcinoma, nasopharyngeal carcinoma, hepatocellular carcinoma, and pancreatic cancer. These targets included arachidonate 5-lipoxygenase (ALOX5), glycogen synthase kinase 3 beta (GSK3B), matrix metalloproteinase-9 (MMP9), matrix metalloproteinase-2 (MMP2), recombinant cytochrome P450 1B1 (CYP1B1), ATP-binding cassette superfamily G member 2 (ABCG2), carbonic anhydrase IX (CA9), epidermal growth factor receptor (EGFR), and mesenchymal to epithelial transition factor (MET). These nine targets have been extensively studied and are known to play pivotal roles in tumor proliferation, drug resistance, and metastasis. Therefore, the potential of these active ingredients in treating tumors warrants further exploration in future studies. Nevertheless, melanoma and glioblastoma share only two overlapping targets (EGFR and MMP2). Furthermore, the active ingredients of group 1 exhibited seven additional targets—F2, ALOX15, NOX4, MAOA, APP, CA2, and CA12—that are distinct from the nine aforementioned cancer types.

Consequently, the complete array of target genes of these four active ingredients was charted into the KEGG pathways and subjected to analysis utilizing Gene Ontology (GO) terms through the DAVID tool (**Figure 1C**). This approach was undertaken to explore the signaling pathways and biological processes through which these four active ingredients exert their anticancer effects.

A false discovery rate threshold of less than or equal to 0.05 was employed to sieve out the signaling pathways and biological processes of higher reliability. Interestingly, we solely acquired the nitrogen metabolism signaling pathway along with 12 additional GO terms (**Figure 1C**). Among them, the GO term exhibiting the strongest correlation with these targets was involved in the plasma membrane. Twelve among the 17 total potential targets (namely, ABCG2, MET, NOX4, APP, ALOX15, CA12, CA2, CA9, F2, EGFR, GSK3B, and MMP2) were associated with this particular biological function. It suggested that the active ingredients we screened potentially exert their anticancer effects by selectively targeting proteins situated on the cellular plasma membrane. In addition, the interconnections among target proteins were investigated using STRING (https://cn.string-db.org/), a highly convenient web platform that facilitates the retrieval of known and predicted protein interactions. The outcomes obtained from the aforementioned website unveil a notable level of interplay between the target proteins, excluding monoamine oxidase A (MAOA) (**Figure 1D**), which displays relatively lower interaction levels.

**Figure 2:**
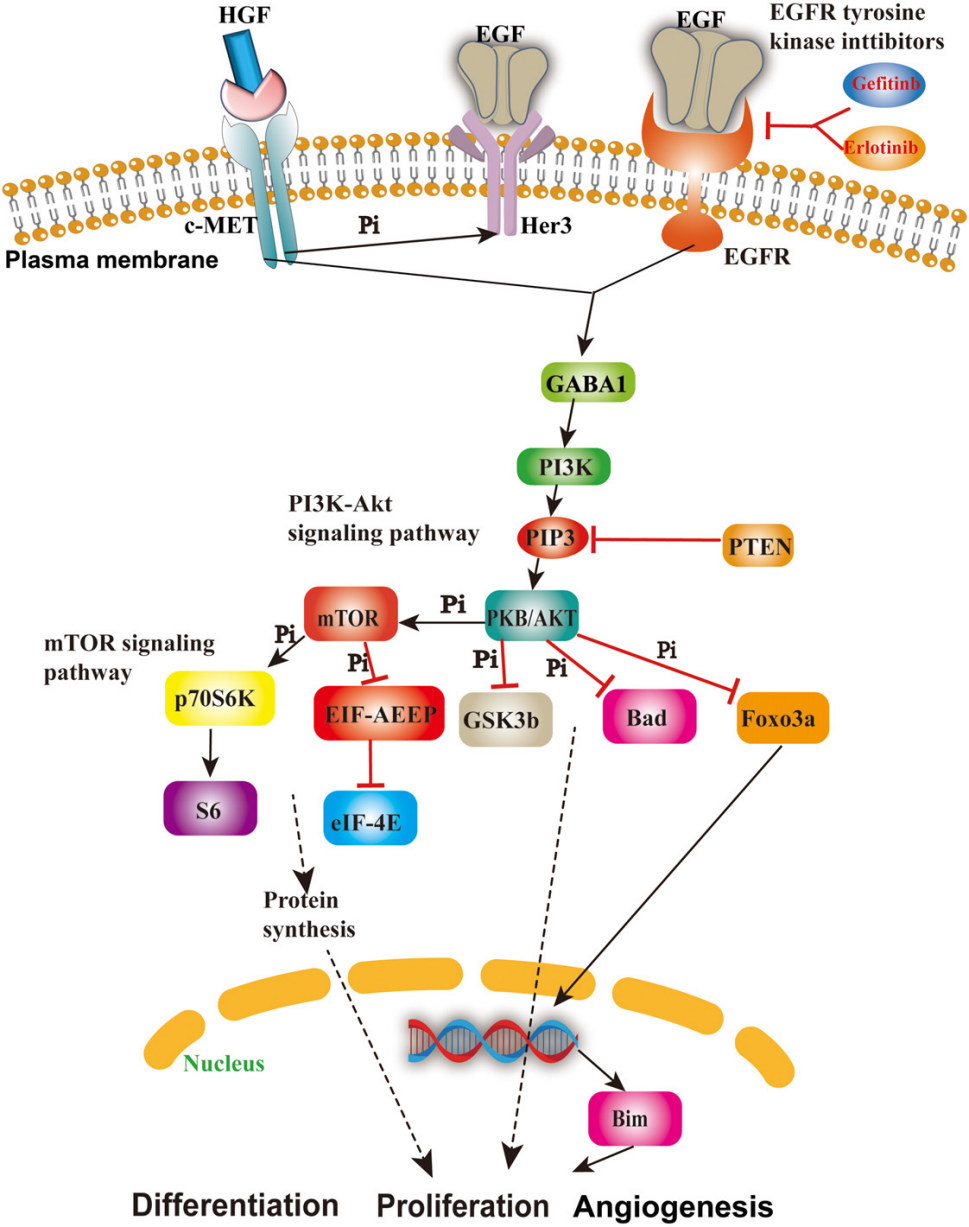
The role and mechanism of the EGFR/MET-mediated signaling pathway in cancer cells. This figure shows the important cascades associated with the EGFR/MET-mediated signaling pathway involved in cell angiogenesis, proliferation, and apoptosis.

Considering that both EGFR (42) and MET (43) reside on the cellular membrane, we have opted to pursue further investigation into these two proteins as potential drug targets. Their localization on the cell membrane underscores their significance and motivates our continued research efforts in this direction. It is well-established that EGFR and its associated pathways significantly contribute to tumor progression. EGFR participates in the ERBB signaling pathway, whereas MET is expressed in its alternative pathway, and GSK3B is situated in its downstream pathway known as the PI3K–Akt signaling pathway (**Figure 2**) (44), which plays critical roles in NSCLC, breast cancer, glioma, etc. According to previous reports, EGFR on the plasma membrane is the receptor for epithelial growth factor (EGF), and mutation or overexpression of EGFR is associated with tumor cell proliferation, angiogenesis, invasion, metastasis, and apoptosis (45). MET is present in the bypass pathway of the ERBB signaling pathway and is a proto-oncogene that interacts with EGFR, thereby promoting drug resistance of the tumor (46). GSK3B is involved in the PI3K–Akt signaling pathway, which can promote the proliferation, differentiation, and angiogenesis of tumor cells (47, 48). In summary, the three proteins mentioned above are most likely the important targets of the four active ingredients we screened; therefore, we speculate that these four active ingredients may have expected therapeutic effects on NSCLC and breast cancer by inhibiting the EGFR and MET (49, 50) in the following text.

### Molecular docking

**Figure 3:**
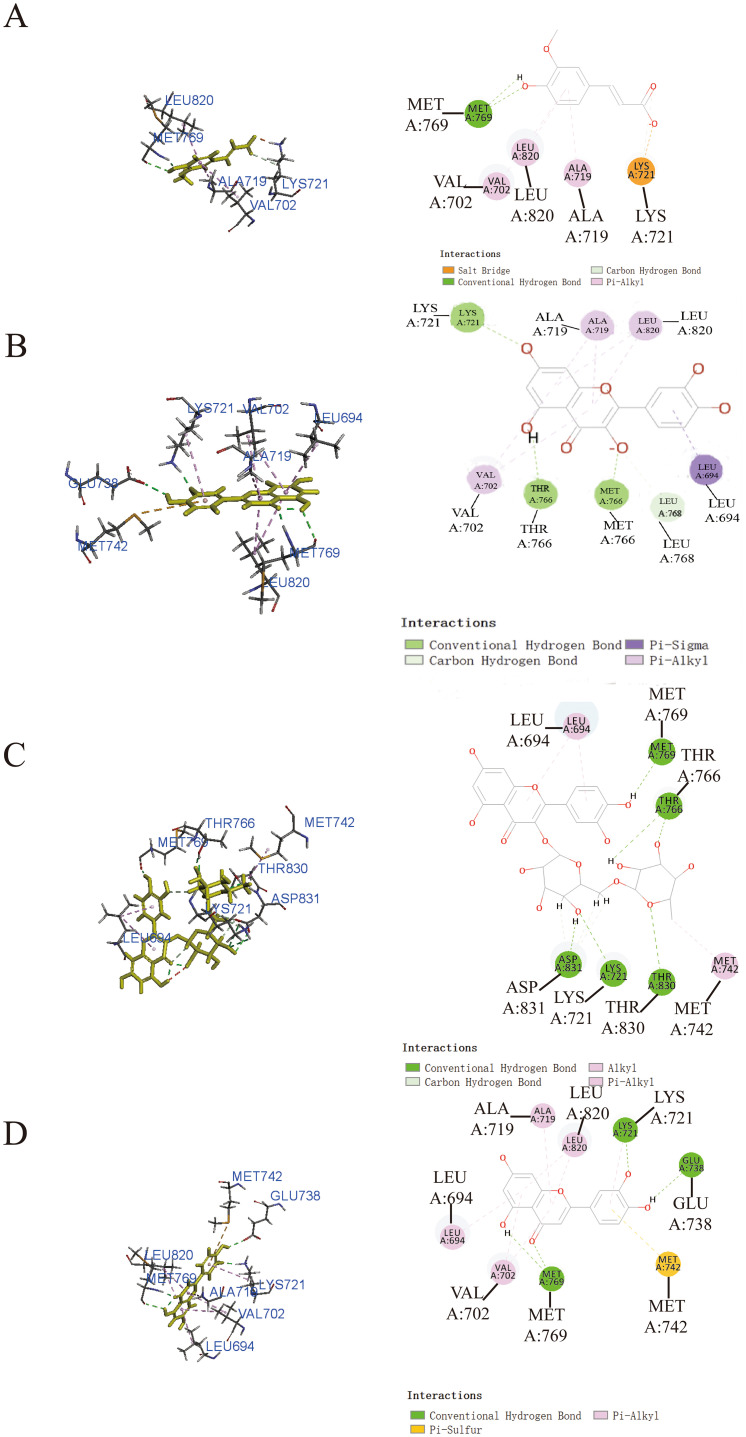
Prediction of the interaction between EGFR and indicated drug by molecular docking. Molecular docking diagrams of ferulic acid **(A)**, quercetin **(B)**, rutin **(C)**, and luteolin **(D)** with the target protein EGFR.

To enhance our understanding of the potential interactions between small molecule active components and protein receptor targets, we employed molecular docking techniques utilizing Discovery Studio 2019. The results from this analysis revealed that ferulic acid (**Figure 3A**), quercetin (**Figure 3B**), and luteolin (**Figure 3D**) exhibited remarkable binding affinity toward the EGFR protein, as evidenced by their -CDOCKER energy values of 25.3315 kcal/mol, 36.8433 kcal/mol, and 36.801 kcal/mol, respectively. These findings highlighted the significant potential of EGFR as a target for these compounds. In addition, rutin (**Figure 3C**) exhibited relatively low binding affinity to EGFR protein with -CDOCKER energies of 3.6186 kcal/mol.

**Figure 4:**
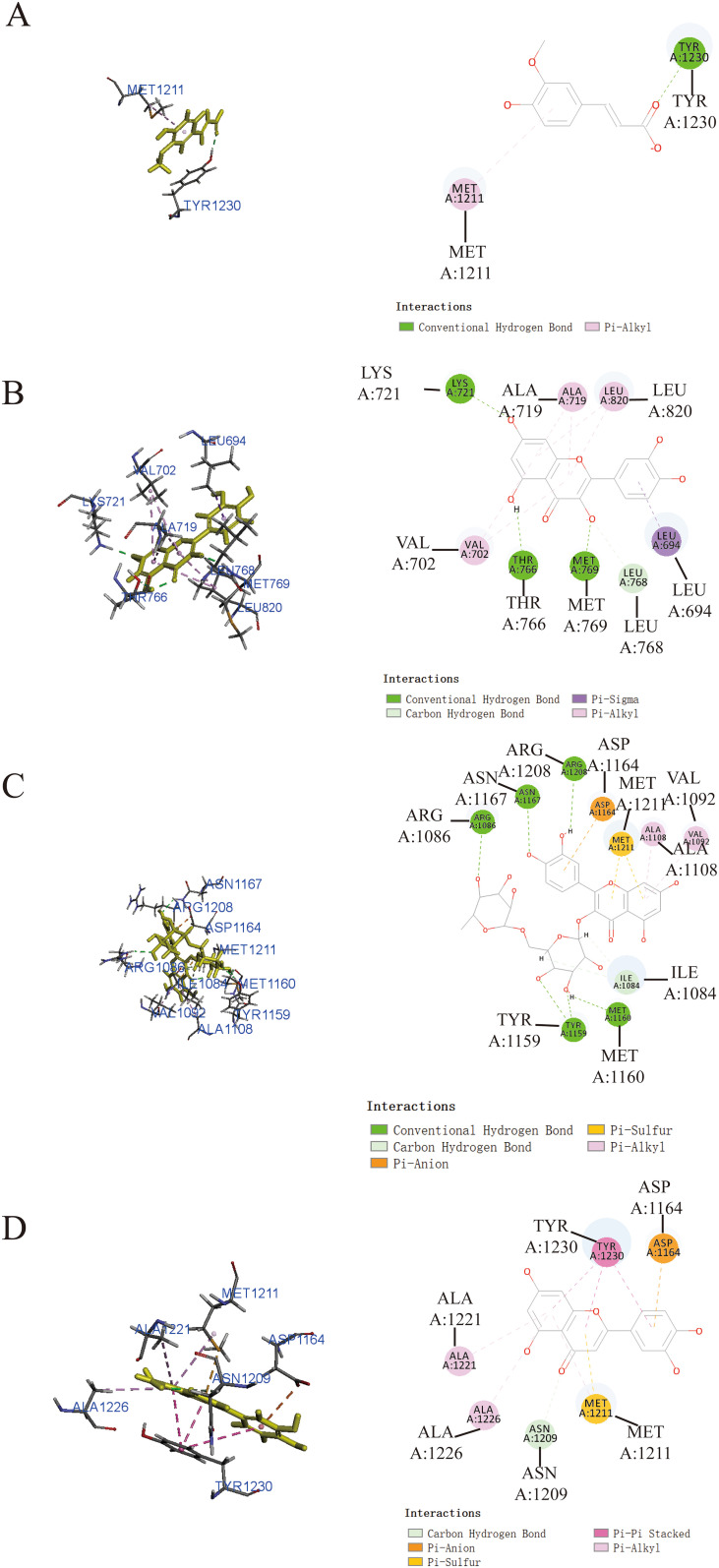
Prediction of the interaction between MET and indicated drug by molecular docking. Molecular docking diagrams of ferulic acid **(A)**, quercetin **(B)**, rutin **(C)**, and luteolin **(D)** with the target protein MET.

Additionally, it was worth noting that ferulic acid (**Figure 4A**), quercetin (**Figure 4B**), and luteolin (**Figure 4D**) also exhibited notable binding affinity to MET, as supported by the theoretical analysis utilizing -CDOCKER energy values of 23.2231 kcal/mol, 33.6036 kcal/mol, and 35.1918 kcal/mol, respectively. In addition, rutin (**Figure 4C**) exhibited relatively low binding affinity to MET protein with -CDOCKER energies of 1.55349 kcal/mol.

### Prediction of ADMET properties of the four active ingredients

The *in-silico* prediction of ADMET properties stands as a pivotal stage in lead compound discovery and drug development, enabling substantial savings in time, manpower, material resources, and financial resources. The ADMET properties of the four active ingredients were individually predicted using the four most used anticancer drugs in clinics (namely, pemetrexed, osimertinib, gemcitabine, and taxol) as control, employing ADMET descriptors and the Lipinski filter tools provided by Discovery Studio 2019. The analysis provided essential physical parameters, such as aqueous solubility, blood–brain barrier penetration, human intestinal absorption, hepatotoxicity, cytochrome P450 2D6 inhibition, and plasma protein binding.

**Figure 5:**
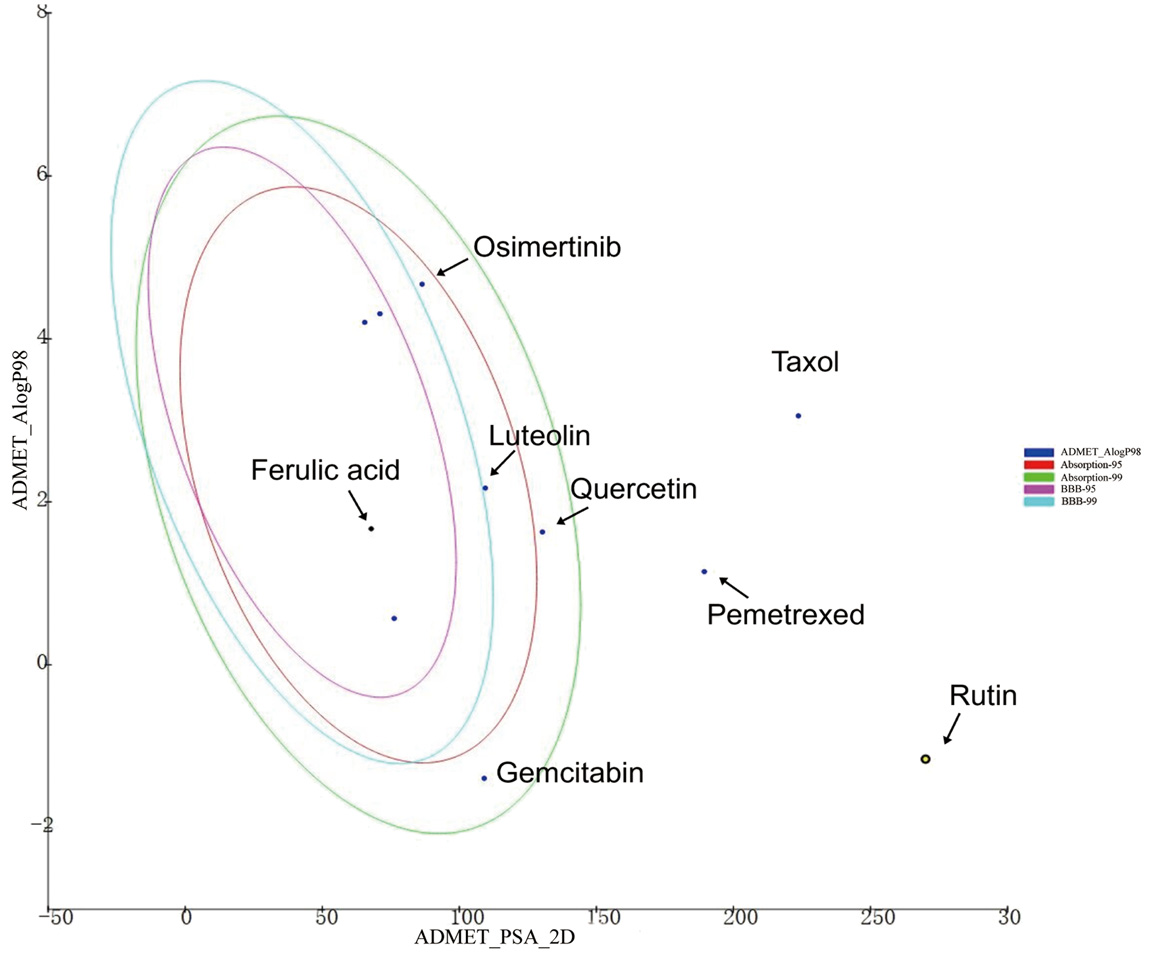
The ADMET plot of indicated drugs plotted by ADMET_PSA_2D vs. ADMET _AlogP98. The dark blue dots represent the AlogP98 of each drug. The red and green ellipses represent the 95% and 99% confidence intervals of the blood–brain barrier (BBB) permeability model, respectively, and the rose red and light blue ellipses represent the 95% and 99% confidence intervals of the human intestinal absorption model, respectively.

As shown in **Figure 5**, ferulic acid exhibited the most favorable ADMET characteristics among the four bioactive constituents. The parameters were evaluated according to Lipinski’s rule of five (ROF), which serves to assess the drug-like characteristics of the compounds. Furthermore, ferulic acid, luteolin, and quercetin exhibited reasonably favorable absorption parameters. However, the analysis results indicated that rutin’s absorption and blood–brain barrier permeability were not ideal.

Among the four active ingredients, rutin stood out with an Alog*P* value below 0, indicating its inferior blood–brain barrier capacity and favorable water solubility (**Table 3**). Except rutin, the other three active ingredients had good absorption in the body (**Table 4**). Meanwhile, we predicted the hepatotoxicity, cytochrome P450 2D6 inhibition, and plasma protein binding properties of these four active ingredients. Similar to many other anticancer drugs, most of them showed no inhibitory effects to cytochrome P450 2D6, except luteolin (**Table 5**). Moreover, quercetin, rutin, and luteolin exhibited low rates of plasma protein binding, indicating their favorable *in-vivo* activity and ability to undergo transmembrane transport, metabolism, and excretion (**Table 6**). Furthermore, quercetin, rutin, and luteolin demonstrated certain levels of hepatotoxicity, but comparatively lower than that of osimertinib and pemetrexed (**Table 7**). In addition, ferulic acid exhibited low hepatotoxicity similar to taxol (**Table 7**).

**Table 3:** Blood–brain barrier (BBB) penetration and human intestinal absorption prediction of group 1 and reference.

Compounds	BBB	BBB level	Absorption level	AlogP98	PSA_2D
**Ferulic acid**	−0.712	3	0	1.669	67.861
**Quercetin**	–	4	1	1.63	130.308
**Rutin**	–	4	3	−1.158	270.106
**Luteolin**	–	4	0	2.168	109.492
**Pemetrexed**	–	4	3	1.144	189.373
**Osimertinib**	–	4	0	4.671	86.426
**Gemcitabine**	–	4	1	−1.394	109.077
**TAX (taxol)**	–	4	3	3.055	223.712

**Table 4:** Aqueous solubility prediction of group 1 and reference drugs.

Compounds	Solubility	Solubility level
**Ferulic acid**	−1.592	4
**Quercetin**	−2.633	3
**Rutin**	−6.182	1
**Luteolin**	−2.856	3
**Pemetrexed**	−3.488	3
**Osimertinib**	−5.694	2
**Gemcitabine**	−0.844	4
**TAX (taxol)**	−3.515	3

**Table 5:** Cytochrome P450 2D6 inhibitor prediction of group 1 and reference drugs.

Compounds	CYP2D6	Prediction	Applicability #MD	Applicability #Md *P*-value
**Ferulic acid**	−7.42105	FALSE	11.8063	0.00446
**Quercetin**	−0.816563	FALSE	8.89275	0.43146
**Rutin**	−2.54544	FALSE	15.866	2.1E-07
**Luteolin**	1.55916	TRUE	8.42243	0.61771
**Pemetrexed**	−10.306	FALSE	15.0908	1.6E-06
**Osimertinib**	−4.75041	FALSE	19.5013	1.3E-11
**Gemcitabine**	−4.0935	FALSE	15.3695	7.8E-07
**TAX (taxol)**	−9.84616	FALSE	12.6519	0.00069

**Table 6:** Plasma protein binding (PPB) rate prediction of group 1 and reference drugs.

Compounds	PPB	Prediction	Applicability #MD	Applicability #Md *P*-value
**Ferulic acid**	−1.45379	TRUE	11.9462	0.10654
**Quercetin**	−5.89414	FALSE	10.7601	0.61132
**Rutin**	−15.9101	FALSE	13.0578	0.0046
**Luteolin**	−2.4864	FALSE	11.3257	0.32408
**Pemetrexed**	−15.3292	FALSE	15.4986	3.8E-08
**Osimertinib**	−5.82259	FALSE	19.2739	2.01E−20
**Gemcitabine**	−25.4599	FALSE	10.9394	0.51845
**TAX (taxol)**	19.7216	TRUE	15.1283	3.3E-07

**Table 7:** Hepatotoxicity prediction of group 1 and reference drugs.

Compounds	Hepatotoxic	Prediction	Applicability #MD	Applicability #Md *P*-value
**Ferulic acid**	−8.58263	FALSE	9.6793	0.17015
**Quercetin**	1.15197	TRUE	7.57486	963553
**Rutin**	−3.06436	TRUE	10.2772	0.04755
**Luteolin**	0.52571	TRUE	8.58253	0.66647
**Pemetrexed**	1.63974	TRUE	12.5966	1.2E-05
**Osimertinib**	2.59551	TRUE	15.515	1.2E-12
**Gemcitabine**	−2.15171	TRUE	8.43163	0.73513
**TAX (taxol)**	−9.53069	FALSE	16.426	3.21E-15

Next, the toxicity of the four ingredients was calculated through the “TOPKAT” module in Discovery Studio 2019. Predicted data on rodent carcinogenicity, mutagenicity, and rat oral LD_50_ toxicity parameters were obtained. Quercetin exhibited the lowest levels of rodent carcinogenicity, but its rat oral LD_50_ was recorded as 175.2 mg/kg. On the other hand, ferulic acid exhibited the highest level of rodent carcinogenicity although its rat oral LD_50_ stood at 651.1 mg/kg, which is considered remarkable performance. In summary, ferulic acid had the most favorable parameters for drug development among these four ingredients because it exhibited minimal hepatotoxicity, the largest rat oral LD_50_ value, and diminished first-pass effect due to its potent inhibition on cytochrome P450 2D6.

### Verification of the anticancer effects of ferulic acid, luteolin, and quercetin

**Figure 6:**
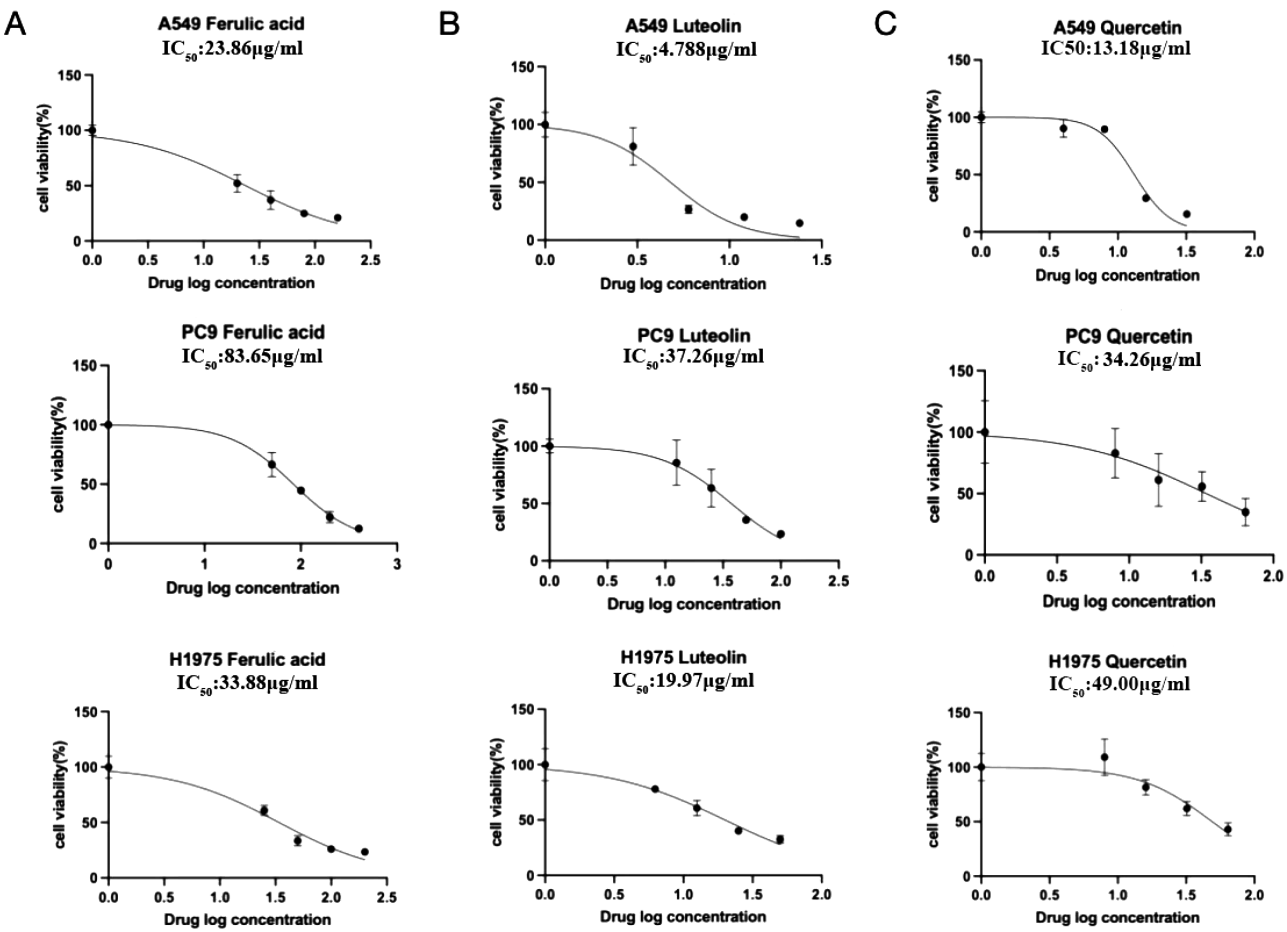
The IC_50_ of indicated drugs in NSCLC cells. The cell proliferation inhibition rate was detected by CCK-8. Ferulic acid **(A)**, luteolin **(B)**, and quercetin **(C)** could significantly inhibit cell proliferation in a concentration-dependent manner.

**Figure 7:**
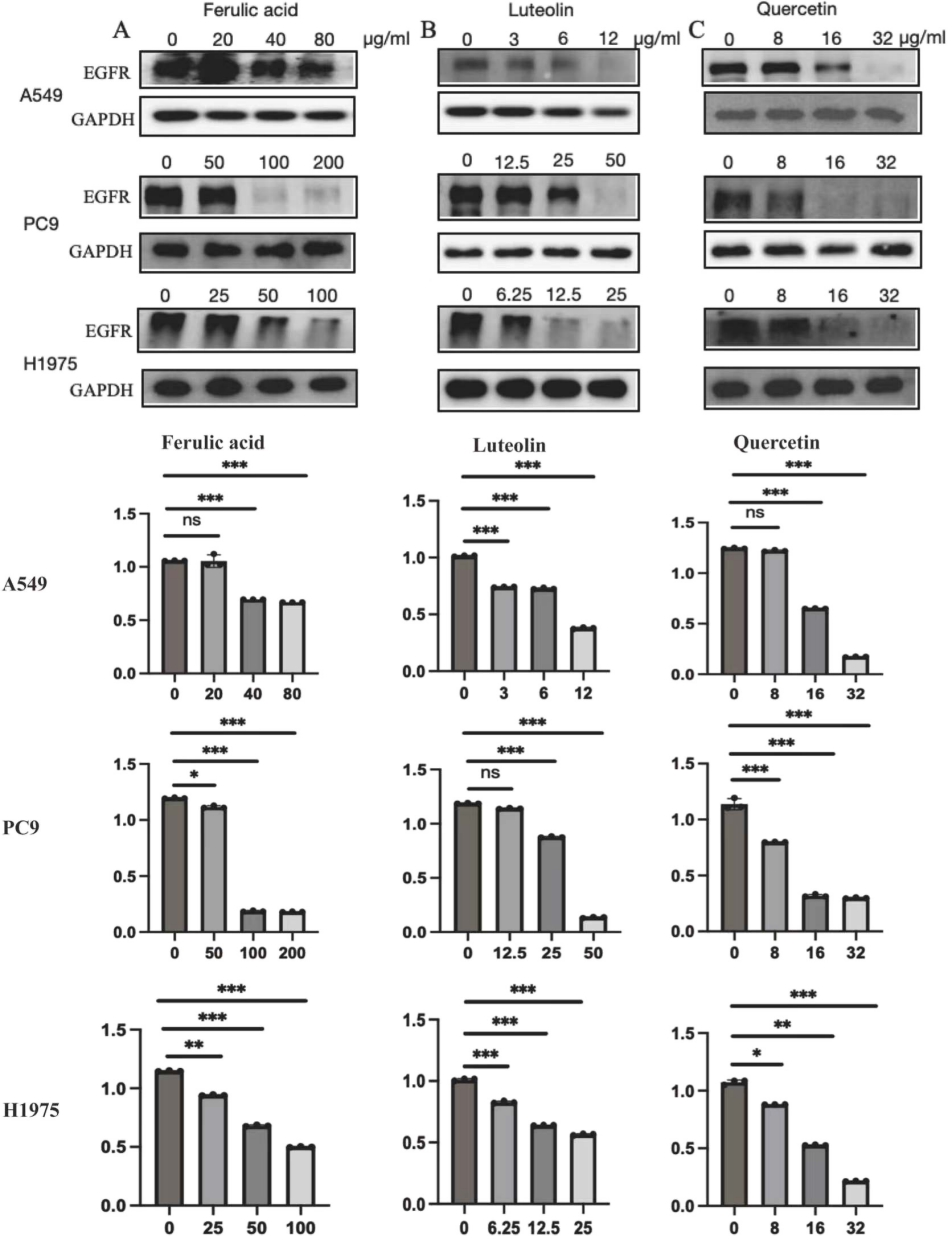
EGFR expression was detected by Western blot and the statistical analysis. Ferulic acid **(A)**, luteolin **(B)**, and quercetin **(C)** could reduce the expression of EGFR in a concentration-dependent manner. ^*^ *P* < 0.05, ^**^ *P* < 0.01, ^***^ *P* < 0.001; ns, not significant.

Since rutin has poor absorption in the body, we chose ferulic acid, luteolin, and quercetin to verify their anticancer activity in NSCLC cells such as A549 (EGFR wild type), PC9 (EGFR mutant without T790M), and H1975 (EGFR mutant with T790M). After the concentration drug treatment was indicated, cell proliferation was detected using CCK-8. The results showed that all of them have good inhibitory effects on NSCLC cells. Notably, ferulic acid (**Figure 6A**), luteolin (**Figure 6B**), and quercetin (**Figure 6C**) showed lower IC_50_ in the EGFR wild-type cell line A549, compared with that of PC9 (EGFR mutant without T790M) and H1975 (EGFR mutant with T790M). However, there was no significant difference between PC9 (EGFR mutant without T790M) and H1975 (EGFR mutant with T790M), suggesting that these drugs may specifically target the EGFR protein. We further detected the expression of the EGFR protein with the indicated drug treatment by Western blot. The results indicated that all of these three active ingredients, namely, ferulic acid (**Figure 7A**), luteolin (**Figure 7B**), and quercetin (**Figure 7C**), had an inhibitory effect on the expression of the EGFR protein in a concentration-dependent manner.

## Discussion

Zhe-Ba-Wei consists of traditional medicinal materials with a long history. In traditional Chinese medicine, Zhe-Ba-Wei plays critical roles in clearing heat and detoxification, reducing swelling and pain, tonifying “Qi” and the blood, regulating secretion, etc. In this study, to systemically study the role and potential application of Zhe-Ba-Wei in cancer, we firstly investigated its active ingredients together with overlapping targets in various types of cancers via network pharmacology and molecular docking. The results showed that 27 overlapping targets were shared by the four active ingredients (ferulic acid, quercetin, rutin, luteolin) that existed in nine types of tumors and were involved diverse biological functions (such as carbonate dehydratase activity, plasma membrane, and hydrolyase activity) and nitrogen metabolism signaling pathway. Meanwhile, based on the combination of KEGG pathway enrichment analysis, molecular docking results, and prediction of ADMET properties, we selected the EGFR protein, which is related to the biological functions of the plasma membrane, as the target. Moreover, we observed the anticancer effects of ferulic acid, luteolin, and quercetin in A549 (EGFR wild type), PC9 (EGFR mutant without T790M), and H1975 (EGFR mutant with T790M) cells. The results showed that all these three drugs had good anticancer effects on A549, PC9, and H1975 cells, and showed lower IC_50_ in the EGFR wild-type cell line A549, compared with that of PC9 (EGFR mutant without T790M) and H1975. Moreover, all of these three drugs had a beneficial suppressive effect on the expression of the EGFR protein.

In summary, the potential anticancer targets of Zhe-Ba-Wei were predicted, and the potential target protein EGFR was experimentally verified. This research suggested that the combination of active components from Zhe-Ba-Wei has the potential to become new therapeutic agents. Besides EGFR, there are other targets identified through network pharmacology screening that may also serve as targets for Zhe-Ba-Wei that still need further research. Meanwhile, this study systematically analyzed the target genes and signaling pathways of ferulic acid, luteolin, quercetin, and rutin in terms of their antitumor effects, providing a basis for further study and potential clinical applications of these four drugs in cancer.

## Data Availability

The original contributions presented in the study are included in the article/**Supplementary Material**. Further inquiries can be directed to the corresponding authors.
