# Supplementary material for: The role and mechanism of “eight famous herbals in Zhejiang” in cancer via network pharmacology and experimental validation
Source: Front Oncol. 2024 Nov 15;14:1475000. doi: 10.3389/fonc.2024.1475000 (PMC11612504; doi:10.3389/fonc.2024.1475000)
Supplement: Supplementary file 1 [file Table1.docx]

| Atractylodes macrocephala lactone 1 | Atractylodes macrocephala lactone 2 | Atractylodes macrocephala lactone 3 | Coumarin | Atractylone |
| --- | --- | --- | --- | --- |
| CYP19A1 | CYP19A1 | RPS6KA5 | CA2 | CHRM5 |
| EPHX1 | ATP12A | PLA2G1B | CA7 | CHRM2 |
| CYP11B2 | AR | ATP12A | CA1 | CHRM3 |
| PGR | PGR | CYP19A1 | CA3 | CHRM4 |
| AR | TTL | CDC25A | CA6 | CHRM1 |
| TBXAS1 | PTPN1 | CDC25B | CA12 | PIK3CA |
| FLT3 | RPS6KA5 | SLC6A3 | CA14 | DRD4 |
| PPARG | TAS2R31 | AR | CA9 | PIK3CG |
| CYP11B1 | PDCD4 | TTL | CA4 | TBXAS1 |
| PRKDC | PLA2G1B | PLA2G2A | CA13 | CYP19A1 |
| KIF11 | PPARG | PTPN1 | CA5B | SLC6A2 |
| CA3 | CDC25C | PGR | CA5A | SLC6A4 |
| CA12 | HMGCR | PARP1 | NFKB1 | SLC6A3 |
| CA14 | RASGRP3 | SLC6A2 | ACHE | CTSD |
| CA9 | CDC25B | SLC6A4 | DAO | CTSS |
| CA13 | PPARA | SIGMAR1 | XDH | CTSL |
| CA5B | PPARD | DCTPP1 | EGFR | CTSB |
| CA5A | CYP11B1 | TAS2R31 | CYP11B1 | MGLL |
| CTSK | CYP11B2 | CYP51A1 | NUDT1 | SIGMAR1 |
| ADRA2C | FLT3 | PTK2B | NQO1 | HSD11B1 |
| TBXA2R | FNTA | HCRTR2 | MAPK14 | P2RX7 |
| CEL | TBXAS1 | HCRTR1 | TYMS | PRKDC |
| KCNK2 | EPHX1 | MAP2K1 | ESR1 | S1PR3 |
| TTL | KCNK2 | MAPK1 | ESR2 | FKBP1A |
| CHRM4 | CDC25A | P2RX7 | NOS2 | SLC5A7 |
| CHRM5 | CHRM4 | FADS1 | LIMK1 | TRPA1 |
| CHRM2 | CHRM5 | ADRA2C | TAAR1 | RAPGEF4 |
| CHRM1 | CHRM2 | PDGFRB | HSD17B3 | NOS2 |
| CHRM3 | CHRM1 | PSEN2 | CYP11B2 | PABPC1 |
| XPO1 | CHRM3 | IMPDH2 | BCHE | PDE4D |
| TYMS | PRKCA | HMOX1 | CES1 | IMPDH2 |
| PARP1 | TYMS | MDM2 | TGM2 | S1PR1 |
| LRRK2 | PRKDC | CHRM4 | CES2 | AKR1C3 |
| PTPRC | ADH1A | CHRM2 | GABRB3 | EPHX2 |
| PTPRF | SRD5A1 | PPARG | CTSA | AR |
| KCNJ5 | KIF11 | CDC25C | BRD9 | RXRA |
| MPO | MAOA | TTR | MAOA | HTR2A |
| ELANE | CA3 | PDCD4 | CTRC | HTR2C |
| MAOB | PARP1 | GSK3A | METAP2 | APP |
| EPHX2 | CYP17A1 | ALK | GPR35 | MTNR1A |
| PABPC1 | HSD11B1 | RASGRP3 | ERBB2 | MTNR1B |
| MAPK14 | CTSK | MAPK14 | AKR1B1 | DRD2 |
| ADH1A | MPO | TRPV3 | CCND1 | PTPN1 |
| DRD4 | HSD11B2 | ADRA2A | PDGFRB | PIK3R1 |
| PGGT1B | DRD4 | BCHE | FLT4 |  |
| HSD17B3 | IL1B | ADRA2B | IGF1R |  |
| PIK3CD | XPO1 | CDK2 | INSR |  |
| ACHE | LRRK2 | EPHX1 | CDK2 |  |
| KDR | ADRA2C | MET | AURKB |  |
| JAK1 | PCSK7 | IMPDH1 | SRC |  |
| JAK2 | F2RL1 | KCNK3 | PTK2 |  |
| AURKA | PPP2CA | KCNK9 | KDR |  |
| STAT3 | AHR | ATP1A1 | PLK1 |  |
| RASGRP3 | CHRNA4 | LRRK2 | CSNK2A1 |  |
| F2 | MAPK14 | KCNH2 | PLK4 |  |
| PRSS1 | ADH1C | IDO1 | TEK |  |
| MTNR1A | NR3C2 | MTNR1A | AKT1 |  |
| MTNR1B | MAOB | MTNR1B | AURKA |  |
| CTRB1 | IARS | DHODH | BACE1 |  |
| SRD5A1 | PDE7A | MAPK8 | MAP3K8 |  |
| CYP2A6 | KAT2B | CCNE2 | BRAF |  |
| TNKS2 | MTNR1A | CCNB3 | EPHB4 |  |
| TNKS | MTNR1B | CCNE1 | HSPA1A |  |
| GRM5 | CEL | DBF4 | NUAK1 |  |
| JAK3 | SLC6A2 | CDC7 | SQLE |  |
| CTSL | PTPRC | SCN5A | FGR |  |
| CTSB | PTPRF | ROCK2 | LYN |  |
| KAT2B | HSD17B3 | SCN9A | HTT |  |
| AHR | PLA2G2A | BACE2 | ADRA2B |  |
| HTR2B | PABPC1 | ADORA2A | GABRA2 |  |
| FYN | SIGMAR1 | ADORA2B | PTGS1 |  |
| ADRA2A | GABRB3 | ADORA3 | CTSK |  |
| ADRA2B | MMP13 | CSNK1D | CHRNA3 |  |
| NR1H4 | MMP1 | HTR2C | CYP17A1 |  |
| GABRB3 | KCNJ5 | PDE10A | GSR |  |
| PTGS1 | CTSL | RIPK2 | F13A1 |  |
| HTR2A | CTSB | CCR3 | CTRB1 |  |
| HTR2C | IMPDH2 | PPARA | NOS1 |  |
| SLC6A4 | CA2 | PPARD | CYP1B1 |  |
| DRD3 | TNKS2 | RPS6KA3 | MCL1 |  |
| ADORA3 | TNKS | NQO2 | ADORA1 |  |
| CYP2D6 | PIK3CD | CDK5 | TBXAS1 |  |
| ADH1C | SIRT2 | FLT1 | GPR55 |  |
| CYP1A2 | P2RX7 | PIM1 | DRD4 |  |
| CYP2C9 | CYP2A6 | AURKB | KDM5A |  |
| CYP3A4 | JAK3 | MAPKAPK2 | KDM5B |  |
| IDO1 | JAK1 | C5AR1 | FYN |  |
| GPR55 | JAK2 | CSNK1G1 | ADORA2B |  |
| CTRC | GSK3B | RPS6KA1 | CDC7 |  |
| HMOX1 | PTPN11 | LCK | ADH1A |  |
| P2RX7 | NQO2 | SYK | IDO1 |  |
| TPO | MGLL | KDR | SRD5A1 |  |
| CHRNA4 | CTRB1 | ROCK1 | ADH1B |  |
| CTSH | GCGR | LYN | HSP90AA1 |  |
| MST1R | PTGS1 | KCNA5 | MGLL |  |
| PTPN1 | HMOX1 | TXK | PTGS2 |  |
| BRD9 | BRD9 | RPS6KB1 | EPHX1 |  |
| NR3C1 | HTR7 | PIM2 | CHRM2 |  |
| PRKCA | TPO | BTK | CCNA1 |  |
| IMPDH2 | IKBKB | CCNA1 | CDK4 |  |
| CHRNB2 | CHRNB2 | CDK1 | CHRNB4 |  |
| FNTA | FNTB | PSENEN | GABRA3 |  |
| GABRG2 | GABRG2 | CCNA2 | GABRG2 |  |
| KCNJ3 | KCNJ3 | CCNB1 | CCNA2 |  |
| PIK3R1 | PIK3R1 | NCSTN | GABRA1 |  |
| GABRA5 | GABRA5 | APH1A | GABRA5 |  |
|  |  | CCNB2 |  |  |
|  |  | PSEN1 |  |  |
|  |  | APH1B |  |  |

| Benzoyl-paeoniflorin | Chlorogenic acid | Luteolin | Quercetin | Rutin |
| --- | --- | --- | --- | --- |
| LGALS3 | AKR1B1 | NOX4 | NOX4 | NMUR2 |
| LGALS9 | AKR1B10 | AKR1B1 | AVPR2 | ADRA2A |
| HSP90AA1 | MMP13 | CDK5R1 | AKR1B1 | ADRA2C |
| SLC6A2 | MMP2 | XDH | XDH | ACHE |
| SSTR2 | APP | MAOA | MAOA | AKR1B1 |
| SSTR1 | MMP12 | FLT3 | IGF1R | CA7 |
| SSTR3 | SLC37A4 | CA2 | FLT3 | CA12 |
| VEGFA | CA2 | CCNB3 | CYP19A1 | CA4 |
| FGF1 | CA1 | ALOX5 | EGFR | NOX4 |
| FGF2 | CA12 | ADORA1 | F2 | CA2 |
| HPSE | CA9 | CA7 | CA2 | NQO2 |
| ADORA2A | PYGL | GLO1 | PIM1 | RPS6KA3 |
| ADORA3 | PRKCD | APP | ALOX5 | XDH |
| ADORA2B | PRKCA | SYK | AURKB | CD38 |
| AMY1A | NEU4 | GSK3B | DRD4 | PTGS2 |
| SERPINE1 | BACE1 | PARP1 | ADORA1 | PDE5A |
| SQLE | CASP3 | TTR | CA7 | TNF |
| ABCB1 | PDE4D | MMP9 | GLO1 | IL2 |
| SSTR5 | PDE9A | CA12 | MPO | ADORA1 |
| TOP1 | PDE1B | MMP2 | PIK3R1 | ALOX5 |
| MMP9 | KDR | CA4 | ADORA2A | TERT |
| SLC29A1 | ENGASE | MMP12 | DAPK1 | VCP |
| IMPDH1 | CA5B | CD38 | PYGL | TNNC1 |
| IMPDH2 | ABCB1 | CYP1B1 | CA1 | SQLE |
| HRAS | NEU3 | ABCG2 | GSK3B | ADORA3 |
| PTAFR | NEU2 | AKR1B10 | SRC | SLC29A1 |
| MMP13 | ELANE | TNKS2 | PTK2 | PLG |
| MMP1 | OGA | TNKS | HSD17B2 | ABCG2 |
| MMP7 | TREH | TOP1 | KDR | TP53 |
| MMP8 | CASP6 | ARG1 | MMP13 | CYP1B1 |
| PTPN1 | CASP7 | PTPRS | MMP3 | KCNA3 |
| MMP3 | CASP8 | ABCC1 | CA3 | TDP1 |
| ADAM17 | CASP1 | HSD17B1 | ALOX15 | PRKCD |
| YARS | CASP2 | ACHE | ABCC1 | PRKCA |
| CA14 | ECE1 | CDK6 | PLK1 | PRKCB |
| MAP2K1 | EGLN1 | ABCB1 | CA6 | PRKCE |
| SLC28A3 | ADAMTS5 | HSD17B2 | CDK1 | PRKCH |
| MAP3K7 | FTO | CYP19A1 | MMP9 | SERPINE1 |
| BCHE | YARS | ESR2 | CA12 | SRC |
| OGA | DNMT3B | ADORA2A | MMP2 | PRKCG |
| CCNA2 | SELL | CSNK2A1 | PKN1 | PRKACA |
| MAPK1 | SELP | ALOX15 | CA14 | PRKCZ |
| ST6GAL1 | NAALAD2 | ALOX12 | CA9 | ESR1 |
| F10 | SLC13A5 | ESR1 | CSNK2A1 | APP |
| GPR55 | HPRT1 | PTGS2 | ALOX12 | F10 |
| SSTR4 | ADORA3 | CFTR | MET | MCL1 |
| MAP2 | MGAM | AMY1A | CA4 | CHEK2 |
| SLC28A2 | MAPK8 | GRK6 | NEK2 | CHEK1 |
| MME | SI | CA1 | CXCR1 | ALDH2 |
| CASP3 | PTPN22 | CA9 | CAMK2B | KISS1R |
| CASP6 | FOLH1 | CDK2 | ALK | HSP90AB1 |
| CASP8 | AHCY | TERT | AKT1 | ADRB1 |
| ECE1 | MGLL | CDK1 | ABCB1 | CA1 |
| CASP1 | ITGAL | TYR | NEK6 | CA9 |
| BACE1 | ACLY | AHR | PLA2G1B | CA13 |
| PDE4D | CFTR | ESRRA | CA5A | EGFR |
| PARP1 | MME | GPR35 | BACE1 | ADORA2A |
| TNKS2 | GSR | AVPR2 | CYP1B1 | MAPT |
| TNKS | PIK3CG | IGF1R | AXL | KDM4E |
| IRAK4 | AMPD3 | EGFR | ABCG2 | GPR35 |
| PRKCA | PTGS1 | F2 | NUAK1 | AVPR2 |
| HCAR2 | MAPK1 | PIM1 | AKR1C2 | TOP2A |
| UPP1 | AKR1C4 | AURKB | AKR1C1 | MAOA |
| EIF4A1 | ADA | DRD4 | AKR1C3 | IGF1R |
| HSPA8 | MMP16 | MPO | AKR1C4 | FLT3 |
| HSPA5 | GRIK2 | PIK3R1 | CA13 | CYP19A1 |
| GAPDH | GRIK3 | DAPK1 | AKR1A1 | INSR |
| LTA4H | MMP14 | PYGL | GPR35 | F2 |
| MARS | MMP8 | SRC | MAPT | PIM1 |
| ABL1 | CA14 | PTK2 | KDM4E | AURKB |
| EPHA2 | ENPEP | KDR | TOP2A | DRD4 |
| LCK | ALOX5 | MMP13 | INSR | GLO1 |
| SRC | IGF1R | MMP3 | ACHE | MYLK |
| KDR | INSR | CA3 | MYLK | MPO |
| MAP3K9 | GRK6 | PLK1 | SYK | PIK3R1 |
| SLC37A4 | IKBKB | CA6 | PIK3CG | DAPK1 |
| ADK | SRC | PKN1 | APEX1 | PYGL |
| FGFR1 | POLB | CA14 | PTPRS | SYK |
| AURKA | PIN1 | MET | ESR2 | GSK3B |
| BTK | HK2 | NEK2 | MPG | PTK2 |
| P2RX3 | HK1 | CXCR1 | SLC22A12 | HSD17B2 |
| EIF4H | ACE | CAMK2B | CDK5R1 CDK5 | KDR |
| PABPC1 | CDK2 | ALK | CCNB3 CDK1 CCNB1 CCNB2 | MMP13 |
| HK2 | IGFBP3 | AKT1 | ARG1 | MMP3 |
| HK1 | GNPAT | NEK6 | CDK6 | CA3 |
| AKR1B1 | ACP1 | PLA2G1B | CDK2 | ALOX15 |
| EGFR | KDM4D | CA5A | TYR | PLK1 |
| AKR1C3 | KDM4C | BACE1 | HSD17B1 | CA6 |
| SLC5A2 | MMP1 | AXL | AHR | CDK1 |
| GBA | FPGS | NUAK1 | ESRRA | MMP9 |
| PDE7A | PYGM | AKR1C2 | APP | PIK3CG |
| TYMP | AMY2A | AKR1C1 | PARP1 | MMP2 |
| CHEK1 | MMP9 | AKR1C3 | TTR | PKN1 |
| ACE | GPR35 | AKR1C4 | MMP12 | CA14 |
| METAP2 | KMT2A | CA13 | CD38 | CSNK2A1 |
| VHL | DOT1L | AKR1A1 | AKR1B10 | ALOX12 |
| TARS | SUV39H1 | PFKFB3 | TNKS2 | MET |
| STS | DNMT1 | PLG | TNKS | NEK2 |
| F7 | INMT | KDM4E | TOP1 | CXCR1 |
| AGTR1 | SMYD2 | AR | TERT | CAMK2B |
| CDK2 | ICAM1 | CDK5 |  | TNNT2 |
|  | ITGB2 | CCNB1 |  | TNNI3 |
|  |  | CCNB2 |  |  |

| Ferulic Acid | Tetrahy-droberberine | Tetrahy-droberberine | Berberine | Epiberberine |
| --- | --- | --- | --- | --- |
| CA2 | DRD1 | DRD1 | ACHE | SAE1 |
| CA7 | DRD2 | HTR1A | HTR2B | ACHE |
| CA1 | F3 | DRD2 | BCHE | BCHE |
| CA6 | HTR7 | F3 | ADRA2C | SIGMAR1 |
| CA12 | CHRM4 | HTR7 | ADRA2B | HTR2B |
| CA14 | HTR1A | CHRM4 | CHRM1 | ADRA2C |
| CA9 | ADRA1D | SLC6A3 | SIGMAR1 | ADRA2B |
| CA5A | DRD4 | ADRA1A | CYP2D6 | CHRM1 |
| CA5B | SIGMAR1 | ADRA1D | SAE1 | CYP2D6 |
| MAOB | DRD3 | ADRA1B | RAC1 | RAC1 |
| AKR1B1 | ADRA1A | DRD3 | CDC42 | RPS6KB1 |
| ALOX5 | ADRA1B | DRD5 | RPS6KB1 | AURKA |
| MMP9 | SLC6A3 | DRD4 | AURKA | CDC42 |
| MMP1 | DRD5 | SIGMAR1 | AURKB | HPGD |
| MMP2 | HTR2A | HTR2A | CYP11B2 | TBXAS1 |
| PTPN1 | HCRTR1 | ADRB1 | PRF1 | MAOB |
| CA13 | ADRB2 | ADRB3 | GRIA1 | PIK3CG |
| CA3 | ADRB3 | MAOA | TBXAS1 | GRK5 |
| APP | HTR2C | HCRTR1 | HPGD | CYP19A1 |
| NFE2L2 | ADRB1 | CDK2 | SLC1A3 | SCD |
| STAT3 | ABCB1 | TTK | GABRB3 | AURKB |
| HSD11B1 | TBXA2R | CHEK1 | PIM1 | XBP1 |
| ESR2 | ACHE | HTR2B | PIM2 | NTRK1 |
| CA4 | MAOA | HTR2C | MET | PIK3CD |
| TLR4 | HTR2B | CLK4 | HTR3A | PIK3CB |
| PTGS1 | MTNR1B | DHCR7 | GRK5 | GRIA1 |
| MET | BCHE | OPRM1 | CYP11B1 | MAPKAPK2 |
| CYP1A1 | DHCR7 | MTNR1B | IMPDH2 | JAK2 |
| CYP1A2 | ADRA2C | FLT3 | PIK3CA | IMPDH2 |
| NQO2 | KCNN3 | STS | F3 | CHEK2 |
| CYP1B1 | CDC7 | MAPK8 | CYP19A1 | TGM2 |
| CPA1 | CHRNB4 | QTRT1 | BCAT2 | CD38 |
| EGFR | CHRNA3 | CHRNB4 | TRPM8 | LCK |
| PTGS2 | PIK3CD | CHRNA3 | ICAM1 | PIM1 |
| TTR | PIK3CB | CTSL | SELE | PIM2 |
| KDM4E | PIK3CA | ADRA2A | MAOB | MAPK10 |
| KDM3A | GRIA1 | ADRA2C | PIK3CD | MAP4K4 |
| KDM6B | CHRNA4 | PIK3CD | PIK3CB | PARP1 |
| FTO | HCRTR2 | CDK5R1 | PIK3CG | F3 |
| KDM4A | KCNN1 | PIM1 | MAPKAPK2 | TRPM8 |
| KDM4C | KCNN2 | FAAH | PARP10 | ABL1 |
| TUBB1 | CDK2 | DYRK1A | CHEK2 | SIRT3 |
| RELA | MAOB | PTGS2 | CCNC | SIRT2 |
| FYN | PPP1CA | PIK3CG | CDK8 | SIRT1 |
| LCK | CDK4 | DCTPP1 | MAPK10 | NR3C2 |
| SLC16A1 | CTSL | CLK1 | AGPAT2 | DHFR |
| TLR9 | MTNR1A | CLK3 | TGM2 | FLT1 |
| AKR1B10 | CYP19A1 | DYRK2 | SCD | ALOX5AP |
| ALOX15 | ADRA2B | KCNH2 | XBP1 | ROCK1 |
| PRKCE | CYP11B1 | ABCB1 | TYMS | BCAT2 |
| F3 | CYP11B2 | RBBP9 | DHFR | PRKACA |
| NOS2 | SLC1A3 | ACHE | GABRA2 | CYP11B2 |
| NGFR | BTK | CHRNA4 | ROCK1 | ICAM1 |
| CCND1 | SCN9A | CCND1 | PRKACA | SELE |
| TUBB3 | MKNK1 | ABL1 | CHRM4 | AGPAT2 |
| ABCB1 | ADRA2A | CHEK2 | JAK2 | DRD4 |
| FBP1 | CDK5R1 | TBXA2R | LCK | DRD3 |
| TOP2A | CCNB3 | SLC6A4 | CDK9 | KIT |
| GLO1 | PTGS2 | MTNR1A | PTPN1 | SRC |
| BACE1 | TGFBR1 | TYMS | ALOX5AP | MKNK1 |
| ACE | MAPKAPK2 | CDC7 | CHEK1 | FLT3 |
| REN | SYK | XBP1 | KIT | IKBKB |
| PARP1 | CDK9 | MAP2 | SRC | ADORA2A |
| MAOA | HTR6 | HTR1B | MKNK1 | ADORA3 |
| AHR | ADORA1 | HRH2 | IKBKB | PTPN1 |
| KDM2A | GCK | ADRA2B | MAPK14 | ZAP70 |
| TPMT | KCNH2 | HTR1D | LRRK2 | SNCA |
| CTNNB1 | ESR1 | HTR6 | ROCK2 | JAK3 |
| F2 | OPRM1 | HTR5A | ATR | GSK3A |
| SLC13A5 | PARP1 | GRK3 | AOC3 | IRAK4 |
| IKBKG | MAPK8 | CA12 | GRK3 | MAPK1 |
| ECE1 | ABCC1 | GRK2 | PLK1 | SLC22A12 |
| KMO | PDE10A | KIF11 | GRK2 | HTR3A |
| ITGAL | PARP2 | SAE1 | NPY5R | AKR1B1 |
| AMPD3 | SLC18A2 | KDR | MAP4K4 | TYMS |
| PTGDR2 | NR3C2 | RET | NTRK1 | CHEK1 |
| MME | QTRT1 | CCNB3 | ABL1 | ERBB2 |
| MAPK8 | ABL1 | BACE2 | CDK1 | MAPK8 |
| SLC6A2 | CTSK | CDK1 | NR3C2 | SCN9A |
| AKR1C4 | HTR1B | CDK4 | PGR | NPY5R |
| ACLY | TBXAS1 | CDK5 | DRD4 | SLC5A1 |
| DTYMK | TYMS | SIRT1 | PTGS2 | PDE4B |
| OGA | NTRK1 | QPCT | DRD3 | CCNE1 |
| AKR1C3 | GRM5 | BTK | CDK2 | CYP11B1 |
| MIF | GRM1 | EIF2AK2 | CDK4 | EPHA2 |
| DAO | RPS6KB1 | MAOB | SIRT2 | FAAH |
| DYRK1B | AURKA | BRD4 | ADORA2A | MST1R |
| PTPN2 | NEK1 | BRD2 | ADORA3 | DPP4 |
| SLC6A3 | GSTP1 | BRD3 | GRM5 | ROCK2 |
| PYGL | GSTM2 | AURKB | QPCT | CCNC |
| PYGM | GRK3 | MAPKAPK2 | CSF1R | MET |
| ADA | TDP2 | CSNK1G1 | CNR2 | STAT3 |
| GSK3B | AURKB | RPS6KA1 | CBFB | AXL |
| EDNRA | MET | PLK1 | HSD17B1 | CDK8 |
| ALPL | GRK2 | PRKCB | PNMT | MME |
| MAPT | GRK5 | CDK9 | PARP2 | RPS6KA3 |
| NOX4 | CSF1R | PRKDC | PTGES | EPHX2 |
| CAMKK2 | CCNA1 | PIK3CA | RPS27 | QPCT |
| ABCG2 | CDK1 | MAPK1 | CCNB1 | MARS |
| IGFBP3 | CDK5 | CCNA1 | GABRA3 | MMP9 |
| CDK4 | CHRNA2 | CHRNA2 | GABRG2 | CDK2 |
| ICAM1 | CHRNB2 | CHRNB2 | PIK3R1 | UBA2 |
| IKBKB | PIK3R1 | UBA2 | UBA2 |  |
| CHUK | CCNA2 | CCNA2 | GABRA1 |  |
| ITGB2 | CCNB1 | CCNB1 | GABRA5 |  |
|  | CCNB2 | CCNB2 |  |  |

| Coptisine | β-elemene |
| --- | --- |
| ACHE | CXCR3 |
| SIGMAR1 | HTR2A |
| CHRM1 | MAOB |
| HTR2B | CYP1A2 |
| BCHE | MGLL |
| ADRA2C | HSD11B1 |
| ADRA2B | BCHE |
| CYP2D6 | MAOA |
| SAE1 | ACHE |
| RAC1 | SLC6A2 |
| CDC42 | HTR2C |
| CHRM4 | PTGS2 |
| TBXAS1 | UGT2B7 |
| XBP1 | ESR2 |
| IKBKB | FADS1 |
| PLK1 | PPARA |
| PRF1 | CNR2 |
| ABL1 | SHBG |
| CHEK2 | NR1H3 |
| SCN9A | CYP19A1 |
| CDC7 | PTPN1 |
| LIMK1 | NR1I3 |
| SCD | PTGS1 |
| DHFR |  |
| PIK3CD |  |
| PIK3CB |  |
| PIK3CG |  |
| CA2 |  |
| PARP1 |  |
| CSF1R |  |
| HPGD |  |
| RPS6KB1 |  |
| AURKA |  |
| TUBB1 |  |
| ADK |  |
| KIT |  |
| CYP19A1 |  |
| MAP3K8 |  |
| CCNC |  |
| PTGES |  |
| CDK8 |  |
| ADRA1D |  |
| FLT3 |  |
| MAOB |  |
| RPS27 |  |
| MAPK8 |  |
| SNCA |  |
| CYP11B1 |  |
| CYP11B2 |  |
| CHEK1 |  |
| BRAF |  |
| MME |  |
| LRRK2 |  |
| IMPDH2 |  |
| CYP17A1 |  |
| MCL1 |  |
| ALOX5AP |  |
| CDK2 |  |
| AURKB |  |
| PTGS1 |  |
| PTGS2 |  |
| PDE4B |  |
| ERBB2 |  |
| ATP4B |  |
| PDE4A |  |
| ADORA2B |  |
| MAPKAPK2 |  |
| PYGL |  |
| HSD11B1 |  |
| CHRM5 |  |
| ENPP1 |  |
| PDGFRB |  |
| FLT4 |  |
| SRC |  |
| TNNI3K |  |
| RPS6KA3 |  |
| MYLK |  |
| DCK |  |
| TUBB3 |  |
| PLK4 |  |
| ABCB1 |  |
| AKR1B1 |  |
| PIK3CA |  |
| ADORA3 |  |
| AKT2 |  |
| PDPK1 |  |
| PRKCA |  |
| MAP2K1 |  |
| JAK3 |  |
| LCK |  |
| TGM2 |  |
| NTRK1 |  |
| GSK3A |  |
| ITK |  |
| STS |  |
| MET |  |
| IRAK4 |  |
| MAPK1 |  |
| HSD17B3 |  |
| PHLPP2 |  |
| UBA2 |  |
| CCNA1 |  |
| CCNA2 |  |
| PIK3R1 |  |
| ATP4A |  |
