# Supplementary material for: The role and mechanism of “eight famous herbals in Zhejiang” in cancer via network pharmacology and experimental validation
Source: Front Oncol. 2024 Nov 15;14:1475000. doi: 10.3389/fonc.2024.1475000 (PMC11612504; doi:10.3389/fonc.2024.1475000)
Supplement: Supplementary file 2 [file Table2.docx]

| **Lung cancer** | **Cervical cancer** | **Colorectal cancer** | **Bladder cancer** |
| --- | --- | --- | --- |
| ABCA3 | ACVR1B | ACVR1B | ACVR1B |
| ACVR1B | ADH1B | ADH1B | ADH1B |
| ADH1B | AKT1 | AKT1 | AKT1 |
| AKT1 | ALDH2 | ALDH2 | ALDH2 |
| ALDH2 | APC | APC | APC |
| APC | AR | AR | AR |
| ATM | ATM | ATM | ATM |
| ATP13A3 | ATR | ATR | ATR |
| ATR | AURKA | AURKA | AURKA |
| BARD1 | AXIN2 | AXIN2 | AXIN2 |
| BCAR3 | BARD1 | BARD1 | BARD1 |
| BCAR4 | BAX | BAX | BAX |
| BCPR | BCAR3 | BCAR3 | BCAR3 |
| BLACAT1 | BCAR4 | BCAR4 | BCAR4 |
| BMPR2 | BCPR | BCPR | BCPR |
| BRAF | BLACAT1 | BLACAT1 | BLACAT1 |
| BRCA1 | BLCAP | BLCAP | BLCAP |
| BRCA2 | BRAF | BRAF | BRAF |
| BRIP1 | BRCA1 | BRCA1 | BRCA1 |
| BUB1 | BRCA2 | BRCA2 | BRCA2 |
| BUB1B | BRIP1 | BRIP1 | BRIP1 |
| CASC1 | BUB1 | BUB1 | BUB1 |
| CASC11 | BUB1B | BUB1B | BUB1B |
| CASC15 | CASC1 | CAHM | CASC1 |
| CASC19 | CASC11 | CASC1 | CASC11 |
| CASC2 | CASC15 | CASC11 | CASC15 |
| CASC21 | CASC19 | CASC15 | CASC19 |
| CASC8 | CASC2 | CASC19 | CASC2 |
| CASP10 | CASC21 | CASC2 | CASC21 |
| CASP8 | CASC8 | CASC21 | CASC8 |
| CAV1 | CASP10 | CASC8 | CASP10 |
| CCAT1 | CASP8 | CASP10 | CASP8 |
| CCAT2 | CCAT1 | CASP8 | CCAT1 |
| CCND1 | CCAT2 | CCAT1 | CCAT2 |
| CDH1 | CCND1 | CCAT2 | CCND1 |
| CDK2AP1 | CDH1 | CCND1 | CDH1 |
| CDKN2A | CDK2AP1 | CDH1 | CDK2AP1 |
| CHRNA3 | CDKN2A | CDK2AP1 | CDKN2A |
| CHRNA5 | CHEK2 | CDKN2A | CHEK2 |
| COLCA1 | CHRNA3 | CHEK2 | CHRNA3 |
| COLCA2 | CHRNA5 | CHRNA3 | CHRNA5 |
| COPA | COLCA1 | CHRNA5 | COLCA1 |
| COPD | COLCA2 | COLCA1 | COLCA2 |
| CPS1 | CRCS11 | COLCA2 | CRCS11 |
| CRCS2 | CRCS2 | CRCS11 | CRCS2 |
| CRCS5 | CRCS5 | CRCS2 | CRCS5 |
| CRCS6 | CRCS6 | CRCS5 | CRCS6 |
| CRCS7 | CRCS7 | CRCS6 | CRCS7 |
| CRCS8 | CRCS8 | CRCS7 | CRCS8 |
| CRCS9 | CRCS9 | CRCS8 | CRCS9 |
| CTAG3 | CT47A10 | CRCS9 | CT47A10 |
| CTEPH1 | CT47A11 | CRNDE | CT47A11 |
| CTNNB1 | CT47A5 | CT47A10 | CT47A4 |
| CTTN | CT47A6 | CT47A11 | CT47A5 |
| DIRC1 | CT47A7 | CT47A6 | CT47A6 |
| DLC1 | CT47A8 | CT47A7 | CT47A7 |
| DLEC1 | CT47A9 | CT47A8 | CT47A8 |
| EGFR | CT47B1 | CT47A9 | CT47A9 |
| EHBP1 | CT83 | CT47B1 | CT47B1 |
| EIF2AK4 | CTAG3 | CT83 | CT83 |
| EIF4G1 | CTNNB1 | CTAG3 | CTAG3 |
| ELAC2 | CTTN | CTNNB1 | CTNNB1 |
| EPCAM | CYP2A6 | CTTN | CTTN |
| EPHB2 | DCC | CYP2A6 | CYP2A6 |
| ERBB2 | DIRC1 | DCC | DCC |
| ERCC6 | DLC1 | DIRC1 | DIRC1 |
| ESR1 | DLEC1 | DLC1 | DLC1 |
| FALEC | EGFR | DLEC1 | DLEC1 |
| FAM111B | EHBP1 | EGFR | EGFR |
| FARSB | EIF4G1 | EHBP1 | EHBP1 |
| FASLG | ELAC2 | EIF4G1 | EIF4G1 |
| FGFR2 | EP300 | ELAC2 | ELAC2 |
| FGFR3 | EPCAM | EP300 | EP300 |
| FGFR4 | EPHB2 | EPCAM | EPCAM |
| FH | ERBB2 | EPHB2 | EPHB2 |
| FLCN | ERCC6 | ERBB2 | ERBB2 |
| FMO2 | ESR1 | ERCC6 | ERCC6 |
| FOXE1 | FALEC | ESR1 | ESR1 |
| FOXF1 | FASLG | FALEC | FALEC |
| GACAT3 | FGFR2 | FASLG | FASLG |
| GAEC1 | FGFR3 | FGFR2 | FGFR2 |
| GALNT12 | FGFR4 | FGFR3 | FGFR3 |
| GCRG224 | FH | FGFR4 | FGFR4 |
| HABP2 | FLCN | FH | FH |
| HEPN1 | FOXE1 | FLCN | FLCN |
| HIC1 | GACAT2 | FOXE1 | FOXE1 |
| HMMR | GACAT3 | GACAT2 | GACAT2 |
| HMPS1 | GAEC1 | GACAT3 | GACAT3 |
| HOXB13 | GALNT12 | GAEC1 | GAEC1 |
| HPC10 | GCRG224 | GALNT12 | GALNT12 |
| HPC11 | HABP2 | GCRG224 | GCRG224 |
| HPC14 | HEPN1 | HABP2 | HABP2 |
| HPC4 | HIC1 | HEPN1 | HEPN1 |
| HPC5 | HIC2 | HIC1 | HIC1 |
| HPC7 | HMMR | HIC2 | HIC2 |
| HPC9 | HMPS1 | HMMR | HMMR |
| HRAS | HOXB13 | HMPS1 | HMPS1 |
| IGFBP7 | HPC10 | HOXB13 | HOXB13 |
| IL1B | HPC11 | HPC10 | HPC10 |
| IL1RN | HPC14 | HPC11 | HPC11 |
| IQGAP1 | HPC15 | HPC14 | HPC14 |
| IRF1 | HPC3 | HPC15 | HPC15 |
| ITGA3 | HPC4 | HPC3 | HPC3 |
| KCNK3 | HPC5 | HPC4 | HPC4 |
| KLF6 | HPC6 | HPC5 | HPC5 |
| KMHN1 | HPC7 | HPC6 | HPC6 |
| KRAS | HPC9 | HPC7 | HPC7 |
| LCO | HPCQTL19 | HPC9 | HPC9 |
| LNCR1 | HPCX2 | HPCQTL19 | HPCQTL19 |
| LNCR3 | HRAS | HPCX2 | HPCX2 |
| LNCR4 | IL1B | HRAS | HRAS |
| LNCR5 | IL1RN | IL1B | IL1B |
| LUCAT1 | IQGAP1 | IL1RN | IL1RN |
| MAD1L1 | IRF1 | IQGAP1 | IQGAP1 |
| MADH9 | KLF6 | IRF1 | IRF1 |
| MALAT1 | KMHN1 | KLF6 | KLF6 |
| MAP3K8 | KRAS | KMHN1 | KMHN1 |
| MARS1 | LCO | KRAS | KRAS |
| MCC | LNCR1 | LCO | LCO |
| MEN1 | LNCR3 | LNCR1 | LNCR1 |
| MLH1 | LNCR4 | LNCR3 | LNCR3 |
| MLH3 | LNCR5 | LNCR4 | LNCR4 |
| MMP1 | LUCAT1 | LNCR5 | LNCR5 |
| MPO | MAD1L1 | LUCAT1 | LUCAT1 |
| MSH2 | MAP3K8 | MAD1L1 | MAD1L1 |
| MSH6 | MCC | MAP3K8 | MAP3K8 |
| MSMB | MLH1 | MCC | MCC |
| MUC5B | MLH3 | MLH1 | MLH1 |
| MUTYH | MPO | MLH3 | MLH3 |
| MXI1 | MSH2 | MPO | MPO |
| NKX2-1 | MSH6 | MSH2 | MSH2 |
| NQO1 | MSMB | MSH6 | MSH6 |
| NQO2 | MUTYH | MSMB | MSMB |
| NRAS | MXI1 | MUTYH | MUTYH |
| NSMCE3 | NCOA3 | MXI1 | MXI1 |
| OAS1 | NKX2-1 | NCOA3 | NCOA3 |
| OPCML | NQO1 | NKX2-1 | NKX2-1 |
| ORAOV1 | NQO2 | NQO1 | NQO1 |
| OVCAS1 | NRAS | NQO2 | NQO2 |
| PALB2 | OPCML | NRAS | NRAS |
| PALLD | ORAOV1 | OPCML | OPCML |
| PARN | OVCAS1 | ORAOV1 | ORAOV1 |
| PBOV1 | PALB2 | OVCAS1 | OVCAS1 |
| PCA3 | PALLD | PALB2 | PALB2 |
| PCAP | PBOV1 | PALLD | PALLD |
| PCAT1 | PCA3 | PBOV1 | PBOV1 |
| PCAT2 | PCAP | PCA3 | PCA3 |
| PCAT29 | PCAT1 | PCAP | PCAP |
| PCAT4 | PCAT18 | PCAT1 | PCAT1 |
| PDGFRL | PCAT19 | PCAT18 | PCAT18 |
| PHB1 | PCAT2 | PCAT19 | PCAT19 |
| PIK3CA | PCAT29 | PCAT2 | PCAT2 |
| PLA2G2A | PCAT4 | PCAT29 | PCAT29 |
| PLF | PDGFRL | PCAT4 | PCAT4 |
| PMS2 | PHB1 | PDGFRL | PDGFRL |
| POLE | PIK3CA | PHB1 | PHB1 |
| PPM1D | PLA2G2A | PIK3CA | PIK3CA |
| PPP2R1B | PMS2 | PLA2G2A | PLA2G2A |
| PRKN | POLD1 | PMS2 | PMS2 |
| PRNCR1 | POLE | POLD1 | POLD1 |
| PTEN | PPM1D | POLE | POLE |
| PTPN12 | PPP2R1B | PPM1D | PPM1D |
| PTPRJ | PRKN | PPP2R1B | PPP2R1B |
| RABL3 | PRNCR1 | PRKN | PRKN |
| RAD51 | PTEN | PRNCR1 | PRNCR1 |
| RAD51C | PTPN12 | PTEN | PTEN |
| RAD51D | PTPRJ | PTPN12 | PTPN12 |
| RAD54B | RABL3 | PTPRJ | PTPRJ |
| RAD54L | RAD51 | RABL3 | RABL3 |
| RB1 | RAD51C | RAD51 | RAD51 |
| RB1CC1 | RAD51D | RAD51C | RAD51C |
| RNASEL | RAD54B | RAD51D | RAD51D |
| RNF43 | RAD54L | RAD54B | RAD54B |
| RPA1 | RB1 | RAD54L | RAD54L |
| SASH1 | RB1CC1 | RB1 | RB1 |
| SCAI | RHBDF2 | RB1CC1 | RB1CC1 |
| SCHLAP1 | RNASEL | RHBDF2 | RHBDF2 |
| SCLC1 | RNF43 | RNASEL | RNASEL |
| SFTPA1 | SASH1 | RNF43 | RNF43 |
| SFTPA2 | SCAI | SASH1 | SASH1 |
| SFTPB | SCHLAP1 | SCAI | SCAI |
| SFTPC | SCLC1 | SCHLAP1 | SCHLAP1 |
| SFTPD | SLC22A1L | SCLC1 | SCLC1 |
| SLC22A1L | SMAD4 | SLC22A1L | SLC22A1L |
| SLC34A2 | SMAD7 | SMAD4 | SMAD4 |
| SNCG | SNCG | SMAD7 | SMAD7 |
| SRGAP1 | SRC | SNCG | SNCG |
| TAPVR1 | SRGAP1 | SRC | SRC |
| TBX4 | ST3 | SRGAP1 | SRGAP1 |
| TERC | STK11 | STK11 | STK11 |
| TERT | TFF1 | TFF1 | TFF1 |
| TGFBR2 | TGFBR2 | TGFBR2 | TGFBR2 |
| TLR2 | TLR2 | TLR2 | TLR2 |
| TP53 | TP53 | TP53 | TP53 |
| TSG11 | TSG11 | TSG11 | TSG11 |
| VOPP1 | UCA1 | UCA1 | UCA1 |
| XRCC3 | VOPP1 | VOPP1 | VOPP1 |
| ZCCHC8 | XRCC3 | XRCC3 | XRCC3 |
| ZFHX3 | ZFHX3 | ZFHX3 | ZFHX3 |
| ABC3 | ACH | ACH | ACH |
| ACH | ACSTD1 | ACSTD1 | ACSTD1 |
| ACSTD1 | ACVRLK4 | ACVRLK4 | ACVRLK4 |
| ACVRLK4 | ADH2 | ADH2 | ADH2 |
| ADH2 | AIB1 | AIB1 | AIB1 |
| AFURS1 | ALPS4 | ALPS4 | ALPS4 |
| AILJK | ASV | ASV | ASV |
| ALPS4 | ATA | ATA | ATA |
| ATA | ATBF1 | ATBF1 | ATBF1 |
| ATBF1 | BACH1 | BACH1 | BACH1 |
| BACH1 | BC10 | BC10 | BC10 |
| BCSG1 | BCEI | BCEI | BCEI |
| BEK | BCSG1 | BCSG1 | BCSG1 |
| BHD | BEK | BEK | BEK |
| BSCL3 | BHD | BHD | BHD |
| BUBR1 | BUBR1 | BUBR1 | BUBR1 |
| BWSCR1A | BWSCR1A | BWSCR1A | BWSCR1A |
| C9orf126 | C9orf126 | C9orf126 | C9orf126 |
| CAGE1 | CAGE1 | CAGE1 | CAGE1 |
| CARLO5 | CARLO5 | CARLO5 | CARLO5 |
| CC1 | CC1 | CC1 | CC1 |
| CD49C | CKN2 | CKN2 | CKN2 |
| CKN2 | CLOVE | CLOVE | CLOVE |
| CLG | CMM6 | CMM6 | CMM6 |
| CLOVE | COCA1 | COCA1 | COCA1 |
| CMM6 | COCA2 | COCA2 | COCA2 |
| COCA1 | COPEB | COPEB | COPEB |
| COCA2 | COT | COT | COT |
| COPEB | CRAC1 | CRAC1 | CRAC1 |
| COT | CRCS1 | CRCS1 | CRCS1 |
| CRAC1 | CRCS10 | CRCS10 | CRCS10 |
| CRCS1 | CRCS12 | CRCS12 | CRCS12 |
| CRCS12 | CT47A13 | CT47A13 | CT47A13 |
| CWS6 | CWS6 | CWS6 | CWS6 |
| DAN | CYP2A3 | CYP2A3 | CYP2A3 |
| DD3 | DD3 | DD3 | DD3 |
| DEP1 | DEP1 | DEP1 | DEP1 |
| DIA4 | DHTR | DHTR | DHTR |
| DLC1 | DIA4 | DIA4 | DIA4 |
| DOC1 | DLC1 | DLC1 | DLC1 |
| ECOP | DOC1 | DOC1 | DOC1 |
| EIF4G | ECOP | ECOP | ECOP |
| EMS1 | EIF4G | EIF4G | EIF4G |
| EPHT3 | EMS1 | EMS1 | EMS1 |
| ESR | EPHT3 | EPHT3 | EPHT3 |
| FAL1 | ESR | ESR | ESR |
| FANCD1 | FAL1 | FAL1 | FAL1 |
| FANCN | FANCD1 | FANCD1 | FANCD1 |
| FANCO | FANCN | FANCN | FANCN |
| FARSLB | FANCO | FANCO | FANCO |
| FKHL15 | FKHL15 | FKHL15 | FKHL15 |
| FKHL5 | FRP1 | FRP1 | FRP1 |
| FRP1 | GDEP | GDEP | GDEP |
| GCN2 | GS | GS | GS |
| GDEP | GTBP | GTBP | GTBP |
| GS | HLRCC | HLRCC | HLRCC |
| GTBP | HNPCC6 | HNPCC6 | HNPCC6 |
| HLRCC | HNPCC7 | HNPCC7 | HNPCC7 |
| HNPCC6 | HPC13 | HPC13 | HPC13 |
| HNPCC7 | HPC2 | HPC2 | HPC2 |
| HPC13 | HPC9 | HPC9 | HPC9 |
| HPC2 | HR54 | HR54 | HR54 |
| HPC9 | HRG22 | HRG22 | HRG22 |
| HR54 | IRHOM2 | IRHOM2 | IRHOM2 |
| ICPPS | KIAA0790 | KIAA0790 | KIAA0790 |
| KIAA0790 | KIAA0903 | KIAA0903 | KIAA0903 |
| KIAA0903 | KIAA0992 | KIAA0992 | KIAA0992 |
| KIAA0992 | KIAA1304 | KIAA1304 | KIAA1304 |
| KIAA1304 | KKLC1 | KKLC1 | KKLC1 |
| KRAS2 | KRAS2 | KRAS2 | KRAS2 |
| LAS1 | LAS1 | LAS1 | LAS1 |
| LINC00340 | LINC00178 | LINC00178 | LINC00178 |
| LINC00860 | LINC00340 | LINC00340 | LINC00340 |
| LINC00912 | LINC00860 | LINC00468 | LINC00860 |
| LINC00990 | LINC00912 | LINC00860 | LINC00912 |
| LINC01244 | LINC00990 | LINC00912 | LINC00990 |
| LINC01245 | LINC01092 | LINC00990 | LINC01092 |
| LINC01458 | LINC01190 | LINC01092 | LINC01190 |
| LNCR2 | LINC01244 | LINC01190 | LINC01244 |
| MAC25 | LINC01245 | LINC01244 | LINC01245 |
| MAR | LINC01458 | LINC01245 | LINC01458 |
| MARS | LNCR2 | LINC01458 | LNCR2 |
| MCH4 | LOC255313 | LNCR2 | LOC255313 |
| MCH5 | MADH4 | LOC255313 | MADH4 |
| MMAC1 | MADH7 | MADH4 | MADH7 |
| MTS1 | MAR | MADH7 | MAR |
| MVCD4 | MCH4 | MAR | MCH4 |
| MYH | MCH5 | MCH4 | MCH5 |
| NDNL2 | MMAC1 | MCH5 | MMAC1 |
| NEDSDV | MRMV1 | MMAC1 | MRMV1 |
| NGL | MTCL1AS1 | MRMV1 | MTCL1AS1 |
| NISBD2 | MTS1 | MTCL1AS1 | MTS1 |
| NMOR2 | MVCD4 | MTS1 | MVCD4 |
| NS7 | MYH | MVCD4 | MYH |
| OIAS | NEDSDV | MYH | NEDSDV |
| P53 | NGL | NEDSDV | NGL |
| PARK2 | NISBD2 | NGL | NISBD2 |
| PCA2 | NMOR2 | NISBD2 | NMOR2 |
| PCAT114 | NS7 | NMOR2 | NS7 |
| PCAT8 | ODCRCS | NS7 | ODCRCS |
| PDGRL | P53 | ODCRCS | P53 |
| PFBMFT5 | PARK2 | P53 | PARK2 |
| PFBMFT6 | PCA2 | PARK2 | PCA2 |
| PHBP | PCAT114 | PCA2 | PCAT114 |
| PHN | PCAT8 | PCAT114 | PCAT8 |
| PLA2B | PDGRL | PCAT8 | PDGRL |
| PMSL2 | PHBP | PDGRL | PHBP |
| PNCA5 | PJS | PHBP | PJS |
| POIKTMP | PLA2B | PJS | PLA2B |
| PPH1 | PMSL2 | PLA2B | PMSL2 |
| PRAD1 | PNCA5 | PMSL2 | PNCA5 |
| PRO1073 | PRAD1 | PNCA5 | PRAD1 |
| PSCP | PSCP | PRAD1 | PSCP |
| PTPG1 | PTPG1 | PSCP | PTPG1 |
| PULAM | RAD51L3 | PTPG1 | RAD51L3 |
| RAD51L3 | RAD53 | RAD51L3 | RAD53 |
| RECA | RECA | RAD53 | RECA |
| RNF124 | RNF124 | RECA | RNF124 |
| RNS4 | RNS4 | RNF124 | RNS4 |
| SAR1 | RSTS2 | RNS4 | RSTS2 |
| SCAL1 | SAR1 | RSTS2 | SAR1 |
| SFTB3 | SCAL1 | SAR1 | SCAL1 |
| SFTP1 | SH2D3B | SCAL1 | SH2D3B |
| SFTP2 | STK15 | SH2D3B | STK15 |
| SFTP4 | TAOS1 | STK15 | TAOS1 |
| SH2D3B | TIL4 | TAOS1 | TIL4 |
| SMAD9 | TITF1 | TIL4 | TITF1 |
| SPA2 | TNFSF6 | TITF1 | TNFSF6 |
| TAOS1 | TXBP181 | TNFSF6 | TXBP181 |
| TASK | UROC28 | TXBP181 | UROC28 |
| TCS1 | UVO | UROC28 | UVO |
| TIL4 | WIP1 | UVO | WIP1 |
| TITF1 | AAT3 | WIP1 | AAT3 |
| TNFSF6 | ALK4 | AAT3 | ALK4 |
| TRC3 | ALPS2 | ALK4 | ALPS2 |
| TXBP181 | ALPS2B | ALPS2 | ALPS2B |
| UROC28 | APT1LG1 | ALPS2B | APT1LG1 |
| UVO | AT1 | APT1LG1 | AT1 |
| WIP1 | AURORA2 | AT1 | AURORA2 |
| AAT3 | BCD1 | AURORA2 | BCD1 |
| ACDMPV | BCL1 | BCD1 | BCL1 |
| ALK4 | BROVCA1 | BCL1 | BROVCA1 |
| ALPS2 | BROVCA2 | BROVCA1 | BROVCA2 |
| ALPS2B | BROVCA3 | BROVCA2 | BROVCA3 |
| APT1LG1 | BROVCA4 | BROVCA3 | BROVCA4 |
| AT1 | CAPOK | BROVCA4 | CAPOK |
| BCD1 | CARLO1 | CAPOK | CARLO1 |
| BCL1 | CARLO2 | CARLO1 | CARLO2 |
| BROVCA1 | CARLO4 | CARLO2 | CARLO4 |
| BROVCA2 | CARLO6 | CARLO4 | CARLO6 |
| BROVCA3 | CARLO7 | CARLO6 | CARLO7 |
| BROVCA4 | CFD1 | CARLO7 | CFD1 |
| CAPOK | CHK2 | CFD1 | CHK2 |
| CARLO1 | COFS1 | CHK2 | COFS1 |
| CARLO2 | COXPD17 | COFS1 | COXPD17 |
| CARLO4 | CRCS3 | COXPD17 | CRCS3 |
| CARLO6 | CRCS4 | CRCS3 | CRCS4 |
| CARLO7 | CUDR | CRCS4 | CUDR |
| CFD1 | CXorf61 | CUDR | CXorf61 |
| CGL3 | CYP2A | CXorf61 | CYP2A |
| COFS1 | DIRA | CYP2A | DIRA |
| COLEC5 | DPC4 | DIRA | DPC4 |
| COXPD17 | DRT | DPC4 | DRT |
| CRCS4 | EST | DRT | EST |
| DIRA | ESTRR | EST | ESTRR |
| DKCB6 | EVR7 | ESTRR | EVR7 |
| DRT | FANCJ | EVR7 | FANCJ |
| EST | FCC1 | FANCJ | FCC1 |
| EST2 | FILS | FCC1 | FILS |
| ESTRR | FPC | FILS | FPC |
| EVR7 | GASP | FPC | GASP |
| FANCJ | GLM2 | GASP | GLM2 |
| FCC1 | HGFAL | GLM2 | HGFAL |
| FILS | HGPPS2 | HGFAL | HGPPS2 |
| FPC | HNPCC2 | HGPPS2 | HNPCC2 |
| GAPB3 | HNPCC4 | HNPCC2 | HNPCC4 |
| GASP | HNPCC5 | HNPCC4 | HNPCC5 |
| GLM2 | HPC12 | HNPCC5 | HPC12 |
| HGFAL | HRAD54 | HPC12 | HRAD54 |
| HNPCC2 | IMPT1 | HRAD54 | IMPT1 |
| HNPCC4 | JDVS | IMPT1 | JDVS |
| HNPCC5 | KIAA0203 | JDVS | KIAA0203 |
| HPC12 | KIAA1020 | KIAA0203 | KIAA1020 |
| HRAD54 | LCAM | KIAA1020 | LCAM |
| ILD1 | LFS1 | LCAM | LFS1 |
| IMD100 | LINC00913 | LFS1 | LINC00913 |
| IMPT1 | LKB1 | LINC00913 | LKB1 |
| JDVS | MCAP | LKB1 | MCAP |
| KIAA0203 | MCUL1 | MCAP | MCUL1 |
| KIAA1338 | MDPL | MCUL1 | MDPL |
| LCAM | MKHK2 | MDPL | MKHK2 |
| LFS1 | MRMV2 | MKHK2 | MRMV2 |
| LINC00913 | MVA1 | MRMV2 | MVA1 |
| MADH6 | NEU | MVA1 | NEU |
| MAGEG1 | NKX2A | NEU | NKX2A |
| MCAP | NMOR1 | NKX2A | NMOR1 |
| MCUL1 | NMTC2 | NMOR1 | NMTC2 |
| MRMV2 | NS6 | NMTC2 | NS6 |
| MTRNS | NSP2 | NS6 | NSP2 |
| MVA1 | P16 | NSP2 | P16 |
| NEU | PAOD2 | P16 | PAOD2 |
| NKX2A | PARK18 | PAOD2 | PARK18 |
| NMOR1 | PDJ | PARK18 | PDJ |
| NMTC2 | PLA2L | PDJ | PLA2L |
| NS6 | PNCA1 | PLA2L | PNCA1 |
| NSP2 | PNCA3 | PNCA1 | PNCA3 |
| P16 | PPP1R54 | PNCA3 | PPP1R54 |
| PAOD2 | PRCA1 | PPP1R54 | PRCA1 |
| PAPPAS | PRLTS | PRCA1 | PRLTS |
| PARK18 | RASK2 | PRLTS | RASK2 |
| PDJ | SCKL1 | RASK2 | SCKL1 |
| PLA2L | SRC1 | SCKL1 | SRC1 |
| PNCA1 | SSPCS | SRC1 | SSPCS |
| PNCA3 | TFM | SSPCS | TFM |
| POVD1 | TITF2 | TFM | TITF2 |
| PPH4 | TNRC14 | TITF2 | TNRC14 |
| PPH5 | TOC | TNRC14 | TOC |
| PPP1R54 | TROP1 | TOC | TROP1 |
| PRCA1 | UC28 | TROP1 | UC28 |
| PRLTS | BAIPRCK | UC28 | BAIPRCK |
| RAMSVPS | BCC7 | BAIPRCK | BCC7 |
| RASK2 | BTAK | BCC7 | BTAK |
| RILDBC1 | BTPS2 | BTAK | BTPS2 |
| SCKL1 | CDS1 | BTPS2 | CDS1 |
| SMDP1 | CMNS | CDS1 | CMNS |
| SMDP2 | CSB | CMNS | CSB |
| SMDP3 | CWS1 | CSB | CWS1 |
| SSPCS | DUH1 | CWS1 | DUH1 |
| TITF2 | DUP15q | DUH1 | DUP15q |
| TR | ECAD | DUP15q | ECAD |
| TROP1 | ERK | ECAD | ERK |
| UC28 | FANCR | ERK | FANCR |
| BAIPRCK | FASL | FANCR | FASL |
| BCC7 | FCTCS | FASL | FCTCS |
| BTPS2 | FMRD | FCTCS | FMRD |
| CMNS | FSAP | FMRD | FSAP |
| CSB | GLM3 | FSAP | GLM3 |
| CWS1 | HER2 | GLM3 | HER2 |
| DKCA1 | HNPCC1 | HER2 | HNPCC1 |
| DKCA2 | HPC1 | HNPCC1 | HPC1 |
| DUH1 | IMAGEI | HPC1 | IMAGEI |
| DUP15q | JIP | IMAGEI | JIP |
| ECAD | JWS | JIP | JWS |
| ERK | M4S1 | JWS | M4S1 |
| FANCR | MCM | M4S1 | MCM |
| FASL | MFS2 | MCM | MFS2 |
| FCTCS | MLM | MFS2 | MLM |
| FMRD | MMRCS1 | MLM | MMRCS1 |
| FSAP | MMRCS3 | MMRCS1 | MMRCS3 |
| GLM3 | MMRCS4 | MMRCS3 | MMRCS4 |
| HER2 | MOM1 | MMRCS4 | MOM1 |
| HNPCC1 | NS | MOM1 | NS |
| HPC1 | P450C2A | NS | P450C2A |
| ILD2 | PNCA4 | P450C2A | PNCA4 |
| IMAGEI | SBMA | PNCA4 | SBMA |
| JEB7 | THC6 | SBMA | THC6 |
| JWS | TPL2 | THC6 | TPL2 |
| LICS | TTF1 | TPL2 | TTF1 |
| M4S1 | TTF2 | TTF1 | TTF2 |
| MCM | ZF9 | TTF2 | ZF9 |
| METRS | ALPS1B | ZF9 | ALPS1B |
| MFS2 | ARK1 | ALPS1B | ARK1 |
| MLM | ARMD5 | ARK1 | ARMD5 |
| MMRCS1 | BCDS1 | ARMD5 | BCDS1 |
| MMRCS3 | BMFS5 | BCDS1 | BMFS5 |
| MMRCS4 | C15DUPq | BMFS5 | C15DUPq |
| MOM1 | CFC2 | C15DUPq | CFC2 |
| NS | CMM2 | CFC2 | CMM2 |
| PFBMFT4 | DESMD | CMM2 | DESMD |
| PNCA4 | FANCS | DESMD | FANCS |
| PPH2 | KD | FANCS | KD |
| PPH3 | LDS2 | KD | LDS2 |
| PVOD2 | LFS2 | LDS2 | LFS2 |
| TPL2 | MCMTC | LFS2 | MCMTC |
| TTF1 | MIC18 | MCMTC | MIC18 |
| TTF2 | MMRCS2 | MIC18 | MMRCS2 |
| ZF9 | MYHRS | MMRCS2 | MYHRS |
| ALPS1B | NCMS | MYHRS | NCMS |
| ARMD5 | NMTC1 | NCMS | NMTC1 |
| BCDS1 | NMTC4 | NMTC1 | NMTC4 |
| BMFS5 | NMTC5 | NMTC4 | NMTC5 |
| C15DUPq | PCBC | NMTC5 | PCBC |
| CFC2 | PNCA2 | PCBC | PNCA2 |
| CMM2 | TK14 | PNCA2 | TK14 |
| DESMD | VSCN2 | TK14 | VSCN2 |
| DKCB4 | BBDS | VSCN2 | BBDS |
| FANCS | CAPB | BBDS | CAPB |
| ILLD | CWS5 | CAPB | CWS5 |
| LCCNS | DIAR5 | CWS5 | DIAR5 |
| LDS2 | RALD | DIAR5 | RALD |
| MCMTC | SMAX1 | RALD | SMAX1 |
| MIC18 | STK6 | SMAX1 | STK6 |
| MMRCS2 | UVSS1 | STK6 | UVSS1 |
| NCMS | AIK | UVSS1 | AIK |
| NMTC1 | BDPLT22 | AIK | BDPLT22 |
| NMTC4 | CLAPO | BDPLT22 | CLAPO |
| NMTC5 | HNPCC8 | CLAPO | HNPCC8 |
| PCBC | HYSP1 | HNPCC8 | HYSP1 |
| PFBMFT2 | OES | HYSP1 | OES |
| PNCA2 | POF11 | OES | POF11 |
| TK14 | CCM4 | POF11 | CCM4 |
| VSCN2 | MIR21 | CCM4 | MIR21 |
| BBDS | NBN | MIR21 | NBN |
| CAPB | C11orf65 | C11orf65 | MIR221 |
| CMT2U | MIR34A | MET | MIR143 |
| CWS5 | RAD50 | NBN | C11orf65 |
| DIAR5 | MET | MIR34A | MIR34A |
| PFBMFT1 | RET | RET | MIR127 |
| RALD | NF1 | MIR145 | RAD50 |
| UVSS1 | MIR221 | RAD50 | RET |
| BDPLT22 | MIR17 | PMS1 | MET |
| CLAPO | MIR145 | CTNNA1 | LRRC56 |
| CMM9 | MIR146A | MIR141 | MIR10B |
| HNPCC8 | MIR205 | MIR126 | MIR17 |
| OES | MRE11 | BLM | MIR145 |
| POF11 | MIR214 | RAD51L3-RFFL | MRE11 |
| TTD9 | MIR200B | MIR143 | RAD51L3-RFFL |
| CCM4 | MIR222 | MIR203A | MIR222 |
| CHEK2 | MIR20A | MIR127 | NF1 |
| MIR21 | MIR143 | MIR221 | MIR146A |
| MET | MIR200A | NF1 | TERT |
| NBN | MIR126 | MIR34C | MIR141 |
| MIR34A | MIR141 | MRE11 | MIR205 |
| ALK | TERT | MIR17 | MIR200B |
| STK11 | MIR200C | MSH3 | CTNNA1 |
| MIR221 | MIR125A | IGF2 | MIR200A |
| MIR17 | MIR15A | BMPR1A | MIR200C |
| SMAD4 | PTCH1 | MIR34B | MIR20A |
| MIR145 | CTNNA1 | MIR222 | MIR126 |
| MIR222 | MIR127 | MIR451A | TSC1 |
| MIR200B | MIR155 | PPARG | MIR203A |
| MIR126 | MDM2 | MIR146A | MIR125A |
| MIR200C | ALK | MIR200B | MIR31 |
| RET | MIR31 | PIK3R1 | MDM2 |
| MIR205 | MIR203A | MIR200C | CDKN1B |
| MIR20A | BLM | MIR20A | MIR15A |
| MIR125A | SMARCA4 | FBXW7 | BLM |
| C11orf65 | MIR34C | MDM2 | TP63 |
| MIR141 | BMPR1A | MIR125A | SMARCA4 |
| POLD1 | CDKN1B | MIR200A | MAP2K1 |
| RAD50 | MIR27A | MIR192 | ALK |
| MIR200A | DICER1 | TERT | MIR155 |
| MAP2K1 | MIR106B | ALK | MIR34C |
| EGFR-AS1 | KIT | DICER1 | KIT |
| MIR34C | MIR210 | MIR205 | MSH3 |
| MIR15A | FANCC | SMARCA4 | MIR106B |
| AXIN2 | MIRLET7C | MIR31 | CDK4 |
| ROS1 | MIR93 | ARID1A | DICER1 |
| NF1 | BAP1 | MIRLET7A1 | BAP1 |
| SLC22A18 | MYC | MIR155 | MIR27A |
| RAD51L3-RFFL | BCL2 | MIR140 | FANCC |
| MIR34B | XRCC2 | GREM1 | PTCH1 |
| MIRLET7C | TSC2 | FANCM | MIR182 |
| DICER1 | TSC1 | SOX9 | MIR210 |
| MIR93 | MAP2K1 | MIR215 | MYC |
| MIR146A | MIR34B | MIR15A | MIR96 |
| MIRLET7D | MIR29A | MIR27A | BMPR1A |
| MIR29A | SDHB | MIR100 | IGF2 |
| MRE11 | CDK4 | MT-CO1 | MIR34B |
| MIR429 | STAT3 | FANCC | VEGFA |
| MIR18A | MIR10B | BCL10 | MIR214 |
| MIR19A | MIR451A | CDKN1B | PIK3R1 |
| MIR499A | IGF2 | MYC | XRCC2 |
| MIR183 | MIR23B | XRCC2 | MIR93 |
| TSC2 | MIR182 | FZD3 | HNF1B |
| MIRLET7G | VEGFA | MIR106B | MIR23B |
| MIR29C | IL6 | MIR26A1 | MIR223 |
| TSC1 | PPARG | VEGFA | MIR30E |
| SMARCA4 | PMS1 | PTCH1 | MIRLET7C |
| MIRLET7B | MTHFR | MAP2K1 | TSC2 |
| KIT | MIR96 | MIR342 | MIR451A |
| MIR486-1 | MIR429 | KIT | PPARG |
| CYP2A6 | TNF | MIR10B | PMS1 |
| IL6 | MIR30E | DMD | CDKN1A |
| MIR155 | FBXW7 | CDK4 | FBXW7 |
| MIR185 | AOPEP | BAP1 | BCL2 |
| AR | MTOR | MIR19A | ERCC2 |
| TGFB1 | MIR373 | MIR182 | STAT3 |
| MIR107 | CDKN1A | PKHD1 | MIR29C |
| MIR130A | MIR223 | MIR93 | SDHB |
| MIR143 | SOX9 | MIR210 | GSTM1 |
| MDM2 | MIR18A | AMER1 | TNF |
| CDK4 | MIRLET7D | MIR29A | MIR429 |
| CDKN1B | PIK3R1 | MIR96 | MIR19A |
| MIR98 | MIR499A | TSC1 | FANCM |
| MIR128-2 | MIRLET7B | AOPEP | MTOR |
| MXRA5 | MIR19A | TSC2 | IL6 |
| TNF | MIR16-1 | STAT3 | MIR18A |
| MIR30D | RUNX1 | PTGS2 | ERBB3 |
| MIR133B | LRRC56 | MYO1B | MIRLET7D |
| BLM | MMP2 | BCL2 | AOPEP |
| BAP1 | VHL | MIRLET7C | MIR100 |
| MIR31 | MIRLET7G | MIR214 | MIR16-1 |
| MIR203A | MIR204 | IL6 | MIR29A |
| MIR137 | HNF1B | RBFOX1 | MUC1 |
| MIRLET7A3 | MIR335 | MT-ND1 | MIRLET7G |
| PTCH1 | FHIT | MIR18A | ERCC1 |
| MSH3 | PTGS2 | MIR223 | MIR185 |
| BAX | TGFB1 | SDHB | EGF |
| STAT3 | CDC73 | INS-IGF2 | MIR204 |
| MIRLET7E | MIR183 | TCERG1 | VHL |
| MIR27A | MIR140 | SLC9A9 | RUNX1 |
| CTNNA1 | MEN1 | MIR429 | GSTP1 |
| MIR210 | MIR29C | MIRLET7G | MIR373 |
| MIR372 | MIR146B | ERCC2 | SLC22A18 |
| MIR197 | HIF1A | TNF | MIR335 |
| MYC | MIR25 | CDKN1A | MIR499A |
| FANCC | KLLN | KLLN | MMP2 |
| BMPR1A | EGFR-AS1 | MIR30E | PTGS2 |
| MIR127 | ERCC2 | MTOR | MEN1 |
| VEGFA | MIR192 | TGFB1 | MSR1 |
| IGF2 | MAPK1 | ROS1 | MMP9 |
| SRC | ABRAXAS1 | MIR25 | MIR192 |
| TP73 | CASP3 | MIR16-1 | MIR146B |
| EP300 | MIR107 | MIR183 | MAPK1 |
| LRRC56 | TP73 | GSTM1 | MIR140 |
| MIR106B | MIR486-1 | SLC22A18 | CD274 |
| PGBD3 | MIR195 | MMP2 | MIR25 |
| PPARG | TNFRSF10B | MTHFR | ARID1A |
| PIK3R1 | MMP9 | MIR335 | H19 |
| MIR451A | MIR185 | MIR373 | MIR133B |
| XRCC2 | BIRC5 | MMP9 | ROS1 |
| MIR182 | SDHD | MIRLET7D | CASP3 |
| BCL2 | MIR206 | RUNX1 | MIR183 |
| SLMAP | PDGFRA | MIR206 | EGFR-AS1 |
| AURKA | MIR199B | GSTP1 | KLLN |
| FBXW7 | NOTCH1 | MIRLET7B | BIRC5 |
| ERCC2 | MIRLET7A1 | MIR185 | XRCC1 |
| MIR30E | MIR196A2 | HNF1B | MIR195 |
| MIR96 | MIR98 | TYMS | TGFB1 |
| MIR223 | MIR215 | MIR23B | MIR486-1 |
| CD274 | IL10 | MEN1 | FGFR1 |
| MIR204 | GSTM1 | MIR204 | SRD5A2 |
| CXCL8 | MIR193B | CDC73 | SDHA |
| VHL | MIR100 | MIR499A | NAT2 |
| MIR214 | ARID1A | MIR107 | SDHD |
| MTOR | MIR148A | HIF1A | CXCL8 |
| IL10 | CXCR4 | VHL | MIR148A |
| FGFR1 | CD274 | MIR29C | SMARCB1 |
| MUC1 | SMARCB1 | CD274 | MKI67 |
| AOPEP | PTPN11 | SDHD | POLK |
| CDKN1A | EGF | MGMT | MIR206 |
| PTPN11 | MIR122 | ERCC1 | MMP1 |
| MIR10B | PGR | EGFR-AS1 | PDCD1 |
| MIR335 | ERCC1 | NTHL1 | MIRLET7B |
| MAPK1 | POLK | MUC1 | KLK3 |
| GSTM1 | TP63 | EGF | FHIT |
| MMP2 | MIR150 | MIR133B | WWOX |
| MMP9 | MIR133B | XRCC1 | MIR107 |
| ERCC1 | MIR128-2 | ABRAXAS1 | MIR150 |
| PTGS2 | MIR483 | TP73 | GSTT1 |
| FANCM | POT1 | MIR146B | MIR199B |
| SOX9 | GNAS | MIR128-2 | MIR193B |
| MIR140 | MAP3K1 | MIR150 | MIRLET7A1 |
| PMS1 | MKI67 | MIR137 | ABCB1 |
| CYP1A1 | H19 | MSR1 | KRT20 |
| RUNX1 | MIRLET7E | CASP3 | SOX9 |
| IFNG | WWOX | CEACAM5 | RASSF1 |
| MIR146B | MIRLET7A3 | MAPK1 | MIR196A2 |
| SDHB | GSTP1 | PDGFRA | EZH2 |
| PDGFRA | MUC1 | CXCR4 | CD44 |
| CDC73 | PARP1 | MIR483 | TOP2A |
| MIR25 | MIR130A | TNFRSF10B | OGG1 |
| HIF1A | MIR15B | FAN1 | SUFU |
| GSTP1 | MIR128-1 | ABCB1 | MIR10A |
| FHIT | CD44 | LRRC56 | MAP3K1 |
| NOTCH1 | BCL10 | MIR195 | BCL2L1 |
| SERPINA1 | SMAD3 | BIRC5 | MIR215 |
| MSR1 | CXCL8 | AXIN1 | PDGFRA |
| EGF | MIR137 | IGF1 | HIF1A |
| CASP3 | FAS | SMAD3 | SMAD3 |
| MIR199B | MIR331 | CDKN3 | IDH1 |
| FAS | SDHA | MIR196A2 | TERC |
| CTLA4 | TERC | OGG1 | TP73 |
| POLK | RASSF1 | MIR199B | MIR23A |
| MIR192 | HLA-DQB1 | FHIT | PSCA |
| MIR148A | CDKN3 | MIR372 | KRT7 |
| MIR150 | HOTAIR | TGFBR1 | TYMS |
| MIR373 | VEGFC | MIR486-1 | MIR128-2 |
| CXCR4 | TGFBR1 | FAS | MALAT1 |
| SMAD3 | SUFU | MIR15B | CYP1A1 |
| MIR23B | MIR372 | MIR106A | MIR331 |
| RARB | MIR30D | JUN | TGFBR1 |
| HNF1B | MIR10A | MT-CYB | DNMT1 |
| XRCC1 | MIR106A | POLK | CTAG1B |
| ABCB1 | XRCC1 | ESR2 | KRT19 |
| ABRAXAS1 | MIR196B | SMARCB1 | MIR98 |
| KDR | MIR197 | MT-CO2 | KDR |
| JAK2 | JAK2 | JAK2 | IL2 |
| CFTR | FGFR1 | H19 | CXCR4 |
| MIRLET7A1 | CDK1 | CXCL8 | MIR130A |
| SMARCB1 | MGMT | NOTCH1 | IL10 |
| TNFRSF10B | MALAT1 | CYP1A1 | BCL10 |
| BIRC5 | MIR22 | CTLA4 | MIR137 |
| H19 | MIR26A1 | EZH2 | MIR483 |
| MIR16-1 | CTLA4 | CD44 | CDKN3 |
| NFE2L2 | CASP9 | MIR148A | MIR128-1 |
| NFKB1 | JUN | SRD5A2 | PLAU |
| ESR2 | MIRLET7I | GNAS | BRINP1 |
| IDH1 | RRAS2 | MAP3K1 | TNFRSF10B |
| JUN | MIR342 | MIR122 | PTPN11 |
| MIR195 | KRT7 | IL10 | CASP9 |
| KLLN | MIR9-3 | KDR | MTHFR |
| TGFBR1 | ERBB3 | WWOX | CTAG2 |
| ARID1A | WT1 | MIR193B | AATBC |
| MGMT | MIR181A1 | MIR10A | TNFSF10 |
| HGF | ESR2 | DPYD | TYMP |
| MIR100 | MEG3 | MMP7 | LZTS1 |
| SDHD | IFNG | MIR98 | FAS |
| TP63 | LNMICC | IGFBP3 | ERCC4 |
| IGF1R | ABCB1 | NFKB1 | POT1 |
| MIR206 | MIR449A | SMAD2 | NOTCH1 |
| RAF1 | HERC2 | BCL2L1 | ESR2 |
| BCL10 | FANCD2 | MIR181A1 | FGF2 |
| DCC | BSCL2 | MIRLET7A3 | FN1 |
| MIR483 | IGF1 | MIR128-1 | ISL1 |
| IGF1 | IDH1 | MIR22 | MIRLET7E |
| CEACAM5 | FN1 | DNMT1 | HOTAIR |
| LOC111589215 | KDR | IGF1R | TGFA |
| OGG1 | TWIST1 | FANCE | E2F1 |
| SETD2 | FANCE | ABCG2 | MIR15B |
| PDGFRB | KRT19 | SDHA | MIR181A1 |
| MIR196A2 | CEACAM5 | FGFR1 | MT-CO1 |
| CD44 | CYP1A1 | TWIST1 | MIR26A1 |
| WWOX | MIR142 | PLAU | JAK2 |
| RASSF1 | CDK2 | SDHC | CDKN2B |
| POT1 | MIR502 | MIRLET7E | BSCL2 |
| STAT1 | MYCN | MIR130A | CDK1 |
| SMAD7 | CREBBP | PARP1 | VEGFC |
| ERBB3 | EZH2 | MKI67 | GJA1 |
| SPP1 | DMD | MIR23A | MIR106A |
| SDHA | TOP2A | NAT2 | MIR449A |
| BCL2L1 | TYMP | MIR331 | RRAS2 |
| MIR122 | NFKB1 | PTPN11 | NTHL1 |
| EZH2 | TYMS | MMP1 | MIR342 |
| TYMS | CXCL12 | IFNG | PARP1 |
| MYCN | IL2 | VDR | XIAP |
| SUFU | MIR23A | CDKN2B | MIR372 |
| MTHFR | SDHC | POT1 | GNAS |
| RTEL1 | CYCS | RAF1 | PDGFRB |
| FN1 | MIR224 | IGF2R | CCL2 |
| WT1 | AXIN1 | KRT20 | MIR30D |
| FGF2 | NF2 | TYMP | CAV1 |
| MIR26A1 | GREM1 | TOE1 | MGMT |
| HLA-DRB1 | CCNB1 | SUFU | MIR22 |
| MAP3K1 | IGF1R | MIR224 | NF2 |
| PLAU | PVT1 | ERBB3 | MIR142 |
| MIR15B | OGG1 | PRKD1 | MIR197 |
| CSF3 | MMP1 | MIR142 | MIRLET7I |
| KRT19 | MIR181A2 | CASP9 | DMD |
| CCL2 | CCNA2 | TOP2A | FANCE |
| ABCG2 | LOC107303340 | IL2 | FANCD2 |
| UCA1 | CTAG1B | DDB2 | ERBB4 |
| HMOX1 | RARB | SETD2 | IGF1 |
| HPC3 | KLK3 | TCF7L2 | RBBP8 |
| HFE | ERCC4 | VEGFC | PVT1 |
| PARP1 | CDKN2B-AS1 | HERC2 | HBEGF |
| GNAS | HLA-DRB1 | MIR30D | GREM1 |
| ERCC4 | RAF1 | RASSF1 | CTLA4 |
| CSF2 | ING1 | PDGFRB | FZD3 |
| MIR106A | TGFA | IDH1 | CEACAM5 |
| CASP9 | BCL2L1 | MIR191 | JUN |
| MIR193B | ABCG2 | MIR197 | AXIN1 |
| MKI67 | MMP14 | FN1 | NFE2L2 |
| CDKN2B | MIR191 | MALAT1 | LOC109504725 |
| MIR375 | NTHL1 | ZEB1 | SMARCE1 |
| PDCD1 | GAS5 | SOD2 | SDHC |
| TOP2A | PLAU | MAP3K6 | MIR224 |
| NFKBIA | TIMP3 | RRAS2 | CCNE1 |
| SRD5A2 | XIAP | KRT7 | MEG3 |
| KRT7 | IGF2R | CXCL12 | NAT1 |
| SFTA3 | AMER1 | TGFA | MIR661 |
| IL2 | CCEPR | PROM1 | CDK2 |
| ENO2 | DROSHA | HOTAIR | MIR502 |
| IL4 | FGF2 | CDK1 | PLAUR |
| ABCC1 | IGFBP3 | GSTT1 | PGR |
| HPC6 | DDB2 | SNAI1 | THBS1 |
| MIR22 | SOD2 | NF2 | HSPB1 |
| DSP | SETD2 | TNFSF10 | WT1 |
| XPA | NFE2L2 | NFKBIA | HNRNPUL2-BSCL2 |
| DDB2 | DNMT1 | MIR181A2 | STAT1 |
| FGF10 | DPYD | MIR9-3 | ZFHX3-AS1 |
| MAX | SOX2 | MIRLET7I | HSP90AA1 |
| PTK2 | HLA-A | MAPK3 | IFNG |
| HPC15 | BMP2 | STAT1 | NFKB1 |
| SOD2 | SPP1 | ERBB4 | ABCG2 |
| RELA | GSTT1 | FGF2 | KRT18 |
| DDR2 | PRKD1 | PDCD1 | PKHD1 |
| VEGFC | CDKN2B | HGF | CCNA2 |
| MAPK8 | PDGFRB | PTK2 | MIR521-1 |
| CTAG1B | FLT4 | GLI1 | GAS5 |
| MEG3 | ITGB1 | SPP1 | TIMP2 |
| MIR181A1 | SLMAP | YAP1 | ENO2 |
| CXCL12 | MUC16 | MXRA5 | TWIST1 |
| DNMT3A | STAT1 | RHOA | SOD2 |
| TNFSF10 | PTK2 | XIAP | IGF1R |
| CDKN3 | PDCD1 | E2F1 | PCNA |
| ING1 | ETV6 | DNMT3A | WRAP53 |
| PGR | NFKBIA | KRT19 | IGF2R |
| TWIST1 | CYP19A1 | FLT1 | MIR9-1 |
| TGFA | CTAG2 | MIR520C | TUG1 |
| LOC110806263 | GDF6 | MMP14 | CDKN2B-AS1 |
| RHOA | SLC2A1 | MIR449A | SETD2 |
| MMP7 | E2F1 | FANCD2 | MT-ND1 |
| FLT4 | CA9 | PCNA | TNFRSF10A |
| MIR128-1 | MIR181B1 | RBBP8 | IGFBP3 |
| ALB | TIMP2 | CDK2 | ALB |
| MIF | PRKAR1A | CYCS | DDB2 |
| MAPK3 | MAPK8 | SNAI2 | CDH2 |
| GLI1 | ABL1 | CDX2 | RAF1 |
| SMAD2 | NEAT1 | ITGB1 | AREG |
| DNMT1 | TNFSF10 | GNG3 | SYP |
| ITGB1 | DNMT3A | AKT2 | TIMP3 |
| GREM1 | CCL2 | ERCC4 | KRT8 |
| MIR331 | DAPK1 | RIPK1 | CREBBP |
| TLR4 | MIR9-1 | SLC2A1 | XPC |
| HOTAIR | PCNA | MAPK8 | CDK6 |
| WRAP53 | NTRK1 | SP1 | MT-CYB |
| MIR142 | MT-ND1 | SOX2 | CA9 |
| IGFBP3 | SMAD2 | CDK6 | HPSE2 |
| RRAS2 | MEOX1 | PVT1 | AMER1 |
| E2F1 | FLNB | CCND2 | UPK3A |
| AKT2 | MAPK3 | MCL1 | XPA |
| ERBB4 | XPA | WRN | MIR191 |
| TIMP1 | MYO1B | MIR661 | MMP14 |
| SOX2 | RHOA | CYP1B1 | MMP7 |
| MT-CO1 | ZEB1 | FLT4 | CYP19A1 |
| ENG | CADM1 | GSK3B | MYCN |
| MIR215 | CDK6 | PGR | RELA |
| FLT1 | HFE | DNMT3B | SLMAP |
| YAP1 | GLI1 | WRAP53 | NFKBIA |
| MIR23A | KRT20 | CHEK1 | CYCS |
| CDK2 | MMP7 | CTAG1B | CXCL12 |
| CYCS | SP1 | ABCC1 | E2F3 |
| CCR6 | MCL1 | MAPK14 | MIR181A2 |
| CDK1 | NPM1 | TOP1 | MAP3K6 |
| FOXP3 | ENO2 | RAC1 | SMAD2 |
| CDK6 | SMARCE1 | MIR24-2 | EFEMP1 |
| XIAP | TIMP1 | CCL2 | VDR |
| TOP1 | IDH2 | MIR375 | DROSHA |
| DROSHA | DKK1 | NFE2L2 | MIR193A |
| SP1 | TLR4 | CDKN2B-AS1 | VIM |
| SNAI2 | RBFOX1 | RELA | LOC107303340 |
| MAPK14 | NME1 | CCNA2 | UPK2 |
| IDH2 | RELA | CCNB1 | ING1 |
| SNAI1 | TUG1 | MUC16 | CFLAR |
| PDGFB | KRT8 | HSP90AA1 | MIR196B |
| GRP | MIR375 | TIMP1 | HGF |
| FANCD2 | VDR | KLK3 | SERPINB5 |
| NF2 | INS-IGF2 | AKT3 | MIR139 |
| SHH | TGFB3 | PLAUR | SPP1 |
| MMP14 | FLT1 | MYCN | LRIG2 |
| CYP1B1 | AIP | BMP6 | MAPK14 |
| MUC16 | FAN1 | CAV1 | ABL1 |
| KRT18 | SNAI2 | LEF1 | DAPK1 |
| MIR10A | SLC9A9 | MUC5AC | MAX |
| MCL1 | MIR196A1 | HSPB1 | SLC2A1 |
| ZEB1 | YAP1 | PGBD3 | GLI1 |
| NAT2 | MIR16-2 | CTSB | S100A4 |
| SDHC | WRAP53 | TLR4 | CDKN1C |
| CREB1 | HGF | PLK1 | EBF3 |
| MIR191 | POU5F1 | MEG3 | NPM1 |
| ABL1 | CSF2 | INS | COL1A1 |
| IL13 | MYO18B | MIR196B | RHOA |
| PCNA | EBAG9 | NME1 | PTK2 |
| HERC2 | NAT2 | FOXP3 | MUC16 |
| TRIP13 | HSP90AA1 | CSF2 | ANXA5 |
| PVT1 | PAX8 | ETV6 | MCL1 |
| NME1 | FOXP3 | SPINK1 | BSG |
| MIR224 | CAV1 | KCNQ1OT1 | SNAI1 |
| NOS2 | HULC | ING1 | FLT1 |
| CHGA | MIR24-2 | FOXM1 | GAPDH |
| SLC2A1 | ERBB4 | HDAC1 | CSF3 |
| CDKN1C | NOTCH3 | KRT18 | RBFOX1 |
| PIK3CG | MIR424 | MIR320A | MYO1B |
| NTHL1 | HSPB1 | BMP4 | ITGB1 |
| AXIN1 | TMEM127 | LZTS1 | CLU |
| HSP90AA1 | MIR199A1 | CYP2E1 | MYH11 |
| DNMT3B | COL1A1 | THBS1 | JUP |
| MIR9-3 | LOC100507346 | WT1 | YAP1 |
| BSCL2 | CSF3 | GAS5 | MIR199A1 |
| DPYD | PIK3CG | TIMP2 | CLCN6 |
| HSPB1 | CHEK1 | CRP | ZEB1 |
| PKHD1 | VIM | CTSD | SOX2 |
| AKT3 | IL4 | TIMP3 | DNMT3A |
| HLA-G | CCR6 | RUNX3 | INS-IGF2 |
| LCAL1 | ABCC1 | NOS2 | SP1 |
| MIR449A | MAP3K7 | AREG | HLA-DQB1 |
| MIR342 | DNMT3B | LOC107303340 | ITGA6 |
| IL17A | AKT2 | CREB1 | MAPK3 |
| PRKCA | PLK1 | RPS6KB1 | IFNA2 |
| BMP6 | TGFB2 | MIR199A1 | SNAI2 |
| RAC1 | LMNA | ABL1 | GCASPC |
| SMARCE1 | GSK3B | GDF15 | CYP1B1 |
| CCNB1 | ERCC5 | BMP2 | LNC-LBCS |
| CRP | CRP | BMI1 | DPYD |
| MIR30A | SNAI1 | HSPA5 | ENG |
| CCL5 | MIR320A | XPA | XIST |
| RBBP8 | XPC | MIR30A | MAPK8 |
| ICAM1 | MAPK14 | CD24 | AIP |
| CCN2 | MME | SLMAP | TCERG1 |
| CHEK1 | RIPK1 | ENG | AKT2 |
| CCNA2 | MIR30A | DROSHA | PRKAR1A |
| MIRLET7I | GDF3 | TGFB2 | FOS |
| POU5F1 | THBS1 | PRKCA | MIR144 |
| MIR502 | BMP4 | CCR6 | MIR24-2 |
| FGF7 | ERG | VIM | ABCC1 |
| HMGB1 | SKP2 | PRKAR1A | GATA3 |
| FANCE | ALB | CYP19A1 | TMEM127 |
| TYMP | FOXM1 | POU5F1 | PDGFB |
| PLAUR | YY1 | ALB | IDH2 |
| MIR520C | MTHFD1 | FOS | HFE |
| PROM1 | BMI1 | CTAG2 | CDX2 |
| SOS1 | SPRY4-IT1 | ABCC2 | FGF1 |
| PLK1 | BMP6 | NEAT1 | CD82 |
| STN1 | NOG | UGT1A1 | MYLK |
| GSTT1 | SYP | TERC | BMP4 |
| CDKN2B-AS1 | AFP | SERPINE1 | CRP |
| SYP | CREB1 | IRS1 | CSF2 |
| MIR661 | CHGA | KLF5 | RARB |
| HLA-B | KRT18 | IL4 | SERPINA3 |
| NRG1 | CDX2 | AFP | HULC |
| DMD | IL2RA | TNFRSF10A | IL4 |
| CADM1 | CRNN | CYP3A4 | IFI27 |
| IGF2R | H2AC18 | FASN | PLK1 |
| KEAP1 | TBXT | CDH2 | SPINK1 |
| AREG | KRT14 | KRT8 | ERG |
| CD24 | FOS | CLDN7 | MIR99A |
| LOX | FOXO1 | BAK1 | HLA-DRB1 |
| CCNE1 | RARA | DHFR | PRKD1 |
| ETV6 | SPINK1 | HSPA4 | SLC9A9 |
| FOS | ENG | SPARC | KCNQ1OT1 |
| GATA2 | PLAUR | MT-ND4L | MUC2 |
| FOXM1 | DDR2 | S100A4 | DHFR |
| SERPINE1 | CDH13 | SKP2 | POU5F1 |
| BIRC3 | RAC1 | FOXO3 | CD34 |
| B2M | CCND2 | TRIM28 | MIR30A |
| SERPINA3 | AKT3 | LEP | DRAIC |
| PDPN | MITF | IDH2 | NME1 |
| LOC107303340 | CRAT37 | CA9 | NTRK1 |
| MVP | CCNE1 | SOS1 | TIMP1 |
| PTHLH | MIR152 | ZEB2 | CTSD |
| CDH2 | PROM1 | NTRK1 | FAN1 |
| NEAT1 | U2AF1 | PAK1 | DNMT3B |
| KRT20 | PRKCA | CFLAR | CCND3 |
| CYP19A1 | AREG | HLA-A | SPRY4-IT1 |
| MIR24-2 | TOP1 | TP63 | U2AF1 |
| MIR199A1 | CYP1B1 | HDAC9 | BMI1 |
| BAK1 | SERPINA3 | FOLH1 | ERCC5 |
| TIMP2 | COMT | EPHX1 | HDAC1 |
| RARA | CTSD | APEX1 | CCNB1 |
| NTRK1 | CCL5 | SERPINB5 | CHEK1 |
| MT-ND1 | INS | CCL5 | PRKCA |
| HDAC1 | HDAC1 | CCKBR | MIR181B1 |
| HNRNPUL2-BSCL2 | MMP3 | ANXA5 | INS |
| FZD3 | CD34 | NAT1 | NEAT1 |
| CYP2E1 | HSPA5 | AIP | ACTA2 |
| CHUK | EWSR1 | XPC | H2AC18 |
| TIMP3 | PSCA | SMARCE1 | TLR4 |
| GPC3 | HLA-B | DKK1 | ACTG2 |
| THBS1 | MT-CO2 | WNT5A | CHGA |
| GNG3 | PAX1 | CCND3 | NOS2 |
| IL2RA | MTUS1 | JAK1 | FLT4 |
| FAM13A | CFLAR | LOX | CREB1 |
| MIR510 | WRN | MMP3 | PAX8 |
| KRT8 | HMGA2 | NRP1 | FOXP3 |
| MUC5AC | TCF7L2 | MAX | AKT3 |
| CYP2D6 | DHFR | LGALS3 | CDH3 |
| DPP9 | NOTCH2 | HMGB1 | CTSB |
| EDNRA | CYP2E1 | KLF4 | SNHG16 |
| CEACAM3 | ANXA5 | IFI27 | MIR16-2 |
| SPARC | RECQL | IL17A | PROM1 |
| MT-CYB | HLA-DQA1 | BCL2L11 | FASN |
| CFLAR | NOS2 | KEAP1 | EREG |
| BMP2 | TLR9 | XIST | NOTCH3 |
| PRKD1 | BAK1 | MAP2K2 | ZFAS1 |
| HSPA5 | BDNF | TUG1 | SPARC |
| CTSB | XIST | MIR149 | MIR375 |
| CT83 | MINPP1 | MIR181B1 | FOLH1 |
| APEX1 | TNFRSF10A | MIR193A | TBX3 |
| SKP2 | KCNQ1OT1 | EPHA2 | NGF |
| EPHA2 | RPS6KB1 | PKM | ATP7A |
| ACTA2 | FOXO3 | HMGA2 | TOP1 |
| NRP1 | CDH2 | MIR9-1 | FGF3 |
| CLDN7 | HSPA4 | CYP2D6 | LOX |
| LOC109504725 | IL17A | MIR424 | APEX1 |
| GJA1 | CIZ1 | HFE | RAC1 |
| GAS5 | HLA-G | VEGFD | AFP |
| ZFHX3-AS1 | HDAC9 | MIR139 | EWSR1 |
| DHFR | GATA3 | NOTCH3 | CCND2 |
| ACE | IL7 | ICAM1 | SFRP1 |
| KLK3 | PTHLH | TCF4 | MIR320A |
| XPC | FBN1 | ALDH1A1 | FOXO1 |
| FOXO3 | SOS1 | CTNND1 | UPK1A |
| TH2LCRR | SDHAF2 | CYP1A2 | CHRM3 |
| LZTS1 | GPC3 | BAD | BMP6 |
| TUG1 | KRT5 | MAP2K4 | SMO |
| RPS6KB1 | HMGB1 | EZR | NUMA1 |
| AIP | TRPV4 | WNT1 | HSPA5 |
| MYCL | FANCI | MTA1 | FOXO3 |
| CREBBP | MIR149 | MIR16-2 | COMT |
| PRKAR1A | APEX1 | TPX2 | SF3B1 |
| U2AF1 | MIR193A | SHC1 | WRN |
| HLA-DQB1 | FLT3 | CYP17A1 | ICAM1 |
| KCNQ1OT1 | IFI27 | PDPN | LGALS3 |
| TGFB2 | ENPP1 | YBX1 | CYP2E1 |
| ATP11A | MAP2K2 | EXO1 | WNT3 |
| IRS1 | SERPINE1 | GAST | MIR181C |
| CYP3A4 | CD24 | ETS1 | IRS1 |
| NPM1 | LGALS3 | BSG | GSK3B |
| HSPA4 | ELAVL1 | H2AC18 | CAGE1 |
| FBN1 | SERPINB5 | BECN1 | TGFB2 |
| HLA-A | BSG | DAPK1 | CCL5 |
| RIPK1 | HNF1A | ANXA2 | RECQL4 |
| MIR521-1 | CCND3 | CASP7 | FOXM1 |
| EPHX1 | BECN1 | IFNA2 | AMACR |
| SCGB1A1 | MYLK | NPM1 | RXRA |
| ARAF | GAPDH | HLA-G | SDHAF2 |
| BID | SERPINB3 | SEC23B | CYP17A1 |
| RTEL1-TNFRSF6B | EZR | SFRP1 | RIPK1 |
| NSD1 | RECQL4 | MIR296 | MUC5AC |
| TNFRSF10A | CLDN7 | PTGS1 | MIR149 |
| MIR181A2 | FASN | ITGB3 | CYP3A4 |
| BMP4 | CDKN1C | ELAVL1 | EZR |
| DMBT1 | CLU | CLU | MIR152 |
| VEGFD | GPER1 | HULC | PRC1 |
| ANXA5 | LEF1 | GAPDH | ZEB2 |
| ITGA6 | SNHG16 | BID | RARA |
| MMP3 | MCM3 | FSCN1 | SKP2 |
| EWSR1 | MIR139 | PIK3R2 | CRNN |
| ELANE | CRNDE | MACC1 | ETS1 |
| MIR196B | BCAR1 | ODC1 | RPS6KB1 |
| ABCC2 | RUNX3 | SIRT1 | SERPINE1 |
| CCND3 | ATRX | TLR9 | PIK3CG |
| NCAM1 | PRKCD | MUC2 | MME |
| FASN | CASC9 | EGR1 | RUNX3 |
| BCL2L11 | COL2A1 | NDRG1 | ITGB4 |
| RUNX3 | KLF5 | MMP11 | CD24 |
| SERPINB5 | TRIP13 | BIRC3 | CALR |
| NOTCH3 | CTSB | AREL1 | LMOD1 |
| ENO1 | E2F3 | EPHA3 | MTUS1 |
| S100A4 | MIR296 | HPGD | CYP2D6 |
| ALOX5 | SPARC | PPARD | CD40 |
| EDN1 | RMRP | ITGAV | MIR196A1 |
| VIM | MUC5B | GRP | DKK1 |
| MIR181C | GJA1 | JUP | KIF20B |
| FENDRR | WNT5A | SPRY4-IT1 | PTPRC |
| CTAG2 | GDF15 | NOTCH2 | PDPN |
| WNT4 | NCAM1 | IGFBP2 | HSPA4 |
| E2F3 | PANDAR | ITGA5 | ABCC2 |
| HDAC9 | LHX3 | IFNA1 | TCF7L2 |
| VDR | EEF1A1 | GADD45A | GHET1 |
| GDF15 | EPHB4 | SHH | CASC9 |
| H2AC18 | ICAM1 | MTDH | GSN |
| MITF | TNFRSF1A | ENO1 | BDNF |
| HPGD | ZEB2 | PIK3CB | ERCC3 |
| TMEM127 | VEGFD | ID1 | KLF5 |
| MAP2K2 | CDC42 | RPS20 | MT-CO2 |
| ALDH1A1 | ERCC3 | MIR196A1 | SHH |
| ELN | GATA2 | CALR | CCR6 |
| ANGPT2 | ST20 | XRCC5 | WNT5A |
| CTNND1 | PCAT6 | CDC42 | SHC1 |
| IL1A | MYB | KITLG | MITF |
| BSG | LEP | CDH13 | GDF15 |
| DAPK1 | HOXA11-AS | PSCA | BMP2 |
| MAP3K6 | BCL2L11 | CLMAT3 | BID |
| PTPRC | APOE | RARB | NOTCH2 |
| IFI27 | LETMD1 | LGALS1 | MMP3 |
| AMER1 | NRP1 | ERCC5 | EGR1 |
| ZEB2 | LOX | MIR29B1 | HDAC9 |
| HMGA2 | TSG101 | HOTTIP | EPHB4 |
| EML4 | ETS1 | LINC-ROR | PTHLH |
| BCL6 | TG | MUC6 | GNRH1 |
| MIR9-1 | CYP3A4 | CCR7 | HSPD1 |
| GSTM3 | RUNX2 | CCNE1 | HYAL1 |
| CD4 | EPHA2 | MTR | BECN1 |
| CA9 | CYP2D6 | RNF6 | CYP1A2 |
| GADD45A | TFAP2A | ANGPT2 | PANDAR |
| CDH13 | ITGA6 | GPX1 | LEP |
| CCR7 | MUC5AC | WNT6 | BIRC3 |
| MIR152 | BID | ARAF | TRIM28 |
| MIR424 | YBX1 | PXN | BAK1 |
| CXCR2 | ALDH1A1 | FANCI | MIR424 |
| ALDOA | FOLH1 | CD82 | FLT3 |
| ITGAV | CCR7 | MMP13 | MINPP1 |
| SHC1 | MUC6 | ENO2 | IGFBP2 |
| CASP7 | MIR361 | LDHA | ID1 |
| BAD | FGF1 | SOX4 | IL17A |
| WRN | DES | ZFAS1 | IL1A |
| ERG | MIR31HG | NOD2 | HLA-A |
| CD36 | PKM | ANXA1 | HMGB1 |
| AXL | UBE3A | ST14 | ALDH1A1 |
| YBX1 | IRS1 | CSF3 | MIR31HG |
| LOC100507346 | CTNND1 | MIR181C | ATRX |
| TLR9 | CD28 | PIK3CG | GPER1 |
| LAMC2 | MIR181B2 | ERG | ELN |
| PTK2B | CBL | XRCC6 | GATA2 |
| ASCL1 | SERPINA1 | UICLM | EPHA2 |
| CDC42 | BCYRN1 | MIR125B1 | MIR29B1 |
| NOD2 | TRIM28 | MIR532 | ACP3 |
| CASC9 | CSF1 | CD34 | CCR7 |
| COL1A1 | RUBCNL | CXCR2 | KRT5 |
| MIR193A | S100A4 | ILK | BCL6 |
| CD40 | PAK1 | TFAP2A | MAP2K2 |
| MIR144 | MIF | MIR32 | GRP |
| ECT2 | ANXA2 | COMT | HMGA2 |
| MYO1B | CDH3 | NCAM1 | LEF1 |
| CEACAM6 | MUC4 | EREG | SQSTM1 |
| RECQL | CASP7 | RECK | LAMC2 |
| ID1 | PDPN | TMEFF2 | BCAR1 |
| GATA6 | SMO | PIK3CD | YBX1 |
| FSCN1 | STMN1 | NANOG | TNFRSF1A |
| POSTN | MTDH | SEMA4A | GPX1 |
| BPIFA1 | MTA1 | ADIPOQ | NCAM1 |
| XIST | XRCC5 | CAGE1 | FGF7 |
| TCIM | MMP13 | LRP6 | PCAT6 |
| BECN1 | FANCA | SERPINA1 | NRG1 |
| LEP | CDC6 | CYP24A1 | CXCR2 |
| EGR1 | RPS20 | JAG1 | MIR532 |
| TH2-LCR | SIRT1 | GRB2 | TRIP13 |
| ERCC5 | FSCN1 | U2AF1 | MYOCD |
| DKC1 | CD4 | S100A8 | CTNND1 |
| PCAT6 | RAD51B | TGFB3 | SST |
| GAPDH | IFNA1 | EPHB4 | IL7 |
| SDHAF2 | SNHG12 | YY1 | JAK1 |
| MIR30B | MIR186 | MIR29B2 | SOS1 |
| LGALS3 | BIRC3 | PIK3R3 | FSCN1 |
| PRKCD | ID1 | MIR152 | BCL2L11 |
| KRT5 | ITGB3 | PTTG1 | HLA-G |
| KMT2D | ITGAV | MTUS1 | KLF4 |
| MIR320A | FGF7 | HDAC2 | PAX2 |
| GSK3B | VANGL1 | SULT1A1 | VEGFD |
| COMT | KLF4 | STAT5A | TINCR |
| XRCC5 | SHBG | PTGER4 | MIR24-1 |
| TCF7L2 | CALR | CEACAM3 | BANCR |
| MIR181B1 | ABCC2 | DANCR | CASP7 |
| IKBKB | HBEGF | IL6R | FANCA |
| PRKCI | EGR1 | MIR144 | ILK |
| MYO18B | CDC25A | MITF | MIR26B |
| MUC4 | CD82 | RECQL | HLA-B |
| EPHB4 | MIR532 | CHGA | SOX4 |
| TXN | HAGLR | LASP1 | LIG4 |
| STMN1 | MIR125B1 | BCAR1 | ITGB3 |
| SPINK1 | CD40 | CDC25C | KDM4C |
| TRIM28 | ANXA1 | CD36 | SREBF1 |
| PIK3R2 | LRRK2 | WNT3A | FBN1 |
| MIR324 | GADD45A | CXCL1 | DKC1 |
| CDK12 | SHC1 | AMACR | PAK1 |
| MMP12 | IL1A | WIF1 | APOE |
| IL3 | JAK1 | CSNK2A1 | HAGLR |
| AURKB | NTRK3 | HMOX1 | NRP1 |
| MIR139 | MIR144 | IGFBP1 | BAD |
| MAP2K4 | KITLG | INSR | RAD51B |
| MIR149 | IFNA2 | TNFRSF1A | CLDN7 |
| MIR296 | GUSB | PSG2 | PTGS1 |
| WNT7B | ST14 | KDM1A | LGALS1 |
| AGER | SOX4 | FOXO1 | HNF1A |
| TKT | SHH | MIF | PKM |
| CCKBR | MACC1 | IRS2 | FLNA |
| RBFOX1 | ETV4 | CDKN1C | NORAD |
| CDKN2C | STAT5B | TEK | MIR296 |
| YY1 | CALB2 | CYTOR | FANCG |
| CCND2 | TP53COR1 | SOCS3 | MDM4 |
| HOTTIP | ENO1 | CRCS10 | XRCC5 |
| MYB | HMOX1 | HLA-B | VANGL1 |
| ERCC3 | LGALS1 | LAMC2 | GBE1 |
| E2F2 | MIR124-1 | PDGFB | WNT4 |
| CDC25A | EDNRA | UHRF1 | BRMS1 |
| PIK3CB | CT45A5 | WNT2 | CD4 |
| CD34 | TCF4 | MIR124-1 | RALA |
| MSLN | TUBB | CDX1 | MIR370 |
| LGALS1 | MFN2 | CLDN4 | LZTR1 |
| ILK | GATA1 | FADD | LCP1 |
| RRM1 | PTPRC | PTPN13 | HNF1A-AS1 |
| FCGR2A | HP | DIABLO | ENO1 |
| NCOA3 | ACTB | LRP5 | PIK3R2 |
| FAN1 | TNFSF11 | SDHAF2 | LMNA |
| JUP | LDHA | FLT3 | KITLG |
| PXN | EPHX1 | ITGA6 | ANXA1 |
| SLC11A1 | PTGS1 | H2AX | TPX2 |
| CCL11 | NANOG | PDCD4 | ELAVL1 |
| MMP13 | NDRG1 | ERCC3 | CTNNA2 |
| CYP17A1 | PLG | PTHLH | PAX5 |
| DLK1 | MIR29B1 | MIR92A1 | PRKCD |
| SMO | ALDOA | CASR | CASP2 |
| CALR | ZNF268 | IL4R | STAT5B |
| TFAP2A | SQSTM1 | CEACAM6 | IFNA1 |
| INS | CFTR | MINPP1 | ANXA2 |
| INS-IGF2 | CBFB | SNHG16 | CCNH |
| NAPSA | SST | CSNK1A1 | GREB1 |
| FOXO1 | TPX2 | HNF1A | NSD1 |
| EIF4E | FANCG | SYP | KRT14 |
| MIR16-2 | MPL | SERPINA3 | SLC35E3 |
| XRCC6 | WNT1 | TKT | CADM1 |
| GACAT2 | PLK2 | PRKDC | ETV4 |
| LINC-ROR | XRCC6 | CASC9 | MTDH |
| NTRK3 | NNT-AS1 | E2F4 | S100A8 |
| CTSD | ING3 | KDM4B | CDC25A |
| TUBB | IL3 | TXN | MTA1 |
| ITGA5 | MIR29B2 | SNHG1 | MIR32 |
| PCAT7 | MIR99A | ITGB4 | EBAG9 |
| APAF1 | PAX3 | LGR5 | COL1A2 |
| GRPR | SEC23B | MAP2K5 | MUC6 |
| MAGEA3 | IGFBP1 | SOCS1 | IL2RA |
| UGT1A1 | P2RX7 | MIR24-1 | ADH1C |
| IL4R | TCIM | AFAP1-AS1 | XRCC6 |
| COL18A1 | MDM4 | TRIP13 | FOXA1 |
| PLCG1 | RECK | DVL1 | EPHX1 |
| STAT5B | MSLN | ALCAM | SLC45A3 |
| PPP2R1A | MIR324 | HAVCR2 | PCAT7 |
| DUSP1 | CSF1R | GATA2 | LCN2 |
| BMI1 | IGFBP2 | CEACAM7 | HMOX1 |
| MINPP1 | THPO | HSPA8 | JAG1 |
| WIF1 | ANGPT2 | CREBBP | CBL |
| ITGA2 | CEACAM3 | TCF7 | SULT1A1 |
| MTUS1 | ACAN | IL7 | MIR103A1 |
| DIABLO | STAT5A | BBC3 | MTR |
| BIRC7 | HDAC4 | RARA | COL18A1 |
| FLT3 | JAG1 | KLRK1 | FANCI |
| RECK | EIF4E | CD4 | ZEB2-AS1 |
| MIR196A1 | ZFAS1 | WNT2B | CSF1 |
| SST | GRP | ALOX12 | DEPDC1 |
| HSD17B1 | PXN | RECQL4 | CCKBR |
| MAGEA4 | PIK3CD | MIR338 | TG |
| TCERG1 | PAX5 | EWSR1 | HIF1A-AS2 |
| CD8A | FUZ | MIR497 | YY1 |
| MIR29B1 | CALCA | ITGA2 | LAMB3 |
| KITLG | LIG4 | SPHK1 | SOCS3 |
| IL7 | FGF8 | MAP2K3 | LINC-ROR |
| WNT3 | PIK3R2 | PHLPP1 | TAC1 |
| ANGPT1 | SF3B1 | SLC19A1 | UGT1A1 |
| ETS1 | CD36 | PANDAR | CDH13 |
| ADRB2 | JUP | PTENP1 | GPC3 |
| WNT5A | MAP2K4 | CLDN3 | TOE1 |
| MIR125B1 | LOC110806263 | IL13 | NOS3 |
| BIRC2 | MIR32 | FZD7 | LIMK1 |
| RBM6 | MVP | SFRP2 | CDC25C |
| SLC9A9 | CDC25C | ATRX | STMN1 |
| ANXA1 | MC1R | GPER1 | SIRT1 |
| NOS3 | LAMC2 | ATF3 | GLIPR1 |
| PIK3R3 | EXT2 | HLA-DQB1 | GADD45A |
| CDC25C | DANCR | LMNA | CTAG1A |
| CBL | UGT1A1 | WNT3 | MVP |
| CXCL10 | KLK10 | PRKCD | PPARA |
| ITGB3 | MIR101-1 | CASP2 | HSD17B1 |
| MAGEA1 | FGF4 | DSP | MUC4 |
| SPRY4-IT1 | MIR24-1 | HNF4A | MIR125B1 |
| FOXA1 | NSD1 | TCF3 | TYR |
| TFF1 | CXCR2 | SUZ12 | IL3 |
| CDX2 | MIR338 | WNT10B | NTRK3 |
| CALCA | MCM2 | E2F3 | ANGPT2 |
| NCOR1 | SIX1 | PECAM1 | CXCL1 |
| LMNA | SETBP1 | NTRK3 | MMP13 |
| MIR32 | MIR30B | REG4 | MAGEA3 |
| FGF1 | APAF1 | MIR27B | CALCA |
| KDM4C | TBX1 | HNRNPK | GRN |
| RMRP | CDKN2C | PCAT6 | CRNDE |
| EXO1 | AHR | FOSL1 | HOTTIP |
| SOX4 | PIK3R3 | SMO | DUXAP9 |
| MIAT | KRT17 | NRG1 | B2M |
| SNHG1 | CHUK | MELK | HPGD |
| RHOB | GPX1 | PLG | ROBO1 |
| ANXA2 | FLNA | ETV4 | MUC7 |
| PAX8 | MAGEA3 | MIR132 | PXN |
| BDNF | PRKACA | LIG4 | TACC1 |
| DANCR | HSPD1 | HOXA11-AS | CDK12 |
| NOTCH2 | AURKB | CTNNA2 | IDO1 |
| EZR | MYOD1 | FANCG | PRKACA |
| MME | BRMS1 | PRMT1 | MIR490 |
| AFP | BMPR2 | TRAF6 | AHR |
| INPP5E | MTR | NOTCH4 | SHBG |
| IL18 | HPGD | BCYRN1 | NDRG1 |
| MT-CO2 | BAG1 | LYVE1 | WNT1 |
| ROBO1 | PRL | NRP2 | PSAP |
| CD28 | PHOX2B | TCF7L1 | H2AX |
| HSPA8 | ILK | CYP2C9 | APAF1 |
| HOPX | S100A8 | HK2 | EFEMP2 |
| GCLC | GNRH1 | SOX2-OT | LDHA |
| YWHAE | BIRC2 | GJA1 | FENDRR |
| TET2 | TNFRSF1B | BNIP3 | TSG101 |
| MIR99A | CCNG1 | MAPK9 | RHOB |
| CXCL1 | HDAC2 | FAP | TGFB3 |
| AFAP1-AS1 | NOS3 | BANCR | ELF3 |
| AHR | EED | E2F2 | RECK |
| TGFB3 | B2M | ACTB | IL13 |
| RXRA | AGR3 | NES | CD36 |
| PDCD4 | PML | GRPR | SERPINA1 |
| TLR5 | ECT2 | TMPRSS4 | MIF |
| JAK1 | FOXA1 | NTN1 | FBLN5 |
| LAMB3 | TEK | RRM2 | ZFYVE26 |
| ROCK1 | ODC1 | CBL | PLK2 |
| CSNK1A1 | PRLR | GREB1 | FGF8 |
| RACK1 | AXL | DACOR1 | CYP24A1 |
| BCAR1 | PTENP1 | MIR181B2 | FADD |
| DKK1 | ITGA5 | SAT1 | STAT6 |
| CYP1A2 | UHRF1 | TIAM1 | TRPV1 |
| KLF4 | FADD | TP53COR1 | GAST |
| PTGER4 | NCOR1 | AGER | RPS20 |
| HLA-DQA1 | HMGA1 | MCM7 | DEK |
| NFKB2 | MIR132 | FZD8 | SOX17 |
| JAG1 | TXN | FZD4 | CCN2 |
| IL6R | SUZ12 | ALDOA | HPSE |
| TUSC2 | CYP1A2 | WNT11 | RNY1 |
| TIMELESS | NGF | CD8A | RNY3 |
| LINC00511 | KDM1A | MIR378A | ODC1 |
| GPRC5A | MMP11 | SERPINB2 | PECAM1 |
| TPX2 | EREG | GHET1 | HNRNPK |
| CAGE1 | PIK3CB | MMP12 | DIABLO |
| CD9 | CXCL1 | TJP1 | SNHG5 |
| TOE1 | HSD17B1 | UMPS | NANOG |
| CCNE2 | ZNRD1ASP | VTN | ITGAV |
| ZFAS1 | MAGEA4 | LCP1 | ICOSLG |
| SYK | FGF3 | UCHL1 | MYH9 |
| TNFRSF1B | POU4F1 | PMAIP1 | ZEB1-AS1 |
| MIR338 | IL18 | MUC4 | RELB |
| CSF1 | TNFRSF11A | NUDT1 | TNFSF11 |
| CCL3 | EIF4EBP1 | HMGCR | FANCF |
| PDGFA | DIABLO | TFF3 | MAGEA1 |
| TRAF6 | DKC1 | CDH3 | MAGEA4 |
| CLU | FGF9 | F3 | SEC23B |
| SEZ6L2 | SOCS3 | MIR31HG | E2F2 |
| COL4A3 | BCL6 | FES | COL7A1 |
| TNFRSF1A | BMP7 | LGALS3BP | AXL |
| BAG1 | SNHG1 | ICOSLG | MAP2K4 |
| DDR1 | TKT | PRKACA | NOD2 |
| SKP1 | MAGEA1 | MUC12 | UHRF1 |
| HDAC2 | ELANE | DPP4 | CFTR |
| SIRT1 | MIR92A1 | WNT10A | PTENP1 |
| SSTR2 | MYOG | MIR133A1 | DPH1 |
| MIR124-1 | IL4R | CYP27B1 | MIR30B |
| MIR29B2 | CT45A1 | SLC5A8 | SNHG1 |
| HAVCR2 | VANGL2 | WNT4 | FBLN1 |
| GAST | CD8A | S100A9 | MYB |
| TG | PLCG1 | CDH17 | TEK |
| PEBP1 | FANCF | MDM4 | CSF1R |
| RNF6 | DVL1 | LNCRNA-ATB | PIK3CB |
| IL24 | WNT6 | SATB2 | PRL |
| PAX5 | MIR423 | TLR3 | IGFBP1 |
| RXRB | FANCL | MTHFD1 | ACE |
| CHI3L1 | KDM4C | DDIT3 | STAT5A |
| SOX2-OT | IL6R | ADH1C | TKT |
| IGFBP2 | KEAP1 | CSF1 | GNAQ |
| NR3C1 | GAST | SYNE1 | ITGA5 |
| FLNA | HAVCR2 | TG | GRB2 |
| NODAL | POLH | MAD2L1 | RUNX2 |
| IL5 | POSTN | STUB1 | CDKN2C |
| BTNL2 | CDK12 | PPARA | CD28 |
| S100A2 | KMT2D | CCL20 | EDN1 |
| BTK | KLRK1 | MYB | CGB3 |
| EPB41L3 | PRMT7 | NORAD | MACC1 |
| PECAM1 | EXO1 | NTS | PLG |
| APOE | PTTG1 | PHLPP2 | S100A6 |
| ABCC3 | SFRP1 | CBFB | IL6R |
| PSCA | SSX2 | DLL4 | PML |
| SNHG16 | GLI2 | TDGF1 | NCOR1 |
| BCR | MIAT | SST | CD9 |
| ETV4 | MYD88 | PTP4A1 | AURKB |
| RYR1 | CEBPA | CDK8 | DANCR |
| RIOX2 | NAT1 | CBR3-AS1 | KMT2D |
| CRNN | PAWR | EBAG9 | EDNRA |
| DRAIC | CEACAM7 | PROX1 | SPHK1 |
| UCHL1 | IDO1 | CLDN1 | DUSP1 |
| EPAS1 | COL18A1 | PTP4A3 | ASXL1 |
| LEF1 | SULF1 | TINCR | MUC5B |
| PIK3CD | MUC2 | WNT7B | POSTN |
| RECQL4 | THAP1 | CDCP1 | MIR29B2 |
| RCVRN | GALNS | FGF1 | TMPRSS2 |
| SLC19A1 | IL13 | MAPK12 | LINC00958 |
| GATA4 | PTK2B | TUSC7 | TCIM |
| MIR24-1 | HPSE | CEACAM1 | EIF4E |
| TMPRSS2 | CCN2 | HBEGF | POLH |
| CEBPA | ADH1C | DVL2 | AKR1C3 |
| MYLK | IKBKB | LINC00858 | XBP1 |
| DCK | E2F2 | HLTF | DAB2IP |
| RRM2 | MIR27B | TLR5 | S100A7 |
| F2 | TOR1A | PLK2 | KDM5B |
| SCUBE3 | GNAL | STAT5B | NCOR2 |
| ACTC1 | INSR | USF3 | MED12 |
| POLR1C | ANO3 | BDNF | MIR423 |
| JAK3 | TMPRSS2 | AHR | SSTR2 |
| TNFRSF10D | CEBPB | FBN1 | MIR186 |
| FOXJ1 | GSTM3 | RAD51B | LRP6 |
| ICOSLG | RPS19 | CCR5 | PTK2B |
| IFNA1 | H2AX | HNF1A-AS1 | ZNF224 |
| FGF8 | PEBP1 | IL15 | BIRC2 |
| MIR27B | FOXC2 | WNT5B | TCF4 |
| GPER1 | NOD2 | GPC3 | SFTA3 |
| MIR497 | NFKB2 | SEPTIN9 | CDC42 |
| MIR101-1 | CCNE2 | SNHG12 | PHOX2B |
| WEE1 | FLI1 | PAX8 | BCR |
| BRDT | REST | COL4A1 | TMEFF2 |
| TYR | ITGB4 | APOE | HSPA8 |
| MYD88 | EPHA3 | TNFRSF10D | TET2 |
| PTPN13 | MIR497 | TET2 | MIR330 |
| ATRX | RNY1 | STMN1 | ALDOA |
| KLF5 | RNY3 | RALA | EIF4EBP1 |
| DYNC2H1 | LRP5 | WNT8B | PRKDC |
| SREBF1 | RELB | MTAP | PMF1 |
| MTDH | NES | FOXC2 | RTEL1 |
| MIR31HG | TUSC3 | KRT5 | ERCC8 |
| REST | RPL5 | SELE | ETV1 |
| MPL | NPTN-IT1 | USP28 | VWF |
| MAGEC2 | GRB2 | MSLN | TFAP2A |
| CEACAM7 | WIF1 | AXL | DDR2 |
| SEMA3B | PECAM1 | HLA-DRB1 | MSLN |
| TMEM71 | PPP2R1A | DELEC1 | HDAC2 |
| PLG | CSNK2A1 | CCN4 | MIR324 |
| SAT1 | SERPINB4 | FZD6 | IL4R |
| GATA3 | SLC26A2 | UGT1A7 | RASA1 |
| CXCR1 | PDCD4 | COL1A1 | ARAF |
| PRKAA1 | FABP4 | SF3B1 | PLCG1 |
| BCYRN1 | CASP2 | COL4A3 | KEAP1 |
| HLA-DPB1 | TMPOP2 | MIR211 | KAT5 |
| SETBP1 | PRKDC | CD80 | NFKB2 |
| PRTN3 | GRN | CADM1 | MIR133A1 |
| LIMD1 | COL11A1 | CHI3L1 | KLK10 |
| ELAVL1 | PAX7 | KLK10 | DES |
| LIG4 | FOSL1 | SQSTM1 | TRPV4 |
| LAMA5 | CYTOR | TACC1 | INSR |
| PRKDC | AGER | HPRT1 | ST14 |
| RAD51B | SOX2-OT | EIF4EBP1 | CCNG1 |
| TINF2 | MIR330 | CSE1L | FOXD2-AS1 |
| CALB2 | CIP2A | MAP2K6 | TLR9 |
| TK1 | YWHAE | NOS3 | PCAT14 |
| PPP2R2A | PBRM1 | MIR130B | ANG |
| SLPI | TIMELESS | CD28 | DELEC1 |
| STK4 | EXTL3 | GATA3 | RAB27A |
| MTA1 | LCN2 | ECT2 | WIF1 |
| HOXA11-AS | CCNA1 | MIR99A | MTAP |
| PKM | ZNF667-AS1 | MIR328 | TUBB |
| FANCA | CYP24A1 | MIR345 | ALOX5 |
| S100A8 | CHST3 | SHBG | ACTN4 |
| SPAG9 | MCM7 | EIF3H | DIRC3 |
| AKR1B10 | MIR377 | FAM98A | RYR1 |
| PML | RHOB | EIF5A2 | PCSEAT |
| MIR377 | HSPA8 | MAGEA3 | LOC110806263 |
| LAMC1 | KMT2A | AGR2 | NKX3-1 |
| GLI3 | SYK | DKC1 | AGR3 |
| THY1 | RRM2 | MC1R | PTTG1 |
| IREB2 | STS | FZD1 | SEMA4A |
| NDRG1 | HK2 | GZMB | IGFBP5 |
| STAT6 | PIWIL4 | TYR | HPRT1 |
| TNFSF11 | CCR5 | NSD1 | APC2 |
| SOCS3 | CT45A8 | FGF7 | KDM1A |
| COL4A2 | RYR1 | CSNK1E | HLA-DQA1 |
| IL15 | NDUFA13 | EED | PCAT5 |
| CTSL | SYNE1 | MIR675 | MIR129-1 |
| MIR423 | IGFBP5 | TCIM | RPL11 |
| PAK1 | PIN1 | NNT-AS1 | CEACAM3 |
| MBL2 | CD9 | PAX5 | SLC19A1 |
| HBEGF | AMACR | TSG101 | TXN |
| HNF1A-AS1 | PTPN3 | OLFM4 | GNA11 |
| F3 | MCM4 | STRAP | ITGA3 |
| RHBDF2 | PTGER4 | CYP2C19 | MIR211 |
| ANPEP | IGF2BP3 | PLK4 | TNFRSF1B |
| PTGS1 | CEACAM6 | ADAM17 | CSNK1A1 |
| CXCR3 | MIR574 | WNT7A | SETBP1 |
| GLI2 | EDN1 | ETV1 | EXO1 |
| FLI1 | AMPH | GNRH1 | NNMT |
| MDM4 | BTK | MAGEA1 | UPK1B |
| MCM3 | KLK6 | IL3 | PRMT7 |
| LCN2 | DSP | IL2RA | KMT2A |
| MED19 | XBP1 | MME | MIR27B |
| PHOX2B | NR1H2 | CLDN18 | GHR |
| NNMT | ACTA2 | CACNA1G | MIR497 |
| GPX1 | PTCH2 | TPBG | LASP1 |
| TTK | LRP6 | VCAM1 | WNT6 |
| RUNX2 | MIR212 | CD86 | ACTB |
| PRDM14 | TGM2 | CDC25A | PEBP1 |
| EXT2 | LINP1 | FOXA1 | MIR124-1 |
| EIF2AK2 | ICOSLG | DLL1 | HNRNPA2B1 |
| CBFB | ATAD2 | SLC16A7 | CCN1 |
| TCF4 | LZTR1 | FANCA | HAVCR2 |
| TEK | SFTPA1 | SNHG5 | ABHD11-AS1 |
| CCR3 | DAB2IP | SNHG20 | VIP |
| RPSA | MIR370 | HOXA-AS2 | MAP3K7 |
| LRP1B | GRPR | FOXQ1 | RAD52 |
| TCF7 | SF3B2 | GUCY2C | MMP11 |
| RASA1 | PAX8-AS1 | CDK12 | CXCL10 |
| ADAM12 | ELK1 | MBD4 | P2RX3 |
| PLK2 | S100A2 | CTBP1 | BRD4 |
| FANCG | GHR | FCGR2A | LRP5 |
| ADIPOQ | DPH1 | EIF4E | ATF3 |
| PANDAR | TFRC | CSK | F2R |
| CD79A | CD247 | PHOX2B | FGF10 |
| IGFBP5 | SLC45A3 | IL1A | FABP4 |
| CP | GHET1 | AURKB | MIR101-1 |
| GNAQ | SPHK1 | B2M | MIR675 |
| POMC | NOTCH4 | HMGA1 | CALB2 |
| FABP4 | NTRK2 | LINC01133 | CASR |
| SNHG15 | LTA | UGT1A9 | ALPP |
| GSN | PRSS1 | PPARGC1A | AFAP1-AS1 |
| IL6ST | ALOX5 | NCOR1 | PBRM1 |
| BANCR | RPL15 | SETDB1 | NGFR |
| HNF1A | ITGA2 | WNT9A | RPS6KA5 |
| MIR361 | COL7A1 | FCGR3A | UCHL1 |
| HNRNPA2B1 | S100A9 | FAM98B | NR1H2 |
| HAGLR | PSG2 | SOX17 | ING3 |
| THPO | EPOR | LATS2 | MYD88 |
| CSF1R | VIP | RICTOR | MT-ND4L |
| SOD1 | RAD52 | LINC00261 | PDCD4 |
| MCM4 | SALL4 | DUXAP9 | HMGA1 |
| SMAD6 | KDM5B | MIR30B | FZD8 |
| ADA | DMBT1 | BRMS1 | CLDN4 |
| MTAP | TLR3 | CCNG1 | PRLR |
| LINC00673 | CD40LG | MIR135B | MMP12 |
| GNRH1 | CCN1 | TMPRSS2 | TLNRD1 |
| SNHG12 | PPARA | CHUK | CSNK2A1 |
| CD82 | RRAS | TUSC3 | CIP2A |
| EPHA3 | ANGPT1 | UGT1A6 | MIR132 |
| DDX5 | NR3C1 | PML | SPG7 |
| ACVRL1 | CLDN4 | ALOX15 | S100A9 |
| TRAF2 | CLPTM1L | CDKN2C | RBX1 |
| CCNA1 | STAT6 | PTK2B | AGR2 |
| MIR138-1 | ACVR1 | NTPCR | GATA1 |
| MIR212 | ITGA3 | CD40 | MNX1 |
| SMPD1 | FOXC1 | JAG2 | FUS |
| ETV1 | GLB1 | KDM4C | ALCAM |
| DMTF1 | LYVE1 | WNT16 | ACER3 |
| SIRT3 | CUL1 | COX5A | LINC00312 |
| WNT1 | NTN1 | TBK1 | BTK |
| CLPTM1L | RRM1 | CXCL5 | IRS2 |
| MIR330 | SSTR2 | LCK | PAWR |
| SNHG7 | ATF3 | ABCC3 | CHUK |
| CDCP1 | BRD4 | HSP90B1 | POMC |
| RXRG | E2F4 | MVP | SPG11 |
| CCR5 | DCK | CSF1R | LIFR |
| STAT5A | BCR | PLCG1 | MIR92A1 |
| HP | COL3A1 | APAF1 | GRPR |
| TSG101 | FCGR2A | PAWR | FLI1 |
| F2RL3 | HLA-C | PTGER2 | LEPR |
| CHRNB4 | PTK6 | POLH | SIX1 |
| EBAG9 | MAP2K3 | PTPRC | PPP2R1A |
| NTRK2 | F3 | CLDN23 | WEE1 |
| CASP1 | IL24 | ALOX5 | CLPTM1L |
| CCNH | MIR503 | MIR103A1 | BBC3 |
| MIR133A1 | MIR378A | HNRNPA1 | GNB1 |
| ENPP2 | LINC01133 | AGR3 | SPAST |
| HOXB9 | MVK | SETBP1 | MIR326 |
| TP53COR1 | CDH11 | IGFBP7 | CDH5 |
| FADD | POU2F3 | MIRLET7F1 | HOXA13 |
| DUS2 | ROCK1 | RAN | TJP1 |
| SERPINB3 | COL1A2 | SFRP4 | ITGA2 |
| AIMP2 | CAT | TFDP1 | KRT13 |
| CAVIN3 | CSNK1A1 | DMBT1 | SFTPA1 |
| EPHA5 | SRA1 | TNFRSF1B | ROCK1 |
| AREL1 | SLC19A1 | MAPRE1 | FGF4 |
| SQSTM1 | WFDC2 | MAGEA4 | ETS2 |
| SIX1 | WNT11 | PRLR | HMGCR |
| PDK1 | WNT4 | PRL | RMRP |
| IFNA2 | SLC5A8 | PRSS1 | BCYRN1 |
| TLCD3A | MIR455 | ID2 | WNT11 |
| SSX2 | ROBO1 | FZD10 | MPL |
| PRKACA | NT5E | DDR2 | SOX2-OT |
| HSF1 | LAMB3 | MIR135A1 | COL3A1 |
| SNHG20 | BBC3 | LGALS4 | BMPR2 |
| NOVA1 | NGFR | CCN2 | TFF3 |
| ACTB | LIN28B | BCL6 | EXT2 |
| CDH5 | SOCS1 | MMP15 | ARID4B |
| GHET1 | EPAS1 | DCLK1 | SYNPO2 |
| HULC | AGR2 | DVL3 | KISS1 |
| WNT2 | PRKCI | FUT4 | MIR135A1 |
| LNCRNA-ATB | EYA1 | SHMT1 | EPAS1 |
| RPL5 | UBE2T | NLRP3 | PRSS1 |
| TLR3 | SLPI | HSD17B1 | PIK3CD |
| RAD52 | BNIP3 | S100A6 | PSG2 |
| THBD | MMP12 | LINC00472 | GSTM3 |
| CXCR5 | SULT1A1 | ZEB1-AS1 | HNRNPA1 |
| RBM5 | TCF7L1 | CD276 | SALL4 |
| HDGF | CTCF | TLR7 | CD80 |
| TRAF1 | EMD | MIR324 | FOXC1 |
| TINCR | MBL2 | RUNX2 | HP |
| SF3B1 | WNT2 | GPX2 | WFS1 |
| ASXL1 | NKX3-1 | SELENBP1 | RHEB |
| CTNNA2 | FAP | FEZF1-AS1 | NR3C1 |
| EPHA7 | WNT3A | MNX1-AS1 | APOA1 |
| BRMS1 | EPO | HLA-DQA1 | MIR574 |
| SCGB3A2 | ASXL1 | TAGLN | COL4A1 |
| MIR186 | ALCAM | CTBP2 | BIRC7 |
| AGR3 | RPL11 | POSTN | SDC1 |
| VCAM1 | TMC8 | BTRC | PFAS |
| RARS1 | GREB1L | FUT3 | STS |
| CEBPB | ALPP | TWIST2 | HOXA11-AS |
| MIR132 | CXCL14 | ZFP36L1 | HDAC4 |
| SOCS1 | CD80 | LOC110806263 | IL18 |
| MIR17HG | OXT | BIRC2 | MDC1-AS1 |
| NPC1 | DCN | APC2 | MIR338 |
| NAT1 | TUSC8 | NLK | CYTOR |
| CTCF | ADA | ING3 | MAGEC2 |
| ODC1 | BCAS1 | GSDME | SELENBP1 |
| TGIF1 | LIMK1 | PEBP1 | CCK |
| CYTOR | LNCRNA-ATB | NFKB2 | S100A2 |
| LTA | CXCL10 | GUSB | LTBP4 |
| EREG | TP53BP1 | MPL | TUSC3 |
| IL11 | KISS1 | CTNNBIP1 | MIAT |
| MIR218-1 | POMC | EPDR1 | EPHA3 |
| LCP1 | TRIM24 | TUBB | MIR452 |
| NR1H2 | OTX2 | SLIT2 | TRPM8 |
| F2R | PDPK1 | PBK | FUZ |
| RELB | CEMIP | AGO2 | CAT |
| PRKCB | MLANA | GATA1 | MIR1247 |
| TACC1 | RAD21 | GNAQ | CDH11 |
| KCNN4 | ACTN4 | MIR542 | TBXT |
| LAMA1 | DUSP1 | KRT14 | CGB7 |
| TRAF4 | UCHL1 | IKBKB | S100B |
| MIR181B2 | SSX1 | FPGS | SRA1 |
| FOLH1 | SERPINB2 | SNHG6 | POLR1C |
| TFRC | TNFRSF10D | NR1H2 | GPR68 |
| TFPI2 | WNT10B | CLDN2 | FRGCA |
| CASP2 | DDR1 | SSX2 | STUB1 |
| GADD45G | RPS27 | RELB | ATL1 |
| PPP6C | HNF4A | TLR1 | TRAF7 |
| MIR455 | EIF2AK2 | CD55 | CHD7 |
| MACC1 | ARL11 | MYD88 | CD40LG |
| CAT | BUB3 | ARRB1 | GNRHR |
| AKR1C1 | HPN | THPO | TTR |
| SPDEF | ROR2 | DKK2 | ABCC3 |
| KRT14 | TCF7 | LEPR | CYP4B1 |
| FGA | EFNA1 | CT45A1 | FOSL1 |
| SLC6A14 | ARSB | RNY1 | TFRC |
| PTTG1 | F2 | RNY3 | CDC6 |
| MIR574 | CLCA2 | DKK4 | PLEC |
| TRAF3 | F2R | FMNL2 | SYK |
| LUADT1 | POLR1C | BRINP1 | CEBPB |
| TRPV4 | IBSP | RHO | POLG |
| SFRP1 | BIRC7 | LCN2 | UBE2T |
| SEMA3F | MIR2861 | CUL1 | DSP |
| PRL | CASR | PRKCE | EIF3H |
| CDK5 | MIR133A1 | HNRNPU | SF3B2 |
| LDHA | S100B | H4-16 | SPINT2 |
| DLL1 | CCDC7 | HLA-C | CXCR1 |
| GADD45B | CDK5 | DAB2IP | LYVE1 |
| IST1 | INHA | STAT6 | TRIM24 |
| USP8 | STK4 | CLDN8 | CD8A |
| GREB1 | DDIT3 | EDNRA | SUMO1P3 |
| S100A9 | MIR138-1 | CFTR | ZNF154 |
| CTAG1A | IDUA | PPP2R1A | DMBT1 |
| DPYSL5 | TRAF6 | GNB1 | TP53COR1 |
| ZMYND10 | CIB1 | SELP | ALOX12 |
| CCN4 | AKR1C3 | ZFP36L2 | CCEPR |
| NMBR | NCOA1 | WDCP | MST1R |
| POLH | LGR5 | RUVBL1 | PTPN3 |
| LY6K |  | MIR370 | DACOR1 |
| COL3A1 |  | CLCA2 | IGF2BP3 |
| MIR503 |  | RBPJ | NES |
| ARG1 |  | SYK | ADA |
| TLE1 |  | LINC00460 | SPDEF |
| KDM4B |  | CD9 | MYL9 |
| SOX17 |  | NTRK2 | MCM7 |
| ZNRD1ASP |  | STS | REST |
| TDGF1 |  | BMPR2 | CCNE2 |
| PRDX1 |  | CEMIP | IL15 |
| ELAVL4 |  | HPSE | GRB7 |
| LINC01133 |  | CHD8 | EPIST |
| HBB |  | FGF3 | MAGED2 |
| NANOG |  | HDAC4 | PIK3R3 |
| DES |  | FER1L4 | KLRK1 |
| MIR124-3 |  | RTEL1 | CUL1 |
| WNT3A |  | CEBPB | THPO |
| CD40LG |  | MIR423 | SUZ12 |
| CUL1 |  | DES | SOCS1 |
| INSR |  | GRN | MIR377 |
| MIR345 |  | PTBP1 | IKBKB |
| IL1R1 |  | BAG1 | SERPINB2 |
| AGT |  | PBRM1 | ELK1 |
| HJURP |  | GSTM3 | SLC7A5 |
| ADH1C |  | ANTXR1 | NTRK2 |
| PTPRG |  | FANCF | ADRB3 |
| GBA |  | REST | TCF3 |
| CACNA2D2 |  | FGF8 | LSP1 |
| CCL4 |  | FGF4 | NCOA1 |
| MIR532 |  | ATAD2 | PIM1 |
| MIR216A |  | NUMB | IL6ST |
| PKD1 |  | TMEM238L | BMP7 |
| IDO1 |  | MIR95 | TCHP |
| RPS20 |  | ACP3 | PTCH2 |
| ING3 |  | SAPCD2 | ZMYND8 |
| RASSF5 |  | SSTR2 | DBH |
| TBX5 |  | CLCA1 | TAGLN |
| MYOD1 |  | MAP3K7 | KLK4 |
| SELENBP1 |  | TGM2 | WNT3A |
| AGTR1 |  | TRIM24 | IL7R |
| CLCA2 |  | KISS1 | NCOA2 |
| SLC26A9 |  | XBP1 | KDM4B |
| MIR30C1 |  | POLE2 | HSF1 |
| LINC00261 |  | SMAD6 | BNC2 |
| PRKCE |  | EDN1 | MECP2 |
| ITGB4 |  | EPAS1 | GJB2 |
| NMB |  | MIR101-1 | CP |
| MC1R |  | RMRP | ANGPT1 |
| HDAC4 |  | PTPN3 | WNT2B |
| STRA6 |  | MIR339 | MIR28 |
| MST1R |  | GRK2 | FANCL |
| TP73-AS1 |  | LLGL1 | TNFRSF10D |
| NCOR2 |  | ITGA3 | SOD1 |
| CDH3 |  | MAGEC2 | F2 |
| FARSA |  | PIK3R4 | WNT10B |
| AOC3 |  | PALS1 | ACTC1 |
| SAA1 |  | ATF1 | BAG1 |
| CDA |  | MST1R | LTA |
| GRB2 |  | RMI1 | ADIPOQ |
| CEP57 |  | SLC45A3 | PIN1 |
| RIOX1 |  | CALCA | CAPN1 |
| MIR129-1 |  | DEAF1 | HK2 |
| SHBG |  | TIMELESS | SMARCA2 |
| PLAG1 |  | VWF | CEBPA |
| PIP |  | HIF1A-AS1 | RPL5 |
| WNT2B |  | WNT9B | DCK |
| PTPN3 |  | IGF2BP3 | PLP1 |
| NLRP3 |  | CIP2A | XAGE1B |
| FANCI |  | FAT4 | SFN |
| GPR68 |  | BTK | PGM5-AS1 |
| CLDN4 |  | ZNF148 | JAK3 |
| ITGA2B |  | NPTN-IT1 | NCOA4 |
| TUT1 |  | CCN1 | TIMELESS |
| VWF |  | CASP5 | TGIF1 |
| BUB3 |  | S100A11 | RHOC |
| MIR4435-2HG |  | GCNT3 | SLX4 |
| MIR211 |  | MIR625 | MCM2 |
| C20orf85 |  | PKP1 | TLR5 |
| ABCA1 |  | BCL9 | TTK |
| FANCF |  | NCOR2 | RRM1 |
| CD19 |  | KDM5B | PPP2R2A |
| CCL20 |  | ACTA2 | SEPTIN9 |
| CYP2A13 |  | ST6GAL1 | SNCA |
| SCNN1A |  | NR3C1 | BGLAP |
| EIF4EBP1 |  | RINT1 | PPARD |
| TP53BP2 |  | ARL11 | CTCF |
| CSNK2A1 |  | MIR377 | CD164 |
| TUSC1 |  | MIAT | MAPK7 |
| ADAM15 |  | NDUFA13 | P2RX7 |
| ACY1 |  | CDH5 | LINC00355 |
| BMP7 |  | ROBO1 | CBR3-AS1 |
| SULT1A1 |  | ACVR2A | MAP2K5 |
| GHR |  | OLA1 | MAP2K3 |
| RALBP1 |  | RHOB | FZD7 |
| KDM1A |  | KLK4 | RRM2 |
| MUC6 |  | RAD52 | TRPS1 |
| LAMA4 |  | RPL34-DT | PRAC1 |
| AGAP2-AS1 |  | KMT2A | TFE3 |
| KLK10 |  | MCM4 | GLI3 |
| TRPS1 |  | ANGPTL4 | LIN28B |
| PRF1 |  | COL18A1 | ALDH18A1 |
| TLR1 |  | BCR | WNT2 |
| MIR219A1 |  | CALB2 | MIR212 |
| MIR198 |  | NUAK1 | PLOD1 |
| STIM1 |  | EXT2 | CEP290 |
| CXCL5 |  | ANGPT1 | SKP1 |
| LEPR |  | CCNH | PAX6 |
| PIN1 |  | RALB | SRD5A1 |
| CD80 |  | GLI3 | F3 |
| E2F5 |  | NGF | POU5F1B |
| DCTN4 |  | MIR422A | ZMIZ1 |
| SLFN11 |  | PIN1 | LIG3 |
| STING1 |  | PTPRG | XAGE1A |
| CCNG1 |  | TLR10 | MAPK10 |
| PTENP1 |  | BCAS1 | CEACAM6 |
| LOC111811965 |  | GPA33 | DVL2 |
| CYP24A1 |  | PRKACB | FGF19 |
| DELEC1 |  | KLK6 | ESRRA |
| TUSC7 |  | DUSP1 | MACROD1 |
| PCLAF |  | ROCK1 | CNOT9 |
| MIR22HG |  | NCOA2 | AGER |
| WWP2 |  | CCNE2 | CDK7 |
| CD63 |  | ABCA1 | RPS19 |
| CD22 |  | MACROD1 | MIR489 |
| ACHE |  | TOX3 | KIF5A |
| LZTR1 |  | PRKAA1 | CHI3L1 |
| RNH1 |  | P2RX7 | PRECSIT |
| MMP11 |  | SFRP5 | PAX3 |
| NGF |  | MIR129-2 | AQP2 |
| FGL1 |  | HP | ABCD1 |
| PIK3C3 |  | OCLN | LMNB1 |
| XBP1 |  | CHD1 | RRAS |
| CCN1 |  | NAMPT | BNIP3 |
| PAWR |  | PRMT7 | MORC2 |
| HLA-C |  | NCOA4 | SAT1 |
| MAP3K2 |  | BMP7 | VTN |
| F5 |  | F2R | ANO1 |
| COL2A1 |  | HOXB8 | TINF2 |
| DNAH5 |  | WEE1 | ACTG1 |
| MED12 |  | LAMC1 | DVL1 |
| AGR2 |  | GAPLINC | MYCL |
| EXT1 |  | TMSB4X | ADAM9 |
| MIR425 |  | CT47A3 | RPL15 |
| IGFBP1 |  | PTGES2 | SYNE1 |
| NOP10 |  | YES1 | TNFRSF11B |
| LBR |  | STK4 | LRP1B |
| UBA7 |  | NGFR | SLC5A8 |
| MTR |  | FENDRR | CSNK1D |
| VTN |  | SNHG15 | PLAGL1 |
| PRSS1 |  | SPDEF | SELE |
| API5 |  | IL11 | WASHC5 |
| HMGA1 |  | RPL5 | MIR361 |
| SEC23B |  | PTPRH | MIR378A |
| H2AX |  | IL24 | CTC1 |
| DLL4 |  | YWHAE | ARNT |
| NKX2-5 |  | GHR | S100P |
| NHP2 |  | AFAP1 | INHA |
| PCAT19 |  | FLI1 | TGFBR3 |
| HSPD1 |  | CT47A1 | SMAD6 |
| PRMT7 |  | CT47A2 | CCR5 |
| NCOA1 |  | CT47A4 | NRP2 |
| CYP3A5 |  | NCOA1 | NAMPT |
| IFNAR1 |  | CAMP | CLDN3 |
| GPC4 |  | TTK | IGFBP4 |
| DAB2IP |  | FURIN | VCAM1 |
| PBRM1 |  | TFRC | CISD2 |
| LRP6 |  | SKP1 | KLK2 |
| GUSB |  | TNIK | DACT1 |
| CUL5 |  | S100A2 | NTS |
| ITGAE |  | REV3L | GDNF |
| APC2 |  | NUMA1 | LTO1 |
| LPAR1 |  | SDC2 | IGFBP6 |
| DOCK8 |  | LOC107303338 | BRDT |
| LIMK1 |  | FBLN1 | SNHG12 |
| CASR |  | IL6ST | EPO |
| ITGAM |  | MIR212 | MIR130B |
| MIR33A |  | FOXC1 | URI1 |
| CLEC7A |  | MAPK13 | FREM2 |
| ITGA11 |  | DPP10-AS1 | TCF7 |
| MIR148B |  | MAPK7 | EFNA1 |
| NFIB |  | DUXAP10 | CCNA1 |
| TAP1 |  | IQANK1 | TGM2 |
| TTR |  | RAC3 | UMPS |
| TRIM24 |  | EFNA1 | PTPN13 |
| AKAP13 |  | SOX5 | PRKCE |
| LOXL2 |  | MIR489 | SLIT2 |
| GATA1 |  | EPHA7 | PIP |
| LASP1 |  | XAGE1B | WFDC2 |
| TCF21 |  | UBE2T | SPAG9 |
| PSG2 |  | RPS19 |  |
| IGF2-AS |  | LAMB3 |  |
| P2RX7 |  | DPH1 |  |
| CT45A5 |  | BUB3 |  |
| NPRL2 |  | FUT6 |  |
| PDPK1 |  | CLPTM1L |  |
| SPHK1 |  | PIM1 |  |
| TUSC3 |  | CAT |  |
| CRABP2 |  | SI |  |
| RRAS |  | RRAS |  |
| EPO |  | EPOR |  |
| FLNB |  | RXRA |  |
| PAX9 |  | CSNK2B |  |
| VDAC1 |  | CIB1 |  |
| SOD3 |  | LIN28B |  |
| IRS2 |  | DCK |  |
| GNA11 |  | TPD52 |  |
| SF3B2 |  | BCAS2 |  |
| FLNC |  | PPP2R2A |  |
| SSX1 |  | RRM1 |  |
| EFNB3 |  | PRKCI |  |
| TGM2 |  | PRDM2 |  |
| PTCH2 |  | MCM3 |  |
| PLA2G1B |  | IDO1 |  |
| CT45A2 |  | BIRC7 |  |
| ST14 |  | MIR455 |  |
| DHCR7 |  | MCM2 |  |
| MIRLET7F1 |  | PROS1 |  |
| S100A6 |  | PLXDC1 |  |
| LRP5 |  | PDPK1 |  |
| NKILA |  | COL7A1 |  |
| MUC2 |  | SRA1 |  |
| IGF2BP3 |  | ASXL1 |  |
| DPH1 |  | RBBP4 |  |
| COL1A2 |  | KMT2D |  |
| STX1A |  | MAGED2 |  |
| KMT2A |  | NKD1 |  |
| TPM3 |  | HAGLR |  |
| LINC00858 |  | GNG4 |  |
| MIR339 |  | CT47A5 |  |
| RSPO2 |  | REEP5 |  |
| METTL13 |  | MYLK |  |
| MIR125B2 |  | CT47A12 |  |
| HERPUD1 |  | ENPP7 |  |
| TCF3 |  | XAGE1A |  |
| ZEB2-AS1 |  | SLC25A25-AS1 |  |
| SEMA4A |  | MIR361 |  |
| H1-0 |  | URI1 |  |
| CBR1 |  | MIR186 |  |
| BGLAP |  | SLX4 |  |
| TTN |  | LINC-PINT |  |
| MECP2 |  | FTX |  |
| DPP4 |  | PAX3 |  |
| WFDC2 |  | SLC29A1 |  |
| CFTR-AS1 |  | PIP |  |
| NTS |  | S100P |  |
| BCOR |  | CTCF |  |
| DGCR5 |  | RPL15 |  |
| DDIAS |  |  |  |
| AMACR |  |  |  |
| FOXK2 |  |  |  |
| STS |  |  |  |
| CD226 |  |  |  |
| IRF4 |  |  |  |
| CDK8 |  |  |  |
| HPSE |  |  |  |
| SPAAR |  |  |  |
| KLF2 |  |  |  |
| BRMS1L |  |  |  |
| TNFRSF11A |  |  |  |
| MIR340 |  |  |  |
| DNAH8 |  |  |  |
| IRF5 |  |  |  |
| SYNE1 |  |  |  |
| DNAJC21 |  |  |  |
| KLRK1 |  |  |  |
| FEZF1-AS1 |  |  |  |
| NES |  |  |  |
| LAMA2 |  |  |  |
| LINC00460 |  |  |  |
| MAPK7 |  |  |  |
| SOX30 |  |  |  |
| AKR1C3 |  |  |  |
| CT45A1 |  |  |  |
| CCDC40 |  |  |  |
| STUB1 |  |  |  |
| ALCAM |  |  |  |
| ATF1 |  |  |  |
| ATF3 |  |  |  |
| PTPN22 |  |  |  |
| SEZ6L |  |  |  |
| MIR365A |  |  |  |
| NTPCR |  |  |  |
| ATAD2 |  |  |  |
| CASC22 |  |  |  |
| LYVE1 |  |  |  |
| FGF4 |  |  |  |
| EPOR |  |  |  |
| ACTN4 |  |  |  |
| EEF1A1 |  |  |  |
| MIR370 |  |  |  |
| USF2 |  |  |  |
| RNY1 |  |  |  |
| RNY3 |  |  |  |
| UHRF1 |  |  |  |
| CXCL9 |  |  |  |
| DIRC3 |  |  |  |
| MST1 |  |  |  |
| TNFRSF6B |  |  |  |
| TMEFF2 |  |  |  |
| NOTCH4 |  |  |  |
| WNT11 |  |  |  |
| CT45A6 |  |  |  |
| FOXP1 |  |  |  |
| TOB1 |  |  |  |
| SCNN1B |  |  |  |
| NAB2 |  |  |  |
| IL7R |  |  |  |
| HYAL1 |  |  |  |
| PCAT14 |  |  |  |
| PCAT18 |  |  |  |
| GZMB |  |  |  |
| FBLN5 |  |  |  |
| SRPK1 |  |  |  |
| CCL17 |  |  |  |
| XAGE1B |  |  |  |
| FOXC1 |  |  |  |
| COTL1 |  |  |  |
| CLDN18 |  |  |  |
| FOSL1 |  |  |  |
| SELE |  |  |  |
| HMGCR |  |  |  |
| MIR154 |  |  |  |
| EDNRB |  |  |  |
| PRLR |  |  |  |
| TBX3 |  |  |  |
| GRN |  |  |  |
| NAMPT |  |  |  |
| NRP2 |  |  |  |
| MIR92A1 |  |  |  |
| TFE3 |  |  |  |
| FGF9 |  |  |  |
| SERPINC1 |  |  |  |
| CEMIP |  |  |  |
| CCBE1 |  |  |  |
| MCM7 |  |  |  |
| BBC3 |  |  |  |
| IRAIN |  |  |  |
| WWTR1 |  |  |  |
| VIP |  |  |  |
| SERPINH1 |  |  |  |
| FOXD2-AS1 |  |  |  |
| GDF1 |  |  |  |
| ADAM17 |  |  |  |
| NECTIN4 |  |  |  |
| MIR218-2 |  |  |  |
| HLA-DPA1 |  |  |  |
| IL12A |  |  |  |
| NEK8 |  |  |  |
| PPIEL |  |  |  |
| MCM2 |  |  |  |
| MIR95 |  |  |  |
| HK2 |  |  |  |
| CCNO |  |  |  |
| CSK |  |  |  |
| ZIC3 |  |  |  |
| SLC9A3 |  |  |  |
| CLDN3 |  |  |  |
| TRPM2-AS |  |  |  |
| IL12RB1 |  |  |  |
| EIF3H |  |  |  |
| XAGE1A |  |  |  |
| PPARA |  |  |  |
| SERPINB2 |  |  |  |
| CD81 |  |  |  |
| DYRK1B |  |  |  |
| TRB |  |  |  |
| LOC111674475 |  |  |  |
| NGFR |  |  |  |
| UBE2T |  |  |  |
| PCAT5 |  |  |  |
| MAPK10 |  |  |  |
| TSHR |  |  |  |
| HNF4A |  |  |  |
| IL9 |  |  |  |
| PIM1 |  |  |  |
| ESM1 |  |  |  |
| LINC00312 |  |  |  |
| WDR19 |  |  |  |
| GJB5 |  |  |  |
| FGF3 |  |  |  |
| DHDH |  |  |  |
| CRNDE |  |  |  |
| EPB41L4A-DT |  |  |  |
| TNFRSF11B |  |  |  |
| HIF1A-AS1 |  |  |  |
| NCOA2 |  |  |  |
| NCOA6 |  |  |  |
| GAS8 |  |  |  |
| PROS1 |  |  |  |
| PCSEAT |  |  |  |
| TNFRSF8 |  |  |  |
| LIFR |  |  |  |
| IQANK1 |  |  |  |
| KDM5B |  |  |  |
| XPO1 |  |  |  |
| LOC111674472 |  |  |  |
| CCL18 |  |  |  |
| RGMB-AS1 |  |  |  |
| LIN28B |  |  |  |
| SH2B3 |  |  |  |
| ITGA9 |  |  |  |
| LTBP4 |  |  |  |
| POU6F2 |  |  |  |
| MUSK |  |  |  |
| NUMA1 |  |  |  |
| LINC01116 |  |  |  |
| ARHGAP5 |  |  |  |
| ZFPM2 |  |  |  |
| MT-ND4L |  |  |  |
| TOX3 |  |  |  |
| CHD7 |  |  |  |
| DIS3L2 |  |  |  |
| BRINP1 |  |  |  |
| ALPP |  |  |  |
| ZNF793 |  |  |  |
| BRS3 |  |  |  |
| CSNK2B |  |  |  |
| SPAG1 |  |  |  |
| STAT4 |  |  |  |
| ACP3 |  |  |  |
| GNB1 |  |  |  |
| TPD52 |  |  |  |
| TP53TG1 |  |  |  |
| IFNB1 |  |  |  |
| COL4A5 |  |  |  |
| PAX3 |  |  |  |
| SBF2-AS1 |  |  |  |
| PMAIP1 |  |  |  |
| DDIT3 |  |  |  |
| OLA1 |  |  |  |
| PTRH2 |  |  |  |
| MEFV |  |  |  |
| HOXC13 |  |  |  |
| FANCL |  |  |  |
| BLCAP |  |  |  |
| E2F4 |  |  |  |
| CXCL2 |  |  |  |
| GIMAP6 |  |  |  |
| ROR2 |  |  |  |
| LINC00472 |  |  |  |
| FZD7 |  |  |  |
| CT45A3 |  |  |  |
| PTH1R |  |  |  |
| RPGR |  |  |  |
| CD86 |  |  |  |
| TP53BP1 |  |  |  |
| MIR326 |  |  |  |
| ELK1 |  |  |  |
| NDUFA13 |  |  |  |
| CLDN1 |  |  |  |
| SRA1 |  |  |  |
| LIF |  |  |  |
| MIRLET7A2 |  |  |  |
| CTBP1 |  |  |  |
| WNT6 |  |  |  |
| CIP2A |  |  |  |
| GUCY2C |  |  |  |
| USP33 |  |  |  |
| CFAP298 |  |  |  |
| EFNA1 |  |  |  |
| RSPH1 |  |  |  |
| IFNGR1 |  |  |  |
| FANCB |  |  |  |
| ADAMTS9-AS2 |  |  |  |
| CDC6 |  |  |  |
| HPS1 |  |  |  |
| SPRY4 |  |  |  |
| BRD4 |  |  |  |
| IL12B |  |  |  |
| ACP5 |  |  |  |
| SPINT2 |  |  |  |
| FZD1 |  |  |  |
| HYDIN |  |  |  |
| CYP27B1 |  |  |  |
| LOC113633877 |  |  |  |
| UBE2K |  |  |  |
| GGPS1 |  |  |  |
| LOC113664106 |  |  |  |
| DNAH9 |  |  |  |
| CIB1 |  |  |  |
| CR2 |  |  |  |
| FBXO5 |  |  |  |
| PSAP |  |  |  |
| PAX6 |  |  |  |
| TNC |  |  |  |
| MAP2K5 |  |  |  |
| IL13RA2 |  |  |  |
| MAPK9 |  |  |  |
| MAD2L1 |  |  |  |
| CLCA4 |  |  |  |
| CTSK |  |  |  |
| HNRNPK |  |  |  |
| GFAP |  |  |  |
| PROX1 |  |  |  |
| RBM38 |  |  |  |
| DNAAF1 |  |  |  |
| TLR7 |  |  |  |
| CHAT |  |  |  |
| OFD1 |  |  |  |
| MMP8 |  |  |  |
| SCGB2A2 |  |  |  |
| COL4A1 |  |  |  |
| MYH11 |  |  |  |
| NUP214 |  |  |  |
| DDX41 |  |  |  |
| PIEZO2 |  |  |  |
| RPS19 |  |  |  |
| SELP |  |  |  |
| IGFBP6 |  |  |  |
| AMPH |  |  |  |
| IFIH1 |  |  |  |
| INHA |  |  |  |
| INTS6 |  |  |  |
| LOC111674477 |  |  |  |
| IKZF1 |  |  |  |
| LPP |  |  |  |
| CIITA |  |  |  |
| LINC00968 |  |  |  |
| SLC45A3 |  |  |  |
| DVL1 |  |  |  |
| PSEN2 |  |  |  |
| ETS2 |  |  |  |
| KCNQ1 |  |  |  |
| AATBC |  |  |  |
| MIR184 |  |  |  |
| FBLN1 |  |  |  |
| SFN |  |  |  |
| PTP4A1 |  |  |  |
| CCK |  |  |  |
| ARID4B |  |  |  |
| LINC01852 |  |  |  |
| SBDS |  |  |  |
| ASAP1-IT1 |  |  |  |
| LCK |  |  |  |
| H3C2 |  |  |  |
| RANBP2 |  |  |  |
| ARL11 |  |  |  |
| SLC29A1 |  |  |  |
| F2RL1 |  |  |  |
| VIPR1 |  |  |  |
| MLLT10 |  |  |  |
| TPM1 |  |  |  |
| SNHG5 |  |  |  |
| MAP2K3 |  |  |  |
| HDAC6 |  |  |  |
| IL33 |  |  |  |
| MAFA-AS1 |  |  |  |
| DNAH11 |  |  |  |
| EFEMP1 |  |  |  |
| CDK7 |  |  |  |
| CCR2 |  |  |  |
| TBK1 |  |  |  |
| WNT7A |  |  |  |
| TNFRSF13B |  |  |  |
| ZNF461 |  |  |  |
| FOXC2 |  |  |  |
| TUBA4B |  |  |  |
| ALOX12 |  |  |  |
| S100P |  |  |  |
| S100B |  |  |  |
| KLK4 |  |  |  |
| MACROD1 |  |  |  |
| SUZ12 |  |  |  |
| CEP290 |  |  |  |
| URI1 |  |  |  |
| CLCN1 |  |  |  |
| BCAS3 |  |  |  |
| PRDM2 |  |  |  |
| TJP1 |  |  |  |
| HPS6 |  |  |  |
| GACAT1 |  |  |  |
| TFF3 |  |  |  |
| HSPG2 |  |  |  |
| MALT1 |  |  |  |
| RHOBTB2 |  |  |  |
| TNFRSF10C |  |  |  |
| FAR2P1 |  |  |  |
| EMSY |  |  |  |
| NCOA4 |  |  |  |
| STARD13 |  |  |  |
| ZBTB16 |  |  |  |
| FRGCA |  |  |  |
| H3-3A |  |  |  |
| LINC01589 |  |  |  |
| PRMT1 |  |  |  |
| PON1 |  |  |  |
| CCR1 |  |  |  |
| WNT10B |  |  |  |
| CPS1-IT1 |  |  |  |
| CD46 |  |  |  |
| MB |  |  |  |
| NUDT1 |  |  |  |
| IRF2BP2 |  |  |  |
| SLC16A1-AS1 |  |  |  |
| TATDN1 |  |  |  |
| LINC00313 |  |  |  |
| MIR378A |  |  |  |
| RPL11 |  |  |  |
| LOC100506321 |  |  |  |
| ODAD1 |  |  |  |
| CT62 |  |  |  |
| ECM1 |  |  |  |
| CT45A7 |  |  |  |
| DNAI1 |  |  |  |
| INSM1 |  |  |  |
| SCNN1G |  |  |  |
| PIAS1 |  |  |  |
| ANO1 |  |  |  |
| NR3C2 |  |  |  |
| KLK6 |  |  |  |
| MECOM |  |  |  |
| NT5E |  |  |  |
| RHOC |  |  |  |
| RBPJ |  |  |  |
| ASAH1 |  |  |  |
| ELF3 |  |  |  |
| CYBB |  |  |  |
| CAMP |  |  |  |
| SCAT1 |  |  |  |
| KAT5 |  |  |  |
| CCDC39 |  |  |  |
| FZD8 |  |  |  |
| ERGIC3 |  |  |  |
| CARD11 |  |  |  |
| SMARCA2 |  |  |  |
| HPRT1 |  |  |  |
| LINC00473 |  |  |  |
| AGO2 |  |  |  |
| PCBP2-OT1 |  |  |  |
| CT47B1 |  |  |  |
| IFT140 |  |  |  |
| PPARGC1A |  |  |  |
| PTPN1 |  |  |  |
| IKBKG |  |  |  |
| SLX4 |  |  |  |
| ESRRA |  |  |  |
| DNAI7 |  |  |  |
| PRAL |  |  |  |
| FAP |  |  |  |
| GDNF |  |  |  |
| SFTA1P |  |  |  |
| PPARD |  |  |  |
| IL2RB |  |  |  |
| REN |  |  |  |
| ARNT |  |  |  |
| GGT1 |  |  |  |
| BNIP3 |  |  |  |
| ADAM9 |  |  |  |
| BCAS1 |  |  |  |
| GJB2 |  |  |  |
| CUL3 |  |  |  |
| MFN2 |  |  |  |
| GPSM2 |  |  |  |
| OCA2 |  |  |  |
| GDF2 |  |  |  |
| ADA2 |  |  |  |
| CD55 |  |  |  |
| BUB1B-PAK6 |  |  |  |
| CT47A6 |  |  |  |
| FZD6 |  |  |  |
| NORAD |  |  |  |
| LINC02633 |  |  |  |
| RAG2 |  |  |  |
| GAS5-AS1 |  |  |  |
| C4A |  |  |  |
| CT47A3 |  |  |  |
| ZMYND8 |  |  |  |
| G6PD |  |  |  |
| MTHFD1 |  |  |  |
| DCN |  |  |  |
| CT45A10 |  |  |  |
| LINC00857 |  |  |  |
| XRCC4 |  |  |  |
| LINC01433 |  |  |  |
| SPTAN1 |  |  |  |
| CHRM3 |  |  |  |
| SLC5A8 |  |  |  |
| CYP27A1 |  |  |  |
| TRIM37 |  |  |  |
| DLGAP2 |  |  |  |
| ARID1B |  |  |  |
| RBBP4 |  |  |  |
| TRPM8 |  |  |  |
| HES1 |  |  |  |
| GAA |  |  |  |
| TBX1 |  |  |  |
| MUC20-OT1 |  |  |  |
| SH3GL1 |  |  |  |
| SULF1 |  |  |  |
| RNASE3 |  |  |  |
| NEK10 |  |  |  |
| SOX11 |  |  |  |
| UMPS |  |  |  |
| USP7 |  |  |  |
| LGALS3BP |  |  |  |
| MIR376A1 |  |  |  |
| ELP1 |  |  |  |
| CDC25B |  |  |  |
| DAB2 |  |  |  |
| MIR511 |  |  |  |
| NPTN-IT1 |  |  |  |
| PTGER2 |  |  |  |
| RPL15 |  |  |  |
| GHRH |  |  |  |
| CD164 |  |  |  |
| PICALM |  |  |  |
| SUSD2 |  |  |  |
| AQP3 |  |  |  |
| PTK6 |  |  |  |
| SDC1 |  |  |  |
| CITED2 |  |  |  |
| MELK |  |  |  |
| MYH7 |  |  |  |
| FOXA2 |  |  |  |
| MUC7 |  |  |  |
| LATS2 |  |  |  |
| NMRAL2P |  |  |  |
| STK36 |  |  |  |
| KCNMB2-AS1 |  |  |  |
| PELP1 |  |  |  |
| PHLPP1 |  |  |  |
| CT45A8 |  |  |  |
| CT45A9 |  |  |  |
| IBSP |  |  |  |
| MIR328 |  |  |  |
| EVC2 |  |  |  |
| TNFSF12 |  |  |  |
| PSAT1 |  |  |  |
| KISS1 |  |  |  |
| ADAR |  |  |  |
| DVL2 |  |  |  |
| FAT4 |  |  |  |
| ID2 |  |  |  |
| GPT |  |  |  |
| CKB |  |  |  |
| SLC39A1 |  |  |  |
| GNAS-AS1 |  |  |  |
| DKK3 |  |  |  |
| DNAAF4 |  |  |  |
| DNAAF5 |  |  |  |
| CYP2C19 |  |  |  |
| CSNK1D |  |  |  |
| CCL22 |  |  |  |
| GHRL |  |  |  |
| TCF7L1 |  |  |  |
| GAS1 |  |  |  |
| CASC16 |  |  |  |
| NCF2 |  |  |  |
| PTN |  |  |  |
| SRSF2 |  |  |  |
| PGF |  |  |  |
| HSPA1A |  |  |  |
| SMC1A |  |  |  |
| GAS6-AS1 |  |  |  |
| CC2D2A |  |  |  |
| TPO |  |  |  |
| F13A1 |  |  |  |
| PLA2G10 |  |  |  |
| GUCY1B2 |  |  |  |
| LINC00210 |  |  |  |
| MIR26B |  |  |  |
| FUS |  |  |  |
| ARHGAP27P1 |  |  |  |
| LINC00880 |  |  |  |
| TAC1 |  |  |  |
| LSM1 |  |  |  |
| ERN1 |  |  |  |
| CSNK1E |  |  |  |
| PLCE1 |  |  |  |
| CXCL14 |  |  |  |
| GMNN |  |  |  |
| IFI16 |  |  |  |
| HPN |  |  |  |
| TPR |  |  |  |
| PDCD1LG2 |  |  |  |
| ICOS |  |  |  |
| EFEMP2 |  |  |  |
| BACH1 |  |  |  |
| NTN1 |  |  |  |
| CX3CR1 |  |  |  |
| INSL6 |  |  |  |
| GH1 |  |  |  |
| LOC730101 |  |  |  |
| MS4A1 |  |  |  |
| CDC20 |  |  |  |
| SGK1 |  |  |  |
| CHD1 |  |  |  |
| DNAAF11 |  |  |  |
| LOC107303338 |  |  |  |
| C14orf132 |  |  |  |
| LIG1 |  |  |  |
| MIR489 |  |  |  |
| COL14A1 |  |  |  |
| AAGAB |  |  |  |
| KIF15 |  |  |  |
| CTC1 |  |  |  |
| TTC7A |  |  |  |
| MAGED2 |  |  |  |
| CT47A1 |  |  |  |
| CT47A11 |  |  |  |
| CT47A2 |  |  |  |
| CT47A4 |  |  |  |
| CCR4 |  |  |  |
| WAS |  |  |  |
| NEU1 |  |  |  |
| FZD4 |  |  |  |
| AFAP1 |  |  |  |
| JAG2 |  |  |  |
| RNF139 |  |  |  |
| RBX1 |  |  |  |
| ZMIZ1 |  |  |  |
| S100A1 |  |  |  |
| IL1RL1 |  |  |  |
| TFPI |  |  |  |
| SLC6A3 |  |  |  |
| SDCCAG8 |  |  |  |
| MYEF2 |  |  |  |
| MIR151A |  |  |  |
| TF |  |  |  |
| CYBA |  |  |  |
| RSPH9 |  |  |  |
| DTNBP1 |  |  |  |
| CSF2RA |  |  |  |
| TRPC6 |  |  |  |
| SERPINF1 |  |  |  |
| CDH17 |  |  |  |
| CDC27 |  |  |  |
| SLIT2 |  |  |  |
| KLK2 |  |  |  |
| RPS15 |  |  |  |
| TNFAIP3 |  |  |  |
| PICART1 |  |  |  |
| CT47A7 |  |  |  |
| CT47A8 |  |  |  |
| CT47A9 |  |  |  |
| RALA |  |  |  |
| BTRC |  |  |  |
| CDH11 |  |  |  |
| CHRNA1 |  |  |  |
| FURIN |  |  |  |
| SGO1-AS1 |  |  |  |
| PLA2G7 |  |  |  |
| NOG |  |  |  |
| ZFP36 |  |  |  |
| FUT4 |  |  |  |
| TYK2 |  |  |  |
| FEN1 |  |  |  |
| LINC01186 |  |  |  |
| ENSG00000266919 |  |  |  |
| CACNA1G-AS1 |  |  |  |
| COL7A1 |  |  |  |
| LINC00342 |  |  |  |
| FES |  |  |  |
| MIR130B |  |  |  |
| CXADR |  |  |  |
| USB1 |  |  |  |
| HOXA-AS2 |  |  |  |
| PRKG1 |  |  |  |
| SLC43A1 |  |  |  |
| HIPK2 |  |  |  |
| FCGR3A |  |  |  |
| NEXN-AS1 |  |  |  |
| SLC9A3R1 |  |  |  |
| ANXA3 |  |  |  |
| MIR92A2 |  |  |  |
| PLOD1 |  |  |  |
| HPS3 |  |  |  |
| GRB7 |  |  |  |
| IKZF3 |  |  |  |
| NOS1 |  |  |  |
| COL11A1 |  |  |  |
| ARRB1 |  |  |  |
| CCAR2 |  |  |  |
| MAPK12 |  |  |  |
| HDAC5 |  |  |  |
| SMAD9 |  |  |  |
| FKBP5 |  |  |  |
| NEK2 |  |  |  |
| AQP5 |  |  |  |
| GNRHR |  |  |  |
| MAP3K7 |  |  |  |
| IKBKE |  |  |  |
| CA2 |  |  |  |
| HOXA5 |  |  |  |
| RPS24 |  |  |  |
| SEMA3A |  |  |  |
| ZAP70 |  |  |  |
| AVP |  |  |  |
| HPS5 |  |  |  |
| GPC6 |  |  |  |
| IGFBP4 |  |  |  |
| ACTA1 |  |  |  |
| SPEF2 |  |  |  |
| RSPH4A |  |  |  |
| THRB |  |  |  |
| VAV3 |  |  |  |
| SCAP |  |  |  |
| HTATIP2 |  |  |  |
| MIR675 |  |  |  |
| ODAD2 |  |  |  |
| FAM3C |  |  |  |
| CSF3R |  |  |  |
| NNT-AS1 |  |  |  |
| ELF5 |  |  |  |
| NPPB |  |  |  |
| GSDME |  |  |  |
| CD27 |  |  |  |
| CBS |  |  |  |
| NKX3-1 |  |  |  |
| PRC1 |  |  |  |
| HOXB2 |  |  |  |
| TBL1XR1 |  |  |  |
| S100A14 |  |  |  |
| LDLR |  |  |  |
| CT47A5 |  |  |  |
| LACTB |  |  |  |
| PPP1CB |  |  |  |
| CT47A12 |  |  |  |
| PTPA |  |  |  |
| UBE2C |  |  |  |
| RIT1 |  |  |  |
| TERF1 |  |  |  |
| PAX7 |  |  |  |
| MIR584 |  |  |  |
| RPS10 |  |  |  |
| RBP4 |  |  |  |
| TMPRSS4 |  |  |  |
| NIPBL |  |  |  |
| FOLR1 |  |  |  |
| CEP85L |  |  |  |
| TNS4 |  |  |  |
| FSHR |  |  |  |
| SPIN1 |  |  |  |
| HSP90B1 |  |  |  |
| HNRNPA1 |  |  |  |
| TFAP2C |  |  |  |
| HNMT |  |  |  |
| AGTR2 |  |  |  |
| SOX10 |  |  |  |
| ANKRD30A |  |  |  |
| LGR5 |  |  |  |
| SPON2 |  |  |  |
| PORCN |  |  |  |
| LTO1 |  |  |  |
| TGFBR3 |  |  |  |
| RAD21 |  |  |  |
| WNT5B |  |  |  |
| TUBA1A |  |  |  |
| ELOC |  |  |  |
| DNAAF2 |  |  |  |

| **Breast cancer** | **Oral Squamous Cell Carcinoma** | | **Nasopharyngeal carcinoma** | **Hepatocellular carcinoma** |
| --- | --- | --- | --- | --- |
| ACVR1B | AAMP | | ANC | ANC |
| ADH1B | ABCB6 | | AXIN1 | AXIN1 |
| AKT1 | ACKR1 | | BCAS4 | BCAS4 |
| ALDH2 | ADH1B | | BCC1 | BCC1 |
| APC | ALCAM | | BCC2 | BCC2 |
| AR | BANK1 | | BCC3 | BCC3 |
| ATM | BCAP29 | | BCC4 | BCC4 |
| ATR | BCC1 | | BCC5 | BCC5 |
| AURKA | BCC2 | | BCC6 | BCC6 |
| AXIN2 | BCC3 | | CASP8 | CASP8 |
| BARD1 | BCC6 | | CDC73 | CDC73 |
| BAX | BCL10 | | CDH1 | CDH1 |
| BCAR3 | BCL11A | | CRTC1 | CRTC1 |
| BCAR4 | BCL7B | | CTNNB1 | CTNNB1 |
| BCAS4 | BCL9 | | DCC | DCC |
| BCPR | BLACE | | DIRC3 | DIRC3 |
| BLACAT1 | BOD1 | | FAM107A | FAM107A |
| BLCAP | BRAF | | FAS | FAS |
| BRAF | BST1 | | FLCN | FLCN |
| BRCA1 | C5orf20 | | GHET1 | GHET1 |
| BRCA2 | CADM2 | | HCCAT5 | HCCAT5 |
| BRIP1 | CADM3 | | HDMCP | HDMCP |
| BUB1 | CARD11 | | HEIH | HEIH |
| BUB1B | CASP2 | | HNF1A | HNF1A |
| CASC1 | CASP8 | | HNF1B | HNF1B |
| CASC11 | CCAR2 | | HRAS | HRAS |
| CASC15 | CD2 | | HULC | HULC |
| CASC19 | CD48 | | IGF2R | IGF2R |
| CASC2 | CDC14A | | ING1 | ING1 |
| CASC21 | CDC20 | | KRAS | KRAS |
| CASC8 | CDC23 | | LOC344967 | LOC344967 |
| CASP10 | CDC25A | | LZTS1 | LZTS1 |
| CASP8 | CDC25C | | MAML2 | MAML2 |
| CCAT1 | CDC40 | | MET | MET |
| CCAT2 | CDC42 | | MINPP1 | MINPP1 |
| CCND1 | CDC5L | | MSH3 | MSH3 |
| CDH1 | CDC7 | | MSPC | MSPC |
| CDK2AP1 | CDC73 | | MST1R | MST1R |
| CDKN2A | CDCA2 | | NDUFA13 | NDUFA13 |
| CHEK2 | CDCA7 | | NLRP1 | NLRP1 |
| CHRNA3 | CDCA7L | | NMTC3 | NMTC3 |
| CHRNA5 | CDCA8 | | NPC1 | NPC1 |
| COLCA1 | CGREF1 | | NPCA2 | NPCA2 |
| COLCA2 | CHL1 | | NRAS | NRAS |
| CRCS11 | CIDEC | | NUTM1 | NUTM1 |
| CRCS2 | CIP2A | | OGG1 | OGG1 |
| CRCS5 | CLNK | | PBRM1 | PBRM1 |
| CRCS6 | CPA3 | | PICSAR | PDGFRL |
| CRCS7 | CRB1 | | PIK3CA | PICSAR |
| CRCS8 | CTAGE4 | | PRCC | PIK3CA |
| CRCS9 | CTNNB1 | | PTCH1 | PRCC |
| CT47A10 | CWH43 | | PTCH2 | PTCH1 |
| CT47A11 | CYREN | | PTCPRN | PTCH2 |
| CT47A8 | DCLAMP | | PTCSC1 | PTCPRN |
| CT47A9 | DEFA5 | | RASA1 | PTCSC1 |
| CT47B1 | DEFA6 | | RBBP8 | RASA1 |
| CT83 | DELE1 | | RET | RBBP8 |
| CTAG3 | DIRC3 | | RNF139 | RET |
| CTNNB1 | EBF | | RNF6 | RNF139 |
| CTTN | ECSCR | | RRAS2 | RNF6 |
| CYP2A6 | ECT2 | | RSPO1 | RRAS2 |
| DCC | EGFR | | SERPINB3 | RSPO1 |
| DIRC1 | EIF4G1 | | SERPINB4 | SERPINB3 |
| DLC1 | ELMO1 | | SLC49A4 | SERPINB4 |
| DLEC1 | ESM1 | | SMO | SLC49A4 |
| EGFR | ESRG | | ST3 | SMO |
| EHBP1 | EVA1A | | TCO | ST3 |
| EIF4G1 | FAM107A | | TFE3 | TCO |
| ELAC2 | FDCSP | | TNFRSF10B | TFE3 |
| EP300 | FH | | TP53 | TNFRSF10B |
| EPCAM | FREB | | TSHR | TP53 |
| EPHB2 | GCM1 | | VHL | TSHR |
| ERBB2 | GCM2 | | WWOX | VHL |
| ERCC6 | GDNF | | ALPS4 | WWOX |
| ESR1 | GHET1 | | AXIN | ALPS4 |
| FALEC | GMCL1 | | BHD | AXIN |
| FASLG | GMCL2 | | C14orf68 | BHD |
| FGFR2 | HAVCR2 | | CHNG1 | C14orf68 |
| FGFR3 | HCLS1 | | CLOVE | CHNG1 |
| FGFR4 | HECA | | DFNB97 | CLOVE |
| FH | HEIH | | DIRC2 | DFNB97 |
| FLCN | HESX1 | | DR5 | DIRC2 |
| FOXE1 | HK3 | | DRR1 | DR5 |
| GACAT2 | HMCES | | F37 | DRR1 |
| GACAT3 | HTLF | | FAP4 | F37 |
| GAEC1 | HULC | | FLJ40906 | FAP4 |
| GALNT12 | ICA1 | | FOR | FLJ40906 |
| GCRG224 | ICK | | GAP | FOR |
| HABP2 | IGF2R | | GRIM19 | GAP |
| HEPN1 | IL12A | | HIPER1 | GRIM19 |
| HIC1 | IL12B | | HRPT2 | HIPER1 |
| HIC2 | INTU | | HTA | HRPT2 |
| HMMR | IRF1 | | KRAS2 | HTA |
| HMPS1 | ITK | | LINC00162 | KRAS2 |
| HOXB13 | KIT | | LINC00848 | LINC00162 |
| HPC10 | LAKLG | | MAM3 | LINC00848 |
| HPC11 | LAT2 | | MCH5 | MAM3 |
| HPC14 | LECT2 | | MECT1 | MCH5 |
| HPC15 | LHCGR | | MEN2A | MECT1 |
| HPC3 | LOC344967 | | MPRI | MEN2A |
| HPC4 | LZTS1 | | MRMV1 | MPRI |
| HPC5 | MAK | | NALP1 | MRMV1 |
| HPC6 | MALL | | NBCCS | NALP1 |
| HPC7 | MCF2L2 | | NCRNA00197 | NBCCS |
| HPC9 | MCL1 | | NEDSDV | NCRNA00197 |
| HPCQTL19 | MEMO1 | | NPCA1 | NEDSDV |
| HPCX2 | MET | | P53 | NPCA1 |
| HRAS | MNDA | | PB1 | P53 |
| IL1B | MSH3 | | PRN1 | PB1 |
| IL1RN | MST1R | | RCCP1 | PDGRL |
| IQGAP1 | MUC1 | | RCCX1 | PRN1 |
| IRF1 | MUC13 | | RIM | RCCP1 |
| KLF6 | MUC20 | | RON | RCCX1 |
| KMHN1 | MUC21 | | SCCA1 | RIM |
| KRAS | MYB | | SCCA2 | RON |
| LCO | MYCT1 | | SMOH | SCCA1 |
| LNCR1 | MZB1 | | TC21 | SCCA2 |
| LNCR3 | NEDD9 | | TCF1 | SMOH |
| LNCR4 | NKAIN2 | | TCF2 | TC21 |
| LNCR5 | NKS1 | | TNFRSF6 | TCF1 |
| LUCAT1 | NMTC3 | | TRC8 | TCF2 |
| MAD1L1 | NOS3 | | UVO | TNFRSF6 |
| MAP3K8 | NPC1 | | ALPS2B | TRC8 |
| MCC | NPCA2 | | APT1 | UVO |
| MLH1 | NRAS | | BAF180 | ALPS2B |
| MLH3 | NRCAM | | BCNS | APT1 |
| MPO | NTT | | C1orf28 | BAF180 |
| MSH2 | OGG1 | | CMAVM1 | BCNS |
| MSH6 | PARD3B | | CRJS | C1orf28 |
| MSMB | PBCA | | EVR7 | CMAVM1 |
| MUTYH | PBRM1 | | FEZ1 | CRJS |
| MXI1 | PBX1 | | HGPPS2 | EVR7 |
| NCOA3 | PBX2 | | HNF2 | FEZ1 |
| NKX2-1 | PDCD1 | | HSCR1 | HGPPS2 |
| NQO1 | PDCD10 | | KIAA0616 | HNF2 |
| NQO2 | PDCD2 | | KIAA0926 | HSCR1 |
| NRAS | PDCD6 | | LCAM | KIAA0616 |
| OPCML | PDCD6IP | | LFS1 | KIAA0926 |
| ORAOV1 | PIK3CA | | MC1DN28 | LCAM |
| OVCAS1 | PRCC | | MCAP | LFS1 |
| PALB2 | PSHK2 | | MODY3 | MC1DN28 |
| PALLD | PTCH2 | | MRXSPF | MCAP |
| PBOV1 | PTCPRN | | NLC1C | MODY3 |
| PCA3 | PTCRA | | NPCA3 | MRXSPF |
| PCAP | PTPRN | | NS12 | NLC1C |
| PCAT1 | RAP1GDS1 | | NS6 | NPCA3 |
| PCAT18 | RASA1 | | OSFD | NS12 |
| PCAT19 | RHEX | | PCH16 | NS6 |
| PCAT2 | RIPOR2 | | RASK2 | OSFD |
| PCAT29 | RSPO1 | | RCA1 | PCH16 |
| PCAT4 | SCLC1 | | RCC4 | PRLTS |
| PDGFRL | SDF4 | | SCAR12 | RASK2 |
| PHB1 | SDK1 | | SCKL2 | RCA1 |
| PIK3CA | SLC49A4 | | TRAILR2 | RCC4 |
| PLA2G2A | SMO | | TU3A | SCAR12 |
| PMS2 | SPDYA | | BCC7 | SCKL2 |
| POLD1 | SPDYE1 | | CD95 | TRAILR2 |
| POLE | SPDYE2 | | CMNS | TU3A |
| PPM1D | SPDYE3 | | DEE28 | BCC7 |
| PPP2R1B | TAGAP | | DEFCAP | CD95 |
| PRKN | TAL1 | | ECAD | CMNS |
| PRLR | TARP | | FLJ14027 | DEE28 |
| PRNCR1 | TCF7 | | HPE7 | DEFCAP |
| PTEN | TCL4 | | IDDM20 | ECAD |
| PTPN12 | TCTA | | JWDS | FLJ14027 |
| PTPRF | TIAM2 | | MCM | HPE7 |
| PTPRJ | TIGIT | | NS | IDDM20 |
| RABL3 | TIMED4 | | PHLS | JWDS |
| RAD51 | TLX2 | | PKWS | MCM |
| RAD51C | TLX3 | | RCAD | NS |
| RAD51D | TNFRSF10B | | RCC | PHLS |
| RAD54B | TOP2B | | ALPS1A | PKWS |
| RAD54L | TRAT1 | | BCDS1 | RCAD |
| RB1 | TRBC1 | | BMFS5 | RCC |
| RB1CC1 | TRBC2 | | CARD7 | ALPS1A |
| RHBDF2 | TRBD1 | | CFC2 | BCDS1 |
| RNASEL | TRBD2 | | MCMTC | BMFS5 |
| RNF43 | TRBJ@ | | NCMS | CARD7 |
| SASH1 | TRBV@ | | T2D | CFC2 |
| SCAI | TREM1 | | ADTKD3 | MCMTC |
| SCHLAP1 | TREM2 | | CWS5 | NCMS |
| SCLC1 | TREML1 | | RALD | T2D |
| SLC22A1L | TREML2 | | VAMAS1 | ADTKD3 |
| SMAD4 | TREML3 | | CLAPO | CWS5 |
| SMAD7 | TREML4 | | MSPC | RALD |
| SNCG | TRGC1 | | OES | VAMAS1 |
| SRC | TRGC2 | | AIADK | CLAPO |
| SRGAP1 | TRGJ@ | | CCM4 | MSPC |
| ST7 | TRGV@ | | JRRP | OES |
| STK11 | TRNT1 | | PTEN | AIADK |
| TFF1 | TS546 | | EGFR | CCM4 |
| TGFBR2 | TTIM1 | | CDKN2A | JRRP |
| TLR2 | URGCP | | ERBB2 | PTEN |
| TP53 | VANGL2 | | APC | EGFR |
| TSG11 | VCAM1 | | BRAF | CDKN2A |
| UCA1 | VCTN1 | | AKT1 | ERBB2 |
| VOPP1 | VHL | | MIR21 | APC |
| XRCC3 | WDPCP | | BRCA1 | BRAF |
| ZFHX3 | ZYG11A | | ATM | AKT1 |
| ACH | ZYG11B | | MLH1 | MIR21 |
| ACSTD1 | ADH2 | | MTOR | BRCA1 |
| ACVRLK4 | AIP1 | | CCND1 | ATM |
| ADH2 | ALG2 | | TERT | MLH1 |
| AIB1 | ALPS4 | | MIR29C | MTOR |
| ALPS4 | APC8 | | SMAD4 | CCND1 |
| ASV | ARHGEF22 | | MIR141 | TERT |
| ATA | B7H4 | | CHEK2 | MIR29C |
| ATBF1 | BAP29 | | FGFR3 | SMAD4 |
| BACH1 | BCM1 | | MIR17 | MIR141 |
| BC10 | BENE | | MIR34A | CHEK2 |
| BCEI | BILU | | RB1 | FGFR3 |
| BCSG1 | C1orf186 | | FHIT | MIR17 |
| BEK | C2orf86 | | MIR100 | MIR34A |
| BHD | C4orf7 | | PMS2 | RB1 |
| BUBR1 | C6orf205 | | VEGFA | FHIT |
| BWSCR1A | C7orf49 | | MIR200C | MIR100 |
| C9orf126 | CAB45 | | MIR146A | PMS2 |
| CAGE1 | CAKUHED | | STK11 | VEGFA |
| CARLO5 | CALL | | MIR222 | MIR200C |
| CC1 | CARMA1 | | ESR1 | MIR146A |
| CKN2 | CDC7L1 | | MIR205 | STK11 |
| CLOVE | CED12 | | TGFBR2 | MIR222 |
| CMM6 | CGR11 | | MIR145 | ESR1 |
| COCA1 | CLOVE | | MIR143 | MIR205 |
| COCA2 | CTIP1 | | NFKBIA | TGFBR2 |
| COPEB | DARC | | SETD2 | MIR145 |
| COT | DBC1 | | NKX2-1 | MIR143 |
| CRAC1 | DCNP1 | | MIR18A | NFKBIA |
| CRCS1 | DEF5 | | LRRC56 | SETD2 |
| CRCS10 | DEF6 | | MIR34C | NKX2-1 |
| CRCS12 | DFNB32 | | EP300 | MIR18A |
| CT47A13 | DFNB97 | | FGFR2 | LRRC56 |
| CWS6 | DIRC2 | | MIR200B | MIR34C |
| CYP2A3 | DR5 | | BCL2 | EP300 |
| DD3 | DRR1 | | EPCAM | FGFR2 |
| DEP1 | EC1 | | MYC | MIR200B |
| DHTR | ECSM2 | | BAX | BCL2 |
| DIA4 | EIF4G | | ALK | EPCAM |
| DLC1 | EMT | | MIR93 | MYC |
| DOC1 | F37 | | MDM2 | BAX |
| ECOP | FAM44B | | MIR155 | ALK |
| EIF4G | FAM65B | | KIT | MIR93 |
| EMS1 | FAP4 | | MIR125A | MDM2 |
| EPHT3 | FLJ20706 | | MIR20A | MIR155 |
| ESR | FLJ40906 | | FASLG | KIT |
| FAL1 | FSP27 | | MMP9 | MIR125A |
| FANCD1 | GAP | | CDKN1A | MIR20A |
| FANCN | GCMA | | HIF1A | FASLG |
| FANCO | GCMB | | MIR31 | MMP9 |
| FKHL15 | HDC | | MUC1 | CDKN1A |
| FRP1 | HEF1 | | MIR195 | HIF1A |
| GDEP | HESRG | | NBN | MIR31 |
| GS | HLRCC | | MIR200A | MUC1 |
| GTBP | HOX11L1 | | MIR203A | MIR195 |
| HLRCC | HOX11L2 | | MIR27A | NBN |
| HNPCC6 | HOX12 | | STAT3 | MIR200A |
| HNPCC7 | HRPT2 | | KRT7 | MIR203A |
| HPC13 | HSCR3 | | CD274 | MIR27A |
| HPC2 | IA2 | | NTRK1 | STAT3 |
| HPC9 | IGSF4B | | MIR210 | KRT7 |
| HR54 | IGSF4D | | CDKN1B | CD274 |
| HRG22 | IMD37 | | EGF | NTRK1 |
| IRHOM2 | JPO1 | | MIR30E | MIR210 |
| KIAA0790 | KIAA0141 | | MIR10B | CDKN1B |
| KIAA0903 | KIAA1284 | | MIR223 | EGF |
| KIAA0992 | KIAA1524 | | CD44 | MIR30E |
| KIAA1304 | LAKL | | MIR34B | MIR10B |
| KKLC1 | LHR | | MMP2 | MIR223 |
| KRAS2 | LINC00848 | | MIR19A | CD44 |
| LAR | LTAP | | PTGS2 | MIR34B |
| LAS1 | MAR | | MIR29A | MMP2 |
| LINC00178 | MCH5 | | FBXW7 | MIR19A |
| LINC00340 | MEMO | | MIR148A | PTGS2 |
| LINC00860 | MIST | | NPCA1 | MIR29A |
| LINC00912 | MPRI | | MIR15A | FBXW7 |
| LINC00990 | MRK | | CDK4 | MIR148A |
| LINC01092 | MTABC3 | | MIR185 | NPCA1 |
| LINC01190 | MTLC | | TP63 | MIR15A |
| LINC01244 | NEDD2 | | PPARG | CDK4 |
| LINC01245 | NEDNMS | | MIR106B | MIR185 |
| LINC01458 | NEDSDV | | PGR | TP63 |
| LNCR2 | NISBD2 | | MKI67 | PPARG |
| LOC255313 | NKSF2 | | CDKN3 | MIR106B |
| MADH4 | NPCA1 | | MIR483 | PGR |
| MADH7 | NS7 | | MIR98 | MKI67 |
| MAR | OLF1 | | CASP3 | CDKN3 |
| MCH4 | PACAP | | MIR122 | MIR483 |
| MCH5 | PB1 | | SDHB | MIR98 |
| MFAB | PBT | | XRCC1 | CASP3 |
| MMAC1 | PGAP2IP | | CXCR4 | MIR122 |
| MRMV1 | PLOSL2 | | EZH2 | SDHB |
| MTCL1AS1 | PPP1R81 | | H19 | XRCC1 |
| MTS1 | PRN1 | | GSTM1 | CXCR4 |
| MVCD4 | PRP17 | | XRCC3 | EZH2 |
| MYH | PUM | | CA9 | H19 |
| NEDSDV | R1 | | MIR23B | GSTM1 |
| NGL | RCCP1 | | MIR335 | XRCC3 |
| NISBD2 | RON | | RASSF1 | CA9 |
| NMOR2 | RP12 | | BIRC5 | MIR23B |
| NS7 | RP62 | | KRT19 | MIR335 |
| ODCRCS | RPX | | SRC | RASSF1 |
| P53 | SIFD | | MIR204 | BIRC5 |
| PARK2 | SLEB2 | | FH | KRT19 |
| PCA2 | SMOH | | IL6 | SRC |
| PCAT114 | SPATA29 | | MIR373 | MIR204 |
| PCAT8 | SPDYB2 | | MMP1 | FH |
| PDGRL | SPDYB2L1 | | MIR96 | IL6 |
| PHBP | SPDYB2L2 | | NME1 | MIR373 |
| PJS | SPY1 | | RAD51 | MMP1 |
| PLA2B | TCBA | | IGF2 | MIR96 |
| PMSL2 | TCL5 | | CXCL8 | NME1 |
| PNCA5 | TFAR15 | | VEGFC | RAD51 |
| PRAD1 | TIM3 | | TNF | IGF2 |
| PSCP | TIM4 | | MIRLET7C | CXCL8 |
| PTPG1 | TKS | | NOTCH1 | VEGFC |
| RAD51L3 | TLT1 | | MIR182 | TNF |
| RAD53 | TLT2 | | VIM | MIRLET7C |
| RECA | TLT3 | | MIRLET7G | NOTCH1 |
| RNF124 | TLT4 | | MIR196A2 | MIR182 |
| RNS4 | TMEM166 | | TGFB1 | VIM |
| RSTS2 | TRIM | | KDR | MIRLET7G |
| SAR1 | URG4 | | JUN | MIR196A2 |
| SCAL1 | WBSCR5 | | MIR429 | TGFB1 |
| SH2D3B | ADTKD2 | | MIR451A | KDR |
| STK15 | ALIX | | BCL2L1 | JUN |
| TAOS1 | ALPS2B | | MIR25 | MIR429 |
| TIL4 | ARIA | | KRT20 | MIR451A |
| TITF1 | B29 | | PCNA | BCL2L1 |
| TNFSF6 | B7X | | MIR192 | MIR25 |
| TSG7 | BAF180 | | MIR23A | KRT20 |
| TXBP181 | BANK | | IL1B | PCNA |
| UROC28 | BBS15 | | SFPQ | MIR192 |
| UVO | BIMP3 | | SMARCB1 | MIR23A |
| WIP1 | BLAST1 | | MIR197 | IL1B |
| AAT3 | BTBD13 | | IFNG | SFPQ |
| ALK4 | C1orf28 | | AURKA | SMARCB1 |
| ALPS2 | C2orf4 | | TP73 | MIR197 |
| ALPS2B | C6orf32 | | ERCC2 | IFNG |
| APT1LG1 | CASL | | MIR224 | AURKA |
| AT1 | CCM3 | | TWIST1 | TP73 |
| AURORA2 | CIDE3 | | MIR181A1 | ERCC2 |
| BCD1 | CMAVM1 | | MIR183 | MIR224 |
| BCL1 | CPHD5 | | NFE2L2 | TWIST1 |
| BNAH2 | CRJS | | IRF1 | MIR181A1 |
| BROVCA1 | EVI9 | | MIR214 | MIR183 |
| BROVCA2 | EVR7 | | MIR140 | NFE2L2 |
| BROVCA3 | FAM176A | | FGF2 | IRF1 |
| BROVCA4 | FEZ1 | | MIR486-1 | MIR214 |
| CAPOK | FY | | PRKN | MIR140 |
| CARLO1 | G17 | | MALAT1 | FGF2 |
| CARLO2 | HRPT4 | | MIR296 | MIR486-1 |
| CARLO4 | ICF3 | | MIR146B | PRKN |
| CARLO6 | ICH1 | | MIR372 | MALAT1 |
| CARLO7 | IMD29 | | HOTAIR | MIR296 |
| CFD1 | JPO2 | | KLF6 | MIR146B |
| CHK2 | KIAA0861 | | GSTP1 | MIR372 |
| COFS1 | KIAA0936 | | CDKN2B | HOTAIR |
| COXPD17 | KIAA1967 | | ARID1A | KLF6 |
| CRCS3 | L1CAM2 | | CEACAM5 | GSTP1 |
| CRCS4 | LAB | | BPIFA1 | CDKN2B |
| CUDR | LCA8 | | SYP | ARID1A |
| CXorf61 | LCGR | | CCNH | CEACAM5 |
| CYP2A | LPFS1 | | CCNB1 | BPIFA1 |
| DIRA | MASTC | | ENO2 | SYP |
| DPC4 | MCAP | | TMEM8B | CCNH |
| DRT | MCOPCB7 | | MIR107 | CCNB1 |
| EST | MCUL1 | | CASP9 | ENO2 |
| ESTRR | MGC29506 | | CHGA | TMEM8B |
| EVR7 | MRI | | CTNNA1 | MIR107 |
| FANCJ | NPCA3 | | MIR24-2 | CASP9 |
| FCC1 | NS6 | | PARP1 | CHGA |
| FILS | OSFD | | KRT8 | CTNNA1 |
| FPC | p90 | | GNAS | MIR24-2 |
| GASP | PARK18 | | KRT18 | PARP1 |
| GLM2 | PCH15 | | HLA-A | KRT8 |
| HGFAL | PDZK6 | | BAP1 | GNAS |
| HGPPS2 | RCC4 | | AFP | KRT18 |
| HNPCC2 | RHEX | | DLEC1 | HLA-A |
| HNPCC4 | RINGO1 | | MIR106A | BAP1 |
| HNPCC5 | RINGOA | | MAPK8 | AFP |
| HPC12 | RNX | | DLC1 | DLEC1 |
| HPRL | RPEM | | MYB | MIR106A |
| HRAD54 | SCL | | MIR193A | MAPK8 |
| IMPT1 | SMUCKLER | | ABCB1 | DLC1 |
| JDVS | SPTCL | | IGF1R | MYB |
| KIAA0203 | SYNCAM2 | | FGFR1 | MIR193A |
| KIAA1020 | TRAILR2 | | IL10 | ABCB1 |
| LCAM | TSLL1 | | FLT4 | IGF1R |
| LFS1 | TU3A | | XRCC2 | FGFR1 |
| LINC00913 | B7S1 | | MEG3 | IL10 |
| LKB1 | CHDTHP | | PHB1 | FLT4 |
| MCAP | CMNS | | MAPK1 | XRCC2 |
| MCUL1 | ECO | | NTRK3 | MEG3 |
| MDPL | FIH2 | | MIR150 | PHB1 |
| MKHK2 | FMRD | | IL2 | MAPK1 |
| MRMV2 | FPLD5 | | DNMT1 | NTRK3 |
| MVA1 | GPD | | CDKN2B-AS1 | MIR150 |
| NEU | KIAA0386 | | CXCL12 | IL2 |
| NKX2A | KIAA1375 | | PPM1D | DNMT1 |
| NMOR1 | KIAA1809 | | PVT1 | CDKN2B-AS1 |
| NMTC2 | LAN | | MIR130A | CXCL12 |
| NS6 | MCM | | ERCC1 | PPM1D |
| NSP2 | NECL1 | | TG | PVT1 |
| P16 | NECL3 | | LINC00312 | MIR130A |
| PAOD2 | NTAL | | MIR181A2 | ERCC1 |
| PARK18 | PHLS | | FLT1 | TG |
| PDJ | PKWS | | CYP1A1 | LINC00312 |
| PLA2L | PPBL | | ERBB3 | MIR181A2 |
| PNCA1 | RCC | | CYP2A6 | FLT1 |
| PNCA3 | SRTD20 | | BRD4 | CYP1A1 |
| PPP1R54 | BENTA | | CYP2E1 | ERBB3 |
| PRCA1 | DILOS | | MMP3 | CYP2A6 |
| PRLTS | DUH3 | | NFKB1 | BRD4 |
| RASK2 | EJM10 | | PDCD1 | CYP2E1 |
| RAY1 | MCMTC | | MIRLET7A1 | MMP3 |
| SCKL1 | NCMS | | HGF | NFKB1 |
| SRC1 | OFD17 | | SOX2 | PDCD1 |
| SSPCS | PL48 | | LGALS3 | MIRLET7A1 |
| TFM | SYNCAM3 | | TGFA | HGF |
| TITF2 | WBCQ1 | | CAV1 | SOX2 |
| TNRC14 | CMT2FF | | DNMT3B | LGALS3 |
| TOC | CWS5 | | MIR342 | TGFA |
| TROP1 | DFNB104 | | MIR206 | CAV1 |
| UC28 | IMD11A | | DAPK1 | DNMT3B |
| BAIPRCK | PSHK2 | | CYCS | MIR342 |
| BCC7 | CLAPO | | ABCC1 | MIR206 |
| BTAK | DFNA21 | | DICER1 | DAPK1 |
| BTPS2 | CCM4 | | CREBBP | CYCS |
| CDS1 | TP53 | | CYP19A1 | ABCC1 |
| CMNS | PTEN | | EZR | DICER1 |
| CSB | CDKN2A | | BMI1 | CREBBP |
| CWS1 | KRAS | | MIR30A | CYP19A1 |
| DUH1 | MIR21 | | SLC2A1 | EZR |
| DUP15q | HRAS | | TNFSF10 | BMI1 |
| ECAD | ERBB2 | | RHOA | MIR30A |
| ERK | CDH1 | | FN1 | SLC2A1 |
| FAM4A1 | AKT1 | | HLA-B | TNFSF10 |
| FANCR | CCND1 | | MIR215 | RHOA |
| FASL | ATM | | NEAT1 | FN1 |
| FCTCS | RB1 | | MMP7 | HLA-B |
| FMRD | MTOR | | HSPA4 | MIR215 |
| FSAP | FGFR3 | | BUB1B | NEAT1 |
| GLM3 | TERT | | STAT1 | MMP7 |
| HER2 | BRCA1 | | TLR2 | HSPA4 |
| HNPCC1 | BCL2 | | GAS5 | BUB1B |
| HPC1 | MIR205 | | MIR9-3 | STAT1 |
| IMAGEI | BRCA2 | | JAK2 | TLR2 |
| JIP | MYC | | ENG | GAS5 |
| JWS | STK11 | | MIR142 | MIR9-3 |
| M4S1 | PTPRC | | PLAU | JAK2 |
| MCM | VEGFA | | RAC1 | ENG |
| MFS2 | APC | | SPP1 | MIR142 |
| MLM | RET | | CCAT1 | PLAU |
| MMRCS1 | MIR34A | | SMAD2 | RAC1 |
| MMRCS3 | STAT3 | | TYMP | SPP1 |
| MMRCS4 | ALK | | RELA | CCAT1 |
| MOM1 | ING1 | | XIST | SMAD2 |
| NS | CDKN1A | | MAPK3 | TYMP |
| P450C2A | FASLG | | MIR16-1 | RELA |
| PNCA4 | PTCH1 | | RAF1 | XIST |
| SBMA | ADA | | MIR26A1 | MAPK3 |
| THC6 | LRRC56 | | BSG | MIR16-1 |
| TPL2 | MLH1 | | SOX9 | RAF1 |
| TTF1 | TGFBR2 | | MIR9-1 | MIR26A1 |
| TTF2 | MSH2 | | WRAP53 | BSG |
| ZF9 | CHEK2 | | ACTC1 | SOX9 |
| ALPS1B | SMAD4 | | IL7 | MIR9-1 |
| ARK1 | IL6 | | TYMS | WRAP53 |
| ARMD5 | FAS | | MAD1L1 | ACTC1 |
| BCDS1 | TGFB1 | | MIR320A | IL7 |
| BMFS5 | MIR145 | | PRKAR1A | TYMS |
| C15DUPq | MIR221 | | MMP14 | MAD1L1 |
| CFC2 | TNF | | RARB | MIR320A |
| CMM2 | MIR210 | | CCEPR | PRKAR1A |
| DESMD | CDKN1B | | MIR144 | MMP14 |
| FANCS | BAX | | GSTT1 | RARB |
| KD | TP63 | | MUC5AC | CCEPR |
| LDS2 | MIR17 | | MIR331 | MIR144 |
| LFS2 | FHIT | | E2F1 | GSTT1 |
| MCMTC | MAP2K1 | | MAP3K8 | MUC5AC |
| MIC18 | SETD2 | | CDX2 | MIR331 |
| MMRCS2 | RAG1 | | ETV6 | E2F1 |
| MYHRS | FGFR2 | | ETS1 | MAP3K8 |
| NCMS | MIR200C | | IFI27 | CDX2 |
| NMTC1 | EPCAM | | SP1 | ETV6 |
| NMTC4 | MIR143 | | CTTN | ETS1 |
| NMTC5 | MIR141 | | MIR138-1 | IFI27 |
| PCBC | ESR1 | | CCR7 | SP1 |
| PNCA2 | MIR222 | | PDPN | CTTN |
| TK14 | FLCN | | PDGFRA | MIR138-1 |
| VSCN2 | IFNG | | MGMT | CCR7 |
| BBDS | EGF | | AFAP1-AS1 | PDPN |
| CAPB | HNF1B | | TIMP2 | PDGFRA |
| CWS5 | PTGS2 | | CCR6 | MGMT |
| DIAR5 | IL7R | | TRIM24 | AFAP1-AS1 |
| RALD | CD44 | | CDH2 | TIMP2 |
| SMAX1 | MIR126 | | LDHA | CCR6 |
| STK6 | HIF1A | | PSMB9 | TRIM24 |
| UVSS1 | KRT5 | | CLDN7 | CDH2 |
| AIK | MMP9 | | TERC | LDHA |
| BDPLT22 | EP300 | | CASC9 | PSMB9 |
| CLAPO | NOTCH1 | | ZEB1 | CLDN7 |
| HNPCC8 | HNF1A | | CDK2 | TERC |
| HYSP1 | MIR98 | | SNAI1 | CASC9 |
| OES | MMP2 | | ABCC2 | ZEB1 |
| POF11 | MDM2 | | CDK6 | CDK2 |
| CCM4 | MSH6 | | MIR199A1 | SNAI1 |
| NBN | CASP3 | | HMGA2 | ABCC2 |
| RAD51L3-RFFL | PRKD1 | | SOX4 | CDK6 |
| C11orf65 | MIR155 | | LINC-ROR | MIR199A1 |
| RAD50 | MMP1 | | PRECSIT | HMGA2 |
| MIR21 | CXCL8 | | EPHA2 | SOX4 |
| MRE11 | CDK4 | | SERPINB5 | LINC-ROR |
| MIR34A | MAPK1 | | TGFBR1 | PRECSIT |
| RET | CXCR4 | | EIF4E | EPHA2 |
| MIR221 | CD274 | | KITLG | SERPINB5 |
| MIR17 | PPARG | | ALB | TGFBR1 |
| MIR200B | RAG2 | | MIR139 | EIF4E |
| MIR146A | SRC | | CD24 | KITLG |
| MIR222 | DDR2 | | BMP6 | ALB |
| MIR200C | IL10 | | ZFHX3 | MIR139 |
| MIR205 | MIR31 | | CADM1 | CD24 |
| MIR141 | NFE2L2 | | MCL1 | BMP6 |
| MIR125A | FBXW7 | | CYLD | ZFHX3 |
| MIR20A | KDR | | PTPN12 | CADM1 |
| MIR126 | IL2 | | FANCD2 | MCL1 |
| MIR200A | NBN | | PIGR | CYLD |
| FANCC | MIR200B | | B2M | PTPN12 |
| MIR155 | KRT14 | | ABCG2 | FANCD2 |
| XRCC2 | STAT1 | | ICAM1 | PIGR |
| MIR31 | TGFBR1 | | YAP1 | B2M |
| MIR27A | MIR20A | | PLAUR | ABCG2 |
| MIR127 | MITF | | HSP90AA1 | ICAM1 |
| FANCM | PCNA | | UBAP1 | YAP1 |
| MIR210 | MKI67 | | MIR338 | PLAUR |
| MET | MIR29A | | SNAI2 | HSP90AA1 |
| MIR10B | PALB2 | | SKP2 | UBAP1 |
| NF1 | FGFR1 | | POU5F1 | MIR338 |
| AOPEP | BIRC5 | | BLM | SNAI2 |
| LOC111589215 | KITLG | | NCAM1 | SKP2 |
| SLC22A18 | MIR146A | | IFNA2 | POU5F1 |
| ABRAXAS1 | MIR137 | | CCNA2 | BLM |
| MIR451A | AR | | ANXA2 | NCAM1 |
| MIR182 | MIR200A | | VEGFD | IFNA2 |
| KLLN | IL1B | | THBS1 | CCNA2 |
| MIR429 | MIR15A | | TYR | ANXA2 |
| MIR96 | MIR29C | | PDGFRB | VEGFD |
| MIR30E | ING3 | | CEACAM3 | THBS1 |
| MIR373 | JAK3 | | IL4 | TYR |
| MIR335 | PMS2 | | MAPK14 | PDGFRB |
| CTNNA1 | KRT19 | | CDK1 | CEACAM3 |
| MIR204 | MIR19A | | TKT | IL4 |
| CDC73 | SMARCA4 | | CDH3 | MAPK14 |
| MIR499A | JUN | | FOXM1 | CDK1 |
| MIR145 | MIR18A | | ITGB1 | TKT |
| MIR146B | ABCB1 | | CTNND1 | CDH3 |
| MIR206 | NFKBIA | | PTHLH | FOXM1 |
| MDM2 | NKX2-1 | | XIAP | ITGB1 |
| MIR199B | KRT7 | | IGF1 | CTNND1 |
| MIR196A2 | EGFR-AS1 | | XPA | PTHLH |
| MIR193B | TP73 | | TIMP1 | XIAP |
| BLM | NFKB1 | | MSMB | IGF1 |
| MIR128-1 | BCL2L1 | | XPC | XPA |
| TERT | CD4 | | CFLAR | TIMP1 |
| HERC2 | FGF2 | | CD40 | MSMB |
| PTCH1 | MIR203A | | CTLA4 | XPC |
| MIR520C | ERCC2 | | AXL | CFLAR |
| BSCL2 | ABL1 | | SOD2 | CD40 |
| MIR143 | VIM | | PKM | CTLA4 |
| MIR502 | MIR133B | | MIR423 | AXL |
| MIR9-3 | ICAM1 | | MMP13 | SOD2 |
| BAP1 | CDKN2B | | KRT13 | PKM |
| ALK | MIR373 | | KLF4 | MIR423 |
| GNG3 | IL7 | | SERPINA3 | MMP13 |
| HNRNPUL2-BSCL2 | MEN1 | | CRP | KRT13 |
| MIR661 | MIR100 | | MIR99A | KLF4 |
| BMPR1A | ITGB1 | | MIR212 | SERPINA3 |
| MIR510 | TSC1 | | PECAM1 | CRP |
| CDK4 | MIR34C | | S100A1 | MIR99A |
| CDKN1B | INS | | IDH2 | MIR212 |
| SMARCA4 | MIR125A | | MIR152 | PECAM1 |
| MIR203A | MIR30E | | FOS | S100A1 |
| MYC | TSC2 | | SPARC | IDH2 |
| KIT | RAC1 | | HNF1A-AS1 | MIR152 |
| MSH3 | MIR93 | | NAT2 | FOS |
| TSC1 | CTLA4 | | CTSD | SPARC |
| MIR34C | IGF1R | | AMACR | HNF1A-AS1 |
| PGR | PIK3R1 | | NOS2 | NAT2 |
| CYP19A1 | TGFA | | SMAD3 | CTSD |
| MAP2K1 | JAK2 | | IGFBP3 | AMACR |
| IGF2 | CREBBP | | GSK3B | NOS2 |
| MIR15A | WWOX | | HSPB1 | SMAD3 |
| BCL2 | IL4 | | NPTN-IT1 | IGFBP3 |
| TSC2 | RHOA | | MIR375 | GSK3B |
| VEGFA | CDK2 | | PRKCD | HSPB1 |
| MIR34B | CYP1A1 | | HSPA5 | NPTN-IT1 |
| PPARG | MIR34B | | POSTN | MIR375 |
| PIK3R1 | SOX2 | | TLR9 | PRKCD |
| SDHB | RELA | | CSF3 | HSPA5 |
| DICER1 | B2M | | THY1 | POSTN |
| MIRLET7D | PTPN11 | | MTA1 | TLR9 |
| STAT3 | GSTM1 | | IL17A | CSF3 |
| MAP3K1 | TFE3 | | CCL2 | THY1 |
| MUC1 | IGF2 | | WNT5A | MTA1 |
| MIRLET7C | MIR223 | | HLA-G | IL17A |
| CDKN1A | HLA-DRB1 | | MPP1 | CCL2 |
| ESR2 | NTRK1 | | MTHFR | WNT5A |
| MTOR | GSTP1 | | ELAVL1 | HLA-G |
| PMS1 | XRCC1 | | FOXO3 | MPP1 |
| MIR93 | FOXN1 | | MIR149 | MTHFR |
| IL6 | NCAM1 | | MIR196A1 | ELAVL1 |
| FGFR1 | MIR195 | | TLR4 | FOXO3 |
| MIR18A | MIR148A | | BMP2 | MIR149 |
| MIR106B | MIR27A | | CSF2 | MIR196A1 |
| FBXW7 | CEACAM5 | | CCND2 | TLR4 |
| ERCC2 | DICER1 | | ROCK1 | BMP2 |
| TGFB1 | DCC | | ITGB4 | CSF2 |
| TNF | MIRLET7D | | NANOG | CCND2 |
| MIR214 | ZAP70 | | HMGB1 | ROCK1 |
| MIR29C | SMARCB1 | | CCNE1 | ITGB4 |
| MEN1 | HGF | | GLI1 | NANOG |
| MIR223 | MIRLET7G | | ZEB2 | HMGB1 |
| EGF | TERC | | EIF4EBP1 | CCNE1 |
| TP63 | MIR193A | | PIK3CG | GLI1 |
| MIR19A | EZH2 | | ANXA5 | ZEB2 |
| MIR29A | NUTM1 | | MMP11 | EIF4EBP1 |
| MIRLET7G | BCL6 | | HPSE | PIK3CG |
| IGF1 | XIAP | | SFRP1 | ANXA5 |
| SDHD | PECAM1 | | NQO1 | MMP11 |
| LRRC56 | CXCL12 | | IL1RN | HPSE |
| ABCG2 | MIRLET7C | | ZBTB7A | SFRP1 |
| PTGS2 | CDK6 | | PRKCA | NQO1 |
| WWOX | ROS1 | | MIR449A | IL1RN |
| FANCD2 | MUTYH | | MAD2L1 | ZBTB7A |
| MMP2 | IL2RA | | FASN | PRKCA |
| HIF1A | CD40 | | NKILA | MIR449A |
| MSR1 | MIR296 | | NOTCH2 | MAD2L1 |
| RRAS2 | DLEC1 | | VDR | FASN |
| MMP9 | AURKA | | TGFB3 | NKILA |
| GSTM1 | PTK2 | | S100A4 | NOTCH2 |
| GSTP1 | MAPK8 | | KLRK1 | VDR |
| CD274 | IGF1 | | HDAC1 | TGFB3 |
| MAPK1 | MIR106B | | FOXP3 | S100A4 |
| CASP3 | CASP9 | | LOX | KLRK1 |
| ABCB1 | PGR | | TOP1 | HDAC1 |
| RUNX1 | PRKN | | PLK1 | FOXP3 |
| RBBP8 | PARP1 | | HLA-DRB1 | LOX |
| FAS | BRIP1 | | ENO1 | TOP1 |
| CXCR4 | MIR372 | | MIR29B1 | PLK1 |
| XRCC1 | ENG | | FOSL1 | HLA-DRB1 |
| PTPN11 | ERBB3 | | CDKN1C | ENO1 |
| MIR122 | RNF139 | | AKT3 | MIR29B1 |
| IGF1R | RAF1 | | FOXD2-AS1 | FOSL1 |
| MIR128-2 | SDHB | | LINC00460 | CDKN1C |
| TWIST1 | MGMT | | PTPRC | AKT3 |
| NOTCH1 | NPM1 | | DDR1 | FOXD2-AS1 |
| MKI67 | CCNA2 | | PROM1 | LINC00460 |
| MIR98 | IFNA1 | | FOXCUT | PTPRC |
| ERBB3 | FLT4 | | ANXA1 | DDR1 |
| MIR148A | CSF2 | | TNFRSF10A | PROM1 |
| CDKN3 | LAMC2 | | SSTR2 | FOXCUT |
| FHIT | ERCC6 | | TOP2A | ANXA1 |
| MIR25 | CAV1 | | BIRC3 | TNFRSF10A |
| MIR23B | MIR486-1 | | CSNK2A1 | SSTR2 |
| HNF1B | FN1 | | MTDH | TOP2A |
| MIRLET7A3 | SLC2A1 | |  | BIRC3 |
| GNAS | HLA-B | |  | CSNK2A1 |
| SOX9 | ESR2 | |  | MTDH |
| BIRC5 | MIR107 | |  |  |
| PDGFRA | MIR197 | |  |  |
| MIR183 | ENO2 | |  |  |
| IDH1 | KEAP1 | |  |  |
| PARP1 | CD34 | |  |  |
| EZH2 | FLT1 | |  |  |
| JAK2 | RAD51 | |  |  |
| SRD5A2 | H19 | |  |  |
| EGFR-AS1 | TLR2 | |  |  |
| VHL | MIR127 | |  |  |
| MIRLET7I | CA9 | |  |  |
| ERCC1 | KRT8 | |  |  |
| ROS1 | CCNB1 | |  |  |
| MTHFR | MIRLET7E | |  |  |
| MAP3K6 | CRNN | |  |  |
| MIR192 | FOXP3 | |  |  |
| CYP1A1 | FLT3 | |  |  |
| JUN | FANCC | |  |  |
| MIRLET7B | MIR106A | |  |  |
| MIR195 | PDPN | |  |  |
| CD44 | SPP1 | |  |  |
| MIR185 | PRKCD | |  |  |
| PLAU | CDKN3 | |  |  |
| TP73 | MAPK3 | |  |  |
| DROSHA | MIR204 | |  |  |
| MIR140 | HBB | |  |  |
| KLK3 | WT1 | |  |  |
| CTSD | KRT20 | |  |  |
| TOP2A | CCL2 | |  |  |
| TNFRSF10B | PDGFRA | |  |  |
| MIR16-1 | MMP14 | |  |  |
| H19 | PIK3CG | |  |  |
| IGFBP3 | SYP | |  |  |
| CXCL8 | POU5F1 | |  |  |
| TYMS | TNFSF10 | |  |  |
| IL10 | CSF3 | |  |  |
| SMARCB1 | VEGFC | |  |  |
| MIR107 | KRT18 | |  |  |
| MIR483 | CHGA | |  |  |
| NFKB1 | ERCC1 | |  |  |
| BCAR1 | MIR182 | |  |  |
| FANCE | HLA-A | |  |  |
| MIR486-1 | GNAS | |  |  |
| KDR | CDK1 | |  |  |
| BCL2L1 | PDGFRB | |  |  |
| CDK1 | SYK | |  |  |
| MIRLET7A1 | RARA | |  |  |
| KRT19 | CTTN | |  |  |
| RAF1 | XRCC3 | |  |  |
| TGFBR1 | OFD1 | |  |  |
| SMAD3 | TFEB | |  |  |
| TGFA | SMAD2 | |  |  |
| SOD2 | MIR30D | |  |  |
| CTLA4 | PLAU | |  |  |
| RIPK1 | MIR185 | |  |  |
| IGF2R | CDH2 | |  |  |
| CYP17A1 | TLR4 | |  |  |
| SDHC | MIR23B | |  |  |
| MIR181A1 | KRT13 | |  |  |
| MT-CYB | MALAT1 | |  |  |
| POLK | TYR | |  |  |
| ARID1A | HOTAIR | |  |  |
| POT1 | NME1 | |  |  |
| AXIN1 | CCR6 | |  |  |
| FGF2 | PAX8 | |  |  |
| MIR22 | TYMS | |  |  |
| PRKAR1A | COL7A1 | |  |  |
| MIR100 | SUFU | |  |  |
| MIR215 | BRD4 | |  |  |
| CASP9 | CYCS | |  |  |
| PDGFRB | RARB | |  |  |
| MMP1 | MAPK14 | |  |  |
| RASSF1 | TYMP | |  |  |
| MIR150 | RNF6 | |  |  |
| CEACAM5 | MME | |  |  |
| MMP14 | MIR183 | |  |  |
| OGG1 | TFRC | |  |  |
| CDK6 | RASSF1 | |  |  |
| ERBB4 | YAP1 | |  |  |
| CDK2 | SLC22A18 | |  |  |
| FZD3 | E2F1 | |  |  |
| TNFSF10 | ARID1A | |  |  |
| KRT7 | MIRLET7A3 | |  |  |
| MGMT | BAP1 | |  |  |
| VEGFC | DNMT1 | |  |  |
| CAV1 | ERBB4 | |  |  |
| CYP1B1 | NF1 | |  |  |
| SNAI2 | AKT2 | |  |  |
| SP1 | BSG | |  |  |
| ABCC1 | ABCG2 | |  |  |
| ING1 | MIRLET7B | |  |  |
| RHOA | MIR499A | |  |  |
| MIR137 | CD8A | |  |  |
| RELA | TIMP2 | |  |  |
| DNMT1 | ITGB4 | |  |  |
| E2F1 | MIR122 | |  |  |
| MIR130A | CYP2A6 | |  |  |
| MIR133B | CD40LG | |  |  |
| CXCL12 | MMP3 | |  |  |
| BCL10 | WRAP53 | |  |  |
| AKT2 | CCNH | |  |  |
| DDB2 | GJB2 | |  |  |
| PTK2 | POLE | |  |  |
| WRAP53 | ZEB1 | |  |  |
| CHEK1 | ALB | |  |  |
| IFNG | MIR214 | |  |  |
| ABL1 | SOX9 | |  |  |
| LEP | EPHA2 | |  |  |
| GREB1 | FOS | |  |  |
| ERCC4 | CLTC | |  |  |
| MIR10A | MIR10B | |  |  |
| MIRLET7E | MIR16-1 | |  |  |
| PCNA | DOCK8 | |  |  |
| FN1 | DNMT3B | |  |  |
| NAT2 | CD3D | |  |  |
| HOTAIR | PIK3CD | |  |  |
| MAPK3 | IKBKB | |  |  |
| CSF3 | SP1 | |  |  |
| VDR | MIR429 | |  |  |
| STAT1 | RAD51C | |  |  |
| IL2 | TMC8 | |  |  |
| FLT1 | PROM1 | |  |  |
| MAPK8 | IL1A | |  |  |
| SDHA | HSP90AA1 | |  |  |
| MIR15B | LMNA | |  |  |
| HGF | SHH | |  |  |
| BRMS1 | CTNNA1 | |  |  |
| MIR342 | AFP | |  |  |
| MALAT1 | MIR22 | |  |  |
| GLI1 | MRE11 | |  |  |
| MIR191 | XPA | |  |  |
| KRT5 | NOS2 | |  |  |
| CCNA2 | CD36 | |  |  |
| PLAUR | IDH1 | |  |  |
| XIAP | EPO | |  |  |
| NTHL1 | THBS1 | |  |  |
| WT1 | FANCD2 | |  |  |
| SPP1 | TWIST1 | |  |  |
| NRG1 | MIR130A | |  |  |
| TYMP | IDH2 | |  |  |
| PDCD1 | IL3 | |  |  |
| KRT18 | AXIN1 | |  |  |
| CYCS | FADD | |  |  |
| SNAI1 | KLF6 | |  |  |
| CTNNA2 | LGALS3 | |  |  |
| MIR372 | HNF4A | |  |  |
| CCNB1 | ANXA5 | |  |  |
| SUFU | MEG3 | |  |  |
| ZEB1 | CCR7 | |  |  |
| SMAD2 | BCL11B | |  |  |
| FLT4 | IGFBP3 | |  |  |
| PTHLH | GATA3 | |  |  |
| KRT8 | TIMP1 | |  |  |
| GPER1 | KRT4 | |  |  |
| HSP90AA1 | GATA1 | |  |  |
| CCNE1 | MIR150 | |  |  |
| NF2 | HLA-G | |  |  |
| CDKN2B | PLAUR | |  |  |
| MIR331 | BUB1B | |  |  |
| MEG3 | NONO | |  |  |
| DPYD | MIR144 | |  |  |
| RAD51B | DKC1 | |  |  |
| NTRK1 | NLRP1 | |  |  |
| BMP6 | DSG3 | |  |  |
| LCP1 | EIF4E | |  |  |
| MIR30D | MIR96 | |  |  |
| CCND2 | POLD1 | |  |  |
| ITGB1 | PTHLH | |  |  |
| SOX2 | SERPINE1 | |  |  |
| HSPB1 | MIRLET7A1 | |  |  |
| PLK1 | KRT17 | |  |  |
| GATA3 | CD247 | |  |  |
| MIR181A2 | KLRK1 | |  |  |
| CCL2 | TINF2 | |  |  |
| MIR106A | LOX | |  |  |
| BCAS1 | FOXE1 | |  |  |
| HDAC1 | CRP | |  |  |
| FOS | CCL5 | |  |  |
| MMP7 | PAX5 | |  |  |
| PRKCA | MAP3K8 | |  |  |
| MIR197 | MIR128-2 | |  |  |
| MCL1 | COL17A1 | |  |  |
| LOC109504725 | ETV6 | |  |  |
| AREG | CADM1 | |  |  |
| FGF8 | NDUFA13 | |  |  |
| PVT1 | GSTT1 | |  |  |
| INS | ERCC4 | |  |  |
| COMT | XPC | |  |  |
| MAPK14 | MMP7 | |  |  |
| HFE | IFI27 | |  |  |
| KRT20 | U2AF1 | |  |  |
| NME1 | CASP10 | |  |  |
| AKT3 | MIR10A | |  |  |
| SLC2A1 | RUNX1 | |  |  |
| RAC1 | PLK1 | |  |  |
| GSK3B | GLI1 | |  |  |
| TIMP1 | DPP4 | |  |  |
| THBS1 | CD28 | |  |  |
| VIM | NOTCH2 | |  |  |
| CSF2 | GJA1 | |  |  |
| SEC23B | MTHFR | |  |  |
| PRKACA | MIR483 | |  |  |
| IRS1 | BIRC3 | |  |  |
| PRL | MIR23A | |  |  |
| PIK3CG | ITGA6 | |  |  |
| RPS6KB1 | PRKCA | |  |  |
| TIMP3 | THY1 | |  |  |
| MIR26A1 | MIR140 | |  |  |
| RARB | MUC4 | |  |  |
| FASN | LEP | |  |  |
| PRKCD | NTRK3 | |  |  |
| TIMP2 | LAMB3 | |  |  |
| GNRH1 | BMP2 | |  |  |
| DNMT3B | TG | |  |  |
| SERPINA3 | VDR | |  |  |
| MXRA5 | MC1R | |  |  |
| AIP | GPC3 | |  |  |
| SERPINB5 | SERPINB3 | |  |  |
| CDH2 | CCND3 | |  |  |
| FOXP3 | BMI1 | |  |  |
| SERPINE1 | DNMT3A | |  |  |
| CDKN2B-AS1 | TET2 | |  |  |
| TCF7L2 | CCR5 | |  |  |
| PLK2 | MIR181A1 | |  |  |
| CYP2D6 | KLF4 | |  |  |
| CREB1 | GATA2 | |  |  |
| TOP1 | SNAI1 | |  |  |
| MTUS1 | MYCN | |  |  |
| GAS5 | HLA-DQB1 | |  |  |
| FOXO1 | MIR146B | |  |  |
| BMI1 | SMAD3 | |  |  |
| TGFB2 | CFLAR | |  |  |
| WRN | FGFR4 | |  |  |
| NFE2L2 | CREB1 | |  |  |
| MIR142 | MMP13 | |  |  |
| ETV6 | ABCC1 | |  |  |
| MIR181B1 | HMGB1 | |  |  |
| KLK10 | IFNA2 | |  |  |
| MT-CO1 | DPYD | |  |  |
| SKP2 | SNAI2 | |  |  |
| CD24 | ANXA1 | |  |  |
| HERPUD1 | CSF1 | |  |  |
| FOXA1 | MIR224 | |  |  |
| BCAS3 | F2 | |  |  |
| IFI27 | SST | |  |  |
| HSPA5 | RIPK1 | |  |  |
| BAK1 | HSPA4 | |  |  |
| CYP3A4 | INSR | |  |  |
| MIR30A | MIR25 | |  |  |
| PDGFB | CTNND1 | |  |  |
| CTAG1B | BMP6 | |  |  |
| RARA | SKP2 | |  |  |
| PAK1 | CLDN7 | |  |  |
| BRMS1L | JUP | |  |  |
| MIR449A | SDHD | |  |  |
| KRT14 | CAT | |  |  |
| PGBD3 | RHBDF2 | |  |  |
| INSR | CHEK1 | |  |  |
| SHBG | BLM | |  |  |
| FOXM1 | HMGA2 | |  |  |
| DMD | SOD2 | |  |  |
| KISS1 | CALCA | |  |  |
| ALDH1A1 | TUG1 | |  |  |
| POU5F1 | KDM4C | |  |  |
| TNFRSF10A | AREG | |  |  |
| MIR199A1 | NANOG | |  |  |
| GREM1 | IL17A | |  |  |
| DHFR | CDH3 | |  |  |
| FOXO3 | SOD1 | |  |  |
| IDH2 | PDGFB | |  |  |
| MTA1 | BMPR1A | |  |  |
| BCAS2 | LCK | |  |  |
| CCND3 | HSPB1 | |  |  |
| NFKBIA | EPHB2 | |  |  |
| PIP | MIR199A1 | |  |  |
| MIR23A | CD3G | |  |  |
| SHC1 | FANCM | |  |  |
| CTSB | FCGR3A | |  |  |
| H2AC18 | UCA1 | |  |  |
| CREBBP | ERCC3 | |  |  |
| IL4 | AXIN2 | |  |  |
| AGR3 | CEACAM1 | |  |  |
| AHR | CSF1R | |  |  |
| CCL5 | DSP | |  |  |
| SOS1 | CD3E | |  |  |
| ACTB | PVT1 | |  |  |
| MITF | FANCE | |  |  |
| MIR320A | XRCC2 | |  |  |
| BMP4 | RECQL4 | |  |  |
| GJA1 | GSK3B | |  |  |
| CA9 | CYP2E1 | |  |  |
| VEGFD | PRF1 | |  |  |
| HSPA4 | TRA | |  |  |
| NEAT1 | EZR | |  |  |
| BMP2 | DAPK1 | |  |  |
| MIR152 | MSR1 | |  |  |
| CASP7 | MIR191 | |  |  |
| TSG101 | DDB2 | |  |  |
| CTNND1 | NFKB2 | |  |  |
| CLDN7 | NEAT1 | |  |  |
| NRP1 | TMC6 | |  |  |
| HSD17B1 | ITGB2 | |  |  |
| TOX3 | MIR199B | |  |  |
| NOS2 | PDCD4 | |  |  |
| LEF1 | FERMT1 | |  |  |
| ITGA6 | IL15 | |  |  |
| SYP | MIR181A2 | |  |  |
| CDH3 | CALR | |  |  |
| TACC1 | GZMB | |  |  |
| YBX1 | TIMP3 | |  |  |
| TNFSF11 | CTSD | |  |  |
| SETD2 | LIG4 | |  |  |
| E2F3 | TOP1 | |  |  |
| AXL | TOP2A | |  |  |
| GSTT1 | CEACAM3 | |  |  |
| LZTS1 | CCND2 | |  |  |
| CCR6 | IVL | |  |  |
| HDAC9 | ITGAV | |  |  |
| DNMT3A | AXL | |  |  |
| MIR9-1 | SELE | |  |  |
| MYB | EDN1 | |  |  |
| LGALS3 | NF2 | |  |  |
| BID | KLK3 | |  |  |
| S100A4 | MCM4 | |  |  |
| JAK1 | RHOH | |  |  |
| SPARC | ACTA2 | |  |  |
| ETS1 | TCF7L2 | |  |  |
| NOTCH3 | CBL | |  |  |
| FGF1 | MUC16 | |  |  |
| KLF5 | HDAC1 | |  |  |
| BECN1 | TGFB2 | |  |  |
| NTRK3 | MIR375 | |  |  |
| TFAP2A | HMOX1 | |  |  |
| MUC16 | SERPINB5 | |  |  |
| RECQL | HLA-C | |  |  |
| ANKRD30A | PRKACA | |  |  |
| XPC | IL18 | |  |  |
| STAT5A | PTK2B | |  |  |
| BSG | CXCR3 | |  |  |
| MIR196B | PDCD1LG2 | |  |  |
| HNF1A | EWSR1 | |  |  |
| SLMAP | TYK2 | |  |  |
| HMGA2 | TEK | |  |  |
| TH2LCRR | RPS6KB1 | |  |  |
| BCL2L11 | ICOS | |  |  |
| GADD45A | MIR142 | |  |  |
| ABCC2 | IL1RN | |  |  |
| ENG | CYP19A1 | |  |  |
| MMP3 | SF3B1 | |  |  |
| LOX | MIR16-2 | |  |  |
| APEX1 | ITGB3 | |  |  |
| KCNQ1OT1 | GAS5 | |  |  |
| EPHA2 | LAMA3 | |  |  |
| EZR | MIR331 | |  |  |
| ICAM1 | HSPA5 | |  |  |
| PTK6 | PPP2R1A | |  |  |
| MIR224 | TRB | |  |  |
| ITGB3 | CCNE1 | |  |  |
| DKK1 | MIR424 | |  |  |
| WNT5A | PLEC | |  |  |
| ST14 | BAD | |  |  |
| AMER1 | ERCC5 | |  |  |
| MYCN | SERPINA3 | |  |  |
| GDF15 | ABCC2 | |  |  |
| CLU | WNT5A | |  |  |
| TERC | PIK3CB | |  |  |
| XPA | KRT10 | |  |  |
| SIRT1 | MYD88 | |  |  |
| IGFBP2 | NAT2 | |  |  |
| LEPR | GAST | |  |  |
| HMGB1 | HFE | |  |  |
| BAD | S100B | |  |  |
| STAT5B | MIR196A2 | |  |  |
| NOTCH2 | COL1A1 | |  |  |
| HDAC4 | CHUK | |  |  |
| ETV4 | CLU | |  |  |
| PKHD1 | HDAC9 | |  |  |
| CTAG2 | CD79A | |  |  |
| SRA1 | RUNX3 | |  |  |
| GAPDH | BMP4 | |  |  |
| ENO2 | VEGFD | |  |  |
| KLF4 | TNFSF11 | |  |  |
| XIST | POSTN | |  |  |
| STS | CXADR | |  |  |
| ELAVL1 | NSD1 | |  |  |
| MMP11 | CYLD | |  |  |
| GMNN | MIR139 | |  |  |
| CDC25A | PRKDC | |  |  |
| SOX4 | MAP2K2 | |  |  |
| CSF1 | IL6R | |  |  |
| HLA-G | ITGA4 | |  |  |
| ANXA2 | IRS1 | |  |  |
| CCNG1 | MIR149 | |  |  |
| CBFB | ANGPT2 | |  |  |
| ITGAV | CYP1B1 | |  |  |
| HBEGF | CYP2D6 | |  |  |
| PIK3R2 | STAT5B | |  |  |
| MIR375 | BIRC2 | |  |  |
| ECT2 | CDKN2B-AS1 | |  |  |
| XBP1 | EPHB4 | |  |  |
| ANXA1 | MPO | |  |  |
| WNT1 | BAK1 | |  |  |
| MTDH | MIR128-1 | |  |  |
| ERCC5 | MIR335 | |  |  |
| YAP1 | FSCN1 | |  |  |
| XRCC6 | IL2RB | |  |  |
| IL17A | CDX2 | |  |  |
| GPX1 | H2AC18 | |  |  |
| IGFBP1 | BCOR | |  |  |
| PDPN | PDGFRL | |  |  |
| NCOR1 | NQO1 | |  |  |
| CSF1R | DHFR | |  |  |
| XRCC5 | ETS1 | |  |  |
| MUCL1 | ANXA2 | |  |  |
| EREG | ICOSLG | |  |  |
| SF3B1 | CD80 | |  |  |
| PRKD1 | HMMR | |  |  |
| SMARCE1 | ADH1C | |  |  |
| MMP13 | CYP3A4 | |  |  |
| LOC107303340 | GAPDH | |  |  |
| FGF3 | FOXO1 | |  |  |
| FSCN1 | MSN | |  |  |
| TUG1 | EPAS1 | |  |  |
| FGF7 | CTSB | |  |  |
| MAP2K2 | NR3C1 | |  |  |
| LINC-ROR | GFAP | |  |  |
| FAN1 | SDC1 | |  |  |
| SPRY4-IT1 | TNFRSF10A | |  |  |
| NAT1 | JAG1 | |  |  |
| EGR1 | KRT1 | |  |  |
| KITLG | SOCS1 | |  |  |
| CCR7 | CTAG1B | |  |  |
| LDHA | KRT16 | |  |  |
| FGF17 | EIF4EBP1 | |  |  |
| TEK | PRL | |  |  |
| MIR149 | FOXM1 | |  |  |
| GRB2 | DLC1 | |  |  |
| TUBB | NGF | |  |  |
| PML | MTUS1 | |  |  |
| ENO1 | MVK | |  |  |
| CDC42 | MALT1 | |  |  |
| EPHB4 | NOD2 | |  |  |
| PIK3CB | ZEB2 | |  |  |
| EPSTI1 | FGF7 | |  |  |
| SULT1A1 | RUNX2 | |  |  |
| NR1H2 | JAK1 | |  |  |
| BDNF | SPRY4 | |  |  |
| CYP1A2 | TNFAIP3 | |  |  |
| ARID4B | BCL2L11 | |  |  |
| WNT3 | F3 | |  |  |
| LGALS1 | LDHA | |  |  |
| MVP | PXN | |  |  |
| MT-ND1 | TFAP2A | |  |  |
| PXN | PKM | |  |  |
| PTK2B | CDKN1C | |  |  |
| DRAIC | BUB1 | |  |  |
| STMN1 | ELAVL1 | |  |  |
| MAGED2 | NRP1 | |  |  |
| MAX | THBD | |  |  |
| CDC25C | FOXO3 | |  |  |
| PPP2R1A | STAT5A | |  |  |
| MIR125B1 | DCLRE1C | |  |  |
| ODC1 | BECN1 | |  |  |
| CCN2 | SQSTM1 | |  |  |
| LASP1 | EGR1 | |  |  |
| EIF4E | EPOR | |  |  |
| IL2RA | CD24 | |  |  |
| CLCA2 | IL5 | |  |  |
| SPDEF | AKT3 | |  |  |
| IL3 | HLA-DPB1 | |  |  |
| CTCF | SASH1 | |  |  |
| MYO1B | SIRT1 | |  |  |
| PLCG1 | CCAT1 | |  |  |
| FADD | MIR9-1 | |  |  |
| AURKB | SOX4 | |  |  |
| SCGB2A2 | KCNJ11 | |  |  |
| MIR24-2 | WRN | |  |  |
| DKC1 | TNFRSF1A | |  |  |
| NR3C1 | MLH3 | |  |  |
| RAD52 | BGLAP | |  |  |
| NFKB2 | HABP2 | |  |  |
| RUNX2 | DES | |  |  |
| RECQL4 | SOS1 | |  |  |
| REST | NRG1 | |  |  |
| HMGA1 | SHC1 | |  |  |
| ID1 | CDC6 | |  |  |
| FGF4 | IL6ST | |  |  |
| ATAD2 | ACE | |  |  |
| ABCC11 | SLC4A1 | |  |  |
| MME | PLCG1 | |  |  |
| PIK3CD | SPARC | |  |  |
| CHUK | IKBKG | |  |  |
| NCOA1 | GRP | |  |  |
| ITGA3 | MIR30A | |  |  |
| E2F2 | SDHC | |  |  |
| AMPH | FANCA | |  |  |
| CSNK2A1 | RPS27 | |  |  |
| NPM1 | ACTB | |  |  |
| PTTG1 | MIR99A | |  |  |
| HDAC2 | ATR | |  |  |
| TH2-LCR | ALDH1A1 | |  |  |
| CHGA | BLNK | |  |  |
| SYK | PDX1 | |  |  |
| BAG1 | VWF | |  |  |
| RHOB | MIR196A1 | |  |  |
| TCIM | CEACAM6 | |  |  |
| PROM1 | XRCC5 | |  |  |
| ERCC3 | MIR26A1 | |  |  |
| WNT10B | GRHL2 | |  |  |
| H2AX | DDR1 | |  |  |
| KDM5B | PPM1D | |  |  |
| RBFOX1 | ELANE | |  |  |
| CD34 | ACTC1 | |  |  |
| PSCA | MIR193B | |  |  |
| SOCS3 | IL13 | |  |  |
| LRP6 | DCUN1D1 | |  |  |
| NCOA6 | SF3B2 | |  |  |
| RELB | XPO1 | |  |  |
| ALB | MIR192 | |  |  |
| ZFAS1 | REL | |  |  |
| HOTTIP | HLA-DQA1 | |  |  |
| EPHA3 | RNASEL | |  |  |
| FOXC1 | TJP1 | |  |  |
| CEBPB | CD86 | |  |  |
| NCOR2 | IDO1 | |  |  |
| TBX3 | LYN | |  |  |
| TXN | CDKN2C | |  |  |
| MIR29B2 | ALDH2 | |  |  |
| BRCA3 | TGFB3 | |  |  |
| LCN2 | FOSL1 | |  |  |
| JAG1 | FGF1 | |  |  |
| EMSY | NOTCH3 | |  |  |
| PTENP1 | CALB2 | |  |  |
| TRIM24 | GREM1 | |  |  |
| TCERG1 | ITGA5 | |  |  |
| BLTP2 | NGFR | |  |  |
| ROBO1 | RXRA | |  |  |
| GRN | DDX3X | |  |  |
| SPINK1 | LGALS1 | |  |  |
| RRAS | S100A4 | |  |  |
| ITGB4 | NES | |  |  |
| TMEM71 | LIF | |  |  |
| PIN1 | YY1 | |  |  |
| IRS2 | RECK | |  |  |
| HMOX1 | SELP | |  |  |
| ARAF | KMT2D | |  |  |
| PRMT7 | ITGAL | |  |  |
| IGFBP5 | LEF1 | |  |  |
| TIMELESS | TLR3 | |  |  |
| RHOBTB2 | SFN | |  |  |
| TGFB3 | APOE | |  |  |
| CDK7 | MTDH | |  |  |
| ALCAM | PML | |  |  |
| PIK3R3 | FYN | |  |  |
| CYP24A1 | MIR342 | |  |  |
| OLA1 | NFIB | |  |  |
| HEATR6 | NTRK2 | |  |  |
| SPHK1 | L1CAM | |  |  |
| CASC22 | SERPINB4 | |  |  |
| MIR16-2 | MIF | |  |  |
| APOD | RPL5 | |  |  |
| MAPK10 | CFTR | |  |  |
| KIF15 | CCAT2 | |  |  |
| CEMIP | NECTIN1 | |  |  |
| PTPN3 | HPSE | |  |  |
| INS-IGF2 | MCM2 | |  |  |
| ST18 | CXCL10 | |  |  |
| BCYRN1 | AHR | |  |  |
| CCAR2 | RTEL1 | |  |  |
| DANCR | SMARCE1 | |  |  |
| CUL1 | KCNN4 | |  |  |
| UHRF1 | HSPA8 | |  |  |
| CSNK1A1 | MIR206 | |  |  |
| ESRRA | CCN2 | |  |  |
| CEP85L | PMS1 | |  |  |
| AKR1C3 | MAGEA3 | |  |  |
| RHOC | THPO | |  |  |
| PELP1 | HBEGF | |  |  |
| ROCK1 | GRB2 | |  |  |
| DDR1 | KLRD1 | |  |  |
| F2R | CYTOR | |  |  |
| HSPA8 | GALC | |  |  |
| S100A6 | CDH5 | |  |  |
| AGR2 | MIR320A | |  |  |
| BTK | ITGA3 | |  |  |
| TFAP2C | FANCG | |  |  |
| GNRHR | TRAF3 | |  |  |
| NGFR | IL4R | |  |  |
| WNT3A | BDNF | |  |  |
| ELF3 | ITGA2 | |  |  |
| MIR193A | XRCC6 | |  |  |
| DIRC3 | MAX | |  |  |
| CCN1 | IFNB1 | |  |  |
| FANCA | CP | |  |  |
| RINT1 | RBBP8 | |  |  |
| TLR4 | F5 | |  |  |
| MIR181C | MDM4 | |  |  |
| LSP1 | TCF3 | |  |  |
| BIRC3 | APEX1 | |  |  |
| SSTR2 | RPS19 | |  |  |
| ATRX | PALLD | |  |  |
| MST1R | IL21 | |  |  |
| BEX2 | ROCK1 | |  |  |
| TPD52 | TLR9 | |  |  |
| PTPRG | ENO1 | |  |  |
| CFLAR | PTGER4 | |  |  |
| CYTOR | PLA2G2A | |  |  |
| DUSP1 | NLRP3 | |  |  |
| PSEN2 | GJB6 | |  |  |
| CASP2 | RARS1 | |  |  |
| MYLK | PRKCB | |  |  |
| CCNH | S100A7 | |  |  |
| CRP | CD68 | |  |  |
| MDM4 | S100A1 | |  |  |
| EIF3H | ILK | |  |  |
| HNRNPK | FOXP1 | |  |  |
| CCNE2 | TNFRSF1B | |  |  |
| EPOR | FASN | |  |  |
| TNFRSF11B | CASP7 | |  |  |
| HDAC6 | CD9 | |  |  |
| NGF | MS4A1 | |  |  |
| MACROD1 | KNSTRN | |  |  |
| UGT1A1 | MAPK10 | |  |  |
| S100A2 | TCF4 | |  |  |
| WEE1 | POMC | |  |  |
| FBLN1 | CEACAM7 | |  |  |
| MIR497 | ANPEP | |  |  |
| MIR101-1 | STIM1 | |  |  |
| MC1R | CD151 | |  |  |
| BPIFA4P | CD81 | |  |  |
| BMPR2 | MIR9-3 | |  |  |
| TNFRSF11A | SELL | |  |  |
| MIAT | MIR15B | |  |  |
| ERGIC3 | IL11 | |  |  |
| ATF1 | CPLANE1 | |  |  |
| HMGCR | RPSA | |  |  |
| WNT6 | CR2 | |  |  |
| MCM2 | YWHAE | |  |  |
| SNHG16 | RRAS2 | |  |  |
| RAD21 | RHEB | |  |  |
| LMNA | TTN | |  |  |
| LRP5 | SFRP1 | |  |  |
| KDM4B | GLI3 | |  |  |
| NOTCH4 | APP | |  |  |
| MUC5AC | CD55 | |  |  |
| FBN1 | PSMB9 | |  |  |
| CDKN2C | STMN1 | |  |  |
| MAPK7 | POLK | |  |  |
| MED12 | G6PD | |  |  |
| CDX2 | CASC9 | |  |  |
| AFP | GLI2 | |  |  |
| NTRK2 | TRIM24 | |  |  |
| NCOA2 | SPRY4-IT1 | |  |  |
| FOSL1 | PRKCI | |  |  |
| IBSP | KCNQ1OT1 | |  |  |
| GHRH | FAT1 | |  |  |
| ZEB2 | FGF4 | |  |  |
| LYVE1 | CCL3 | |  |  |
| CDKN1C | TXN | |  |  |
| WWTR1 | MUC5AC | |  |  |
| SOX2-OT | CTSC | |  |  |
| GH1 | MIR138-1 | |  |  |
| PDGFA | CD82 | |  |  |
| DVL1 | TRAF6 | |  |  |
| SULT1E1 | CD5 | |  |  |
| IL6ST | KIR3DL1 | |  |  |
| SCGB1D2 | RACK1 | |  |  |
| MT-CO2 | MMP11 | |  |  |
| MAP2K5 | NUMA1 | |  |  |
| MAP2K3 | PVR | |  |  |
| IL11 | ALPP | |  |  |
| ELK1 | SERPINA1 | |  |  |
| TP53BP1 | RRM2B | |  |  |
| MINPP1 | LAT | |  |  |
| CCDC170 | POLH | |  |  |
| EPAS1 | FLNA | |  |  |
| SMAD6 | MLANA | |  |  |
| SKP1 | BID | |  |  |
| PRDM14 | TNFRSF8 | |  |  |
| CRNN | MCAM | |  |  |
| GRB7 | MICA | |  |  |
| IDO1 | CXCR2 | |  |  |
| FANCG | ZBTB16 | |  |  |
| BRCATA | MIR215 | |  |  |
| TCF7L1 | CCR1 | |  |  |
| YY1 | SREBF1 | |  |  |
| CYP2E1 | TNC | |  |  |
| RNF6 | LEPR | |  |  |
| PKM | ANGPT1 | |  |  |
| MAPK12 | ADIPOQ | |  |  |
| PRDM2 | ODC1 | |  |  |
| PANDAR | POT1 | |  |  |
| TCF7 | ABCB11 | |  |  |
| ANXA5 | KLRC1 | |  |  |
| WNT2 | ADAM17 | |  |  |
| HOXA11-AS | AGER | |  |  |
| FLT3 | SLC19A1 | |  |  |
| AKAP13 | CYP1A2 | |  |  |
| MUC4 | TF | |  |  |
| USF3 | WEE1 | |  |  |
| BMP7 | CARMIL2 | |  |  |
| NET1 | CEBPA | |  |  |
| MIR196A1 | CYP2C19 | |  |  |
| CDK12 | MAGEA1 | |  |  |
| PIAS1 | STK4 | |  |  |
| CRNDE | CDH13 | |  |  |
| TRIM28 | FGF10 | |  |  |
| PPARA | GRN | |  |  |
| MAPK9 | CTSK | |  |  |
| CAGE1 | SLC7A5 | |  |  |
| CXCL10 | FLG | |  |  |
| AKIP1 | SOCS3 | |  |  |
| TG | RPS20 | |  |  |
| LIMK1 | DKK1 | |  |  |
| CSNK1D | TUBB | |  |  |
| PTP4A1 | SMARCAD1 | |  |  |
| ERG | CSNK2A1 | |  |  |
| FANCI | ASCL1 | |  |  |
| IGFBP6 | CUL3 | |  |  |
| CSNK2B | ADAR | |  |  |
| CBL | MSLN | |  |  |
| MAD2L1 | MIR29B1 | |  |  |
| GRP | FBN1 | |  |  |
| MIR29B1 | TCL1A | |  |  |
| SFN | FDPS | |  |  |
| HLA-A | PARD3 | |  |  |
| EFNA1 | AMACR | |  |  |
| NOVA1 | SOX10 | |  |  |
| RUNX3 | TKT | |  |  |
| LRATD2 | PTPN1 | |  |  |
| TDGF1 | CD47 | |  |  |
| NEK2 | CD59 | |  |  |
| ECM1 | KRT15 | |  |  |
| FZD7 | TLR7 | |  |  |
| BCL6 | FANCL | |  |  |
| SLC9A3R1 | COL14A1 | |  |  |
| RAB25 | AURKB | |  |  |
| DLL4 | GCG | |  |  |
| MIR424 | GDF15 | |  |  |
| MIR31HG | UCHL1 | |  |  |
| LIFR | PSEN1 | |  |  |
| XPO1 | MIRLET7I | |  |  |
| TRIM37 | MAGEA4 | |  |  |
| ANGPT2 | HERC2 | |  |  |
| HLA-DQB1 | PDGFA | |  |  |
| CALR | SLC2A2 | |  |  |
| SEMA3A | PIK3R2 | |  |  |
| GPC3 | NAPSA | |  |  |
| IFI16 | KMT2A | |  |  |
| S100A14 | ID1 | |  |  |
| NCAM1 | ITGAM | |  |  |
| IL7 | H2AX | |  |  |
| ZMYND8 | GADD45A | |  |  |
| NRP2 | PRKAA1 | |  |  |
| RBBP4 | WNT1 | |  |  |
| AFAP1 | CCL4 | |  |  |
| POLQ | RPL11 | |  |  |
| CCN4 | MIR181B1 | |  |  |
| CASC9 | BIRC7 | |  |  |
| HDAC5 | MSMB | |  |  |
| PYHIN1 | TFF1 | |  |  |
| MAP2K4 | PTPN22 | |  |  |
| RBX1 | ALOX12 | |  |  |
| TPM3 | IRF4 | |  |  |
| S100A7 | EDNRB | |  |  |
| FGF10 | NEUROD1 | |  |  |
| LNCRNA-ATB | LTA | |  |  |
| ENSG00000215409 | PTPN12 | |  |  |
| ENSG00000230087 | GSN | |  |  |
| PPP2R2A | XIST | |  |  |
| PRKD3 | SNHG1 | |  |  |
| WNT7B | MTA1 | |  |  |
| PCAT6 | FANCF | |  |  |
| NR5A1 | FGF3 | |  |  |
| EDNRA | IGFBP2 | |  |  |
| ACTN4 | PSENEN | |  |  |
| SHH | ATRX | |  |  |
| EWSR1 | PKP1 | |  |  |
| LIMD1 | CDC27 | |  |  |
| UIMC1 | F2R | |  |  |
| MIR139 | NDRG1 | |  |  |
| SUSD2 | SMAD7 | |  |  |
| PAX8 | ALOX5 | |  |  |
| BMP10 | NEU1 | |  |  |
| ZNF365 | RAP1A | |  |  |
| STIM1 | ADAM10 | |  |  |
| NR3C2 | CASP1 | |  |  |
| CDC27 | PTPN6 | |  |  |
| SDHAF2 | GATA4 | |  |  |
| TPR | IL12RB1 | |  |  |
| NORAD | MIR181C | |  |  |
| PTPA | COMT | |  |  |
| LINC00511 | POLR1C | |  |  |
| GATA2 | PLG | |  |  |
| BLID | MIR30B | |  |  |
| SGO1 | VTCN1 | |  |  |
| CSNK2A2 | CASR | |  |  |
| EBAG9 | HOPX | |  |  |
| ITGB3BP | HDAC2 | |  |  |
| THBS4 | WAS | |  |  |
| S100A8 | PAK1 | |  |  |
| NT5E | MAPK9 | |  |  |
| RALA | DST | |  |  |
| STARD13 | CD1A | |  |  |
| PAX5 | ARNT | |  |  |
| ABCA1 | LYVE1 | |  |  |
| KDM4C | PGF | |  |  |
| ELOC | ASXL1 | |  |  |
| CDC25B | MMP10 | |  |  |
| SPRY4 | PRDX1 | |  |  |
| ZMIZ1 | CLDN1 | |  |  |
| TET2 | AIFM1 | |  |  |
| PES1 | MAP2K4 | |  |  |
| IKBKE | ATP2A2 | |  |  |
| LOXL2 | PAX6 | |  |  |
| ZFP36 | CSF3R | |  |  |
| MIR328 | BMP7 | |  |  |
| MIR296 | CCNL1 | |  |  |
| SNHG15 | ORAI1 | |  |  |
| HIPK2 | DSG1 | |  |  |
| FANCF | HOTTIP | |  |  |
| FZD8 | PAX2 | |  |  |
| MSLN | CEBPB | |  |  |
| LOC106721785 | NR1H2 | |  |  |
| COL1A1 | SHBG | |  |  |
| SLC39A1 | PDPK1 | |  |  |
| TP53COR1 | PTGS1 | |  |  |
| PTPRC | MAL | |  |  |
| PLEKHA8 | KLRB1 | |  |  |
| FAM3C | RHOC | |  |  |
| CSK | NT5E | |  |  |
| SAFB | PLAT | |  |  |
| DLL1 | FUS | |  |  |
| ENAH | RELB | |  |  |
| SLC39A6 | BST2 | |  |  |
| BACH1 | ARID1B | |  |  |
| ARHGEF2 | APAF1 | |  |  |
| LOC107303338 | HSPD1 | |  |  |
| CALML4 | TNFRSF11A | |  |  |
| TPM1 | LRBA | |  |  |
| CD28 | CD58 | |  |  |
| ABI1 | C2CD3 | |  |  |
| SQSTM1 | PPARA | |  |  |
| SF3B2 | AAGAB | |  |  |
| LSM1 | ELN | |  |  |
| FOLH1 | KRT6A | |  |  |
| P3H2 | GFI1 | |  |  |
| FGF19 | ANAPC1 | |  |  |
| SST | ANO1 | |  |  |
| WNT11 | MT-CO1 | |  |  |
| CST6 | NAMPT | |  |  |
| CDC6 | CD99 | |  |  |
| PTN | FAP | |  |  |
| DVL2 | ITGA2B | |  |  |
| PRMT2 | AQP1 | |  |  |
| HES1 | NR5A1 | |  |  |
| DAPK1 | YBX1 | |  |  |
| CD82 | MVP | |  |  |
| NR1I2 | VTN | |  |  |
| SMO | S100A2 | |  |  |
| DUSP3 | PSMB8 | |  |  |
| TRIP13 | CDC25B | |  |  |
| CERNA2 | ST14 | |  |  |
| KMT2D | SPHK1 | |  |  |
| EGOT | WNT10A | |  |  |
| PPP1R14C | VCP | |  |  |
| TNFRSF1B | PIEZO1 | |  |  |
| RYBP | ARAF | |  |  |
| PRKDC | ALDOA | |  |  |
| INPPL1 | HSPG2 | |  |  |
| ARFGEF3 | GBA | |  |  |
| PSMB5 | BCAM | |  |  |
| MIR202 | CD38 | |  |  |
| PRKCQ | P2RX7 | |  |  |
| CD36 | PRKCQ | |  |  |
| KSR1 | PTPN13 | |  |  |
| MIR124-3 | GGT1 | |  |  |
| CFTR | PRKCE | |  |  |
| JUP | KLF5 | |  |  |
| ESRRG | KRT6B | |  |  |
| KLK13 | CDK5 | |  |  |
| FGF6 | TDGF1 | |  |  |
| WNT5B | SMPD1 | |  |  |
| CHD7 | DEFB1 | |  |  |
| ROR2 | MIR132 | |  |  |
| MAP2K6 | MAML2 | |  |  |
| GPC6 | LUCAT1 | |  |  |
| B2M | CD69 | |  |  |
| FGF9 | TJP2 | |  |  |
| UGDH | NECTIN4 | |  |  |
| EXO1 | TGIF1 | |  |  |
| ANKRD17 | CCR4 | |  |  |
| PECAM1 | IRF6 | |  |  |
| EIF4EBP1 | PIP | |  |  |
| ROCK2 | GSTM3 | |  |  |
| NNT-AS1 | TSHR | |  |  |
| JAG2 | CCKBR | |  |  |
| SRRM3 | OCA2 | |  |  |
| FZD1 | FGF8 | |  |  |
| BCDIN3D | PCAT1 | |  |  |
| CKS1B | TNFRSF11B | |  |  |
| ADIPOQ | DMD | |  |  |
| HPR | TPX2 | |  |  |
| IGFBP4 | TIAM1 | |  |  |
| CA2 | CXCL14 | |  |  |
| CLOCK | DAXX | |  |  |
| SNHG7 | KNG1 | |  |  |
| SMAD1 | LBR | |  |  |
| ZNF703 | XBP1 | |  |  |
| PFN1 | CD244 | |  |  |
| KLF17 | HNRNPK | |  |  |
| SLX4 | RPS24 | |  |  |
| SPHK2 | PNP | |  |  |
| HOXA-AS2 | ADAM12 | |  |  |
| LINC00472 | RBX1 | |  |  |
| MRC2 | CIITA | |  |  |
| PSMD6 | TGM1 | |  |  |
| POSTN | GNRH1 | |  |  |
| PRSS50 | TGM2 | |  |  |
| IFNA2 | CSNK2B | |  |  |
| RPS20 | VCL | |  |  |
| SFRP1 | RPS6 | |  |  |
| ELF5 | MICB | |  |  |
| GRM1 | STAT4 | |  |  |
| FSHR | PTTG1 | |  |  |
| NDRG1 | GPT | |  |  |
| GPATCH2 | ACTN4 | |  |  |
| MIR32 | CD46 | |  |  |
| HULC | SSTR2 | |  |  |
| FZR1 | TUBB3 | |  |  |
| HSPB8 | PIM1 | |  |  |
| ILK | CDH23 | |  |  |
| NUS1 | BAG1 | |  |  |
| CUL3 | MYO1B | |  |  |
| KLK6 | ROR2 | |  |  |
| FZD6 | FZR1 | |  |  |
| PSMA1 | MIR338 | |  |  |
| PDLIM2 | F2RL1 | |  |  |
| PEA15 | SRD5A2 | |  |  |
| MCM7 | LRP6 | |  |  |
| RHNO1 | EDNRA | |  |  |
| NSD1 | ABCC3 | |  |  |
| CCNB2 | XRCC4 | |  |  |
| KEAP1 | GNAQ | |  |  |
| HLA-B | SMARCA2 | |  |  |
| CDH13 | FOLH1 | |  |  |
| U2AF1 | SRSF2 | |  |  |
| ST8SIA1 | CCK | |  |  |
| TOE1 | TH | |  |  |
| CLDN4 | HDAC6 | |  |  |
| MATK | UBE3A | |  |  |
| GSE1 | MIR324 | |  |  |
| MIR99A | LINC-ROR | |  |  |
| RAP1A | IRS2 | |  |  |
| SOX10 | ROBO1 | |  |  |
| VWA5A | CTSL | |  |  |
| NBAT1 | E2F2 | |  |  |
| MORC2 | DSE | |  |  |
| NFIB | TRAF2 | |  |  |
| WASF3 | ACHE | |  |  |
| CUEDC2 | SPI1 | |  |  |
| HIF1A-AS2 | LTF | |  |  |
| POMC | DDX59 | |  |  |
| MED1 | PTPN3 | |  |  |
| PCAT7 | S100A8 | |  |  |
| FOSL2 | MYCL | |  |  |
| RPS6KB2 | TAP1 | |  |  |
| MIR181B2 | APOA1 | |  |  |
| TP73-AS1 | DDIT3 | |  |  |
| HPGD | PARN | |  |  |
| CALCA | CD63 | |  |  |
| CADM1 | CXCR1 | |  |  |
| ADH1C | CD22 | |  |  |
| NKILA | USB1 | |  |  |
| APOE | HSF1 | |  |  |
| ACTA2 | HBG2 | |  |  |
| IL1A | PLA2G4A | |  |  |
| FGF5 | RPL18 | |  |  |
| CD40 | MIR101-1 | |  |  |
| NANOG | CYP17A1 | |  |  |
| PPP1R1B | AGO2 | |  |  |
| ARRDC3 | WNT3 | |  |  |
| LOC110806263 | PRTN3 | |  |  |
| EDN1 | PPARGC1A | |  |  |
| RECK | LTO1 | |  |  |
| LIG4 | ABCB4 | |  |  |
| MMP17 | PAX3 | |  |  |
| WDR11 | PLA2G6 | |  |  |
| CSN2 | CXCL1 | |  |  |
| CT45A10 | CD14 | |  |  |
| PBXIP1 | RPS10 | |  |  |
| RRM2 | S100A9 | |  |  |
| SRY | HIRA | |  |  |
| FZD5 | ADRB2 | |  |  |
| TRERF1 | MIR494 | |  |  |
| CTAG1A | IL1R1 | |  |  |
| GPR161 | EPHX1 | |  |  |
| FABP4 | GLUL | |  |  |
| KLC1 | GHRL | |  |  |
| PIGR | MIR186 | |  |  |
| ABCC12 | CASC2 | |  |  |
| AREL1 | E2F3 | |  |  |
| LIN28B | MBP | |  |  |
| STRADA | MYOG | |  |  |
| KDM1A | BTC | |  |  |
| NR0B1 | MYH9 | |  |  |
| TCF4 | CCR3 | |  |  |
| TFPI | KIR2DL1 | |  |  |
| WT1-AS | PODXL | |  |  |
| MAGEA1 | PERP | |  |  |
| NOS3 | HMGA1 | |  |  |
| WWC1 | C4A | |  |  |
| APOBEC3G | CXCR5 | |  |  |
| USP15 | SET | |  |  |
| FGF21 | CORO1A | |  |  |
| HOXA5 | PRDM1 | |  |  |
| CYP17A1-AS1 | NUP214 | |  |  |
| HPSE | DLL1 | |  |  |
| RABL6 | MIR365A | |  |  |
| TLR9 | DUSP1 | |  |  |
| LHCGR | GATA6 | |  |  |
| TPX2 | UGT1A1 | |  |  |
| FABP3 | ACP3 | |  |  |
| MIRLET7F1 | SLPI | |  |  |
| TAB1 | VRK1 | |  |  |
| MT-TT | TACSTD2 | |  |  |
| RANBP9 | SPN | |  |  |
| PTGS1 | REN | |  |  |
| TPM2 | CLDN4 | |  |  |
| SAFB2 | HSPA1A | |  |  |
| SEPTIN1 | MAP3K7 | |  |  |
| SELENOP | RPL15 | |  |  |
| GAST | MIR211 | |  |  |
| BIRC2 | SALL4 | |  |  |
| ALDOA | EGLN1 | |  |  |
| IFNA1 | NNMT | |  |  |
| SERPINA1 | INHA | |  |  |
| MIR423 | IGF2BP2 | |  |  |
| IRAIN | CXCL9 | |  |  |
| FGF13 | KIF7 | |  |  |
| KDM3B | VIP | |  |  |
| PRSS1 | SH2B3 | |  |  |
| PHLDA2 | PAX1 | |  |  |
| CDH5 | DAB2 | |  |  |
| MTAP | TRD | |  |  |
| MAGEA4 | PSAP | |  |  |
| MAGEA3 | CCN1 | |  |  |
| MIR532 | WNT3A | |  |  |
| SCGB3A1 | EIF2AK2 | |  |  |
| MIR663A | NCOA3 | |  |  |
| CETN3 | TBK1 | |  |  |
| CCKBR | H3C11 | |  |  |
| ETV1 | CRYAB | |  |  |
| HLA-DRB1 | RABL3 | |  |  |
| TPD52L2 | DIABLO | |  |  |
| CXCR2 | TLR5 | |  |  |
| NOP16 | LDLR | |  |  |
| RNF146 | GHR | |  |  |
| PRC1 | WIF1 | |  |  |
| CLPTM1L | MVD | |  |  |
| IKBKB | MIR27B | |  |  |
| DDR2 | TRPV4 | |  |  |
| CXCL1 | CYP26A1 | |  |  |
| APPBP2 | LIN28B | |  |  |
| POLH | SLC2A4 | |  |  |
| LMTK3 | REST | |  |  |
| DIABLO | FUT4 | |  |  |
| DLEU1 | HES1 | |  |  |
| MAPK6 | TIA1 | |  |  |
| LYPD3 | SLX4 | |  |  |
| FAP | RRM1 | |  |  |
| TKT | MIR32 | |  |  |
| EIF3A | FOXC1 | |  |  |
| AIF1L | IL9 | |  |  |
| MNX1-AS1 | CSNK1A1 | |  |  |
| KISS1R | LAMA5 | |  |  |
| SVEP1 | ACTG1 | |  |  |
| GLI2 | SLC3A2 | |  |  |
| UBE2T | MIR148B | |  |  |
| TYR | ATP7B | |  |  |
| BANP | MUC2 | |  |  |
| BCR | PARK7 | |  |  |
| CD4 | TRPV3 | |  |  |
| RASGRP3 | DCK | |  |  |
| HBP1 | EML4 | |  |  |
| PPP5C | MIR374A | |  |  |
| SEMA4A | PFKFB3 | |  |  |
| EPB41L4A-DT | MTHFD1 | |  |  |
| CASC16 | RPA1 | |  |  |
| CERK | ALOX15B | |  |  |
| MACC1 | GLB1 | |  |  |
| KLK9 | LAG3 | |  |  |
| ADAM11 | CCNG1 | |  |  |
| KIAA1671 | TBX2 | |  |  |
| COX7A2L | KCNQ1 | |  |  |
| FAM83D | AGTR1 | |  |  |
| DUSP6 | FCGR2A | |  |  |
| PCID2 | MIR340 | |  |  |
| MCM4 | CDK2AP1 | |  |  |
| FGF14 | GPER1 | |  |  |
| CITED4 | SNCA | |  |  |
| GREB1L | MYLK | |  |  |
| PLK3 | TBX21 | |  |  |
| RNF11 | BCAR1 | |  |  |
| MUC2 | PICALM | |  |  |
| TRADD | GPX1 | |  |  |
| SETBP1 | HP | |  |  |
| NBR2 | SLC17A9 | |  |  |
| FANCB | CDC16 | |  |  |
| PLA2G10 | PIK3C2A | |  |  |
| PROX1 | MAP2 | |  |  |
| EPHX1 | CRABP2 | |  |  |
| CCDC26 | LATS1 | |  |  |
| PDCD4 | SLC16A1 | |  |  |
| RERG | C3 | |  |  |
| SMC4 | VAV1 | |  |  |
| LINC00052 | FLI1 | |  |  |
| ITGA5 | MIR125B1 | |  |  |
| LINP1 | CDK7 | |  |  |
| PAXIP1 | CRH | |  |  |
| DSCAM-AS1 | MECOM | |  |  |
| MIR30B | DCN | |  |  |
| MIF | STAT6 | |  |  |
| RLIM | RHOB | |  |  |
| TPD52L1 | LY6D | |  |  |
| F3 | MAD2L1 | |  |  |
| MIR144 | PTH | |  |  |
| PAWR | IRF2BP2 | |  |  |
| ALOX5 | HK2 | |  |  |
| MT-TE | CGA | |  |  |
| MAPK4 | RRM2 | |  |  |
| HP | SEMA4A | |  |  |
| GNA11 | SLC17A5 | |  |  |
| HEYL | P2RY1 | |  |  |
| MIR24-1 | ROCK2 | |  |  |
| NOD2 | NOTCH4 | |  |  |
| CASR | VANGL1 | |  |  |
| MTR | HMGCR | |  |  |
| FANCL | CSK | |  |  |
| WNT4 | ACP1 | |  |  |
| ACTC1 | MUC3A | |  |  |
| MIR124-1 | CR1 | |  |  |
| LINC01116 | CDC37 | |  |  |
| CEACAM3 | CUL2 | |  |  |
| TCHP | NFATC3 | |  |  |
| MUC6 | LCN2 | |  |  |
| CCNT2 | CHD7 | |  |  |
| LINC01234 | ANG | |  |  |
| TUNAR | MYH11 | |  |  |
| MIR136 | NTS | |  |  |
| AZGP1 | ATP7A | |  |  |
| IL13 | ACVRL1 | |  |  |
| SRD5A1 | SERPINC1 | |  |  |
| LINC00628 | AICDA | |  |  |
| APAF1 | HNRNPA1 | |  |  |
| AFAP1-AS1 | RPL27 | |  |  |
| PSMC3IP | DMBT1 | |  |  |
| HSD17B3 | DVL1 | |  |  |
| GSN | NR1H4 | |  |  |
| MIR181D | CRTC1 | |  |  |
| MYD88 | MYBL1 | |  |  |
| PLG | OSM | |  |  |
| GATA4 | PVALB | |  |  |
| MAGEC2 | PKHD1 | |  |  |
| GHR | MB | |  |  |
| RMST | TUBA1A | |  |  |
| PDPK1 | GSDMB | |  |  |
| NTPCR | MIR370 | |  |  |
| IKBKG | SOX11 | |  |  |
| DPP10-AS1 | IGF2BP3 | |  |  |
| CAT | EREG | |  |  |
| HMMR-AS1 | MED12 | |  |  |
| WIF1 | PIN1 | |  |  |
| DELEC1 | MIR184 | |  |  |
| KLK4 | TMPRSS6 | |  |  |
| CHD1 | HOXA11-AS | |  |  |
| MIR92A1 | DSC3 | |  |  |
| DSP | CKS1B | |  |  |
| CIB1 | MIR130B | |  |  |
| GNAQ | CTCF | |  |  |
| DPH1 | NHP2 | |  |  |
| LINC00901 | CDK9 | |  |  |
| NES | MEFV | |  |  |
| ZNF667-AS1 | RBL2 | |  |  |
| GATA1 | DSG2 | |  |  |
| PSG2 | FAT2 | |  |  |
| TUSC3 | ACKR3 | |  |  |
| TTK | ID2 | |  |  |
| PEBP1 | LGALS7 | |  |  |
| MIR27B | SERPINF1 | |  |  |
| CALB2 | MIR26B | |  |  |
| SULF1 | CDC45 | |  |  |
| PHOX2B | AIRE | |  |  |
| TNFRSF1A | NHEJ1 | |  |  |
| FOXC2-AS1 | MTAP | |  |  |
| SLC30A2 | LYST | |  |  |
| MAGI2-AS3 | CMA1 | |  |  |
| IL24 | CCNA1 | |  |  |
| OR3A4P | DTNBP1 | |  |  |
| ITGA2 | DEFB4A | |  |  |
| THPO | KDM5B | |  |  |
| COL18A1 | HLA-E | |  |  |
| FEZF1 | ERG | |  |  |
| TGM2 | HDAC4 | |  |  |
| MIRLET7F2 | HSP90B1 | |  |  |
| LINC00958 | HCK | |  |  |
| TRPS1 | FOXL2 | |  |  |
| TWIST2 | NCOR1 | |  |  |
| CLDND1 | MAL2 | |  |  |
| CT45A1 | GNAI2 | |  |  |
| MIR132 | USP8 | |  |  |
| ERVH48-1 | CYP11A1 | |  |  |
| LINC00993 | RMRP | |  |  |
| E2F4 | BACH2 | |  |  |
| MIR324 | MAPK7 | |  |  |
| IGF2BP3 | HLA-DPA1 | |  |  |
| XRCC4 | LOXL2 | |  |  |
| DMBT1 | PAX7 | |  |  |
| TMPRSS2 | SCRIB | |  |  |
| HLA-DQA1 | TFF3 | |  |  |
| LINC00574 | MIR502 | |  |  |
| SNHG1 | IL24 | |  |  |
| SSX2 | GNA11 | |  |  |
| PRKAA1 | MAGT1 | |  |  |
| EXT2 | FABP4 | |  |  |
| LINC00160 | TOM1 | |  |  |
| CT47A3 | MMP12 | |  |  |
| VTN | PEBP1 | |  |  |
| DAB2IP | SPINT2 | |  |  |
| TJP1 | CCAR1 | |  |  |
| MIR338 | DANCR | |  |  |
| EFNA3 | HLA-DRA | |  |  |
| RACK1 | NPY | |  |  |
| ING3 | MYOD1 | |  |  |
| MANCR | E2F4 | |  |  |
| IL6R | FOLR1 | |  |  |
| CT47A1 | MAP3K5 | |  |  |
| CT47A2 | CCR2 | |  |  |
| CT47A4 | ATF1 | |  |  |
| SERPINB2 | MADCAM1 | |  |  |
| SFTPA1 | CD226 | |  |  |
| GPR68 | MIR24-1 | |  |  |
| IL4R | RAB27A | |  |  |
| INHA | CXCL13 | |  |  |
| AMACR | MIR330 | |  |  |
| CLDN3 | PFN1 | |  |  |
| IL18 | MIR423 | |  |  |
| RTEL1 | PTPA | |  |  |
| PBRM1 | FGA | |  |  |
| PTGER4 | LIMS1 | |  |  |
| DCK | IBSP | |  |  |
| CD9 | TMPRSS2 | |  |  |
| STAT6 | CD83 | |  |  |
| WNT2B | CSTA | |  |  |
| MIR370 | NCSTN | |  |  |
| FOXCUT | PHLDA1 | |  |  |
| MIR2052HG | KDM1A | |  |  |
| HAVCR2 | ADAM9 | |  |  |
| CXCL14 | IGFBP6 | |  |  |
| LINC00339 | MDK | |  |  |
| MT-ND4L | BCYRN1 | |  |  |
| LINC01016 | ARID2 | |  |  |
| LCAL1 | PPP6C | |  |  |
| GNB1 | PRNP | |  |  |
| MELK | TFPI2 | |  |  |
| APC2 | RPL31 | |  |  |
| AGER | BCL3 | |  |  |
| YWHAE | INHBA | |  |  |
| ANGPT1 | DNTT | |  |  |
| LINC00520 | RPS27A | |  |  |
| HK2 | HNF1A-AS1 | |  |  |
| SLC19A1 | TARDBP | |  |  |
| SUZ12 | TTR | |  |  |
| BGLAP | FCGR2B | |  |  |
| WFDC2 | FOXA1 | |  |  |
| TFRC | RBL1 | |  |  |
| FGF14-AS2 | IGFBP1 | |  |  |
| CDH17 | MIR212 | |  |  |
| LINC01671 | MUC5B | |  |  |
| RRM1 | FURIN | |  |  |
| ACP3 | JUNB | |  |  |
| SLC5A8 | CCL20 | |  |  |
| LINC00598 | TAPBP | |  |  |
| LINC01089 | STXBP2 | |  |  |
| LINC02099 | CNR1 | |  |  |
| NUMA1 | SNHG16 | |  |  |
| ACE | B3GAT1 | |  |  |
| REV3L | SFTPC | |  |  |
| ICOSLG | LLGL1 | |  |  |
| MPL | MYO18B | |  |  |
| BNIP3 | ALPG | |  |  |
| CT47A12 | MTR | |  |  |
| IRF4 | PTPN2 | |  |  |
| PON1 | CASK | |  |  |
| KLRK1 | PTK7 | |  |  |
| TNFRSF10D | MCM7 | |  |  |
| CIP2A | NCOR2 | |  |  |
| NCOA4 | EPIST | |  |  |
| PTPN13 | SELENBP1 | |  |  |
| MCM3 | TPM3 | |  |  |
| PEG10 | COL4A1 | |  |  |
| BRD4 | DLK1 | |  |  |
| GRPR | DDX58 | |  |  |
| POLR1C | UROS | |  |  |
| LAMC2 | UNC13D | |  |  |
| NTS | HPRT1 | |  |  |
| ST8SIA6-AS1 | MIR532 | |  |  |
| ABCC3 | ELF3 | |  |  |
| WNT7A | TAP2 | |  |  |
| XAGE1B | FCER2 | |  |  |
| MYCL | SCGB1A1 | |  |  |
| BRINP1 | CYP2C9 | |  |  |
| TMEFF2 | MAPK8IP1 | |  |  |
| FLI1 | LOC110806263 | |  |  |
| RMRP | KDM5C | |  |  |
| ASXL1 | S100A6 | |  |  |
| SAT1 | IRAK1 | |  |  |
| GACAT1 | PLIN2 | |  |  |
| CD8A | TK1 | |  |  |
| MAD2L2 | DLG1 | |  |  |
| RASSF1-AS1 | DRD2 | |  |  |
| COL7A1 | WNT7A | |  |  |
| S100A9 | TP53BP1 | |  |  |
| PRKCE | ADM | |  |  |
| SOCS1 | TNFRSF10D | |  |  |
| ATF3 | CCL11 | |  |  |
| MEFV | MIR124-1 | |  |  |
| PTPRG-AS1 | GP5 | |  |  |
| CD40LG | NRP2 | |  |  |
| DPP4 | TOR1A | |  |  |
| SELENBP1 | TINCR | |  |  |
| SELE | MT-ND1 | |  |  |
| HSD3B2 | TLR8 | |  |  |
| CDH23 | LAMA1 | |  |  |
| CEACAM6 | LAMC1 | |  |  |
| GSTM3 | RPS7 | |  |  |
| FUT3 | LGR5 | |  |  |
| HSPD1 | MECP2 | |  |  |
| ADAM12 | PMAIP1 | |  |  |
| XAGE1A | CLDN3 | |  |  |
| KCNK15-AS1 | EIF2AK3 | |  |  |
| PNPLA6 | RICTOR | |  |  |
| STXBP5-AS1 | WWTR1 | |  |  |
| IGKV@ | IGF2BP1 | |  |  |
| NAMPT | LGALS9 | |  |  |
| PMAIP1 | NOP10 | |  |  |
| GZMB | CA12 | |  |  |
| TNFRSF10C | HELLS | |  |  |
| TNFRSF8 | TNFRSF10C | |  |  |
| RASGRP1 | MEIS1 | |  |  |
| CD79A | UPK3A | |  |  |
| MIR361 | CTC1 | |  |  |
| RXRA | NSD2 | |  |  |
| LZTR1 | UMPS | |  |  |
| NECTIN4 | WNT4 | |  |  |
| KDM6A | LOXL4 | |  |  |
| RAD23B | ITGB6 | |  |  |
| IL15 | CYP3A5 | |  |  |
| ACD | PRKCZ | |  |  |
| KLK2 | IFNGR1 | |  |  |
| ALPP | CFL1 | |  |  |
| UCHL1 | PAK4 | |  |  |
| GHET1 | PROX1 | |  |  |
| SDC1 | SGK1 | |  |  |
| SLC5A5 | LPP | |  |  |
| LINC00636 | LAMA2 | |  |  |
| TFF3 | GUSB | |  |  |
| PTPN1 | H4-16 | |  |  |
| MIR376A1 | SOX17 | |  |  |
| PINK1-AS | ATF3 | |  |  |
| LINC02130 | SIRPA | |  |  |
| PDE11A | MIR590 | |  |  |
| EPO | CACNA1C | |  |  |
| CEBPA | RHOD | |  |  |
| G6PD | PGK1 | |  |  |
| STK4 | SLC2A3 | |  |  |
| UGT1A9 | NR1I2 | |  |  |
| ADARB2-AS1 | DVL2 | |  |  |
| STUB1 | CRNDE | |  |  |
| HAGLR | F13A1 | |  |  |
| LINC01405 | PRKACB | |  |  |
| NDUFA13 | CCNE2 | |  |  |
| PRKCI | HNRNPH1 | |  |  |
| KLF3-AS1 | CD163 | |  |  |
| PDCD4-AS1 | PIGR | |  |  |
| PKD1P6-NPIPP1 | KISS1 | |  |  |
| KMT2A | STN1 | |  |  |
| CCNA1 | BTRC | |  |  |
| EED | COPS5 | |  |  |
| DLK1 | HTR2A | |  |  |
| HSF1 | CD200 | |  |  |
| HPN | DNAH8 | |  |  |
| COL14A1 | ETV4 | |  |  |
| NUP214 | MDC1 | |  |  |
| MIR377 | SKP1 | |  |  |
| URI1 | TRAF4 | |  |  |
| CDT1 | MERTK | |  |  |
| CYP3A5 | PPP1CB | |  |  |
| ALOX12 | WNT10B | |  |  |
| CYP27B1 | SLAMF1 | |  |  |
| PAX3 | PLAG1 | |  |  |
| HNF4A | TNFRSF4 | |  |  |
| FRGCA | TBL1XR1 | |  |  |
| MIR212 | CLPTM1L | |  |  |
| ANTXR1 | ACD | |  |  |
| CCR5 | ARPC1B | |  |  |
| CTSK | MUC6 | |  |  |
| BRCD1 | IL23A | |  |  |
| S100P | DPF2 | |  |  |
| TSHR | CRKL | |  |  |
| TRB | SEMA4D | |  |  |
| MIR330 | MIR328 | |  |  |
| JADRR | KAT5 | |  |  |
| SOD1 | FAM168A | |  |  |
| PCM1 | PSG2 | |  |  |
| FSHB | PRPF8 | |  |  |
| SMARCA2 | SFRP2 | |  |  |
| H3-3A | DDX5 | |  |  |
| EEF1A1 | KIR2DS1 | |  |  |
| BUB3 | MCM3 | |  |  |
| EFNB1 | KDM6A | |  |  |
| HLA-C | ICAM2 | |  |  |
| BBC3 | PMEL | |  |  |
| TRAF6 | ENPP2 | |  |  |
| RPL5 | AP3B1 | |  |  |
| MIR489 | SPTLC1 | |  |  |
| CYP2C19 | IQGAP1 | |  |  |
| S100B | SRA1 | |  |  |
| DES | RHO | |  |  |
| MUC5B | FOXA2 | |  |  |
| CXCR5 | IGHE | |  |  |
| CTBP1 | PAX9 | |  |  |
| PLAG1 | BBC3 | |  |  |
| SIX1 | JUND | |  |  |
| PRMT1 | FLNB | |  |  |
| KLK7 | PON1 | |  |  |
| TGIF1 | CCL17 | |  |  |
| MST1 | COL4A2 | |  |  |
| CDC45 | BANCR | |  |  |
| MIR455 | LORICRIN | |  |  |
| SPINT2 | IGFBP5 | |  |  |
| KLK15 | CD109 | |  |  |
| LIF | HPGD | |  |  |
| RHEB | IL18R1 | |  |  |
| VWF | SMN1 | |  |  |
| SEPTIN9 | ITGAE | |  |  |
| F2 | CDCA5 | |  |  |
| SLC45A3 | CTPS1 | |  |  |
| BANCR | POLG | |  |  |
| PROKR2 | CAPRIN1 | |  |  |
| CT47A6 | NUMB | |  |  |
| BRDT | TEC | |  |  |
| CXCR1 | TP53BP2 | |  |  |
| PTH | HBA2 | |  |  |
| VTCN1 | LAMA4 | |  |  |
| WNT10A | NKILA | |  |  |
| HNF1A-AS1 | SFTPA1 | |  |  |
| LTA | CUL1 | |  |  |
| CCL20 | NOX4 | |  |  |
| TINCR | TRIP13 | |  |  |
| ARID1B | SETDB1 | |  |  |
| DDX5 | PRKCH | |  |  |
| MDK | IL21R | |  |  |
| CDCP1 | ZFP36 | |  |  |
| CT47A7 | PRMT5 | |  |  |
| AMH | TARS1 | |  |  |
| RCVRN | NCOA1 | |  |  |
| JAK3 | IFIH1 | |  |  |
| EIF2AK2 | TFDP1 | |  |  |
| ANPEP | COL18A1 | |  |  |
| MIR378A | ZFAS1 | |  |  |
| MMP12 | DNM1L | |  |  |
| SRARP | IKBKE | |  |  |
| LAMB3 | RHAG | |  |  |
| GJB2 | PPIA | |  |  |
| FENDRR | DSC2 | |  |  |
| BIRC7 | IL10RA | |  |  |
| LDOC1 | RPTOR | |  |  |
| VIP | RASSF5 | |  |  |
| DNAJC21 | NR4A2 | |  |  |
| SPIN1 | PAWR | |  |  |
| TRPM8 | SNHG12 | |  |  |
| NDNF | KIR2DS4 | |  |  |
| ANXA3 | ELMO2 | |  |  |
| RETN | IRF3 | |  |  |
| MIR186 | HNRNPA2B1 | |  |  |
| MYOD1 | SCARB1 | |  |  |
| MECOM | MBL2 | |  |  |
| SATB1 | ECM1 | |  |  |
| LINC00673 | AHI1 | |  |  |
| TLR3 | EPHA4 | |  |  |
| SLC29A1 | PFAS | |  |  |
| LTF | RAP1B | |  |  |
| SPINT1 | CPOX | |  |  |
| FST | CLEC7A | |  |  |
| AMHR2 | PDK1 | |  |  |
| TK1 | STING1 | |  |  |
| CHI3L1 | GCH1 | |  |  |
| KRT17 | TGFBR3 | |  |  |
| AAGAB | ACTA1 | |  |  |
| CXCR3 | COL4A3 | |  |  |
| PROK2 | YWHAZ | |  |  |
| MIR345 | SAA1 | |  |  |
| SREBF1 | SIX1 | |  |  |
| TINF2 | SATB2 | |  |  |
| LGALS3BP | RETN | |  |  |
| VAV3 | ELMO3 | |  |  |
| GLI3 | IAPP | |  |  |
| PIM1 | DNASE1 | |  |  |
| MLLT10 | SMARCAL1 | |  |  |
| MIR133A1 | RECQL | |  |  |
| CT47A5 | BMPR2 | |  |  |
| CD80 | CALD1 | |  |  |
| MIEN1 | KL | |  |  |
| MIR129-1 | FUZ | |  |  |
| IFNB1 | MAD2L2 | |  |  |
| SOX3 | OPRM1 | |  |  |
| SFRP4 | VDAC1 | |  |  |
| MIR211 | FCGR3B | |  |  |
| SIRT3 | CRK | |  |  |
| CEP57 | GHRH | |  |  |
| NKX3-1 | BNIP3 | |  |  |
| CBR3-AS1 | EBAG9 | |  |  |
| DKK3 | ACE2 | |  |  |
| VCAM1 | SLC29A1 | |  |  |
| MIR92A2 | PPARD | |  |  |
|  | CARD9 | |  |  |
|  | FES | |  |  |
|  | IL23R | |  |  |
|  | ASAH1 | |  |  |
|  | PLA2G1B | |  |  |
|  | SIRT3 | |  |  |
|  | GP6 | |  |  |
|  | EPB41L3 | |  |  |
|  | CA2 | |  |  |
|  | CANX | |  |  |
|  | PTK6 | |  |  |
|  | GSR | |  |  |
|  | KRT2 | |  |  |
|  | CHI3L1 | |  |  |
|  | KMT2C | |  |  |
|  | MIA2 | |  |  |
|  | RUVBL1 | |  |  |
|  | CCL22 | |  |  |
|  | CDH17 | |  |  |
|  | ELP1 | |  |  |
|  | CCEPR | |  |  |
|  | CSMD1 | |  |  |
|  | HNRNPU | |  |  |
|  | ARG1 | |  |  |
|  | LRP1 | |  |  |
|  | RAN | |  |  |
|  | XDH | |  |  |
|  | FOXD2-AS1 | |  |  |
|  | BRD2 | |  |  |
|  | CYP27B1 | |  |  |
|  | NCOA2 | |  |  |
|  | SCN5A | |  |  |
|  | KCNJ5 | |  |  |
|  | ABCC4 | |  |  |
|  | ACTN1 | |  |  |
|  | RDX | |  |  |
|  | CGB5 | |  |  |
|  | ADAMTSL1 | |  |  |
|  | SEMA3F | |  |  |
|  | NR0B1 | |  |  |
|  | TAGLN | |  |  |
|  | AGT | |  |  |
|  | SAMD9 | |  |  |
|  | RALA | |  |  |
|  | TNFRSF6B | |  |  |
|  | RXRB | |  |  |
|  | DYRK1A | |  |  |
|  | DAG1 | |  |  |
|  | S1PR1 | |  |  |
|  | TRIM28 | |  |  |
|  | UBC | |  |  |
|  | FPGS | |  |  |
|  | FOXCUT | |  |  |
|  | CSNK2A2 | |  |  |
|  | CTSG | |  |  |
|  | RPS14 | |  |  |
|  | NCR3 | |  |  |
|  | IFNAR1 | |  |  |
|  | TPBG | |  |  |
|  | CFH | |  |  |
|  | MIR20B | |  |  |
|  | IFI16 | |  |  |
|  | IFNAR2 | |  |  |
|  | LRIG1 | |  |  |
|  | IL33 | |  |  |
|  | AHCY | |  |  |
|  | MIR302A | |  |  |
|  | GNRHR | |  |  |
|  | DDB1 | |  |  |
|  | NORAD | |  |  |
|  | PRDX2 | |  |  |
|  | KCNMA1 | |  |  |
|  | SULF1 | |  |  |
|  | PKD1 | |  |  |
|  | TAC1 | |  |  |
|  | HSD17B1 | |  |  |
|  | HBA1 | |  |  |
|  | NR3C2 | |  |  |
|  | MPLKIP | |  |  |
|  | TERF1 | |  |  |
|  | IFITM1 | |  |  |
|  | MIR455 | |  |  |
|  | ADCY10 | |  |  |
|  | FSHR | |  |  |
|  | SATB1 | |  |  |
|  | G6PC1 | |  |  |
|  | ELAVL4 | |  |  |
|  | AQP3 | |  |  |
|  | HDGF | |  |  |
|  | EXO1 | |  |  |
|  | TPI1 | |  |  |
|  | SUMO1 | |  |  |
|  | MIR138-2 | |  |  |
|  | TGFBI | |  |  |
|  | TRPM7 | |  |  |
|  | ELK1 | |  |  |
|  | POLB | |  |  |
|  | RPL6 | |  |  |
|  | PRLR | |  |  |
|  | FGF9 | |  |  |
|  | DELEC1 | |  |  |
|  | ANGPTL4 | |  |  |
|  | SAG | |  |  |
|  | RPS15 | |  |  |
|  | NOG | |  |  |
|  | RAD52 | |  |  |
|  | HSP90AB1 | |  |  |
|  | RFX5 | |  |  |
|  | HSPA9 | |  |  |
|  | HYAL1 | |  |  |
|  | DVL3 | |  |  |
|  | AMFR | |  |  |
|  | SMC1A | |  |  |
|  | RIT1 | |  |  |
|  | LIMD1 | |  |  |
|  | NFAT5 | |  |  |
|  | FEN1 | |  |  |
|  | CD207 | |  |  |
|  | PPP2CA | |  |  |
|  | SLC40A1 | |  |  |
|  | HLA-DQA2 | |  |  |
|  | GMNN | |  |  |
|  | SRD5A1 | |  |  |
|  | SLC9A3R1 | |  |  |
|  | CDH11 | |  |  |
|  | MAP3K14 | |  |  |
|  | TPD52 | |  |  |
|  | DDX41 | |  |  |
|  | PTENP1 | |  |  |
|  | TSR2 | |  |  |
|  | SERPINB13 | |  |  |
|  | CAMP | |  |  |
|  | MIR199A2 | |  |  |
|  | FECH | |  |  |
|  | CRABP1 | |  |  |
|  | LRP1B | |  |  |
|  | ECRG4 | |  |  |
|  | TLE1 | |  |  |
|  | CCN4 | |  |  |
|  | SULT1A1 | |  |  |
|  | AKR1B10 | |  |  |
|  | RBPJ | |  |  |
|  | CACNA1G | |  |  |
|  | LASP1 | |  |  |
|  | PRAME | |  |  |
|  | FANCI | |  |  |
|  | DACT1 | |  |  |
|  | MIR133A1 | |  |  |
|  | CCL18 | |  |  |
|  | LINC01133 | |  |  |
|  | PAK2 | |  |  |
|  | NCR1 | |  |  |
|  | TYRP1 | |  |  |
|  | GRIN2B | |  |  |
|  | JAG2 | |  |  |
|  | POLR2A | |  |  |
|  | PSTPIP1 | |  |  |
|  | ICAM3 | |  |  |
|  | DNAJC21 | |  |  |
|  | MIR17HG | |  |  |
|  | ETS2 | |  |  |
|  | PCLAF | |  |  |
|  | GPNMB | |  |  |
|  | GTF2H5 | |  |  |
|  | OXT | |  |  |
|  | PSEN2 | |  |  |
|  | ATXN2 | |  |  |
|  | PRKAA2 | |  |  |
|  | LCP1 | |  |  |
|  | SMAD6 | |  |  |
|  | NME2 | |  |  |
|  | MIR326 | |  |  |
|  | HYOU1 | |  |  |
|  | OCLN | |  |  |
|  | RNF43 | |  |  |
|  | GAL | |  |  |
|  | IL3RA | |  |  |
|  | IL13RA2 | |  |  |
|  | WNT5B | |  |  |
|  | COL11A1 | |  |  |
|  | KIR3DS1 | |  |  |
|  | GAS1 | |  |  |
|  | RAD21 | |  |  |
|  | RIPK4 | |  |  |
|  | RAB11A | |  |  |
|  | ARF6 | |  |  |
|  | CDH15 | |  |  |
|  | HPS6 | |  |  |
|  | HDAC3 | |  |  |
|  | PIK3C3 | |  |  |
|  | MIR196B | |  |  |
|  | AQP2 | |  |  |
|  | FLT3LG | |  |  |
|  | HK1 | |  |  |
|  | ABCA4 | |  |  |
|  | FGFBP1 | |  |  |
|  | SLC12A2 | |  |  |
|  | THRB | |  |  |
|  | DNM2 | |  |  |
|  | STC2 | |  |  |
|  | AKR1C1 | |  |  |
|  | CTHRC1 | |  |  |
|  | IL32 | |  |  |
|  | PTH1R | |  |  |
|  | MIR4435-2HG | |  |  |
|  | FERMT2 | |  |  |
|  | MIRLET7A2 | |  |  |
|  | SAT1 | |  |  |
|  | ATF4 | |  |  |
|  | FMR1 | |  |  |
|  | STX11 | |  |  |
|  | DGCR5 | |  |  |
|  | EPB41L4A-DT | |  |  |
|  | LIN28A | |  |  |
|  | LPAR1 | |  |  |
|  | HOXA10 | |  |  |
|  | MIR498 | |  |  |
|  | MCM5 | |  |  |
|  | IGFBP4 | |  |  |
|  | NCL | |  |  |
|  | MIR637 | |  |  |
|  | TTK | |  |  |
|  | DEF6 | |  |  |
|  | IRAK4 | |  |  |
|  | RARRES2 | |  |  |
|  | NAT1 | |  |  |
|  | AKR1C3 | |  |  |
|  | GPR87 | |  |  |
|  | MAPK12 | |  |  |
|  | TNFRSF9 | |  |  |
|  | LATS2 | |  |  |
|  | STK19 | |  |  |
|  | ANK1 | |  |  |
|  | MIR503 | |  |  |
|  | HAVCR1 | |  |  |
|  | FOXC2 | |  |  |
|  | UBE2T | |  |  |
|  | ISL1 | |  |  |
|  | LAMB1 | |  |  |
|  | GLA | |  |  |
|  | MAP2K3 | |  |  |
|  | MIR28 | |  |  |
|  | MAF | |  |  |
|  | NCK1 | |  |  |
|  | GZMA | |  |  |
|  | IST1 | |  |  |
|  | FERMT3 | |  |  |
|  | TERF2 | |  |  |
|  | PDIA3 | |  |  |
|  | IVNS1ABP | |  |  |
|  | TXNRD2 | |  |  |
|  | GJB1 | |  |  |
|  | LAMP1 | |  |  |
|  | CHAT | |  |  |
|  | PITX2 | |  |  |
|  | TUBA1B | |  |  |
|  | NKX2-5 | |  |  |
|  | AIF1 | |  |  |
|  | MIR378A | |  |  |
|  | TGM3 | |  |  |
|  | GPX3 | |  |  |
|  | NAGA | |  |  |
|  | TACR1 | |  |  |
|  | SIRT6 | |  |  |
|  | PSMC4 | |  |  |
|  | ATF2 | |  |  |
|  | LIMK1 | |  |  |
|  | RNF113A | |  |  |
|  | NOS1 | |  |  |
|  | STUB1 | |  |  |
|  | MIR218-1 | |  |  |
|  | SERPINH1 | |  |  |
|  | CASP6 | |  |  |
|  | F11R | |  |  |
|  | FTO | |  |  |
|  | POFUT1 | |  |  |
|  | CTBP2 | |  |  |
|  | AOC3 | |  |  |
|  | MIR154 | |  |  |
|  | SEMA3B | |  |  |
|  | CAMK2G | |  |  |
|  | BPIFA1 | |  |  |
|  | CX3CL1 | |  |  |
|  | RSF1 | |  |  |
|  | EVPL | |  |  |
|  | TOP3A | |  |  |
|  | RACGAP1 | |  |  |
|  | DGCR8 | |  |  |
|  | KIF11 | |  |  |
|  | BCL2L2 | |  |  |
|  | WDR1 | |  |  |
|  | PTX3 | |  |  |
|  | HAX1 | |  |  |
|  | SFTPB | |  |  |
|  | HOXB9 | |  |  |
|  | HPS3 | |  |  |
|  | MIR216A | |  |  |
|  | E2F5 | |  |  |
|  | BLOC1S5 | |  |  |
|  | CHRNA5 | |  |  |
|  | DKK3 | |  |  |
|  | SPRED2 | |  |  |
|  | FZD2 | |  |  |
|  | UROD | |  |  |
|  | CGB3 | |  |  |
|  | KAT2B | |  |  |
|  | ADAMTS9-AS2 |  | |  |
|  | PYCARD | |  |  |
|  | MIR9-2 | |  |  |
|  | YWHAQ | |  |  |
|  | GNL3 | |  |  |
|  | PTBP1 | |  |  |
|  | LIPC | |  |  |
|  | SFTPA2 | |  |  |
|  | MIR367 | |  |  |
|  | EPS8 | |  |  |
|  | SFTPD | |  |  |
|  | FGF19 | |  |  |
|  | HLA-DRB5 | |  |  |
|  | ERN1 | |  |  |
|  | RARG | |  |  |
|  | FBL | |  |  |
|  | MFN2 | |  |  |
|  | NKX3-1 | |  |  |
|  | SERPINB2 | |  |  |
|  | EPHA1 | |  |  |
|  | UPF1 | |  |  |
|  | TYROBP | |  |  |
|  | AVP | |  |  |
|  | HMBS | |  |  |
|  | SLC6A3 | |  |  |
|  | PLEK | |  |  |
|  | RTN4 | |  |  |
|  | VCAN | |  |  |
|  | CD276 | |  |  |
|  | SMARCC2 | |  |  |
|  | DOT1L | |  |  |
|  | SSTR1 | |  |  |
|  | GAB1 | |  |  |
|  | COX5A | |  |  |
|  | MIAT | |  |  |
|  | LMNB1 | |  |  |
|  | INPP5D | |  |  |
|  | CAD | |  |  |
|  | HLA-DMA | |  |  |
|  | FKBP1A | |  |  |
|  | IL17F | |  |  |
|  | PDGFD | |  |  |
|  | LINC00673 |  | |  |
|  | SLC25A13 | |  |  |
|  | PF4 | |  |  |
|  | PTP4A3 | |  |  |
|  | NR4A1 | |  |  |
|  | HOXA9 | |  |  |
|  | PSMB4 | |  |  |
|  | PRKG1 | |  |  |
|  | UPK1A | |  |  |
|  | CYP24A1 | |  |  |
|  | GRIN1 | |  |  |
|  | TRPV1 | |  |  |
|  | CTNNBIP1 | |  |  |
|  | NACC1 | |  |  |
|  | KIR2DS5 | |  |  |
|  | CHRNA3 | |  |  |
|  | GTF2E2 | |  |  |
|  | ABCC6 | |  |  |
|  | SEPTIN9 | |  |  |
|  | CSPG4 | |  |  |
|  | EFNB2 | |  |  |
|  | PTRH2 | |  |  |
|  | P4HB | |  |  |
|  | PRC1 | |  |  |
|  | CTBP1 | |  |  |
|  | BIN1 | |  |  |
|  | LIN7C | |  |  |
|  | CILK1 | |  |  |
|  | KCNH1 | |  |  |
|  | PRDM14 | |  |  |
|  | ASIP | |  |  |
|  | TTC7A | |  |  |
|  | CCNT1 | |  |  |
|  | PTGER2 | |  |  |
|  | SEMA3A | |  |  |
|  | RASA2 | |  |  |
|  | SNCG | |  |  |
|  | CENPF | |  |  |
|  | KLK7 | |  |  |
|  | SCUBE3 | |  |  |
|  | ITGA7 | |  |  |
|  | CBS | |  |  |
|  | HPS5 | |  |  |
|  | ACTR3 | |  |  |
|  | TCIM | |  |  |
|  | CDCA3 | |  |  |
|  | MT-CYB | |  |  |
|  | SLC52A3 | |  |  |
|  | LGALS7B | |  |  |
|  | SCAI | |  |  |
|  | SAMHD1 | |  |  |
|  | PDE4A | |  |  |
|  | JAM3 | |  |  |
|  | MDH2 | |  |  |
|  | ITCH | |  |  |
|  | SLC5A1 | |  |  |
|  | DMPK | |  |  |
|  | SKIV2L | |  |  |
|  | HOXB-AS3 | |  |  |
|  | SLC9A1 | |  |  |
|  | SUZ12 | |  |  |
|  | DYNC2H1 | |  |  |
|  | IREB2 | |  |  |
|  | SFRP4 | |  |  |
|  | IL17RA | |  |  |
|  | F8 | |  |  |
|  | MIR490 | |  |  |
|  | SCT | |  |  |
|  | SPINK1 | |  |  |
|  | HTATIP2 | |  |  |
|  | AKAP13 | |  |  |
|  | FHL2 | |  |  |
|  | CYBA | |  |  |
|  | EHMT2 | |  |  |
|  | MLST8 | |  |  |
|  | SETX | |  |  |
|  | HIPK2 | |  |  |
|  | RALBP1 | |  |  |
|  | ALAD | |  |  |
|  | FST | |  |  |
|  | TSPO | |  |  |
|  | GSDME | |  |  |
|  | VAV3 | |  |  |
|  | TWIST2 | |  |  |
|  | GP1BA | |  |  |
|  | ELF4 | |  |  |
|  | MSX2 | |  |  |
|  | MADD | |  |  |
|  | KHDRBS1 | |  |  |
|  | GAS6 | |  |  |
|  | YES1 | |  |  |
|  | CXCL11 | |  |  |
|  | MST1 | |  |  |
|  | PBXIP1 | |  |  |
|  | SIRT2 | |  |  |
|  | APC2 | |  |  |
|  | PDGFC | |  |  |
|  | CUX1 | |  |  |
|  | BRD7 | |  |  |
|  | GAD1 | |  |  |
|  | PFKM | |  |  |
|  | SPTBN1 | |  |  |
|  | AGR2 | |  |  |
|  | NPPA | |  |  |
|  | GPC1 | |  |  |
|  | FUCA1 | |  |  |
|  | NTF3 | |  |  |
|  | HAND2-AS1 | |  |  |
|  | RAD9A | |  |  |
|  | MIR337 | |  |  |
|  | EEF2 | |  |  |
|  | PHGDH | |  |  |
|  | NPPB | |  |  |
|  | MIR31HG | |  |  |
|  | CCBE1 | |  |  |
|  | MELK | |  |  |
|  | HPS1 | |  |  |
|  | CXCL2 | |  |  |
|  | RBBP4 | |  |  |
|  | METTL3 | |  |  |
|  | TUSC3 | |  |  |
|  | MIR30C1 | |  |  |
|  | MPP1 | |  |  |
|  | TFAP2C | |  |  |
|  | MIR371A | |  |  |
|  | G3BP1 | |  |  |
|  | AMER1 | |  |  |
|  | RAD17 | |  |  |
|  | PSMD10 | |  |  |
|  | TCOF1 | |  |  |
|  | EIF3I | |  |  |
|  | TNFSF13 | |  |  |
|  | MAP1B | |  |  |
|  | CPQ | |  |  |
|  | SMARCD2 | |  |  |
|  | PPP1R13L | |  |  |
|  | PYY | |  |  |
|  | MIR95 | |  |  |
|  | IHH | |  |  |
|  | XK | |  |  |
|  | BTNL2 | |  |  |
|  | IGFBP7 | |  |  |
|  | TMPO | |  |  |
|  | ANXA4 | |  |  |
|  | PPL | |  |  |
|  | THBS2 | |  |  |
|  | CLDN5 | |  |  |
|  | BUB3 | |  |  |
|  | USF2 | |  |  |
|  | STAT2 | |  |  |
|  | P4HA2 | |  |  |
|  | REG1A | |  |  |
|  | IL16 | |  |  |
|  | AGRN | |  |  |
|  | SAFB | |  |  |
|  | FOSL2 | |  |  |
|  | CSNK1E | |  |  |
|  | CYB5A | |  |  |
|  | MAGEC2 | |  |  |
|  | SYNE1 | |  |  |
|  | SIN3A | |  |  |
|  | ADGRE5 | |  |  |
|  | FBLN1 | |  |  |
|  | PRDM2 | |  |  |
|  | CKB | |  |  |
|  | HOXC13 | |  |  |
|  | YWHAH | |  |  |
|  | MMP8 | |  |  |
|  | ESRRA | |  |  |
|  | FABP5 | |  |  |
|  | TBX3 | |  |  |
|  | INTS6 | |  |  |
|  | AFDN | |  |  |
|  | SLC22A2 | |  |  |
|  | ALPL | |  |  |
|  | FGL1 | |  |  |
|  | MIR345 | |  |  |
|  | ATOH1 | |  |  |
|  | ACY1 | |  |  |
|  | MAPKAPK2 | |  |  |
|  | SHMT1 | |  |  |
|  | KIF23 | |  |  |
|  | CRTC3 | |  |  |
|  | CCNB2 | |  |  |
|  | CSTB | |  |  |
|  | MIR124-3 | |  |  |
|  | FTL | |  |  |
|  | CDA | |  |  |
|  | HAS2 | |  |  |
|  | REV3L | |  |  |
|  | SCN10A | |  |  |
|  | JMJD1C | |  |  |
|  | SNHG20 | |  |  |
|  | EEF1A1 | |  |  |
|  | RORC | |  |  |
|  | RPA2 | |  |  |
|  | NUP98 | |  |  |
|  | ZNRD1ASP | |  |  |
|  | TPM1 | |  |  |
|  | NID1 | |  |  |
|  | OSMR | |  |  |
|  | KANK1 | |  |  |
|  | COL5A2 | |  |  |
|  | FZD1 | |  |  |
|  | ATF6 | |  |  |
|  | MYOF | |  |  |
|  | SLC25A46 | |  |  |
|  | FZD6 | |  |  |
|  | FUT2 | |  |  |
|  | MYO6 | |  |  |
|  | PLD1 | |  |  |
|  | MFGE8 | |  |  |
|  | LPCAT1 | |  |  |
|  | DEK | |  |  |
|  | INPPL1 | |  |  |
|  | PBK | |  |  |
|  | ILF3 | |  |  |
|  | TPM4 | |  |  |
|  | PKD2 | |  |  |
|  | TPP2 | |  |  |
|  | SLC45A2 | |  |  |
|  | ARTN | |  |  |
|  | KRIT1 | |  |  |
|  | DDC | |  |  |
|  | APOD | |  |  |
|  | WNK1 | |  |  |
|  | CTAGE1 | |  |  |
|  | PLAGL1 | |  |  |
|  | ANKRD11 | |  |  |
|  | RAB23 | |  |  |
|  | NEFH | |  |  |
|  | MSI2 | |  |  |
|  | RPS6KA3 | |  |  |
|  | MIR302B | |  |  |
|  | GIP | |  |  |
|  | SLC1A2 | |  |  |
|  | PRMT1 | |  |  |
|  | GAS5-AS1 | |  |  |
|  | SLIT2 | |  |  |
|  | BRINP3 | |  |  |
|  | IGHMBP2 | |  |  |
|  | SLC39A4 | |  |  |
|  | DLEU1 | |  |  |
|  | MAP2K6 | |  |  |
|  | MIR572 | |  |  |
|  | CHRNB4 | |  |  |
|  | MBTPS2 | |  |  |
|  | LACTB | |  |  |
|  | UGCG | |  |  |
|  | FHL1 | |  |  |
|  | FTH1 | |  |  |
|  | CST6 | |  |  |
|  | CDKN2D | |  |  |
|  | MIR628 | |  |  |
|  | GPRC5A | |  |  |
|  | TRPS1 | |  |  |
|  | CHRNA7 | |  |  |
|  | KCNH2 | |  |  |
|  | MLXIPL | |  |  |
|  | MSI1 | |  |  |
|  | SMAD1 | |  |  |
|  | POU2F1 | |  |  |
|  | CDK8 | |  |  |
|  | SLC8A1 | |  |  |
|  | SCARB2 | |  |  |
|  | NUS1 | |  |  |
|  | TPM2 | |  |  |
|  | CHKA | |  |  |
|  | NR6A1 | |  |  |
|  | USP9X | |  |  |
|  | IL22 | |  |  |
|  | NLK | |  |  |
|  | FAM3D-AS1 | |  |  |
|  | KDM6B | |  |  |
|  | AGK | |  |  |
|  | ISG15 | |  |  |
|  | FOXP2 | |  |  |
|  | HSD11B2 | |  |  |
|  | RNASE3 | |  |  |
|  | CD74 | |  |  |
|  | SIX3 | |  |  |
|  | ITGB5 | |  |  |
|  | ZIC2 | |  |  |
|  | PLK2 | |  |  |
|  | PMP22 | |  |  |
|  | TNFAIP8L2 | |  |  |
|  | TMPRSS11A | |  |  |
|  | LPAR3 | |  |  |
|  | RPE65 | |  |  |
|  | COL1A2 | |  |  |
|  | LOC110806306 | |  |  |
|  | SETD1A | |  |  |
|  | ITGB7 | |  |  |
|  | CSF2RA | |  |  |
|  | NUP62 | |  |  |
|  | RNF2 | |  |  |
|  | MUS81 | |  |  |
|  | LRRC8A | |  |  |
|  | SMC3 | |  |  |
|  | ITPR1 | |  |  |
|  | FLII | |  |  |
|  | GPX4 | |  |  |
|  | CCL21 | |  |  |
|  | TACC3 | |  |  |
|  | MTA2 | |  |  |
|  | NEFL | |  |  |
|  | ENTPD1 | |  |  |
|  | DBH | |  |  |
|  | SPRR3 | |  |  |
|  | NMB | |  |  |
|  | FLOT2 | |  |  |
|  | FANCB | |  |  |
|  | SRSF1 | |  |  |
|  | PXDN | |  |  |
|  | MYBL2 | |  |  |
|  | HIC1 | |  |  |
|  | TNFRSF25 | |  |  |
|  | SMARCD1 | |  |  |
|  | WWP2 | |  |  |
|  | CALM1 | |  |  |
|  | RAB25 | |  |  |
|  | BACH1 | |  |  |
|  | BRINP1 | |  |  |
|  | KLF2 | |  |  |
|  | EGR2 | |  |  |
|  | NKX6-1 | |  |  |
|  | RAB5A | |  |  |
|  | KLK10 | |  |  |
|  | SLC22A5 | |  |  |
|  | LGALS3BP | |  |  |
|  | MIR99B | |  |  |
|  | EFNB3 | |  |  |
|  | SFRP5 | |  |  |
|  | TRPV6 | |  |  |
|  | NABP1 | |  |  |
|  | EIF2S1 | |  |  |
|  | PPIG | |  |  |
|  | PDCD7 | |  |  |
|  | ID4 | |  |  |
|  | LDHB | |  |  |
|  | MIR29B2 | |  |  |
|  | KLK4 | |  |  |
|  | ADGRG1 | |  |  |
|  | CTNND2 | |  |  |
|  | MIR491 | |  |  |
|  | RBM10 | |  |  |
|  | GRIN2A | |  |  |
|  | KDM4B | |  |  |
|  | KPNA2 | |  |  |
|  | GAD2 | |  |  |
|  | POR | |  |  |
|  | DEFB103B | |  |  |
|  | CD1B | |  |  |
|  | CLDN10 | |  |  |
|  | HOXA1 | |  |  |
|  | TUBG1 | |  |  |
|  | IL27 | |  |  |
|  | MTRR | |  |  |
|  | SALL2 | |  |  |
|  | MTSS1 | |  |  |
|  | PRSS8 | |  |  |
|  | CST3 | |  |  |
|  | HDAC8 | |  |  |
|  | MIR92A1 | |  |  |
|  | STAG2 | |  |  |
|  | FLOT1 | |  |  |
|  | SKI | |  |  |
|  | UBAC2 | |  |  |
|  | SOAT1 | |  |  |
|  | NIPBL | |  |  |
|  | ECE1 | |  |  |
|  | ZBTB7A | |  |  |
|  | SDCBP | |  |  |
|  | IL15RA | |  |  |
|  | LINC03033 | |  |  |
|  | FOXG1 | |  |  |
|  | PSMD6 | |  |  |
|  | GAB2 | |  |  |
|  | NFKBIL1 | |  |  |
|  | CCL19 | |  |  |
|  | GPR68 | |  |  |
|  | TNFRSF18 | |  |  |
|  | TCHH | |  |  |
|  | VSIR | |  |  |
|  | HHIP | |  |  |
|  | BAG3 | |  |  |
|  | EIF3M | |  |  |
|  | LPAR4 | |  |  |
|  | FXR1 | |  |  |
|  | FGR | |  |  |
|  | PSPN | |  |  |
|  | CD160 | |  |  |
|  | TCERG1 | |  |  |
|  | UBE2C | |  |  |
|  | MAPRE1 | |  |  |
|  | MIR125B2 | |  |  |
|  | RAB7A | |  |  |
|  | SLC6A2 | |  |  |
|  | AIMP2 | |  |  |
|  | UBQLN2 | |  |  |
|  | PES1 | |  |  |
|  | CYB5R3 | |  |  |
|  | MIR377 | |  |  |
|  | PRKAB1 | |  |  |
|  | MIR219A1 | |  |  |
|  | PLCB1 | |  |  |
|  | MIR135A1 | |  |  |
|  | TNXB | |  |  |
|  | HOXA5 | |  |  |
|  | TRPM2 | |  |  |
|  | DUSP6 | |  |  |
|  | AMBP | |  |  |
|  | COL5A1 | |  |  |
|  | GPI | |  |  |
|  | GJC1 | |  |  |
|  | UCP2 | |  |  |
|  | DCT | |  |  |
|  | HBB-LCR | |  |  |
|  | APPL1 | |  |  |
|  | CLDN2 | |  |  |
|  | H1-5 | |  |  |
|  | LIG3 | |  |  |
|  | PLCB4 | |  |  |
|  | MIR574 | |  |  |
|  | ZNF750 | |  |  |
|  | FZD4 | |  |  |
|  | CBR1 | |  |  |
|  | PMVK | |  |  |
|  | OGT | |  |  |
|  | CHFR | |  |  |
|  | ABCD1 | |  |  |
|  | MATR3 | |  |  |
|  | FAT4 | |  |  |
|  | H4C1 | |  |  |
|  | SLC1A5 | |  |  |
|  | CSE1L | |  |  |
|  | TMSB4X | |  |  |
|  | PLK4 | |  |  |
|  | FGB | |  |  |
|  | F2RL3 | |  |  |
|  | SLC6A4 | |  |  |
|  | IRX2 | |  |  |
|  | WNT2B | |  |  |
|  | TRPC4 | |  |  |
|  | FZD5 | |  |  |
|  | MX1 | |  |  |
|  | RIPK2 | |  |  |
|  | UBB | |  |  |
|  | VAPB | |  |  |
|  | ADNP | |  |  |
|  | PRPF6 | |  |  |
|  | WNT6 | |  |  |
|  | MIR376C | |  |  |
|  | CXCL5 | |  |  |
|  | TOB1 | |  |  |
|  | NOX1 | |  |  |
|  | GHSR | |  |  |
|  | FUT3 | |  |  |
|  | TCTN1 | |  |  |
|  | WFDC2 | |  |  |
|  | FLVCR1 | |  |  |
|  | RBP4 | |  |  |
|  | GNA12 | |  |  |
|  | MAP3K20 | |  |  |
|  | MIR103A1 | |  |  |
|  | TNK2 | |  |  |
|  | AFAP1 | |  |  |
|  | TRRAP | |  |  |
|  | BHLHE40 | |  |  |
|  | SYNM | |  |  |
|  | PAH | |  |  |
|  | CASP4 | |  |  |
|  | SLC39A1 | |  |  |
|  | AQP5 | |  |  |
|  | TCHP | |  |  |
|  | PABPC1 | |  |  |
|  | RPS3 | |  |  |
|  | C5AR1 | |  |  |
|  | CD200R1 | |  |  |
|  | H3-3B | |  |  |
|  | CIC | |  |  |
|  | LMOD1 | |  |  |
|  | MAPKAP1 | |  |  |
|  | ATP1A3 | |  |  |
|  | ACVR1 | |  |  |
|  | PTS | |  |  |
|  | FGF5 | |  |  |
|  | CNR2 | |  |  |
|  | TP53COR1 | |  |  |
|  | SLC7A11 | |  |  |
|  | RBP1 | |  |  |
|  | HSPB2 | |  |  |
|  | MORC2 | |  |  |
|  | KISS1R | |  |  |
|  | PKP2 | |  |  |
|  | MAOA | |  |  |
|  | ATG5 | |  |  |
|  | DHX9 | |  |  |
|  | APLN | |  |  |
|  | NDRG2 | |  |  |
|  | CAPN2 | |  |  |
|  | PPP1CA | |  |  |
|  | AGO1 | |  |  |
|  | ADORA2A | |  |  |
|  | PSME3 | |  |  |
|  | MIR495 | |  |  |
|  | THRA | |  |  |
|  | SERPINE2 | |  |  |
|  | POLR2L | |  |  |
|  | SOX6 | |  |  |
|  | TRIM21 | |  |  |
|  | VIL1 | |  |  |
|  | NUDT1 | |  |  |
|  | LRAT | |  |  |
|  | MLKL | |  |  |
|  | PMM2 | |  |  |
|  | LARP1 | |  |  |
|  | MCM6 | |  |  |
|  | PENK | |  |  |
|  | KDM2B | |  |  |
|  | PAPPA | |  |  |
|  | PPBP | |  |  |
|  | TRPM8 | |  |  |
|  | SPRR1B | |  |  |
|  | PURA | |  |  |
|  | KLK6 | |  |  |
|  | EPG5 | |  |  |
|  | CGB7 | |  |  |
|  | CLIC1 | |  |  |
|  | ALX4 | |  |  |
|  | TNFRSF14 | |  |  |
|  | ERCC8 | |  |  |
|  | DSC1 | |  |  |
|  | SLC25A11 | |  |  |
|  | CHAF1A | |  |  |
|  | JARID2 | |  |  |
|  | NOP2 | |  |  |
|  | PDE4D | |  |  |
|  | TAT | |  |  |
|  | PSAT1 | |  |  |
|  | LINC01234 | |  |  |
|  | SPTAN1 | |  |  |
|  | CAPN1 | |  |  |
|  | SERPINB1 | |  |  |
|  | MAP2K7 | |  |  |
|  | CCL7 | |  |  |
|  | PTPRF | |  |  |
|  | NDUFS4 | |  |  |
|  | TXNRD1 | |  |  |
|  | PNPLA6 | |  |  |
|  | EBF3 | |  |  |
|  | ZNF639 | |  |  |
|  | PROS1 | |  |  |
|  | ZNF341 | |  |  |
|  | BAG6 | |  |  |
|  | UBE2I | |  |  |
|  | RORA | |  |  |
|  | NUPR1 | |  |  |
|  | KIF5B | |  |  |
|  | PEX1 | |  |  |
|  | MYH7 | |  |  |
|  | POU3F3 | |  |  |
|  | NIBAN1 | |  |  |
|  | HAS3 | |  |  |
|  | HOXB7 | |  |  |
|  | EIF6 | |  |  |
|  | SOX7 | |  |  |
|  | SPIB | |  |  |
|  | POLI | |  |  |
|  | EFNB1 | |  |  |
|  | ZMIZ1 | |  |  |
|  | ESRP1 | |  |  |
|  | CLCA2 | |  |  |
|  | F7 | |  |  |
|  | SOX3 | |  |  |
|  | BCAR3 | |  |  |
|  | H1-1 | |  |  |
|  | HPN | |  |  |
|  | PLXNA1 | |  |  |
|  | LRP2 | |  |  |
|  | GTF2H4 | |  |  |
|  | MIR129-1 | |  |  |
|  | MIR361 | |  |  |
|  | LAMP3 | |  |  |
|  | MIR744 | |  |  |
|  | MIR675 | |  |  |
|  | M6PR | |  |  |
|  | POGZ | |  |  |
|  | HTR3A | |  |  |
|  | SP3 | |  |  |
|  | NOD1 | |  |  |
|  | LTBP1 | |  |  |
|  | ATP4A | |  |  |
|  | CCT3 | |  |  |
|  | TXNIP | |  |  |
|  | PCBP2 | |  |  |
|  | TNFSF9 | |  |  |
|  | GCLC | |  |  |
|  | FLNC | |  |  |
|  | HTRA1 | |  |  |
|  | NTN1 | |  |  |
|  | UGT1A6 | |  |  |
|  | CD1E | |  |  |
|  | MAP1LC3B | |  |  |
|  | DDIT4 | |  |  |
|  | TBXT | |  |  |
|  | PAEP | |  |  |
|  | VASP | |  |  |
|  | BATF2 | |  |  |
|  | SELPLG | |  |  |
|  | CCNF | |  |  |
|  | ALOX15 | |  |  |
|  | AIM2 | |  |  |
|  | PSMD9 | |  |  |
|  | TRPC1 | |  |  |
|  | CCL27 | |  |  |
|  | ST3GAL4 | |  |  |
|  | PCSK1 | |  |  |
|  | IMPG1 | |  |  |
|  | CTNNBL1 | |  |  |
|  | EMP1 | |  |  |
|  | MYH3 | |  |  |
|  | FBLIM1 | |  |  |
|  | NEDD8 | |  |  |
|  | MAFB | |  |  |
|  | SS18 | |  |  |
|  | TRIM14 | |  |  |
|  | PRDX3 | |  |  |
|  | NOP56 | |  |  |
|  | FOXD3 | |  |  |
|  | HLA-DQB2 | |  |  |
|  | RECQL5 | |  |  |
|  | MUC7 | |  |  |
|  | KRT9 | |  |  |
|  | NMBR | |  |  |
|  | ZNRF3 | |  |  |
|  | CCL26 | |  |  |
|  | SYMPK | |  |  |
|  | S100P | |  |  |
|  | RIPK3 | |  |  |
|  | NBAS | |  |  |
|  | BRS3 | |  |  |
|  | BCL2A1 | |  |  |
|  | CLOCK | |  |  |
|  | NCF2 | |  |  |
|  | CRAT | |  |  |
|  | RAC3 | |  |  |
|  | PELP1 | |  |  |
|  | MIR7-1 | |  |  |
|  | CDC42BPB | |  |  |
|  | C4B | |  |  |
|  | SPOP | |  |  |
|  | NUP88 | |  |  |
|  | CCDC88A | |  |  |
|  | HCFC2 | |  |  |
|  | SRSF3 | |  |  |
|  | NAA10 | |  |  |
|  | UGT8 | |  |  |
|  | APOBEC3B | |  |  |
|  | S100A11 | |  |  |
|  | ILF2 | |  |  |
|  | CDK20 | |  |  |
|  | HGS | |  |  |
|  | PDE5A | |  |  |
|  | AKR1C2 | |  |  |
|  | ACACA | |  |  |
|  | HSPA1B | |  |  |
|  | IL1RAPL2 | |  |  |
|  | RPL29 | |  |  |
|  | ERAP1 | |  |  |
|  | SOCS2 | |  |  |
|  | PRPH | |  |  |
|  | POLR1H | |  |  |
|  | GLS | |  |  |
|  | DSPP | |  |  |
|  | BLZF1 | |  |  |
|  | FTH1P3 | |  |  |
|  | SRP72 | |  |  |
|  | NUP133 | |  |  |
|  | HNRNPM | |  |  |
|  | CCNG2 | |  |  |
|  | COL4A5 | |  |  |
|  | HLA-DOB | |  |  |
|  | IER3 | |  |  |
|  | UBE2N | |  |  |
|  | CCR9 | |  |  |
|  | PKN2 | |  |  |
|  | RETREG1 | |  |  |
|  | SPEN | |  |  |
|  | HTR1A | |  |  |
|  | GRHL3 | |  |  |
|  | GNAT1 | |  |  |
|  | CLDN11 | |  |  |
|  | PRKD3 | |  |  |
|  | EPHX2 | |  |  |
|  | SLC39A7 | |  |  |
|  | LPXN | |  |  |
|  | TUBB4A | |  |  |
|  | KLF10 | |  |  |
|  | EPHB3 | |  |  |
|  | KAT2A | |  |  |
|  | HNRNPL | |  |  |
|  | TRAP1 | |  |  |
|  | EPHB1 | |  |  |
|  | ITGB8 | |  |  |
|  | PTPN14 | |  |  |
|  | P2RY12 | |  |  |
|  | FSCN2 | |  |  |
|  | CXCR6 | |  |  |
|  | KRT3 | |  |  |
|  | APTX | |  |  |
|  | SLC18A2 | |  |  |
|  | ATXN3 | |  |  |
|  | HOXA-AS2 | |  |  |
|  | TAF15 | |  |  |
|  | DPH1 | |  |  |
|  | CNTN1 | |  |  |
|  | COLQ | |  |  |
|  | CELF2 | |  |  |
|  | PPP1R10 | |  |  |
|  | PA2G4 | |  |  |
|  | ALOX5AP | |  |  |
|  | TTN-AS1 | |  |  |
|  | SLC39A6 | |  |  |
|  | SERPINB9 | |  |  |
|  | AKT1S1 | |  |  |
|  | ITGA8 | |  |  |
|  | RPRD1B | |  |  |
|  | PIP5K1A | |  |  |
|  | EIF5A | |  |  |
|  | ASCC1 | |  |  |
|  | DKK2 | |  |  |
|  | PCSK9 | |  |  |
|  | FOXF1 | |  |  |
|  | PRDX6 | |  |  |
|  | DEFA1 | |  |  |
|  | TAB2 | |  |  |
|  | RNF168 | |  |  |
|  | SPINT1 | |  |  |
|  | VAV2 | |  |  |
|  | PER1 | |  |  |
|  | DUSP19 | |  |  |
|  | EMD | |  |  |
|  | MAP3K11 | |  |  |
|  | CHRM3 | |  |  |
|  | EHF | |  |  |
|  | GARS1 | |  |  |
|  | MYH8 | |  |  |
|  | SCD | |  |  |
|  | ACLY | |  |  |
|  | UBASH3B | |  |  |
|  | DPYSL5 | |  |  |
|  | ANLN | |  |  |
|  | KRT12 | |  |  |
|  | PDE2A | |  |  |
|  | SERPINF2 | |  |  |
|  | LMX1B | |  |  |
|  | KPNB1 | |  |  |
|  | CELF1 | |  |  |
|  | ADORA2B | |  |  |
|  | LCOR | |  |  |
|  | CD93 | |  |  |
|  | ATP1A1 | |  |  |
|  | DDX39B | |  |  |
|  | PTGES | |  |  |
|  | DUSP4 | |  |  |
|  | HLA-DMB | |  |  |
|  | SLC16A4 | |  |  |
|  | ANXA3 | |  |  |
|  | C1S | |  |  |
|  | LGALS4 | |  |  |
|  | MBD2 | |  |  |
|  | ADCYAP1 | |  |  |
|  | KCNN3 | |  |  |
|  | GRK2 | |  |  |
|  | HEXA | |  |  |
|  | CDC42BPA | |  |  |
|  | MT-CO2 | |  |  |
|  | LINC00668 |  | |  |
|  | NSD3 | |  |  |
|  | ZCCHC8 | |  |  |
|  | MLLT3 | |  |  |
|  | DACH1 | |  |  |
|  | MIR563 | |  |  |
|  | FCGR1A | |  |  |
|  | CISH | |  |  |
|  | ERVW-1 | |  |  |
|  | NOVA1 | |  |  |
|  | CAMK2N1 | |  |  |
|  | UNC93B1 | |  |  |
|  | TBXAS1 | |  |  |
|  | YWHAB | |  |  |
|  | SP100 | |  |  |
|  | STK3 | |  |  |
|  | RASGRP3 | |  |  |
|  | ADK | |  |  |
|  | HSD11B1 | |  |  |
|  | CENPE | |  |  |
|  | UBE2K | |  |  |
|  | CYP2B6 | |  |  |
|  | ANOS1 | |  |  |
|  | RXRG | |  |  |
|  | GPD2 | |  |  |
|  | MAPRE2 | |  |  |
|  | RALY | |  |  |
|  | GC | |  |  |
|  | TUBA1C | |  |  |
|  | LUC7L2 | |  |  |
|  | BCHE | |  |  |
|  | VIPR1 | |  |  |
|  | NCR2 | |  |  |
|  | ATP2A3 | |  |  |
|  | PYM1 | |  |  |
|  | PRKCG | |  |  |
|  | TRIB3 | |  |  |
|  | HCP5 | |  |  |
|  | MAOB | |  |  |
|  | DLST | |  |  |
|  | RSPO2 | |  |  |
|  | TBCK | |  |  |
|  | SLC1A1 | |  |  |
|  | ATP2C1 | |  |  |
|  | HJURP | |  |  |
|  | HUWE1 | |  |  |
|  | GPC5 | |  |  |
|  | HEPH | |  |  |
|  | ZYX | |  |  |
|  | ID3 | |  |  |
|  | DNASE1L3 | |  |  |
|  | CTSS | |  |  |
|  | GIT1 | |  |  |
|  | HAS2-AS1 | |  |  |
|  | VEGFB | |  |  |
|  | MIR218-2 | |  |  |
|  | PLOD2 | |  |  |
|  | LDB1 | |  |  |
|  | ABCC5 | |  |  |
|  | ZFYVE9 | |  |  |
|  | DFFA | |  |  |
|  | TALDO1 | |  |  |
|  | EFNA5 | |  |  |
|  | NTSR1 | |  |  |
|  | MIEN1 | |  |  |
|  | TCN1 | |  |  |
|  | S100A12 | |  |  |
|  | MPI | |  |  |
|  | UCP1 | |  |  |
|  | PRDX5 | |  |  |
|  | HSPH1 | |  |  |
|  | KAT8 | |  |  |
|  | NR5A2 | |  |  |
|  | MIR33A | |  |  |
|  | ZFP57 | |  |  |
|  | PIP5K1C | |  |  |
|  | PI3 | |  |  |
|  | DRD3 | |  |  |
|  | BPTF | |  |  |
|  | CDSN | |  |  |
|  | UNG | |  |  |
|  | COL6A1 | |  |  |
|  | PHF8 | |  |  |
|  | PRPF4 | |  |  |
|  | BAIAP2L1 | |  |  |
|  | SOX18 | |  |  |
|  | BCAR4 | |  |  |
|  | E2F7 | |  |  |
|  | MAPK6 | |  |  |
|  | GSS | |  |  |
|  | H4C14 | |  |  |
|  | ATP12A | |  |  |
|  | MIR339 | |  |  |
|  | YWHAG | |  |  |
|  | DRD1 | |  |  |
|  | PINK1 | |  |  |
|  | TYRO3 | |  |  |
|  | INCENP | |  |  |
|  | ABCG1 | |  |  |
|  | CPS1 | |  |  |
|  | DPAGT1 | |  |  |
|  | SORT1 | |  |  |
|  | PCSK2 | |  |  |
|  | FUT8 | |  |  |
|  | GIPC1 | |  |  |
|  | CUL4B | |  |  |
|  | CLK2 | |  |  |
|  | SETD5 | |  |  |
|  | SEMA3C | |  |  |
|  | UGT1A9 | |  |  |
|  | LY96 | |  |  |
|  | ARNTL | |  |  |
|  | ING4 | |  |  |
|  | AARS1 | |  |  |
|  | PTGER3 | |  |  |
|  | SORD | |  |  |
|  | RCHY1 | |  |  |
|  | CLMP | |  |  |
|  | TNIP1 | |  |  |
|  | COL6A3 | |  |  |
|  | CFHR1 | |  |  |
|  | BDKRB2 | |  |  |
|  | MCPH1 | |  |  |
|  | ADRB1 | |  |  |
|  | DLL3 | |  |  |
|  | WASF2 | |  |  |
|  | CAST | |  |  |
|  | TUBB2A | |  |  |
|  | GNB3 | |  |  |
|  | SETD1B | |  |  |
|  | MIR151A | |  |  |
|  | NDN | |  |  |
|  | LIMA1 | |  |  |
|  | CEP55 | |  |  |
|  | HSD3B2 | |  |  |
|  | CYSLTR1 | |  |  |
|  | NEK8 | |  |  |
|  | WDR77 | |  |  |
|  | SND1 | |  |  |
|  | PPP1R1B | |  |  |
|  | KIF20B | |  |  |
|  | FBP1 | |  |  |
|  | CABLES1 | |  |  |
|  | TFAM | |  |  |
|  | ANXA11 | |  |  |
|  | FAM3C | |  |  |
|  | PGM1 | |  |  |
|  | EFL1 | |  |  |
|  | ATP1B1 | |  |  |
|  | KAT6A | |  |  |
|  | MSH5 | |  |  |
|  | PADI4 | |  |  |
|  | TREX1 | |  |  |
|  | EEF1A2 | |  |  |
|  | KIF14 | |  |  |
|  | MIR493 | |  |  |
|  | IRX5 | |  |  |
|  | PON2 | |  |  |
|  | SURF1 | |  |  |
|  | SALL1 | |  |  |
|  | SIAH1 | |  |  |
|  | POMGNT2 | |  |  |
|  | SLC7A8 | |  |  |
|  | RGS2 | |  |  |
|  | MIR425 | |  |  |
|  | AP3D1 | |  |  |
|  | NCOA6 | |  |  |
|  | WASHC4 | |  |  |
|  | ACER3 | |  |  |
|  | AKR1A1 | |  |  |
|  | SIRT7 | |  |  |
|  | EPRS1 | |  |  |
|  | CCN5 | |  |  |
|  | PSMA1 | |  |  |
|  | GNAI3 | |  |  |
|  | S1PR2 | |  |  |
|  | PDK2 | |  |  |
|  | FKBP5 | |  |  |
|  | PITX3 | |  |  |
|  | TET1 | |  |  |
|  | DDX11 | |  |  |
|  | MAT2A | |  |  |
|  | LPAR2 | |  |  |
|  | FZD8 | |  |  |
|  | PHOX2B | |  |  |
|  | TRPC3 | |  |  |
|  | LGMN | |  |  |
|  | PTAFR | |  |  |
|  | ARHGAP15 | |  |  |
|  | ACAN | |  |  |
|  | TRH | |  |  |
|  | SP140 | |  |  |
|  | MIR134 | |  |  |
|  | PTPRA | |  |  |
|  | TUBB4B | |  |  |
|  | TTF2 | |  |  |
|  | ABCF1 | |  |  |
|  | SEZ6L2 | |  |  |
|  | EHMT1 | |  |  |
|  | KLF14 | |  |  |
|  | SOX21-AS1 | |  |  |
|  | CEBPA-DT | |  |  |
|  | TRIO | |  |  |
|  | CTNNA3 | |  |  |
|  | H1-4 | |  |  |
|  | EXOC2 | |  |  |
|  | RNF31 | |  |  |
|  | FADS1 | |  |  |
|  | HEY2 | |  |  |
|  | UBA52 | |  |  |
|  | RERE | |  |  |
|  | EDA | |  |  |
|  | SI | |  |  |
|  | GABBR1 | |  |  |
|  | LINC00974 | |  |  |
|  | RPL7A | |  |  |
|  | IMPDH2 | |  |  |
|  | TCF19 | |  |  |
|  | SPA17 | |  |  |
|  | USF1 | |  |  |
|  | SULF2 | |  |  |
|  | CARD10 | |  |  |
|  | GJB5 | |  |  |
|  | SMC4 | |  |  |
|  | ANKRD26 | |  |  |
|  | CLCN3 | |  |  |
|  | GOLM1 | |  |  |
|  | CCR8 | |  |  |
|  | H6PD | |  |  |
|  | FOLR2 | |  |  |
|  | CLEC4A | |  |  |
|  | CYGB | |  |  |
|  | MMP26 | |  |  |
|  | CIRBP | |  |  |
|  | BGN | |  |  |
|  | PCBP1 | |  |  |
|  | NR1I3 | |  |  |
|  | SOX5 | |  |  |
|  | PROC | |  |  |
|  | CD37 | |  |  |
|  | COTL1 | |  |  |
|  | MDH1 | |  |  |
|  | CHRNE | |  |  |
|  | PGD | |  |  |
|  | GIPR | |  |  |
|  | UBE2L3 | |  |  |
|  | TEP1 | |  |  |
|  | LEMD2 | |  |  |
|  | PKP3 | |  |  |
|  | GALNS | |  |  |
|  | BANF1 | |  |  |
|  | CFI | |  |  |
|  | S100A14 | |  |  |
|  | LY6K | |  |  |
|  | EIF4A3 | |  |  |
|  | LTB | |  |  |
|  | HBP1 | |  |  |
|  | BOK | |  |  |
|  | HDC | |  |  |
|  | CKS2 | |  |  |
|  | DUOX2 | |  |  |
|  | PPP5C | |  |  |
|  | OLFM4 | |  |  |
|  | AGA | |  |  |
|  | SLC25A1 | |  |  |
|  | CAMTA1 | |  |  |
|  | SHMT2 | |  |  |
|  | GDF5 | |  |  |
|  | RPL3 | |  |  |
|  | NUDT6 | |  |  |
|  | TOLLIP | |  |  |
|  | MLF1 | |  |  |
|  | TOPBP1 | |  |  |
|  | EIF4B | |  |  |
|  | PTGDR | |  |  |
|  | SNRNP70 | |  |  |
|  | SNRNP200 | |  |  |
|  | DUXAP8 | |  |  |
|  | SIGMAR1 | |  |  |
|  | KCNK2 | |  |  |
|  | P2RY2 | |  |  |
|  | RFC4 | |  |  |
|  | STK33 | |  |  |
|  | FBXO11 | |  |  |
|  | DNAH17 | |  |  |
|  | STT3A | |  |  |
|  | ZFX | |  |  |
|  | POLR2B | |  |  |
|  | RBMX | |  |  |
|  | GIMAP6 | |  |  |
|  | RHOU | |  |  |
|  | LGR4 | |  |  |
|  | CCHCR1 | |  |  |
|  | FKBP4 | |  |  |
|  | ULK1 | |  |  |
|  | MIR136 | |  |  |
|  | MIR485 | |  |  |
|  | FRS2 | |  |  |
|  | MAU2 | |  |  |
|  | MTNR1B | |  |  |
|  | NR2C2 | |  |  |
|  | NOLC1 | |  |  |
|  | IL37 | |  |  |
|  | TM4SF1 | |  |  |
|  | NFIA | |  |  |
|  | LRP8 | |  |  |
|  | WASF3 | |  |  |
|  | MYO1C | |  |  |
|  | KIF1A | |  |  |
|  | CKAP4 | |  |  |
|  | CAMK2A | |  |  |
|  | PLEKHM1 | |  |  |
|  | ANXA6 | |  |  |
|  | PON3 | |  |  |
|  | SSB | |  |  |
|  | UBA7 | |  |  |
|  | TMEM80 | |  |  |
|  | SNHG3 | |  |  |
|  | OPRK1 | |  |  |
|  | GLIS3 | |  |  |
|  | TCF21 | |  |  |
|  | MFAP5 | |  |  |
|  | PFKP | |  |  |
|  | RHOBTB2 | |  |  |
|  | PTPRZ1 | |  |  |
|  | GOLPH3 | |  |  |
|  | USP22 | |  |  |
|  | SRPX2 | |  |  |
|  | LMO4 | |  |  |
|  | MIR885 | |  |  |
|  | PITX1 | |  |  |
|  | GPBAR1 | |  |  |
|  | MIR202 | |  |  |
|  | NELFCD | |  |  |
|  | SEPTIN2 | |  |  |
|  | HSPA14 | |  |  |
|  | BLOC1S5-TXNDC5 | |  |  |
|  | XCL1 | |  |  |
|  | MIR1-1 | |  |  |
|  | SLC34A1 | |  |  |
|  | SKIL | |  |  |
|  | DCUN1D5 | |  |  |
|  | GTF2I | |  |  |
|  | TTLL12 | |  |  |
|  | BAMBI | |  |  |
|  | SARNP | |  |  |
|  | SPINK5 | |  |  |
|  | GFER | |  |  |
|  | F2RL2 | |  |  |
|  | HLA-DOA | |  |  |
|  | MCU | |  |  |
|  | ASPH | |  |  |
|  | SREBF2 | |  |  |
|  | FPR2 | |  |  |
|  | EFNA3 | |  |  |
|  | NSF | |  |  |
|  | TBX5 | |  |  |
|  | YBX2 | |  |  |
|  | FRMD4A | |  |  |
|  | AAAS | |  |  |
|  | OPRD1 | |  |  |
|  | AGR3 | |  |  |
|  | SLC22A4 | |  |  |
|  | SEMA3E | |  |  |
|  | ATP2B1 | |  |  |
|  | GRB10 | |  |  |
|  | CHD1 | |  |  |
|  | GAK | |  |  |
|  | CDKL5 | |  |  |
|  | S100A10 | |  |  |
|  | WDR26 | |  |  |
|  | ENDOG | |  |  |
|  | DLX4 | |  |  |
|  | RDH12 | |  |  |
|  | CSN1S1 | |  |  |
|  | RUVBL2 | |  |  |
|  | KCNA2 | |  |  |
|  | ST3GAL1 | |  |  |
|  | SIPA1 | |  |  |
|  | ABCB5 | |  |  |
|  | CACNA2D2 | |  |  |
|  | CBY1 | |  |  |
|  | SLC12A4 | |  |  |
|  | LYZ | |  |  |
|  | RPL38 | |  |  |
|  | ASPRV1 | |  |  |
|  | IRF9 | |  |  |
|  | MED15 | |  |  |
|  | FSTL1 | |  |  |
|  | GTF2IRD2P1 | |  |  |
|  | SMURF2 | |  |  |
|  | TMPRSS4 | |  |  |
|  | ATF6B | |  |  |
|  | LSM2 | |  |  |
|  | TLE3 | |  |  |
|  | VPS33A | |  |  |
|  | DHPS | |  |  |
|  | PLD2 | |  |  |
|  | SCGB3A1 | |  |  |
|  | TRPC6 | |  |  |
|  | RCN1 | |  |  |
|  | CDK5RAP3 | |  |  |
|  | H4C11 | |  |  |
|  | NLRP12 | |  |  |
|  | CGAS | |  |  |
|  | SMAD5 | |  |  |
|  | C5 | |  |  |
|  | HNRNPC | |  |  |
|  | DDX6 | |  |  |
|  | H4C12 | |  |  |
|  | ADARB1 | |  |  |
|  | ESRRG | |  |  |
|  | FOSB | |  |  |
|  | PLXNC1 | |  |  |
|  | ENOX2 | |  |  |
|  | CXCL16 | |  |  |
|  | PSMC3 | |  |  |
|  | DPM1 | |  |  |
|  | CLCF1 | |  |  |
|  | TFAP2B | |  |  |
|  | GNS | |  |  |
|  | UGT1A8 | |  |  |
|  | PKP4 | |  |  |
|  | AIMP1 | |  |  |
|  | RPS6KA6 | |  |  |
|  | CLCA4 | |  |  |
|  | PEA15 | |  |  |
|  | SMG1 | |  |  |
|  | SELENOP | |  |  |
|  | AMBRA1 | |  |  |
|  | PSMC2 | |  |  |
|  | BMPR1B | |  |  |
|  | NR2F2 | |  |  |
|  | UGT1A7 | |  |  |
|  | HNRNPD | |  |  |
|  | ANXA8 | |  |  |
|  | HOXB2 | |  |  |
|  | IL17RC | |  |  |
|  | NR0B2 | |  |  |
|  | ADAM3A | |  |  |
|  | SPINK7 | |  |  |
|  | ATG16L1 | |  |  |
|  | DAPK3 | |  |  |
|  | ZBTB20 | |  |  |
|  | FBLN5 | |  |  |
|  | DLG4 | |  |  |
|  | MBNL1 | |  |  |
|  | UBR4 | |  |  |
|  | RLIM | |  |  |
|  | ARL2BP | |  |  |
|  | WASL | |  |  |
|  | REG4 | |  |  |
|  | RING1 | |  |  |
|  | LNPEP | |  |  |
|  | HSD3B1 | |  |  |
|  | PC | |  |  |
|  | SLC11A1 | |  |  |
|  | CASP14 | |  |  |
|  | KIF2A | |  |  |
|  | PTGDS | |  |  |
|  | SPAAR | |  |  |
|  | ZNF469 | |  |  |
|  | PEX2 | |  |  |
|  | LEMD3 | |  |  |
|  | WDR5 | |  |  |
|  | NUP153 | |  |  |
|  | NDC80 | |  |  |
|  | MT2A | |  |  |
|  | NCAPG | |  |  |
|  | APLNR | |  |  |
|  | AGAP2 | |  |  |
|  | MAGED2 | |  |  |
|  | PSMD4 | |  |  |
|  | TRIM26 | |  |  |
|  | EEF1D | |  |  |
|  | RPL22 | |  |  |
|  | MYDGF | |  |  |
|  | UBR5 | |  |  |
|  | ATRIP | |  |  |
|  | GSTA1 | |  |  |
|  | RCOR1 | |  |  |
|  | LEFTY2 | |  |  |
|  | MIR198 | |  |  |
|  | ZNF148 | |  |  |
|  | ETV7 | |  |  |
|  | CHD3 | |  |  |
|  | ANGPTL8 | |  |  |
|  | MAGEA9 | |  |  |
|  | POU1F1 | |  |  |
|  | CARM1 | |  |  |

| **Leukaemia** | **Pancreatic cancer** | **Melanoma** | **Glioma** | **Osteosarcoma** |
| --- | --- | --- | --- | --- |
| ABL1 | BRCA2 | CDKN2A | IDH1 | CHEK2 |
| ACSL6 | BRCA1 | CDK4 | LGI2 | RB1 |
| AF10 | TP53 | BRAF | GLM8 | TP53 |
| ALL1 | PALB2 | MC1R | POT1 | P53 |
| ALL2 | CHEK2 | POT1 | LGI3 | RAD53 |
| ARHGAP26 | ATM | NRAS | GLM7 | CHK2 |
| ARHGEF12 | KRAS | MITF | GLM5 | LFS1 |
| ATM | CDH1 | BAP1 | PTEN | BCC7 |
| BAALC | CDKN2A | TP53 | LGI1 | CDS1 |
| BAX | BRIP1 | GNAQ | GLIPR1 | BMFS5 |
| BCL10 | MSH2 | GNA11 | GLM4 | LFS2 |
| BCL2 | MLH1 | PTEN | TP53 | ATRX |
| BCL3 | MSH6 | KIT | LGI4 | RECQL4 |
| BCR | EGFR | TERT | GLM6 | MDM2 |
| BLACE | SMAD4 | CTNNB1 | AMC1 | CDKN2A |
| CBFB | PTEN | STK11 | CMM10 | MIR140 |
| CBL | APC | MAP2K1 | EPT | FOS |
| CEBPA | MIR21 | TYR | GLIPR | OS9 |
| CHIC2 | PIK3CA | HRAS | IDDMDS | CDK4 |
| CLLD6 | NBN | CMM | KIAA1916 | BGLAP |
| CLLS1 | ERBB2 | SF3B1 | MMAC1 | MTAP |
| CLLS2 | STK11 | CDKN2B | P53 |  |
| CLLS3 | PMS2 | CMM4 | ETL1 |  |
| CLLS4 | MIR34A | CMM7 | GLM2 |  |
| CLLS5 | C11orf65 | MGMT | GLM9 |  |
| CLLU1 | BARD1 | BRCA2 | LFS1 |  |
| CLLU1OS | MIR221 | PLCB4 | RTVP1 |  |
| CREBBP | AKT1 | UVM1 | ADLTE |  |
| CXCL10 | MIR17 | UVM2 | BCC7 |  |
| DEK | POLD1 | MCAM | CWS1 |  |
| DLEU1 | CTNNB1 | MLANA | ADPEAF |  |
| DLEU2 | BRAF | TERF2IP | BMFS5 |  |
| DLEU7 | SPINK1 | LOC110806263 | IDH2 |  |
| DNMT3A | MUTYH | PIK3CA | NF1 |  |
| ELL | CCND1 | CYSLTR2 | H3-3A |  |
| ERBB2 | POLE | PMEL | ERBB2 |  |
| ERBB4 | RAD51D | MIR34A | MIR21 |  |
| ERG | MIR155 | MIRLET7B | H3C1 |  |
| ETV6 | MET | MIR193B | BRCA2 |  |
| FLT3 | PALLD | MAGEA3 | EGFR |  |
| FNBP1 | RAD51C | MAGEA1 | LZTR1 |  |
| FUS | PDX1 | ACD | ATM |  |
| GATA1 | RB1 | MIR182 | MIR34A |  |
| GATA2 | RET | MIR221 | MGMT |  |
| GNB1 | HRAS | MIR23B | MIR106A |  |
| HLF | MIR15A | AKT1 | MIR221 |  |
| HOXA9 | MIR34C | LRRC56 | CHEK2 |  |
| HTLF | PRSS1 | XRCC3 | MIR15B |  |
| IRF1 | MIR96 | PALB2 | TERT |  |
| JAK2 | MIR222 | MIR205 | FGFR1 |  |
| KIT | MIR34B | NF1 | C11orf65 |  |
| KRAS | EPCAM | MIR222 | MIR17 |  |
| LALL | MIR145 | AIM2 | PIK3CA |  |
| LCK | FGFR2 | MIR532 | CDKN2A |  |
| LIF | MIR200B | MIA | AKT1 |  |
| LIFR | RAD50 | PMS2 | MIR222 |  |
| LMO1 | CASP8 | BAGE | NF2 |  |
| LMO2 | TGFBR2 | SNAPC5 | MIR146B |  |
| LPP | MIR126 | TYRP1 | MMP2 |  |
| LYL1 | TSC1 | KRAS | NTRK3 |  |
| MCL1 | MIR20A | FBN1 | GFAP |  |
| MKL1 | ESR1 | TSPAN31 | ATRX |  |
| MLF1 | MIR146A | PRAME | MIR184 |  |
| MLF2 | MIR200C | IL2 | MDM2 |  |
| MLLT11 | CFTR | RAF1 | TSC2 |  |
| MLLT3 | MEN1 | CDH1 | MIR296 |  |
| MLLT6 | AXIN2 | BRCA1 | MSH2 |  |
| MLRL | NF1 | RB1 | MIR181A2 |  |
| MLVI2 | MIR125A | MLH1 | TSC1 |  |
| MME | MIR141 | SLC45A2 | KRAS |  |
| MYB | MIR205 | OCA2 | VEGFA |  |
| MYH11 | AR | CDKN1B | GLI1 |  |
| NBN | MIR483 | MYC | MMP9 |  |
| NF1 | RAD51L3-RFFL | CHEK2 | EWSR1 |  |
| NKAIN2 | FGFR3 | MSH2 | LOC111811965 |  |
| NPM1 | CDKN1B | CD63 | IFNG |  |
| NQO1 | MIR127 | MET | ENSG00000277654 |  |
| NUMA1 | MIR200A | S100B | PDGFRA |  |
| NUP214 | MRE11 | CTLA4 | QKI |  |
| NUP98 | RABL3 | IL24 | MYCN |  |
| PAX5 | CTNNA1 | MELTF | DMBT1 |  |
| PBX1 | TERT | ATM |  |  |
| PBX2 | NRAS | CD274 |  |  |
| PBX3 | PNLIP | MAGEA4 |  |  |
| PBX4 | MIR143 | ERCC2 |  |  |
| PICALM | MIR203A | MAGEA6 |  |  |
| PML | VHL | MDM2 |  |  |
| PSMD7 | TGFB1 | CSPG4 |  |  |
| PTPN11 | RAD51 | SAMMSON |  |  |
| RAP1GDS1 | MAP2K1 | CCND1 |  |  |
| RARA | MIR31 | DCT |  |  |
| RBM15 | MIR27A | CDKN1A |  |  |
| RUNX1 | CTRC | EZH2 |  |  |
| SAMD9 | MIR210 | MAGEA10 |  |  |
| SAMD9L | STAT3 | ERBB2 |  |  |
| SCLL | BAX | MAGEA2B |  |  |
| SET | MLH3 | MAGEA12 |  |  |
| SH3GL1 | PPARG | MIR21 |  |  |
| SLC20A1 | HNF1B | SOX10 |  |  |
| SSAV1 | IL6 | MSH6 |  |  |
| STAT5B | CPA1 | MHENCR |  |  |
| TAL1 | HOXB13 | MAGEA2 |  |  |
| TAL2 | MYC | RAC1 |  |  |
| TAM | MIR106B | BCL2 |  |  |
| TCL1A | ALK | MAGED1 |  |  |
| TCL1B | FANCC | MAGEA11 |  |  |
| TCL4 | IGF2 | RMEL3 |  |  |
| TCL6 | CDK4 | ATF1 |  |  |
| TCTA | MIR10B | CXCR4 |  |  |
| TERT | MIR93 | CREB1 |  |  |
| THRB | MIR10A | XPC |  |  |
| TLX1 | FANCE | IFNA2 |  |  |
| TLX2 | VEGFA | SMAD4 |  |  |
| TLX3 | MDM2 | EWSR1 |  |  |
| ZBTB16 | RBBP8 | MAGEC1 |  |  |
| AAT4 | INS | MMP2 |  |  |
| AF17 | SRC | PDCD1 |  |  |
| AF1Q | KIT | MAGEA8 |  |  |
| AF9 | EP300 | MAGEA9 |  |  |
| AMKL | BAP1 | DDB2 |  |  |
| ATA | FASLG | VEGFA |  |  |
| ATXPC | MIR29A | MAGEC2 |  |  |
| BSAP | PMS1 | APC |  |  |
| BTL | PTCH1 | ARAF |  |  |
| C13orf1 | CDKN1A | CRYBG1 |  |  |
| CAKUHED | IL1B | MAP2K2 |  |  |
| CALM | SMARCA4 | MAGED4B |  |  |
| CBFA2 | MIR18A | CDK6 |  |  |
| CBL2 | CASR | MAGEB2 |  |  |
| CBP | FANCM | AKT3 |  |  |
| CD10 | MIR30E | STK19 |  |  |
| CEBP | BMPR1A | SDHD |  |  |
| CHDSKM | MTOR | NBN |  |  |
| CML | MSH3 | MMP1 |  |  |
| D13S25 | PTF1A | CXCL1 |  |  |
| D6S231E | MIR23B | IFNG |  |  |
| D9S46E | BLM | MAGEB1 |  |  |
| DCML | MIR214 | IFNA1 |  |  |
| DIA4 | MIR204 | PIK3R1 |  |  |
| EEN | MIR182 | MAGED2 |  |  |
| ERBA2 | TSC2 | KISS1 |  |  |
| FACL6 | CDC73 | PTPN11 |  |  |
| FBP17 | TNF | BRIP1 |  |  |
| GF1 | IRF1 | ADAM7 |  |  |
| GHISID2 | MIRLET7D | ANKRD36B |  |  |
| GLVR1 | LRRC56 | MAPK1 |  |  |
| GRAF | MIR223 | STAT3 |  |  |
| HER4 | AURKA | MAGEB3 |  |  |
| HILDA | FLCN | POLE |  |  |
| HOX11 | RUNX1 | GPNMB |  |  |
| HOX11L1 | MIRLET7C | MTAP |  |  |
| HOX11L2 | BCL2 | IDH1 |  |  |
| HOX12 | ACVR1B | RAD51D |  |  |
| HOX1G | MIR335 | MAGEH1 |  |  |
| IMD22 | DICER1 | MAGEE1 |  |  |
| IMD37 | MIR451A | MIA3 |  |  |
| INP10 | MIR25 | FBXW7 |  |  |
| KRAS2 | EPHB2 | ABCB5 |  |  |
| LARG | KLF6 | MAGEA5P |  |  |
| LEU1 | MIR146B | NGFR |  |  |
| LEU2 | XRCC3 | PTK2 |  |  |
| MAR | ERCC6 | RAD51C |  |  |
| MOV34 | MIR183 | MIA2 |  |  |
| MRD42 | SOX9 | TNF |  |  |
| MRD58 | FBXW7 | PARP1 |  |  |
| MST | MIR429 | MTOR |  |  |
| MTS | MIR486-1 | MAGEE2 |  |  |
| MYL | MIR16-1 | ASIP |  |  |
| NBS1 | PIK3R1 | PTCH1 |  |  |
| NFTC | MIRLET7B | PWWP3A |  |  |
| NGL | MIR185 | TRPM1 |  |  |
| PBT | PRSS2 | PPP6C |  |  |
| PEBP2B | MIR140 | RET |  |  |
| PTP2C | MIR192 | MIR19A |  |  |
| RBTN1 | SLC22A18 | RECQL4 |  |  |
| RBTNL1 | MIR29C | MAGEB4 |  |  |
| SPEN | HNF4A | CASP3 |  |  |
| STWS | MIR373 | HIF1A |  |  |
| TBRS | TRB | LINC00328 | |  |
| TCBA | MIR19A | NME1 |  |  |
| TCL1 | HIF1A | CTAG2 |  |  |
| TCL5 | EGF | E2F1 |  |  |
| TCS1 | NOTCH1 | BARD1 |  |  |
| TEL | KCNJ11 | EGFR |  |  |
| THCYT3 | MIR499A | SDCBP |  |  |
| TLS | GATA6 | FAP |  |  |
| TML1 | FH | CXCL8 |  |  |
| TNG1 | MUC1 | ERCC3 |  |  |
| VRNF | CEL | EIF1AX |  |  |
| ZNF145 | EGFR-AS1 | ADAM29 |  |  |
| ACS2 | MIR122 | CDK2 |  |  |
| ALL3 | RNASEL | KDR |  |  |
| ALS19 | PRKN | IGF1R |  |  |
| ALS6 | RAD54L | FAS |  |  |
| AML1 | MMP2 | MIR17 |  |  |
| AT1 | PTGS2 | TNFRSF10B | |  |
| BCMSUN | MMP9 | SMARCB1 |  |  |
| CALLA | PPY | ING1 |  |  |
| CAN | AOPEP | MAGED4 |  |  |
| CLTH | BUB1B | SMARCA4 |  |  |
| DBM | GNAS | MBD4 |  |  |
| ERYF1 | MIR148A | MAGEF1 |  |  |
| EST2 | MIR100 | MFI2 |  |  |
| FAA4 | PPM1D | FGF4 |  |  |
| G17 | MIR150 | MAGEB17 |  |  |
| HESJAS | MIRLET7A1 | MAGEB18 |  |  |
| KIAA0382 | MIR128-2 | MAGEB6 |  |  |
| M7MLS1 | TMEM71 | MAGEB5 |  |  |
| MAL | CD274 | MAGEB10 |  |  |
| MASTC | CHGA | MAGEB16 |  |  |
| MDS | ZFHX3 | MAGEC3 |  |  |
| MIRAGE | SDHB | MAGEA9B |  |  |
| MONOMAC | MAPK1 | MAGEA5 |  |  |
| NEU | ROS1 | MAGE1 |  |  |
| NMOR1 | MSR1 | BARTS5 |  |  |
| NSLL | MIR107 | CD146 |  |  |
| OTT | MIR199B | CMM5 |  |  |
| PHL | ELAC2 | CMM8 |  |  |
| PLZF | MIR195 | CT10 |  |  |
| RASK2 | POLK | DAM10 |  |  |
| RHOM1 | MIR193B | DNS |  |  |
| RHOM2 | PPP2R1B | EST2 |  |  |
| RNX | MIR197 | GLM9 |  |  |
| RSTS1 | ABCC8 | KIAA1587 |  |  |
| SCL | BCL10 | KIAA1859 |  |  |
| SHP2 | CXCR4 | KURIS |  |  |
| SWS | BUB1 | LKB1 |  |  |
| TCL3 | CASP3 | MAGE4B |  |  |
| THC5 | DCC | MGSA |  |  |
| THR1 | PTPN11 | P16 |  |  |
| TNG2 | CTLA4 | TPDS2 |  |  |
| WSS | PKHD1 | CMM10 |  |  |
| ALL | MIR342 | CMM3 |  |  |
| CAIN | PLA2G2A | CMM6 |  |  |
| DKCA2 | DLC1 | DAM6 |  |  |
| ETM4 | SMAD7 | DAMAGE |  |  |
| HER2 | TYMS | GRO1 |  |  |
| IMD21 | BIRC5 | HSTF1 |  |  |
| LAP | MIR15B | LINC01212 |  |  |
| M7MLS2 | CXCL8 | MAGE10 |  |  |
| MKHK1 | ARID1A | MAGE11 |  |  |
| NEP | H19 | MAGE12 |  |  |
| NFE1 | ERCC2 | MAGE2 |  |  |
| NFNS | MIR26A1 | MAGE3 |  |  |
| NS | FAS | MAGE4A |  |  |
| NS1 | HNF1A | MAGE5 |  |  |
| PRTH | FANCD2 | MAGE6 |  |  |
| SCA49 | MIR106A | MAGE8 |  |  |
| SJS2 | PHB1 | MAGE9 |  |  |
| TTG2 | KDR | MAGED |  |  |
| VSCM2 | BSCL2 | MAGEE1 |  |  |
| CFC2 | CEACAM5 | MAGEH |  |  |
| CMT2T | SST | MAGEL1 |  |  |
| DKCB4 | IGF1R | MAP97 |  |  |
| IIAE9 | ABRAXAS1 | MED1 |  |  |
| JMML | MIR215 | MLA1 |  |  |
| VSCN2 | FHIT | MLM |  |  |
| XLTDA | SMARCB1 | MTS1 |  |  |
| METCDS | XRCC2 | MUC18 |  |  |
| PFBMFT1 | BCL2L1 | NS7 |  |  |
| RALD | MIR331 | PJS |  |  |
| SCA43 | MIR181A1 | SHEP2 |  |  |
| XLTT | MAP3K1 | SHEP3 |  |  |
| CMM9 | UCA1 | TCS1 |  |  |
| OES | MIR23A | TPDS1 |  |  |
| XLANP | MSMB | WS2A |  |  |
| HAEADA | IL10 | COMMAD |  |  |
| TP53 | MXI1 | DKCA2 |  |  |
| KMT2A | NPHP3 | HCA587 |  |  |
| SETBP1 | LOC111674472 | OCA1A |  |  |
| MLLT10 | WWOX | UVM2 |  |  |
| NRAS | CAV1 | ATN |  |  |
| IDH1 | RNF43 | CMM2 |  |  |
| ASXL1 | MIR142 | DKCB4 |  |  |
| STAT3 | FGFR1 | PFBMFT1 |  |  |
| CDKN2A | SMAD3 | CMM9 |  |  |
| MIR223 | IFNG |  |  |  |
| TET2 | MIR196A2 |  |  |  |
| MIR126 | ERBB3 |  |  |  |
| MYC | CFTR-AS1 |  |  |  |
| IDH2 | TNFRSF10B |  |  |  |
| CCND1 | MIR206 |  |  |  |
| NSD1 | NFKB1 |  |  |  |
| CSF3R | MIR224 |  |  |  |
| DDX41 | JUN |  |  |  |
| MIR155 | MIR137 |  |  |  |
| IKZF1 | IL1RN |  |  |  |
| INSL6 | LOC107303340 |  |  |  |
| BRAF | MIRLET7E |  |  |  |
| MIR203A | MIR98 |  |  |  |
| BCOR | MCC |  |  |  |
| MPL | GSTM1 |  |  |  |
| MIR17 | MIR22 |  |  |  |
| DNAJC21 | KRT7 |  |  |  |
| MIR16-1 | CTAG1B |  |  |  |
| MIR15A | TLR2 |  |  |  |
| MIR34A | NKX2-1 |  |  |  |
| SRSF2 | CD44 |  |  |  |
| FIP1L1 | IGF1 |  |  |  |
| PDGFRB | WT1 |  |  |  |
| IKZF3 | CYP2A6 |  |  |  |
| PTEN | RAF1 |  |  |  |
| THPO | MIR133B |  |  |  |
| MIR424 | IDH1 |  |  |  |
| MIR20A | MUC4 |  |  |  |
| ABCB1 | MIR128-1 |  |  |  |
| MDM2 | U2AF1 |  |  |  |
| RTEL1 | AKT2 |  |  |  |
| PDGFRA | CDKN2B |  |  |  |
| MIR181A2 | MIRLET7A3 |  |  |  |
| MIR143 | PDGFRB |  |  |  |
| MIR106B | TGFBR1 |  |  |  |
| TCF3 | HGF |  |  |  |
| HRAS | MT-CO1 |  |  |  |
| AFF1 | FGF2 |  |  |  |
| MECOM | ABCG2 |  |  |  |
| CSF3 | ERCC4 |  |  |  |
| WT1 | CDKN3 |  |  |  |
| MIR34B | LOC111674475 |  |  |  |
| MIRLET7C | ATR |  |  |  |
| MIR210 | GAST |  |  |  |
| RUNX1T1 | SDHD |  |  |  |
| U2AF1 | RB1CC1 |  |  |  |
| POT1 | JAK2 |  |  |  |
| KAT6A | ERCC1 |  |  |  |
| RTEL1-TNFRSF6B | HMMR |  |  |  |
| CHEK2 | TGFA |  |  |  |
| MIR320A | KRT19 |  |  |  |
| CRLF2 | LOC111674477 |  |  |  |
| MIR204 | PLAU |  |  |  |
| MIR27A | NTHL1 |  |  |  |
| TGM6 | PTK2 |  |  |  |
| NOTCH1 | LOC113664106 |  |  |  |
| MIR10A | PDGFRA |  |  |  |
| ERCC6L2 | PARP1 |  |  |  |
| CSF2 | CDX2 |  |  |  |
| SF3B1 | CDK6 |  |  |  |
| ARL11 | PTRH2 |  |  |  |
| MIR152 | CCK |  |  |  |
| MRTFA | RHOA |  |  |  |
| IGHG1 | DNMT1 |  |  |  |
| IL3 | LOC113633877 |  |  |  |
| P2RX7 | SCT |  |  |  |
| RPS15 | CEACAM6 |  |  |  |
| MIR29A | NTRK1 |  |  |  |
| IGHV3-21 | SDHC |  |  |  |
| RAF1 | FLT1 |  |  |  |
| IFNA1 | HNRNPUL2-BSCL2 |  |  |  |
| MIR145 | MIR372 |  |  |  |
| SETD2 | ABL1 |  |  |  |
| MIR107 | GSTP1 |  |  |  |
| MPO | PRKD1 |  |  |  |
| MTHFR | MALAT1 |  |  |  |
| FGFR1 | RNASE1 |  |  |  |
| BRCA2 | CEACAM3 |  |  |  |
| CASP3 | REG3A |  |  |  |
| MLLT1 | NFKBIA |  |  |  |
| CDKN2B | MIR30D |  |  |  |
| PRKAR1A | ABCB1 |  |  |  |
| SEPTIN9 | GCG |  |  |  |
| MIR331 | CCAT1 |  |  |  |
| CD38 | CDK2 |  |  |  |
| IL6 | MIR191 |  |  |  |
| MIR130B | MIR181B1 |  |  |  |
| GFI1 | TNFSF10 |  |  |  |
| TBL1XR1 | CASC2 |  |  |  |
| ELANE | MAPK3 |  |  |  |
| TRG | CXCL12 |  |  |  |
| IGH | RAD54B |  |  |  |
| CD33 | TERC |  |  |  |
| CD7 | NQO2 |  |  |  |
| C11orf65 | NF2 |  |  |  |
| AKT1 | MUC5AC |  |  |  |
| TRD | PDGFRL |  |  |  |
| ANPEP | MXRA5 |  |  |  |
| CD34 | SP1 |  |  |  |
| JAK3 | FLT4 |  |  |  |
| ZAP70 | HFE |  |  |  |
| BCL2L1 | STAT1 |  |  |  |
| MIRLET7A3 | TYMP |  |  |  |
| TNF | SYP |  |  |  |
| STAT5A | SDHA |  |  |  |
| IRF2BP2 | IGFBP3 |  |  |  |
| KITLG | MAP3K8 |  |  |  |
| NUDT15 | SMAD2 |  |  |  |
| JAK1 | DMD |  |  |  |
| BCL6 | PTPRJ |  |  |  |
| EP300 | TWIST1 |  |  |  |
| MIRLET7D | HOTAIR |  |  |  |
| NABP1 | MIRLET7I |  |  |  |
| IL2 | PGR |  |  |  |
| DNTT | KRT20 |  |  |  |
| MEIS1 | REG1A |  |  |  |
| LRRC56 | KRT8 |  |  |  |
| STIL | GLI1 |  |  |  |
| EPOR | KLK3 |  |  |  |
| ABCC1 | MIR199A1 |  |  |  |
| CALR | HERC2 |  |  |  |
| MIR181A1HG | MT-ND1 |  |  |  |
| CASP8 | SUFU |  |  |  |
| ERBB3 | VEGFC |  |  |  |
| FLI1 | AXIN1 |  |  |  |
| MAP2K1 | CCR6 |  |  |  |
| CD19 | PLAUR |  |  |  |
| CYP2C19 | MMP7 |  |  |  |
| AFDN | FN1 |  |  |  |
| JUN | MIR196B |  |  |  |
| VHL | MAPK8 |  |  |  |
| BMI1 | GCK |  |  |  |
| CDKN1A | ALB |  |  |  |
| LYN | FOXO1 |  |  |  |
| ABCG2 | HSP90AA1 |  |  |  |
| ANXA5 | XRCC1 |  |  |  |
| NALT1 | MGMT |  |  |  |
| IL7 | SERPINA3 |  |  |  |
| NCAM1 | PTHLH |  |  |  |
| XIAP | CDKN1C |  |  |  |
| CEP72 | SNAI1 |  |  |  |
| CDKN1B | TP73 |  |  |  |
| RRAS | TGFB2 |  |  |  |
| CREB1 | CCL2 |  |  |  |
| CXCR4 | ETV6 |  |  |  |
| TRB | GALNT12 |  |  |  |
| RECQL4 | NEK8 |  |  |  |
| DCK | MIR181A2 |  |  |  |
| MAPK1 | NQO1 |  |  |  |
| RB1 | ITGB1 |  |  |  |
| LOC107303340 | FZD3 |  |  |  |
| EGFR | MCL1 |  |  |  |
| GSTM1 | EZH2 |  |  |  |
| ARID5B | ESR2 |  |  |  |
| CSF1R | MIR449A |  |  |  |
| CBFA2T3 | SLC2A1 |  |  |  |
| EZH2 | PVT1 |  |  |  |
| FLVCR1 | MYCN |  |  |  |
| IL10 | TFF1 |  |  |  |
| LUNAR1 | GRP |  |  |  |
| VEGFA | POT1 |  |  |  |
| STAT1 | TOP2A |  |  |  |
| IRF4 | BCAR1 |  |  |  |
| PRAME | XIAP |  |  |  |
| IL6ST | MKI67 |  |  |  |
| CYCS | SNAI2 |  |  |  |
| FBXW7 | H2AC18 |  |  |  |
| CTNNB1 | PRKAR1A |  |  |  |
| PTPRC | MIR30A |  |  |  |
| CD5 | CCNA2 |  |  |  |
| BIRC5 | MTHFR |  |  |  |
| CDK4 | RECQL4 |  |  |  |
| IL4 | PROM1 |  |  |  |
| IL2RA | MAP2K2 |  |  |  |
| FLVCR2 | CYCS |  |  |  |
| CCND2 | GREM1 |  |  |  |
| ABL2 | SERPINA1 |  |  |  |
| CDK2 | MIR24-2 |  |  |  |
| LEF1 | NEUROD1 |  |  |  |
| FAS | GATA2 |  |  |  |
| ADA | MT-CYB |  |  |  |
| IL3RA | PAX4 |  |  |  |
| RAD51 | MEG3 |  |  |  |
| TPMT | RAC1 |  |  |  |
| LNP1 | IGF2R |  |  |  |
| PIK3CA | PAX6 |  |  |  |
| CASP9 | GNA11 |  |  |  |
| FOXO3 | DNMT3A |  |  |  |
| RUNX3 | ZEB1 |  |  |  |
| TNFSF10 | GNG3 |  |  |  |
| KMT2C | ATRX |  |  |  |
| SPI1 | SETD2 |  |  |  |
| MIR125A | PIK3CG |  |  |  |
| CD22 | MIR375 |  |  |  |
| BCL11B | CDH2 |  |  |  |
| IL7R | PLK1 |  |  |  |
| CNOT3 | MIR296 |  |  |  |
| TRA | PNLIPRP1 |  |  |  |
| BTK | MAP3K6 |  |  |  |
| EPO | MIR181C |  |  |  |
| IL1B | SBDS |  |  |  |
| CXCL12 | DPYD |  |  |  |
| TYMS | MMP14 |  |  |  |
| RUNX2 | CNOT1 |  |  |  |
| RPS14 | LEF1 |  |  |  |
| BCL2L11 | LMNA |  |  |  |
| ITGAM | AMER1 |  |  |  |
| DLEU2L | CCAT2 |  |  |  |
| MSH2 | MMP1 |  |  |  |
| CXCL8 | RASSF1 |  |  |  |
| LIG4 | MIR193A |  |  |  |
| PMAIP1 | PSCA |  |  |  |
| MIR92A2 | DDB2 |  |  |  |
| MIR106A | TIMP2 |  |  |  |
| MIR20B | CSF2 |  |  |  |
| BRD4 | TIMP1 |  |  |  |
| IGHV4-34 | CCKBR |  |  |  |
| RPL5 | ING1 |  |  |  |
| SBDS | GDF15 |  |  |  |
| HLA-C | HDAC1 |  |  |  |
| CYP1A1 | MUC6 |  |  |  |
| MIR19B2 | IDH2 |  |  |  |
| SYK | MIR510 |  |  |  |
| BRCA1 | INS-IGF2 |  |  |  |
| CDK6 | CASP9 |  |  |  |
| SRC | RFX6 |  |  |  |
| FANCC | CTSB |  |  |  |
| FANCA | ANXA5 |  |  |  |
| LOC107980440 | TLR4 |  |  |  |
| CD44 | AKT3 |  |  |  |
| MVP | WRAP53 |  |  |  |
| TERC | CCND2 |  |  |  |
| PRAM1 | CD4 |  |  |  |
| MAPK8 | PTPN12 |  |  |  |
| DHFR | FGFR4 |  |  |  |
| CSF1 | MIR9-1 |  |  |  |
| KDM5A | BMI1 |  |  |  |
| TFRC | MIR139 |  |  |  |
| ANKRD26 | SOD2 |  |  |  |
| CTLA4 | MIR196A1 |  |  |  |
| FES | RPS6KB1 |  |  |  |
| SLC19A1 | THBS1 |  |  |  |
| IRF8 | COX4I2 |  |  |  |
| SLC20A2 | OGG1 |  |  |  |
| XPO1 | CDKN2B-AS1 |  |  |  |
| HDAC9 | IL2 |  |  |  |
| APC | CYP1A1 |  |  |  |
| ITGAX | DNAJC21 |  |  |  |
| MYCN | AREG |  |  |  |
| PARP1 | SLMAP |  |  |  |
| HSP90AA1 | FANCG |  |  |  |
| DDX3X | CCNE1 |  |  |  |
| PIK3CG | PDCD1 |  |  |  |
| PBXIP1 | PRKCA |  |  |  |
| GATA3 | MITF |  |  |  |
| FOS | OPCML |  |  |  |
| CD4 | NRP1 |  |  |  |
| CDK1 | TNFRSF10A |  |  |  |
| CBFA2T2 | CD82 |  |  |  |
| MYD88 | GAPDH |  |  |  |
| CCNA2 | MSLN |  |  |  |
| APAF1 | ENO2 |  |  |  |
| IFNG | ABCC1 |  |  |  |
| MIR142 | TOP1 |  |  |  |
| LZTR1 | BID |  |  |  |
| WRAP53 | CRP |  |  |  |
| CD274 | SPP1 |  |  |  |
| MIR21 | ICOSLG |  |  |  |
| SOS1 | BAK1 |  |  |  |
| CRKL | EIF2AK3 |  |  |  |
| CCR6 | MUC16 |  |  |  |
| CD2 | GPC3 |  |  |  |
| TNFRSF8 | MIR29B1 |  |  |  |
| MIR221 | MIR125B1 |  |  |  |
| RPN1 | LPL |  |  |  |
| TP73 | TCF7L2 |  |  |  |
| SRP54 | MIR424 |  |  |  |
| DNMT1 | MT-CO2 |  |  |  |
| ITGAL | DROSHA |  |  |  |
| CDA | CCL5 |  |  |  |
| MTOR | TGFB3 |  |  |  |
| CD40 | TP63 |  |  |  |
| BUB1B | CTAG2 |  |  |  |
| FIM3 | HSPB1 |  |  |  |
| FIM1 | CYP19A1 |  |  |  |
| HNRNPH1 | CDK1 |  |  |  |
| ITGA4 | LGALS3 |  |  |  |
| KIR3DL1 | CDKN2C |  |  |  |
| SH2B3 | MIR324 |  |  |  |
| FASLG | MIR99A |  |  |  |
| KDR | MIR532 |  |  |  |
| IFNA2 | HBEGF |  |  |  |
| CNTN2 | GAS5 |  |  |  |
| MIR9-1 | E2F1 |  |  |  |
| MIR199B | CD8A |  |  |  |
| EWSR1 | VDR |  |  |  |
| IL11 | SERPINB5 |  |  |  |
|  | NFE2L2 |  |  |  |
|  | NEAT1 |  |  |  |
|  | FLT3 |  |  |  |
|  | S100A4 |  |  |  |
|  | SKP2 |  |  |  |
|  | KDM4C |  |  |  |
|  | PKM |  |  |  |
|  | MYO1B |  |  |  |
|  | SHH |  |  |  |
|  | ELANE |  |  |  |
|  | APOE |  |  |  |
|  | NCAM1 |  |  |  |
|  | KRT18 |  |  |  |
|  | MAX |  |  |  |
|  | MUC2 |  |  |  |
|  | RELA |  |  |  |
|  | XPC |  |  |  |
|  | HDAC9 |  |  |  |
|  | GADD45A |  |  |  |
|  | RHBDF2 |  |  |  |
|  | NDRG1 |  |  |  |
|  | FOXE1 |  |  |  |
|  | MIF |  |  |  |
|  | AFP |  |  |  |
|  | MIR149 |  |  |  |
|  | CFLAR |  |  |  |
|  | NCOA3 |  |  |  |
|  | EDNRA |  |  |  |
|  | EZR |  |  |  |
|  | COL1A1 |  |  |  |
|  | EPHA2 |  |  |  |
|  | NPM1 |  |  |  |
|  | MIR330 |  |  |  |
|  | CSF3 |  |  |  |
|  | RBFOX1 |  |  |  |
|  | SSTR2 |  |  |  |
|  | NAT2 |  |  |  |
|  | PCNA |  |  |  |
|  | FAN1 |  |  |  |
|  | MIR101-1 |  |  |  |
|  | CLPS |  |  |  |
|  | RELB |  |  |  |
|  | HABP2 |  |  |  |
|  | NOS2 |  |  |  |
|  | HMOX1 |  |  |  |
|  | CCNB1 |  |  |  |
|  | XPA |  |  |  |
|  | MTAP |  |  |  |
|  | MIR30B |  |  |  |
|  | WRN |  |  |  |
|  | YY1 |  |  |  |
|  | PLG |  |  |  |
|  | MIR144 |  |  |  |
|  | MAP2K4 |  |  |  |
|  | MIR338 |  |  |  |
|  | EFL1 |  |  |  |
|  | FGF1 |  |  |  |
|  | XIST |  |  |  |
|  | MIR152 |  |  |  |
|  | SMARCE1 |  |  |  |
|  | CTSD |  |  |  |
|  | TUG1 |  |  |  |
|  | RARB |  |  |  |
|  | CLDN4 |  |  |  |
|  | SPINT2 |  |  |  |
|  | TCERG1 |  |  |  |
|  | CAGE1 |  |  |  |
|  | SPARC |  |  |  |
|  | ERCC3 |  |  |  |
|  | EWSR1 |  |  |  |
|  | FOXP3 |  |  |  |
|  | SRGAP1 |  |  |  |
|  | MIR181B2 |  |  |  |
|  | MAPK14 |  |  |  |
|  | YAP1 |  |  |  |
|  | GSTT1 |  |  |  |
|  | HULC |  |  |  |
|  | CTNND1 |  |  |  |
|  | GSK3B |  |  |  |
|  | ERBB4 |  |  |  |
|  | CASP7 |  |  |  |
|  | GACAT2 |  |  |  |
|  | HPSE |  |  |  |
|  | MIR24-1 |  |  |  |
|  | BSG |  |  |  |
|  | HLA-B |  |  |  |
|  | HOTTIP |  |  |  |
|  | CREB1 |  |  |  |
|  | VIP |  |  |  |
|  | MIR320A |  |  |  |
|  | DKK1 |  |  |  |
|  | MIR370 |  |  |  |
|  | FOS |  |  |  |
|  | CCND3 |  |  |  |
|  | YARS1 |  |  |  |
|  | FANCA |  |  |  |
|  | ERG |  |  |  |
|  | KCNQ1OT1 |  |  |  |
|  | CXCR2 |  |  |  |
|  | TFF2 |  |  |  |
|  | HLA-A |  |  |  |
|  | CCN2 |  |  |  |
|  | FSCN1 |  |  |  |
|  | SRP54 |  |  |  |
|  | EIF4EBP1 |  |  |  |
|  | LINC-ROR |  |  |  |
|  | ADH1B |  |  |  |
|  | CHEK1 |  |  |  |
|  | RIPK1 |  |  |  |
|  | NPHP3-ACAD11 |  |  |  |
|  | ANXA2 |  |  |  |
|  | ENO1 |  |  |  |
|  | PCA3 |  |  |  |
|  | CD24 |  |  |  |
|  | CD34 |  |  |  |
|  | CD36 |  |  |  |
|  | HSPA5 |  |  |  |
|  | MIR423 |  |  |  |
|  | LCN2 |  |  |  |
|  | CELA3B |  |  |  |
|  | CYP2E1 |  |  |  |
|  | BMP6 |  |  |  |
|  | RPS20 |  |  |  |
|  | AIP |  |  |  |
|  | NTS |  |  |  |
|  | CALCA |  |  |  |
|  | SPRY4-IT1 |  |  |  |
|  | KLF4 |  |  |  |
|  | ILK |  |  |  |
|  | POMC |  |  |  |
|  | GSTM3 |  |  |  |
|  | TMEM127 |  |  |  |
|  | BAD |  |  |  |
|  | LOX |  |  |  |
|  | ACTB |  |  |  |
|  | IL4 |  |  |  |
|  | MIR92A1 |  |  |  |
|  | MIR361 |  |  |  |
|  | SMO |  |  |  |
|  | PECAM1 |  |  |  |
|  | BCL2L11 |  |  |  |
|  | MIR130B |  |  |  |
|  | NME1 |  |  |  |
|  | SERPINE1 |  |  |  |
|  | NRG1 |  |  |  |
|  | NEUROG3 |  |  |  |
|  | BMP2 |  |  |  |
|  | INSR |  |  |  |
|  | TUSC3 |  |  |  |
|  | MIR337 |  |  |  |
|  | ALDH2 |  |  |  |
|  | MIR32 |  |  |  |
|  | ENG |  |  |  |
|  | PAX8 |  |  |  |
|  | NFKB2 |  |  |  |
|  | PRKCD |  |  |  |
|  | CPB1 |  |  |  |
|  | ITGB4 |  |  |  |
|  | TIMP3 |  |  |  |
|  | PDGFB |  |  |  |
|  | VIM |  |  |  |
|  | MME |  |  |  |
|  | MIR497 |  |  |  |
|  | CASP1 |  |  |  |
|  | LINC00673 |  |  |  |
|  | ALOX5 |  |  |  |
|  | FOXM1 |  |  |  |
|  | CLPTM1L |  |  |  |
|  | DNMT3B |  |  |  |
|  | HLA-DRB1 |  |  |  |
|  | IGF2BP3 |  |  |  |
|  | MIR455 |  |  |  |
|  | MIR590 |  |  |  |
|  | ICAM1 |  |  |  |
|  | MIR29B2 |  |  |  |
|  | MIR186 |  |  |  |
|  | VTN |  |  |  |
|  | PKD1 |  |  |  |
|  | S100A6 |  |  |  |
|  | PLA2G1B |  |  |  |
|  | MIR133A1 |  |  |  |
|  | POSTN |  |  |  |
|  | TCF4 |  |  |  |
|  | HSPA4 |  |  |  |
|  | UBR1 |  |  |  |
|  | CREBBP |  |  |  |
|  | HLA-DQB1 |  |  |  |
|  | GZMB |  |  |  |
|  | RECQL |  |  |  |
|  | NOTCH3 |  |  |  |
|  | SCTR |  |  |  |
|  | BMP4 |  |  |  |
|  | DCK |  |  |  |
|  | GRPR |  |  |  |
|  | ITGA6 |  |  |  |
|  | CDC42 |  |  |  |
|  | CEBPA |  |  |  |
|  | TRIP13 |  |  |  |
|  | DKC1 |  |  |  |
|  | SOX2 |  |  |  |
|  | NSD1 |  |  |  |
|  | MTUS1 |  |  |  |
|  | JAK1 |  |  |  |
|  | MST1R |  |  |  |
|  | CYP1B1 |  |  |  |
|  | AFAP1-AS1 |  |  |  |
|  | GPER1 |  |  |  |
|  | LEP |  |  |  |
|  | SOS1 |  |  |  |
|  | MIR216A |  |  |  |
|  | LUCAT1 |  |  |  |
|  | CYP17A1 |  |  |  |
|  | KLF10 |  |  |  |
|  | FOXA2 |  |  |  |
|  | NES |  |  |  |
|  | KLF5 |  |  |  |
|  | MIR132 |  |  |  |
|  | KRT5 |  |  |  |
|  | TGM2 |  |  |  |
|  | CA9 |  |  |  |
|  | IL24 |  |  |  |
|  | BDNF |  |  |  |
|  | SLC29A1 |  |  |  |
|  | PDPN |  |  |  |
|  | AXL |  |  |  |
|  | ASXL1 |  |  |  |
|  | LIF |  |  |  |
|  | CLU |  |  |  |
|  | HMGA1 |  |  |  |
|  | IL1A |  |  |  |
|  | PRF1 |  |  |  |
|  | TH2LCRR |  |  |  |
|  | MIR135A1 |  |  |  |
|  | CHUK |  |  |  |
|  | MIR328 |  |  |  |
|  | APEX1 |  |  |  |
|  | CD28 |  |  |  |
|  | FASN |  |  |  |
|  | RRM1 |  |  |  |
|  | MIR138-1 |  |  |  |
|  | POU5F1 |  |  |  |
|  | MIR376A1 |  |  |  |
|  | KLRK1 |  |  |  |
|  | ETV4 |  |  |  |
|  | IFNA1 |  |  |  |
|  | WNT10B |  |  |  |
|  | PPDPF |  |  |  |
|  | MAGEC2 |  |  |  |
|  | MIR574 |  |  |  |
|  | DAXX |  |  |  |
|  | SIRT1 |  |  |  |
|  | PAK1 |  |  |  |
|  | TTK |  |  |  |
|  | NTRK3 |  |  |  |
|  | PRKACA |  |  |  |
|  | S100A2 |  |  |  |
|  | TRIM28 |  |  |  |
|  | RARA |  |  |  |
|  | MIR151A |  |  |  |
|  | CCNE2 |  |  |  |
|  | CALR |  |  |  |
|  | MPO |  |  |  |
|  | MC1R |  |  |  |
|  | STAT5B |  |  |  |
|  | IRS1 |  |  |  |
|  | ADH1C |  |  |  |
|  | CD9 |  |  |  |
|  | HLA-DQA1 |  |  |  |
|  | E2F3 |  |  |  |
|  | MIR301A |  |  |  |
|  | FOXO3 |  |  |  |
|  | MPL |  |  |  |
|  | MAFA |  |  |  |
|  | SHC1 |  |  |  |
|  | SDHAF2 |  |  |  |
|  | MIR211 |  |  |  |
|  | WNT5A |  |  |  |
|  | HES1 |  |  |  |
|  | TG |  |  |  |
|  | GSK3A |  |  |  |
|  | MMP3 |  |  |  |
|  | MIR490 |  |  |  |
|  | ETS1 |  |  |  |
|  | CYTOR |  |  |  |
|  | BECN1 |  |  |  |
|  | IL15 |  |  |  |
|  | MMP11 |  |  |  |
|  | MIR103A1 |  |  |  |
|  | FOLH1 |  |  |  |
|  | HMGB1 |  |  |  |
|  | MTA1 |  |  |  |
|  | CCL3 |  |  |  |
|  | PTPRN2 |  |  |  |
|  | BLK |  |  |  |
|  | MIR675 |  |  |  |
|  | RXRA |  |  |  |
|  | MIR485 |  |  |  |
|  | DHFR |  |  |  |
|  | MIR33A |  |  |  |
|  | IGFBP2 |  |  |  |
|  | CDR1-AS |  |  |  |
|  | MIR202 |  |  |  |
|  | FGF7 |  |  |  |
|  | IFI27 |  |  |  |
|  | IL18 |  |  |  |
|  | PNLIPRP3 |  |  |  |
|  | IAPP |  |  |  |
|  | ETV1 |  |  |  |
|  | GCLC |  |  |  |
|  | MINPP1 |  |  |  |
|  | CA2 |  |  |  |
|  | CTNNA2 |  |  |  |
|  | MIR28 |  |  |  |
|  | JAG1 |  |  |  |
|  | ST14 |  |  |  |
|  | TFE3 |  |  |  |
|  | ELAVL1 |  |  |  |
|  | RHOB |  |  |  |
|  | TNFRSF1A |  |  |  |
|  | TET2 |  |  |  |
|  | BCAR3 |  |  |  |
|  | EBAG9 |  |  |  |
|  | GGT1 |  |  |  |
|  | CD40 |  |  |  |
|  | FBN1 |  |  |  |
|  | DDR2 |  |  |  |
|  | CBL |  |  |  |
|  | PTPRC |  |  |  |
|  | SEMA4A |  |  |  |
|  | CLDN7 |  |  |  |
|  | RRM2 |  |  |  |
|  | CCKAR |  |  |  |
|  | DRAIC |  |  |  |
|  | TGFBR3 |  |  |  |
|  | CDH5 |  |  |  |
|  | IL17A |  |  |  |
|  | BNIP3 |  |  |  |
|  | GHRL |  |  |  |
|  | CAT |  |  |  |
|  | MIR212 |  |  |  |
|  | B2M |  |  |  |
|  | GLIS3 |  |  |  |
|  | TH2-LCR |  |  |  |
|  | EPHB4 |  |  |  |
|  | GRB2 |  |  |  |
|  | PCAT6 |  |  |  |
|  | SULF1 |  |  |  |
|  | SOX4 |  |  |  |
|  | XBP1 |  |  |  |
|  | MIR582 |  |  |  |
|  | CDH3 |  |  |  |
|  | ZEB2 |  |  |  |
|  | CSF1R |  |  |  |
|  | CRNDE |  |  |  |
|  | PCAT7 |  |  |  |
|  | VEGFD |  |  |  |
|  | RALA |  |  |  |
|  | RAD51B |  |  |  |
|  | LRP5 |  |  |  |
|  | STAT6 |  |  |  |
|  | CXCL1 |  |  |  |
|  | KITLG |  |  |  |
|  | GHET1 |  |  |  |
|  | LINC00261 |  |  |  |
|  | FCGR2A |  |  |  |
|  | RUNX3 |  |  |  |
|  | MVP |  |  |  |
|  | HMGA2 |  |  |  |
|  | SF3B1 |  |  |  |
|  | PLCG1 |  |  |  |
|  | DAPK1 |  |  |  |
|  | GDNF |  |  |  |
|  | DMBT1 |  |  |  |
|  | STX1A |  |  |  |
|  | CASC11 |  |  |  |
|  | ITPR3 |  |  |  |
|  | KLF11 |  |  |  |
|  | IKBKB |  |  |  |
|  | SPOP |  |  |  |
|  | NKX6-1 |  |  |  |
|  | SNHG15 |  |  |  |
|  | GATA3 |  |  |  |
|  | BRD4 |  |  |  |
|  | MIR124-1 |  |  |  |
|  | EPHA3 |  |  |  |
|  | GATA4 |  |  |  |
|  | THPO |  |  |  |
|  | FAM3C |  |  |  |
|  | MIR625 |  |  |  |
|  | GATA1 |  |  |  |
|  | BIRC3 |  |  |  |
|  | IL2RA |  |  |  |
|  | CELA1 |  |  |  |
|  | SEC23B |  |  |  |
|  | COPS5 |  |  |  |
|  | MIR365A |  |  |  |
|  | POLH |  |  |  |
|  | RTEL1 |  |  |  |
|  | CCR7 |  |  |  |
|  | MIR345 |  |  |  |
|  | REL |  |  |  |
|  | FANCF |  |  |  |
|  | ANXA1 |  |  |  |
|  | MIR148B |  |  |  |
|  | PML |  |  |  |
|  | SQSTM1 |  |  |  |
|  | NOTCH2 |  |  |  |
|  | ONECUT1 |  |  |  |
|  | CSF1 |  |  |  |
|  | STAT5A |  |  |  |
|  | HK2 |  |  |  |
|  | CDC25A |  |  |  |
|  | CD80 |  |  |  |
|  | IL7 |  |  |  |
|  | PXN |  |  |  |
|  | ID1 |  |  |  |
|  | GHRH |  |  |  |
|  | LNCRNA-ATB |  |  |  |
|  | ABCC2 |  |  |  |
|  | CBR4 |  |  |  |
|  | ADAM17 |  |  |  |
|  | HDAC4 |  |  |  |
|  | PIK3R2 |  |  |  |
|  | SNCG |  |  |  |
|  | NAT1 |  |  |  |
|  | SDC1 |  |  |  |
|  | NOD2 |  |  |  |
|  | NORAD |  |  |  |
|  | S100A8 |  |  |  |
|  | F2 |  |  |  |
|  | MDM4 |  |  |  |
|  | EGR1 |  |  |  |
|  | WNT7B |  |  |  |
|  | YBX1 |  |  |  |
|  | ITGAV |  |  |  |
|  | MAGEA1 |  |  |  |
|  | IL6R |  |  |  |
|  | PRL |  |  |  |
|  | TPX2 |  |  |  |
|  | MIR615 |  |  |  |
|  | IFNA2 |  |  |  |
|  | IRS2 |  |  |  |
|  | TOE1 |  |  |  |
|  | JUP |  |  |  |
|  | PEBP1 |  |  |  |
|  | MAPK10 |  |  |  |
|  | CYP2D6 |  |  |  |
|  | MIR452 |  |  |  |
|  | CASC19 |  |  |  |
|  | STMN1 |  |  |  |
|  | ECT2 |  |  |  |
|  | BCR |  |  |  |
|  | NUPR1 |  |  |  |
|  | INSM1 |  |  |  |
|  | BCL6 |  |  |  |
|  | TYR |  |  |  |
|  | DIABLO |  |  |  |
|  | DELEC1 |  |  |  |
|  | KRT14 |  |  |  |
|  | ADAM10 |  |  |  |
|  | SEMA3A |  |  |  |
|  | ARAF |  |  |  |
|  | ADIPOQ |  |  |  |
|  | LGALS1 |  |  |  |
|  | RECK |  |  |  |
|  | NGF |  |  |  |
|  | SIRT6 |  |  |  |
|  | MIR371A |  |  |  |
|  | GCGR |  |  |  |
|  | PDPK1 |  |  |  |
|  | KLK10 |  |  |  |
|  | OLFM4 |  |  |  |
|  | HNF1A-AS1 |  |  |  |
|  | PTPN3 |  |  |  |
|  | CADM1 |  |  |  |
|  | GAD2 |  |  |  |
|  | KMT2A |  |  |  |
|  | TACC1 |  |  |  |
|  | POLR1C |  |  |  |
|  | HDAC2 |  |  |  |
|  | ABCB4 |  |  |  |
|  | APOC2 |  |  |  |
|  | GPT |  |  |  |
|  | NCR1 |  |  |  |
|  | DPP4 |  |  |  |
|  | ACE |  |  |  |
|  | STUB1 |  |  |  |
|  | PIK3CB |  |  |  |
|  | LIG4 |  |  |  |
|  | SLC6A14 |  |  |  |
|  | F2RL1 |  |  |  |
|  | GNAQ |  |  |  |
|  | MIR219A1 |  |  |  |
|  | MAGEA3 |  |  |  |
|  | PRODH |  |  |  |
|  | TNFRSF6B |  |  |  |
|  | NCOR2 |  |  |  |
|  | ALDH1A1 |  |  |  |
|  | PPP2R1A |  |  |  |
|  | MYD88 |  |  |  |
|  | FOXA1 |  |  |  |
|  | SRPRA |  |  |  |
|  | MIR92A2 |  |  |  |
|  | CCL1 |  |  |  |
|  | FGF10 |  |  |  |
|  | MIR425 |  |  |  |
|  | AMY2A |  |  |  |
|  | FADD |  |  |  |
|  | FANCI |  |  |  |
|  | COL1A2 |  |  |  |
|  | ACTN4 |  |  |  |
|  | CXCL10 |  |  |  |
|  | MIR198 |  |  |  |
|  | PAX5 |  |  |  |
|  | E2F2 |  |  |  |
|  | BIRC2 |  |  |  |
|  | PLK2 |  |  |  |
|  | IL13 |  |  |  |
|  | DAB2IP |  |  |  |
|  | SOD1 |  |  |  |
|  | CEP290 |  |  |  |
|  | TMPRSS2 |  |  |  |
|  | KCNQ1 |  |  |  |
|  | SLC11A1 |  |  |  |
|  | TNFRSF1B |  |  |  |
|  | CCL4 |  |  |  |
|  | PIK3R3 |  |  |  |
|  | B3GALT5 |  |  |  |
|  | CALB2 |  |  |  |
|  | TEK |  |  |  |
|  | PDCD4 |  |  |  |
|  | PCAT14 |  |  |  |
|  | CHD1 |  |  |  |
|  | GJA1 |  |  |  |
|  | AHR |  |  |  |
|  | H2AX |  |  |  |
|  | CYP3A4 |  |  |  |
|  | PTGS1 |  |  |  |
|  | UHRF1 |  |  |  |
|  | RALB |  |  |  |
|  | ATP8B1 |  |  |  |
|  | ITGA3 |  |  |  |
|  | NTPCR |  |  |  |
|  | PAWR |  |  |  |
|  | EDN1 |  |  |  |
|  | CIB1 |  |  |  |
|  | SSX2 |  |  |  |
|  | APPL1 |  |  |  |
|  | TLR9 |  |  |  |
|  | RMRP |  |  |  |
|  | COL7A1 |  |  |  |
|  | MIRLET7F1 |  |  |  |
|  | CDC25C |  |  |  |
|  | TRPV4 |  |  |  |
|  | MYLK |  |  |  |
|  | ALCAM |  |  |  |
|  | SHBG |  |  |  |
|  | MAGEA4 |  |  |  |
|  | SNHG1 |  |  |  |
|  | ANGPT2 |  |  |  |
|  | GNRH1 |  |  |  |
|  | SLC5A8 |  |  |  |
|  | FABP4 |  |  |  |
|  | SRP19 |  |  |  |
|  | WNT1 |  |  |  |
|  | LDOC1 |  |  |  |
|  | SPTAN1 |  |  |  |
|  | SLC16A1 |  |  |  |
|  | SLC39A4 |  |  |  |
|  | LINC00511 |  |  |  |
|  | SF3B2 |  |  |  |
|  | CCNG1 |  |  |  |
|  | ZFAS1 |  |  |  |
|  | WNT3A |  |  |  |
|  | RABIF |  |  |  |
|  | BRMS1 |  |  |  |
|  | LOC110121471 |  |  |  |
|  | MYB |  |  |  |
|  | MACROD1 |  |  |  |
|  | APAF1 |  |  |  |
|  | ITGA5 |  |  |  |
|  | ALOX12 |  |  |  |
|  | MIR340 |  |  |  |
|  | PYY |  |  |  |
|  | ISL1 |  |  |  |
|  | PROX1 |  |  |  |
|  | ITGAE |  |  |  |
|  | NR5A2 |  |  |  |
|  | MIR1247 |  |  |  |
|  | BTC |  |  |  |
|  | RPS19 |  |  |  |
|  | KCNN4 |  |  |  |
|  | MIR873 |  |  |  |
|  | CXCR1 |  |  |  |
|  | DANCR |  |  |  |
|  | FLI1 |  |  |  |
|  | NAB2 |  |  |  |
|  | IER3 |  |  |  |
|  | CD226 |  |  |  |
|  | DLL1 |  |  |  |
|  | ODC1 |  |  |  |
|  | TMPRSS15 |  |  |  |
|  | CDK12 |  |  |  |
|  | MIR545 |  |  |  |
|  | E2F4 |  |  |  |
|  | NTSR1 |  |  |  |
|  | S100P |  |  |  |
|  | IGFBP5 |  |  |  |
|  | PIK3CD |  |  |  |
|  | IL3 |  |  |  |
|  | SOCS1 |  |  |  |
|  | GSN |  |  |  |
|  | DDIT3 |  |  |  |
|  | PRSS3 |  |  |  |
|  | PTK2B |  |  |  |
|  | MIR367 |  |  |  |
|  | MIR501 |  |  |  |
|  | WNT2 |  |  |  |
|  | MIR494 |  |  |  |
|  | EPO |  |  |  |
|  | SLPI |  |  |  |
|  | MIR339 |  |  |  |
|  | BBC3 |  |  |  |
|  | PRKCB |  |  |  |
|  | KDM4B |  |  |  |
|  | PTPRN |  |  |  |
|  | LSM1 |  |  |  |
|  | MIR877 |  |  |  |
|  | GP2 |  |  |  |
|  | MTDH |  |  |  |
|  | NCOR1 |  |  |  |
|  | SETBP1 |  |  |  |
|  | PIWIL1 |  |  |  |
|  | MIR377 |  |  |  |
|  | RYR1 |  |  |  |
|  | MIR199A2 |  |  |  |
|  | PIP |  |  |  |
|  | CSNK2A1 |  |  |  |
|  | SLC2A2 |  |  |  |
|  | NPY |  |  |  |
|  | MMP13 |  |  |  |
|  | HSD17B1 |  |  |  |
|  | MNX1 |  |  |  |
|  | NKX2-2 |  |  |  |
|  | PRKCE |  |  |  |
|  | ANPEP |  |  |  |
|  | CDH13 |  |  |  |
|  | ALDOA |  |  |  |
|  | MUC5B |  |  |  |
|  | CTRB2 |  |  |  |
|  | TFAP2A |  |  |  |
|  | LAMC2 |  |  |  |
|  | SNHG16 |  |  |  |
|  | SFTPA1 |  |  |  |
|  | HSPD1 |  |  |  |
|  | RPL5 |  |  |  |
|  | MT-ND4L |  |  |  |
|  | SAT1 |  |  |  |
|  | RUNX2 |  |  |  |
|  | LASP1 |  |  |  |
|  | KMT2D |  |  |  |
|  | TSG101 |  |  |  |
|  | NPY4R |  |  |  |
|  | CTTN |  |  |  |
|  | GH1 |  |  |  |
|  | CYP1A2 |  |  |  |
|  | TMEFF2 |  |  |  |
|  | LOC110806263 |  |  |  |
|  | TINF2 |  |  |  |
|  | TFF3 |  |  |  |
|  | IL32 |  |  |  |
|  | NGFR |  |  |  |
|  | LINC01772 |  |  |  |
|  | WNT9A |  |  |  |
|  | MIR31HG |  |  |  |
|  | ROCK1 |  |  |  |
|  | SLX4 |  |  |  |
|  | NUMA1 |  |  |  |
|  | MGAT4A |  |  |  |
|  | MIR509-1 |  |  |  |
|  | IQGAP1 |  |  |  |
|  | FGF3 |  |  |  |
|  | GPC1 |  |  |  |
|  | WT1-AS |  |  |  |
|  | AURKB |  |  |  |
|  | IRAIN |  |  |  |
|  | AATBC |  |  |  |
|  | CDH17 |  |  |  |
|  | PENK |  |  |  |
|  | LRP6 |  |  |  |
|  | LGALS3BP |  |  |  |
|  | MIR154 |  |  |  |
|  | TKT |  |  |  |
|  | ITGA2 |  |  |  |
|  | EPAS1 |  |  |  |
|  | BTK |  |  |  |
|  | DIRC3 |  |  |  |
|  | CEACAM1 |  |  |  |
|  | ANGPT1 |  |  |  |
|  | TH |  |  |  |
|  | MIR216B |  |  |  |
|  | LNCOC1 |  |  |  |
|  | MIR671 |  |  |  |
|  | MIR125B2 |  |  |  |
|  | CTBP2 |  |  |  |
|  | UGT1A1 |  |  |  |
|  | PLCG2 |  |  |  |
|  | NUTM1 |  |  |  |
|  | SFRP1 |  |  |  |
|  | NR3C1 |  |  |  |
|  | F3 |  |  |  |
|  | PNP |  |  |  |
|  | IDO1 |  |  |  |
|  | ABCB5 |  |  |  |
|  | MIAT |  |  |  |
|  | POLG |  |  |  |
|  | MIR628 |  |  |  |
|  | TNFSF11 |  |  |  |
|  | MIR188 |  |  |  |
|  | SLC26A9 |  |  |  |
|  | PVR |  |  |  |
|  | CD46 |  |  |  |
|  | FAT2 |  |  |  |
|  | KDM5B |  |  |  |
|  | PRMT7 |  |  |  |
|  | MIR129-1 |  |  |  |
|  | NR1H2 |  |  |  |
|  | KEAP1 |  |  |  |
|  | FRGCA |  |  |  |
|  | PPP2R2A |  |  |  |
|  | GPR68 |  |  |  |
|  | NTRK2 |  |  |  |
|  | SYK |  |  |  |
|  | GLP1R |  |  |  |
|  | MIR584 |  |  |  |
|  | EREG |  |  |  |
|  | SELP |  |  |  |
|  | TNFRSF10D |  |  |  |
|  | EXO1 |  |  |  |
|  | REST |  |  |  |
|  | MIR181D |  |  |  |
|  | FCGR3A |  |  |  |
|  | PHOX2B |  |  |  |
|  | HOXB2 |  |  |  |
|  | SSTR5 |  |  |  |
|  | AFAP1 |  |  |  |
|  | F2R |  |  |  |
|  | IGFBP1 |  |  |  |
|  | MADD |  |  |  |
|  | WNT4 |  |  |  |
|  | MIR378A |  |  |  |
|  | STK4 |  |  |  |
|  | MMUT |  |  |  |
|  | TIGIT |  |  |  |
|  | SERPINB2 |  |  |  |
|  | SLC9A3 |  |  |  |
|  | TUBB |  |  |  |
|  | ACP3 |  |  |  |
|  | ADA |  |  |  |
|  | NOG |  |  |  |
|  | PPARA |  |  |  |
|  | JAZF1 |  |  |  |
|  | MIR491 |  |  |  |
|  | TFRC |  |  |  |
|  | REG4 |  |  |  |
|  | SOS2 |  |  |  |
|  | REG1B |  |  |  |
|  | YTHDC2 |  |  |  |
|  | CCN1 |  |  |  |
|  | PTH |  |  |  |
|  | MIR432 |  |  |  |
|  | AMACR |  |  |  |
|  | WNT2B |  |  |  |
|  | PDGFA |  |  |  |
|  | LDHA |  |  |  |
|  | ACTA2 |  |  |  |
|  | HLA-C |  |  |  |
|  | PLXNA1 |  |  |  |
|  | TCF7 |  |  |  |
|  | RPS10 |  |  |  |
|  | BGLAP |  |  |  |
|  | BRMS1L |  |  |  |
|  | ZFR |  |  |  |
|  | PEG10 |  |  |  |
|  | ITGB3 |  |  |  |
|  | MFN2 |  |  |  |
|  | EIF4E |  |  |  |
|  | MIR876 |  |  |  |
|  | TLR5 |  |  |  |
|  | CD40LG |  |  |  |
|  | MIR409 |  |  |  |
|  | TSHR |  |  |  |
|  | TACSTD2 |  |  |  |
|  | SUZ12 |  |  |  |
|  | SELE |  |  |  |
|  | SFTPC |  |  |  |
|  | SOCS3 |  |  |  |
|  | SEPTIN9 |  |  |  |
|  | FGF8 |  |  |  |
|  | RPL15 |  |  |  |
|  | SOX2-OT |  |  |  |
|  | DLL4 |  |  |  |
|  | CCDC26 |  |  |  |
|  | TMPRSS9 |  |  |  |
|  | MT-TL1 |  |  |  |
|  | CEBPB |  |  |  |
|  | PRMT1 |  |  |  |
|  | CLDN3 |  |  |  |
|  | TCF3 |  |  |  |
|  | PTGER4 |  |  |  |
|  | NANOG |  |  |  |
|  | YWHAE |  |  |  |
|  | MKS1 |  |  |  |
|  | H4C16 |  |  |  |
|  | PRKAA1 |  |  |  |
|  | ROR1 |  |  |  |
|  | APOB |  |  |  |
|  | CEMIP |  |  |  |
|  | DDR1 |  |  |  |
|  | XRCC5 |  |  |  |
|  | CTCF |  |  |  |
|  | MAPK9 |  |  |  |
|  | TOX3 |  |  |  |
|  | XRCC6 |  |  |  |
|  | PSG2 |  |  |  |
|  | MIR30C1 |  |  |  |
|  | COL18A1 |  |  |  |
|  | EXT2 |  |  |  |
|  | CSNK1A1 |  |  |  |
|  | HSPA1A |  |  |  |
|  | MDK |  |  |  |
|  | PANDAR |  |  |  |
|  | CD86 |  |  |  |
|  | ABCB11 |  |  |  |
|  | MED12 |  |  |  |
|  | RHEB |  |  |  |
|  | FGF21 |  |  |  |
|  | TRPM8 |  |  |  |
|  | GRN |  |  |  |
|  | TTR |  |  |  |
|  | SEL1L |  |  |  |
|  | CSNK2B |  |  |  |
|  | H3-3A |  |  |  |
|  | DDX41 |  |  |  |
|  | CYP27B1 |  |  |  |
|  | GAD1 |  |  |  |
|  | RPS15 |  |  |  |
|  | MIR383 |  |  |  |
|  | ARID4B |  |  |  |
|  | HIC1 |  |  |  |
|  | MIR26B |  |  |  |
|  | GRB7 |  |  |  |
|  | HDGF |  |  |  |
|  | CELA2A |  |  |  |
|  | LZTR1 |  |  |  |
|  | RACK1 |  |  |  |
|  | FGF4 |  |  |  |
|  | PSMB8 |  |  |  |
|  | WNT6 |  |  |  |
|  | MTR |  |  |  |
|  | ABCC3 |  |  |  |
|  | NOS3 |  |  |  |
|  | APC2 |  |  |  |
|  | FURIN |  |  |  |
|  | CPA2 |  |  |  |
|  | NOP14 |  |  |  |
|  | WNT9B |  |  |  |
|  | AGER |  |  |  |
|  | TNK2 |  |  |  |
|  | INHA |  |  |  |
|  | DCTN4 |  |  |  |
|  | MAP2K3 |  |  |  |
|  | CUL1 |  |  |  |
|  | CLIC4 |  |  |  |
|  | AGR2 |  |  |  |
|  | CD19 |  |  |  |
|  | LBR |  |  |  |
|  | GUSB |  |  |  |
|  | TCHP |  |  |  |
|  | LTA |  |  |  |
|  | THY1 |  |  |  |
|  | SRSF2 |  |  |  |
|  | TGIF1 |  |  |  |
|  | CCNA1 |  |  |  |
|  | SSX1 |  |  |  |
|  | PIM1 |  |  |  |
|  | MIR660 |  |  |  |
|  | TUBA1A |  |  |  |
|  | BMP7 |  |  |  |
|  | CSK |  |  |  |
|  | C16orf74 |  |  |  |
|  | NRP2 |  |  |  |
|  | CEACAM7 |  |  |  |
|  | CIP2A |  |  |  |
|  | ARX |  |  |  |
|  | KLK6 |  |  |  |
|  | PAK4 |  |  |  |
|  | PLCB4 |  |  |  |
|  | IFNB1 |  |  |  |
|  | COMT |  |  |  |
|  | TP53BP1 |  |  |  |
|  | SIRT3 |  |  |  |
|  | ELF3 |  |  |  |
|  | NNMT |  |  |  |
|  | EPOR |  |  |  |
|  | SPINT1 |  |  |  |
|  | KLF12 |  |  |  |
|  | PIN1 |  |  |  |
|  | INHBA |  |  |  |
|  | GNB1 |  |  |  |
|  | RHOC |  |  |  |
|  | RBX1 |  |  |  |
|  | PTPN13 |  |  |  |
|  | NOTCH4 |  |  |  |
|  | PRC1 |  |  |  |
|  | BRDT |  |  |  |
|  | RPL11 |  |  |  |
|  | G6PC1 |  |  |  |
|  | MIR184 |  |  |  |
|  | ELN |  |  |  |
|  | PRLR |  |  |  |
|  | VEGFB |  |  |  |
|  | IL4R |  |  |  |
|  | GADD45B |  |  |  |
|  | CLCA4 |  |  |  |
|  | PTTG1 |  |  |  |
|  | ROBO1 |  |  |  |
|  | HSPA8 |  |  |  |
|  | PRKDC |  |  |  |
|  | XRCC4 |  |  |  |
|  | EPHA5 |  |  |  |
|  | GPX1 |  |  |  |
|  | GFRA1 |  |  |  |
|  | ABCA1 |  |  |  |
|  | PCM1 |  |  |  |
|  | BAG1 |  |  |  |
|  | MUC3A |  |  |  |
|  | RPS27 |  |  |  |
|  | AKR1C3 |  |  |  |
|  | GSE1 |  |  |  |
|  | MIR489 |  |  |  |
|  | COL11A1 |  |  |  |
|  | FOXC1 |  |  |  |
|  | RRAS |  |  |  |
|  | UBE2T |  |  |  |
|  | VWF |  |  |  |
|  | DSP |  |  |  |
|  | PTK6 |  |  |  |
|  | RBBP4 |  |  |  |
|  | TRAF6 |  |  |  |
|  | CCNH |  |  |  |
|  | FLNB |  |  |  |
|  | CHKA |  |  |  |
|  | HPGD |  |  |  |
|  | NCOA6 |  |  |  |
|  | TP53BP2 |  |  |  |
|  | CP |  |  |  |
|  | OGFR |  |  |  |
|  | MIR7-1 |  |  |  |
|  | ADAR |  |  |  |
|  | TUSC7 |  |  |  |
|  | STK33 |  |  |  |
|  | RPSA |  |  |  |
|  | AGPAT2 |  |  |  |
|  | E2F5 |  |  |  |
|  | ANO1 |  |  |  |
|  | KLK4 |  |  |  |
|  | NCOA2 |  |  |  |
|  | CASP2 |  |  |  |
|  | TXN |  |  |  |
|  | PGF |  |  |  |
|  | LIG1 |  |  |  |
|  | BMP8B |  |  |  |
|  | NCR3LG1 |  |  |  |
|  | PTCH2 |  |  |  |
|  | ROR2 |  |  |  |
|  | C3 |  |  |  |
|  | HDAC6 |  |  |  |
|  | DIS3L2 |  |  |  |
|  | PRKCI |  |  |  |
|  | TDGF1 |  |  |  |
|  | TIMELESS |  |  |  |
|  | WFS1 |  |  |  |
|  | EIF2AK2 |  |  |  |
|  | AKR1B10 |  |  |  |
|  | PBRM1 |  |  |  |
|  | TLR3 |  |  |  |
|  | PKD2 |  |  |  |
|  | UCHL1 |  |  |  |
|  | MIR495 |  |  |  |
|  | LAMB3 |  |  |  |
|  | CLCA2 |  |  |  |
|  | PTPN22 |  |  |  |
|  | ITGAM |  |  |  |
|  | LOC107303338 |  |  |  |
|  | HNRNPK |  |  |  |
|  | PRTN3 |  |  |  |
|  | CTSL |  |  |  |
|  | WEE1 |  |  |  |
|  | EPRS1 |  |  |  |
|  | PMAIP1 |  |  |  |
|  | DVL1 |  |  |  |
|  | RXRB |  |  |  |
|  | TMPRSS4 |  |  |  |
|  | JAK3 |  |  |  |
|  | ENPP2 |  |  |  |
|  | XPO1 |  |  |  |
|  | TPM3 |  |  |  |
|  | FZD5 |  |  |  |
|  | EFNA1 |  |  |  |
|  | LIN28B |  |  |  |
|  | F5 |  |  |  |
|  | GIPC1 |  |  |  |
|  | LEPR |  |  |  |
|  | MST1 |  |  |  |
|  | ENPP1 |  |  |  |
|  | NCR3 |  |  |  |
|  | HLA-G |  |  |  |
|  | NCOA4 |  |  |  |
|  | HP |  |  |  |
|  | FZD7 |  |  |  |
|  | MIR218-1 |  |  |  |
|  | TRIM24 |  |  |  |
|  | SNHG12 |  |  |  |
|  | FGA |  |  |  |
|  | BANCR |  |  |  |
|  | SELENBP1 |  |  |  |
|  | THBD |  |  |  |
|  | IL5 |  |  |  |
|  | CLDN18 |  |  |  |
|  | SLC9A3R1 |  |  |  |
|  | MIR7-3 |  |  |  |
|  | NAMPT |  |  |  |
|  | MEFV |  |  |  |
|  | HSF1 |  |  |  |
|  | CYP24A1 |  |  |  |
|  | MIR95 |  |  |  |
|  | ELOC |  |  |  |
|  | OFD1 |  |  |  |
|  | DUSP1 |  |  |  |
|  | VCAM1 |  |  |  |
|  | LYVE1 |  |  |  |
|  | MGAT4B |  |  |  |
|  | UCP2 |  |  |  |
|  | CTRL |  |  |  |
|  | IFNGR1 |  |  |  |
|  | AMY2B |  |  |  |
|  | BCAR4 |  |  |  |
|  | BCPR |  |  |  |
|  | BLACAT1 |  |  |  |
|  | CASC1 |  |  |  |
|  | CASC15 |  |  |  |
|  | CASC21 |  |  |  |
|  | CASC8 |  |  |  |
|  | CASP10 |  |  |  |
|  | CDK2AP1 |  |  |  |
|  | CHRNA3 |  |  |  |
|  | CHRNA5 |  |  |  |
|  | COLCA1 |  |  |  |
|  | COLCA2 |  |  |  |
|  | CRCS2 |  |  |  |
|  | CRCS5 |  |  |  |
|  | CRCS6 |  |  |  |
|  | CRCS7 |  |  |  |
|  | CRCS8 |  |  |  |
|  | CRCS9 |  |  |  |
|  | CTAG3 |  |  |  |
|  | DIRC1 |  |  |  |
|  | DLEC1 |  |  |  |
|  | EHBP1 |  |  |  |
|  | EIF4G1 |  |  |  |
|  | FALEC |  |  |  |
|  | GACAT3 |  |  |  |
|  | GAEC1 |  |  |  |
|  | GCRG224 |  |  |  |
|  | HEPN1 |  |  |  |
|  | HMPS1 |  |  |  |
|  | HPC10 |  |  |  |
|  | HPC11 |  |  |  |
|  | HPC14 |  |  |  |
|  | HPC4 |  |  |  |
|  | HPC5 |  |  |  |
|  | HPC7 |  |  |  |
|  | HPC9 |  |  |  |
|  | HPCQTL19 |  |  |  |
|  | KMHN1 |  |  |  |
|  | LCO |  |  |  |
|  | LNCR1 |  |  |  |
|  | LNCR3 |  |  |  |
|  | LNCR4 |  |  |  |
|  | LNCR5 |  |  |  |
|  | MAD1L1 |  |  |  |
|  | ORAOV1 |  |  |  |
|  | OVCAS1 |  |  |  |
|  | PBCA |  |  |  |
|  | PBOV1 |  |  |  |
|  | PCAP |  |  |  |
|  | PCAT1 |  |  |  |
|  | PCAT18 |  |  |  |
|  | PCAT19 |  |  |  |
|  | PCAT2 |  |  |  |
|  | PCAT29 |  |  |  |
|  | PCAT4 |  |  |  |
|  | PNLIPRP2 |  |  |  |
|  | PPY2 |  |  |  |
|  | PPYR1 |  |  |  |
|  | PRNCR1 |  |  |  |
|  | SASH1 |  |  |  |
|  | SCAI |  |  |  |
|  | SCHLAP1 |  |  |  |
|  | SCLC1 |  |  |  |
|  | SLC22A1L |  |  |  |
|  | TSG11 |  |  |  |
|  | VOPP1 |  |  |  |
|  | ACH |  |  |  |
|  | ACSTD1 |  |  |  |
|  | ACVRLK4 |  |  |  |
|  | ADH2 |  |  |  |
|  | ALPS4 |  |  |  |
|  | ATA |  |  |  |
|  | ATBF1 |  |  |  |
|  | AVSD5 |  |  |  |
|  | BACH1 |  |  |  |
|  | BCSG1 |  |  |  |
|  | BEK |  |  |  |
|  | BHD |  |  |  |
|  | BIT1 |  |  |  |
|  | BUBR1 |  |  |  |
|  | BWSCR1A |  |  |  |
|  | C9orf126 |  |  |  |
|  | CAGE1 |  |  |  |
|  | CARLO5 |  |  |  |
|  | CC1 |  |  |  |
|  | CKN2 |  |  |  |
|  | CLOVE |  |  |  |
|  | CMM6 |  |  |  |
|  | COCA1 |  |  |  |
|  | COCA2 |  |  |  |
|  | COPEB |  |  |  |
|  | COT |  |  |  |
|  | CRAC1 |  |  |  |
|  | CRCS1 |  |  |  |
|  | CRCS12 |  |  |  |
|  | CWS6 |  |  |  |
|  | CYP2A3 |  |  |  |
|  | DD3 |  |  |  |
|  | DEP1 |  |  |  |
|  | DIA4 |  |  |  |
|  | DLC1 |  |  |  |
|  | DOC1 |  |  |  |
|  | ECOP |  |  |  |
|  | EIF4G |  |  |  |
|  | EMS1 |  |  |  |
|  | EPHT3 |  |  |  |
|  | ESR |  |  |  |
|  | FAL1 |  |  |  |
|  | FANCD1 |  |  |  |
|  | FANCN |  |  |  |
|  | FANCO |  |  |  |
|  | FKHL15 |  |  |  |
|  | FRP1 |  |  |  |
|  | GDEP |  |  |  |
|  | GS |  |  |  |
|  | GTBP |  |  |  |
|  | HLRCC |  |  |  |
|  | HLXB9 |  |  |  |
|  | HNPCC6 |  |  |  |
|  | HNPCC7 |  |  |  |
|  | HPC13 |  |  |  |
|  | HPC2 |  |  |  |
|  | HPC9 |  |  |  |
|  | HR54 |  |  |  |
|  | IPF1 |  |  |  |
|  | IRHOM2 |  |  |  |
|  | JCK |  |  |  |
|  | KIAA0790 |  |  |  |
|  | KIAA0903 |  |  |  |
|  | KIAA0992 |  |  |  |
|  | KIAA1304 |  |  |  |
|  | KRAS2 |  |  |  |
|  | LAS1 |  |  |  |
|  | LINC00178 |  |  |  |
|  | LINC00340 |  |  |  |
|  | LINC00860 |  |  |  |
|  | LINC00912 |  |  |  |
|  | LINC00990 |  |  |  |
|  | LINC01092 |  |  |  |
|  | LINC01190 |  |  |  |
|  | LINC01244 |  |  |  |
|  | LINC01245 |  |  |  |
|  | LINC01458 |  |  |  |
|  | LNCR2 |  |  |  |
|  | MADH4 |  |  |  |
|  | MADH7 |  |  |  |
|  | MAR |  |  |  |
|  | MCH4 |  |  |  |
|  | MCH5 |  |  |  |
|  | MMAC1 |  |  |  |
|  | MRMV1 |  |  |  |
|  | MTCL1AS1 |  |  |  |
|  | MTS1 |  |  |  |
|  | MVCD4 |  |  |  |
|  | MYH |  |  |  |
|  | NEDSDV |  |  |  |
|  | NGL |  |  |  |
|  | NISBD2 |  |  |  |
|  | NMOR2 |  |  |  |
|  | NOT1 |  |  |  |
|  | NPH3 |  |  |  |
|  | NPY4R |  |  |  |
|  | NS7 |  |  |  |
|  | ODCRCS |  |  |  |
|  | P53 |  |  |  |
|  | PACA |  |  |  |
|  | PARK2 |  |  |  |
|  | PCA2 |  |  |  |
|  | PCAT114 |  |  |  |
|  | PCAT8 |  |  |  |
|  | PDGRL |  |  |  |
|  | PHBP |  |  |  |
|  | PJS |  |  |  |
|  | PLA2A |  |  |  |
|  | PLA2B |  |  |  |
|  | PLRP1 |  |  |  |
|  | PLRP2 |  |  |  |
|  | PMSL2 |  |  |  |
|  | PNCA5 |  |  |  |
|  | PNLIPD |  |  |  |
|  | PRAD1 |  |  |  |
|  | PSCP |  |  |  |
|  | PSPS1 |  |  |  |
|  | PSTI |  |  |  |
|  | PTPG1 |  |  |  |
|  | RAD51L3 |  |  |  |
|  | RECA |  |  |  |
|  | RIM |  |  |  |
|  | RNF124 |  |  |  |
|  | RNS1 |  |  |  |
|  | RNS4 |  |  |  |
|  | SAR1 |  |  |  |
|  | SCAL1 |  |  |  |
|  | SH2D3B |  |  |  |
|  | TAOS1 |  |  |  |
|  | TIL4 |  |  |  |
|  | TITF1 |  |  |  |
|  | TNFSF6 |  |  |  |
|  | TXBP181 |  |  |  |
|  | UROC28 |  |  |  |
|  | UVO |  |  |  |
|  | WIP1 |  |  |  |
|  | YARS |  |  |  |
|  | AAT3 |  |  |  |
|  | ALK4 |  |  |  |
|  | ALPS2 |  |  |  |
|  | ALPS2B |  |  |  |
|  | APT1LG1 |  |  |  |
|  | ASD9 |  |  |  |
|  | AT1 |  |  |  |
|  | BCD1 |  |  |  |
|  | BCL1 |  |  |  |
|  | BROVCA1 |  |  |  |
|  | BROVCA2 |  |  |  |
|  | BROVCA3 |  |  |  |
|  | BROVCA4 |  |  |  |
|  | CAPOK |  |  |  |
|  | CARLO1 |  |  |  |
|  | CARLO2 |  |  |  |
|  | CARLO4 |  |  |  |
|  | CARLO6 |  |  |  |
|  | CARLO7 |  |  |  |
|  | CFD1 |  |  |  |
|  | CMTDIC |  |  |  |
|  | COFS1 |  |  |  |
|  | COXPD17 |  |  |  |
|  | CRCS3 |  |  |  |
|  | CRCS4 |  |  |  |
|  | CUDR |  |  |  |
|  | CYP2A |  |  |  |
|  | DIRA |  |  |  |
|  | DPC4 |  |  |  |
|  | DRT |  |  |  |
|  | EST |  |  |  |
|  | ESTRR |  |  |  |
|  | EVR7 |  |  |  |
|  | FANCJ |  |  |  |
|  | FCC1 |  |  |  |
|  | FILS |  |  |  |
|  | FPC |  |  |  |
|  | GASP |  |  |  |
|  | GLM2 |  |  |  |
|  | HGFAL |  |  |  |
|  | HGPPS2 |  |  |  |
|  | HNPCC2 |  |  |  |
|  | HNPCC4 |  |  |  |
|  | HNPCC5 |  |  |  |
|  | HOXHB9 |  |  |  |
|  | HPC12 |  |  |  |
|  | HPE12 |  |  |  |
|  | HRAD54 |  |  |  |
|  | IMNEPD |  |  |  |
|  | IMPT1 |  |  |  |
|  | JDVS |  |  |  |
|  | KIAA0203 |  |  |  |
|  | LCAM |  |  |  |
|  | LFS1 |  |  |  |
|  | LKB1 |  |  |  |
|  | MCAP |  |  |  |
|  | MCUL1 |  |  |  |
|  | MODY4 |  |  |  |
|  | MRMV2 |  |  |  |
|  | MVA1 |  |  |  |
|  | NEU |  |  |  |
|  | NKX2A |  |  |  |
|  | NMOR1 |  |  |  |
|  | NMTC2 |  |  |  |
|  | NPHP9 |  |  |  |
|  | NS6 |  |  |  |
|  | NSP2 |  |  |  |
|  | P16 |  |  |  |
|  | PAGEN2 |  |  |  |
|  | PAOD2 |  |  |  |
|  | PARK18 |  |  |  |
|  | PCTT |  |  |  |
|  | PDJ |  |  |  |
|  | PLA2 |  |  |  |
|  | PLA2L |  |  |  |
|  | PNCA1 |  |  |  |
|  | PNCA3 |  |  |  |
|  | PP1 |  |  |  |
|  | PPP1R54 |  |  |  |
|  | PRCA1 |  |  |  |
|  | PRLTS |  |  |  |
|  | RASK2 |  |  |  |
|  | REG |  |  |  |
|  | RHPD1 |  |  |  |
|  | SCKL1 |  |  |  |
|  | SCKL2 |  |  |  |
|  | SSPCS |  |  |  |
|  | TITF2 |  |  |  |
|  | TOC |  |  |  |
|  | TROP1 |  |  |  |
|  | UC28 |  |  |  |
|  | BAIPRCK |  |  |  |
|  | BCC7 |  |  |  |
|  | BTPS2 |  |  |  |
|  | CMNS |  |  |  |
|  | CSB |  |  |  |
|  | CWS1 |  |  |  |
|  | DUH1 |  |  |  |
|  | DUP15q |  |  |  |
|  | ECAD |  |  |  |
|  | ERK |  |  |  |
|  | FANCR |  |  |  |
|  | FASL |  |  |  |
|  | FCTCS |  |  |  |
|  | FMRD |  |  |  |
|  | FSAP |  |  |  |
|  | GLM3 |  |  |  |
|  | HER2 |  |  |  |
|  | HNPCC1 |  |  |  |
|  | HPC1 |  |  |  |
|  | IMAGEI |  |  |  |
|  | JIP |  |  |  |
|  | JWDS |  |  |  |
|  | JWS |  |  |  |
|  | M4S1 |  |  |  |
|  | MCM |  |  |  |
|  | MFS2 |  |  |  |
|  | MKS7 |  |  |  |
|  | MLM |  |  |  |
|  | MMRCS1 |  |  |  |
|  | MMRCS3 |  |  |  |
|  | MMRCS4 |  |  |  |
|  | MOM1 |  |  |  |
|  | NS |  |  |  |
|  | P450C2A |  |  |  |
|  | PACHD |  |  |  |
|  | PAGEN1 |  |  |  |
|  | PNCA4 |  |  |  |
|  | PPLA2 |  |  |  |
|  | RHPD2 |  |  |  |
|  | SCRA1 |  |  |  |
|  | TATI |  |  |  |
|  | TPL2 |  |  |  |
|  | TTF1 |  |  |  |
|  | TTF2 |  |  |  |
|  | TYRRS |  |  |  |
|  | VIBOS |  |  |  |
|  | ZF9 |  |  |  |
|  | ALPS1B |  |  |  |
|  | ARMD5 |  |  |  |
|  | BCDS1 |  |  |  |
|  | BMFS5 |  |  |  |
|  | C15DUPq |  |  |  |
|  | CFC2 |  |  |  |
|  | CMM2 |  |  |  |
|  | DESMD |  |  |  |
|  | FANCS |  |  |  |
|  | LDS2 |  |  |  |
|  | MCMTC |  |  |  |
|  | MIC18 |  |  |  |
|  | MMRCS2 |  |  |  |
|  | MYHRS |  |  |  |
|  | NCMS |  |  |  |
|  | NMTC1 |  |  |  |
|  | NMTC4 |  |  |  |
|  | NMTC5 |  |  |  |
|  | PCBC |  |  |  |
|  | PNCA2 |  |  |  |
|  | TCP |  |  |  |
|  | TK14 |  |  |  |
|  | VSCN2 |  |  |  |
|  | YTS |  |  |  |
|  | BBDS |  |  |  |
|  | CAPB |  |  |  |
|  | CWS5 |  |  |  |
|  | DIAR5 |  |  |  |
|  | RALD |  |  |  |
|  | UVSS1 |  |  |  |
|  | YRS |  |  |  |
|  | BDPLT22 |  |  |  |
|  | CLAPO |  |  |  |
|  | HNPCC8 |  |  |  |
|  | IMNEPD2 |  |  |  |
|  | OES |  |  |  |
|  | POF11 |  |  |  |
|  | CCM4 |  |  |  |
